# Supplementary material for: Digestive tolerability and acceptability of Fibersol-2 in healthy and diarrheal children 1–3 years old at a rural facility, Bangladesh: Results from a four arm exploratory study
Source: PLoS One. 2022 Sep 19;17(9):e0274302. doi: 10.1371/journal.pone.0274302 (PMC9484693; doi:10.1371/journal.pone.0274302)
Supplement: S1 Table — (DOCX) [file pone.0274302.s002.docx]

**Table S1. Comparison of socio-demographic characteristics among the healthy and diarrheal children with low (2.5 gm) and high (5 gm) doses of Fibersol-2**

| **Variable of interest** | **Healthy/low dose**  **(n=15) (%)** | **Healthy/high dose**  **(n=15) (%)** | **Diarrheal/low dose**  **(n=15) (%)** | **Diarrheal/ high dose**  **(n=15) (%)** | **p-value** |
| --- | --- | --- | --- | --- | --- |
| Male sex | 5 (33.3) | 9 (60.0) | 7 (46.7) | 10 (66.7) | 0.268 |
| Family size (Median, IQR) | 4 (3, 6) | 5 (4, 7) | 4 (3, 6) | 5 (4, 6) | 0.208 |
| Number of sleeping rooms (Median, IQR) | 2 (1, 3) | 2 (2, 3) | 2 (1, 3) | 2 (2, 3) | 0.662 |
| Maternal illiteracy | 0 (0) | 3 (20.0) | 1 (6.7) | 0 (0) | 0.179 |
| Paternal illiteracy | 0 (0) | 2 (13.3) | 2 (13.3) | 1 (6.7) | 0.740 |
| Use of non-sanitary toilet facility | 3 (20.0) | 5 (33.3) | 5 (33.3) | 5 (33.3) | 0.836 |
| Household had no electricity | 0 (0) | 0 (0) | 1 (6.7) | 0 (0) | 1.000 |
| Deep tube well | 7 (53.3) | 10 (66.7) | 7 (53.3) | 3 (20.0) | 0.083 |
| **Wealth quintile** |  |  |  |  |  |
| Poorest | 1 (6.7) | 4 (26.7) | 3 (20.0) | 4 (26.7) | 0.498 |
| Lower middle | 5 (33.3) | 1 (6.7) | 5 (33.3) | 1 (6.7) | 0.113 |
| Middle | 3 (20.0) | 4 (26.7) | 2 (13.3) | 3 (20.0) | 0.969 |
| Upper middle | 4 (26.7) | 2 (13.3) | 2 (13.3) | 4 (26.7) | 0.715 |
| Rich | 2 (13.3) | 4 (26.7) | 3 (20.0) | 3 (20.0) | 0.969 |

All continuous variables are tested with two-way ANOVA / Kruskal–Wallis test; IQR, Inter- quartile range
