# Supplementary material for: Digestive tolerability and acceptability of Fibersol-2 in healthy and diarrheal children 1–3 years old at a rural facility, Bangladesh: Results from a four arm exploratory study
Source: PLoS One. 2022 Sep 19;17(9):e0274302. doi: 10.1371/journal.pone.0274302 (PMC9484693; doi:10.1371/journal.pone.0274302)
Supplement: S2 Table — (DOCX) [file pone.0274302.s003.docx]

**Table S2. Comparison of characteristics of healthy and diarrheal children with low (2.5 gm) and high (5 gm) doses of Fibersol-2 on last day**

| **Variable of interest** | **Healthy/ low dose**  **(n=15) (%)** | **Healthy/ high dose**  **(n=15) (%)** | **Diarrheal/low dose**  **(n=15) (%)** | **Diarrheal/ high dose**  **(n=15) (%)** | **p-value** |
| --- | --- | --- | --- | --- | --- |
| Height in cm (mean, SD) | 83.0±5.8 | 82.7±6.5 | 80.3±4.2 | 81.1±6.8 | 0.373 |
| Weight in kg (mean, SD) | 10.9±1.1 | 11.1±2.2 | 10.3±1.1 | 11.0±2.8 | 0.514 |
| Mid upper arm circumference rooms (mean, SD) | 15.1±0.8 | 15.4±1.2 | 15.1±0.9 | 15.2±1.7 | 0.705 |
| Stool became formed in days ( median, IQR) | - | - | 3.9 (2.9, 5.1) | 3.5 (2.0, 8.0) | 0.885 |
| **Nutritional status** |  |  |  |  |  |
| Height-for-age z-score (mean, SD) | -1.43±0.79 | -1.84±0.78 | -1.06±1.06 | -1.16±1.51 | 0.113 |
| Weight-for-length/height z-score (mean, SD) | -0.08±0.75 | 0.05±1.04 | -0.13±0.70 | -0.16±1.43 | 0.715 |
| Weight-for-age z-score (mean, SD) | -0.81±0.91 | -0.93±1.01 | -0.63±0.95 | -0.45±1.71 | 0.567 |
| Stunting | 4 (26.7) | 7 (46.7) | 3 (20.0) | 4 (26.7) | 0.540 |
| Underweight | 1 (6.7) | 2 (13.3) | 2 (13.3) | 3 (20.0) | 0.952 |
| Wasting | 0 (0) | 0 (0) | 0 (0) | 1 (6.7) | 1.000 |

All continuous variables are tested with two-way ANOVA / Kruskal–Wallis test; SD, Standard deviation; IQR, Inter-quartile range
