# Supplementary material for: Digestive tolerability and acceptability of Fibersol-2 in healthy and diarrheal children 1–3 years old at a rural facility, Bangladesh: Results from a four arm exploratory study
Source: PLoS One. 2022 Sep 19;17(9):e0274302. doi: 10.1371/journal.pone.0274302 (PMC9484693; doi:10.1371/journal.pone.0274302)
Supplement: S1 Questionnaire — (PDF) [file pone.0274302.s004.pdf]

icddr,b	RRC APPLICATION FORM

RESEARCH PROTOCOL
Number: PR-16091
Version No. 1.4
Version date: 20-02-2017

FOR OFFICE USE ONLY

RRC Approval:	☒ Yes	☐ No	Date:22.01.2017
ERC Approval:	☐ Yes	☐ No	Date: 20.03.2017
AEEC Approval:	☐ Yes	☐ No	Date:
External IRB Approval	☐ Yes	☐ No	Date:
Name of External IRB: ______________________________			

Protocol Title:* (maximum 250 characters including space**)**Tolerability and Acceptability of Fibersol-2 (Resistant Maltodextrin) in healthyand diarrheal children followed by a randomized clinical trial to evaluate the efficacy of Fibersol-2 in diarrheal children 1-3 years old

Short Title: (maximum **100**characters including space**)**Tolerability and Acceptability of Fibersol-2 in Children

Key Words:*Tolerability, Acceptability, Fibersol-2, Children

Name of theResearch Division Hosting theProtocol:*

☐Health Systems and Population Studies Division (HSPSD)
☒Nutrition and Clinical Services Division (NCSD)
☐Infectious Diseases Division (IDD)
☐Maternal and Child Health Division (MCHD)
☐Laboratory Sciences and Services Division (LSSD)
☐ Other (specify)

Has the Protocol been Derived from an Activity:*☐ No ☐ Yes (please provide following information):

Activity No. :
Activity Title:
PI:
Grant No.: Budget Code: Start Date: End Date:

icddr,b Strategic Priority/ Initiative (SP 2015-8):* (check all that apply**)**

☐Reducing maternal and neonatal mortality
☒Controlling enteric and respiratory infections
☒Preventing and treating maternal and childhood malnutrition
☐Detecting and controlling emerging and re-emerging infections
☐Achieving universal health coverage
☐Examining the health consequences of climate change
☐Preventing and treating non-communicable diseases

Research Phase (4 Ds):*(check all that apply**)**

☐ Discovery
☒ Development
☒ Delivery
☐ Evaluation of Delivery

Anticipated Impact of Research:* (check all that apply**)**

☒ Knowledge Production
☐ Capacity Building
☒ Informing Policy
☐ Health and Health Sector Benefits
☐ Economic Benefits

Which of the Sustainable Development Goal This Protocol Relates to?:*Goal 2: No Hunger: [End hunger, achieve food security and improved nutrition and promote sustainable agriculture]

(Please visit:http://www.icddrb.net.bd/jahia/Jahia/....... for selecting SDG Code(s)

☒ Yes (please select SDGs)
☐ No

Does this Protocol Use the Gender Framework:*
(Please visit:http://www.icddrb.net.bd/jahia/Jahia/pid/684 for Gender Alanysis Tool with instructions)

☐ Yes (please complete Gender Analysis Tool)
☒ No

If 'no' is the response, its reason(s) in brief: This is a clinical trial in the rural area, where we will enrollchildren irrespective of sex.

Project Summary
[The summary, within a word limit of 300, should be stand alone and be fully understandable.]

Principal Investigator: Dr. Mohammod Jobayer Chisti
Research Protocol Title: Tolerability and Acceptability of Fibersol-2 (Resistant Maltodextrin) in healthyand diarrhealchildren followed by a randomized clinical trial to evaluate the efficacy of Fibersol-2 in diarrheal children 1-3 years old

Background (brief):

a**. Burden:** Dietary fibre, a non-digestible carbohydrate, has been used in decades for the beneficial effect on health with physiological importance because such compounds have low energy values. These indigestible carbohydrates generally reach the large intestine undigested and unabsorbed, they are often used in many functional and/or low-calorie food and beverages. Although, dietary fibre has a lot of benefit even in Europe and USA the consumption level is only 50-60% of the recommended level due to increased consumption of processed and pre-cooked food. Researchers have stated that dietary fibre especially digestive-resistant maltodextrin has innumerable beneficial effects on human health, such as improving intestinal regularity by increasing faecal bulk, stimulating peristalsis and shortening gastrointestinal transit time. Resistant maltodextrin (Fibersol-2) is a low viscosity, water-soluble, indigestible dextrin produced by the treatment of cornstarch with acid, enzymes, and heat. Resistant maltodextrin also have prebiotic activity. Prebiotic beneficially affects the host by stimulating the growth or activity of specific species of large bowel bacteria, including *Bifidobacterium* and *Lactobacillus*, thought to improve host health. In 1990, Ohkuma et al. showed the results of changing pattern of microbial flora caused by administration of resistant maltodextrin. Other beneficial physiological effect of resistant maltodextrin as prebiotic has been mentioned by several researchers, such as the effect on attenuating postprandial blood glucose levels, improving glucose and lipid metabolism, and preventing obesity. Hypothetically, resistant maltodextrin as prebiotic has been assumed to have the effect on mucosal immune response of the intestine.

b. **Knowledge gap:** Dietary fibre, a non-digestible carbohydrate, has been used in decades for the beneficial effect of health with physiological importance. Although dietary fibre has a lot of benefit even in Europe and USA the consumption level is only 50-60% of the recommended level due to increased consumption of processed and pre-cooked food. Non-digestible maltodextrin as water soluble dietary fibre with relatively low molecular weight and widely acceptable to all kind of foods and beverages can really solve the problem. Researchers have stated that dietary fibre especially digestive-resistant maltodextrin has innumerable beneficial effects on human health, such as improving intestinal regularity by increasing faecal bulk, stimulating peristalsis and shortening gastrointestinal transit time. As a prebiotic, almost half of resistant maltodextrin reach the large intestine and is fermented by intestinalflora. In 1990, Ohkuma et al. showed the result of changing pattern of microbial flora caused by administration of resistant maltodextrin. Other beneficial physiological effect of resistant maltodextrin as prebiotic has been mentioned by several researchers, such as the effect on attenuating postprandial blood glucose levels, improving glucose and lipid metabolism, and preventing obesity. In developing countries most of the diarrheal episodes occur during the first two years of life and till date antibiotics have been found to have no role in over seventy five percent of young childhood diarrhoea. Toddler's diarrhoea which affects children aged 6 to 60 months is known as chronic nonspecific diarrhoea of childhood. The stool is frequently watery or loose and may have food particles in it. Despite the diarrhoea, the childcontinues to grow andgainweight, remains active and has a normal appetite. Infections due to Cryptosporidium, Giardia, andClostridium difficile can cause chronic diarrhoea in children. Other than infectious aetiologies these children may suffer from lactose intolerance, malabsorption, celiac disease, and less commonly

inflammatory bowel disease. Additionally, these episodes may have typical dietary and clinical history. Treatment for these episodes has been suggested to be normalization of feeding patterns according to the “four Fs”: Fat, Fibre, Fluid, and Fruit juice. Due to lack of evidence based studies regarding role of fibres in small apparently healthy children of Bangladesh, we anticipate to determine the safety, tolerability and acceptability of Fibersol-2 among children 1-3 years old.

Relevance: The beneficial effects of resistant maltodextrin are well known in developed countries; however, data are lacking in developing countries.Thus, it has become imperative to know its safety, tolerability and acceptability in small children with or without diarrhea in developing countries such as in Bangladesh. The present clinical trial will find out the safety, tolerability and acceptability of resistant maltodextrin in small children especially those 1-3 years old.

Hypothesis (if any): The resistant maltodextrin as a prebiotic is hypothesized to be safe, well tolerated and accepted in small children especially from 1-3 years old.

Objectives: The objectives of the present study are to assess the digestive tolerability of the Fibersol-2 in children which includes abdominal pain, rumbling, bloating, flatulence, and stool consistency. Additionally, the study will examine whether Fibersol-2 and ORS can reduce duration of watery diarrhea and stool output in children 1-3 years.

Methods: We are proposing to conduct an exploratory study to understand the digestive tolerability of resistant maltodextrin (Fibersol-2) in young healthy children aged 1-3 years. Additionally, we propose to conduct a placebo-controlled, randomized, double blind clinical trial to examine whether Fibersol-2 with ORS can reduce the duration of diarrhea and stool output in children of 1-3 years old.

Outcome measures/variables:

Main outcome measures will be; duration of diarrhoea, proportion of patients recovered within 72 hours, and daily stool output.

Description of the Research Project

Hypothesis to be tested:

In a hypothesis testing research proposal, briefly mention the hypothesis to be tested and provide the scientific basis of the hypothesis, critically examining the observations leading to the formulation of the hypothesis.

Does this research proposal involve testing of hypothesis: ☐ No ☒ Yes (describe below)

The resistant maltodextrin as prebiotic is assumed to be safe, well tolerated and accepted in small children especially from 1-3 years old.

Specific Objectives:

Describe the specific objectives of the proposed study. State the specific parameters, gender aspects, biological functions, rates, and processes that will be assessed by specific methods.

The objectives of the present study are to assess the digestive tolerability of the Fibersol-2 in children which includes abdominal pain, rumbling, bloating, flatulence, and stool consistency. Additionally, the study will examine whether Fibersol-2 and ORS can reduce duration of watery diarrhea and stool output in children 1-3 years.

Background of the Project including Preliminary Observations:

Provide scientific validity of the hypothesis based on background information of the proposed study and discuss previous works on the research topic, including information on sex, gender and diversity (ethnicity, SES) by citing specific references. Critically analyze available knowledge and discuss the questions and gaps in the knowledge that need to be filled to achieve the proposed aims. If there is no sufficient information on the subject, indicate the need to develop new knowledge.

Introduction:

Dietary fibre, a non-digestible carbohydrate, has been used in decades for the beneficial effect on health with physiological importance because such compounds have low energy values. These indigestible carbohydrates generally reach the large intestine undigested and unabsorbed, they are often used in many functional and/or low-calorie food and beverages[1-5]. Although dietary fibre has a lot of benefit even in Europe and USA the consumption level is only 50-60% of the recommended level due to increased consumption of processed and pre-cooked food[6, 7]. Non-digestible maltodextrin as water soluble dietary fibre with relatively low molecular weight and widely acceptable to all kind of foods and beverages can really solve the problem.Researchers have stated that dietary fibre especially digestive-resistant maltodextrin has innumerable beneficial effects on human health, such as improving intestinal regularity by increasing faecal bulk, stimulating peristalsis and shortening gastrointestinal transit time. Resistant maltodextrin (Fibersol-2) is a low viscosity, water-soluble, indigestible dextrin produced by the treatment of cornstarch with acid, enzymes, and heat.

Resistant maltodextrin also have prebiotic activity [8-10]. Prebiotic beneficially affects the host by stimulating the growth or activity of specific species of large bowel bacteria, including *Bifidobacterium* and *Lactobacillus*, thought to improve host health. Prebiotics are known to result in a decrease in pathogenic bacteria, such as Clostridium perfringens. Other than that prebiotics by decreasing pH cause increased production of short chain fatty acid(SCFA) and result in increased competition for nutrients. As a prebiotic, almost half of resistant maltodextrin reach the large intestine and is fermented by intestinal flora [8, 11-13]. In 1990, Ohkuma et al. showed the results of changing pattern of microbial flora caused by administration of resistant maltodextrin. Other beneficial physiological effect of resistant maltodextrin as prebiotic has been mentioned by several researchers, such as the effect on attenuating postprandial blood glucose levels, improving glucose and lipid metabolism, and preventing obesity. Hypothetically, resistant maltodextrin as prebiotic has been assumed to have the effect on mucosal immune response of the intestine.In recent years, low-

molecular-weight water-soluble dietary fibres like maltodextrin have been hypothesized to induce gastrointestinal symptoms, including osmotic diarrhea. One study reported that a 5-10 g single dose of inulin can cause abdominal flatulence in 42-50% of subjects, and diarrhoea in some cases.A number of previous studies conducted in Bangladeshand other parts of the world suggested that partially hydrolyzed guar gum (PHGG) substantially reduced the duration of diarrhea and modestly decreasedstool output in acute non-cholera diarrhea in young children, indicating its potential as a new anti-diarrheal therapy for acute diarrhea in children[4, 14-16]. The evidences showed that PHGG with added oral rehydration solution enhanced early recovery of acute diarrhoea in severely malnourished children in terms of reducing the duration of diarrhoea and stool output. Similar results were also reported when PHGG was used with ORS in the treatment of acute diarrhoea in non-severely malnourished children [14].
Although diet rich in fibre is widely recommended but efficacy of fibre supplements has not been tested sufficiently in children. One study with WHO-ORS containing partially hydrolyzed guar gum (PHGG) and another PHGG supplemented comminute chicken diet have reported to reduce diarrhea duration and stool output in children with acute and persistent diarrhea, respectively. PHGG is a soluble fibre and added PHGG in the ORS or diet escapes digestion in the small intestine and enter into the colon where the fibres are expected to be fermented by colonic bacteria producing SCFAs. SCFAs will stimulate sodium and water absorption in the colon leading to early recovery from diarrhea[17].

The viscosity of resistant maltodextrinis very clear and stable and does not become cloudy or show signs of any precipitation when kept for long periods of time. It also has very good anti-acid properties and can be cooked and sterilized at high temperature in food applications due to its stability in heat processes [9].

According to a systematic review, children admitted to hospital with dehydration associated with diarrhea, reduced osmolarity rehydration solution with diarrhea is associated with reduced need for unscheduled intravenous infusions, lower stool volume, and less vomiting compared with standard WHO rehydration solution. Children with acute diarrhea, therefore, may benefit from a reduced osmolarity ORS solution[18-21].

Dietary fibres can act by changing the nature of the contents of the gastrointestinal tract and by changing how other nutrients and chemicals are absorbed [22]. Advantages of consuming fibre are the production of healthful compounds during fermentation, increased bulk of stool, softening of stool, shortening of transit time through the intestinal tract, blocking of intestinal mucosal adherence, translocation of potentially pathogenic bacteria, and modulation of intestinal inflammation [23-27]. Based on literature review we are hypothesizing that resistant maltodextrin, a water-soluble, nonviscous and highly digestion-resistant maltodextrin is digestively tolerable and may not cause gastrointestinal symptoms including diarrhea in young healthy children. Additionally, it is hypothesized that resistant maltodextrin with ORS will be efficacious in the treatment of watery diarrhea in young children.

The objectives of the present study are to assess the digestive tolerability of the resistant maltodextrin in children which includes abdominal pain, rumbling, bloating, flatulence, and stool consistency. Additionally, the study will examine whether resistant maltodextrin and ORS can reduce duration of watery diarrhea and stool output in children 1-3 years.

It is a water-soluble dietary fiber (digestion resistant dextrin) which can easily be taken every day. It easily gets dissolved in water without changing its smell and taste at all. It can also be dissolved in any drink that one likes. Resistant maltodextrin contains 90% dietary fiber (dry solid base) which helps reduce absorption of sugar and fat so that the rise of blood sugar level and blood RLP-

cholesterol level after meal would be low. Adults are advised to have one packet (as dietary fiber 5g) per meal. Three packets per day are standard. It is advised to dissolve the packet in any drink and have that with meal.

In our study fibersol-2 (resistant maltodextrin) will be given to the study participants twice daily preferably in the morning and at evening with 50 ml of water for study children; but in case of diarrheal children in the proposed RCT we will use Fibersol-2also with water because it is water-soluble dietary fibre dissolve in 50 ml of water without changing its smell and taste.We will evaluate two presumptive dosesof Fibersol-2 inphase 1 to understand the better tolerability and acceptability of one of the doses of Fibersol-2 in healthy as well as diarrheal children and the suitable dose will be used for the clinical trial in diarrheal children.

An experienced researcher at icddr,b, not involved in the study, will prepare the randomization list using the randomization table. The name of random allocation will be indicated on a slip of paper, kept inside the sealed envelope. The sealed envelopes will be supplied to the researcher to supply the intervention package. The children with no dehydration or some dehydration will be randomized immediately after admission.

Research Design and Methods

Describe the research design and methods and procedures to be used in achieving thespecific aims of the research project. If applicable, mention the type of personal protective equipment (PPE), use of aerosol confinement, and the need for the use BSL2 or BSL3 laboratory for different part of the intended research in the methods.. Define the study population with inclusion and exclusion criteria, the sampling design, list the important outcome and exposure variables, describe the data collection methods/tools, and include any follow-up plans if applicable. Justify the scientific validity of the methodological approach (biomedical, social, gender, or environmental).

Also, discuss the limitations and difficulties of the proposed procedures and sufficiently justify the use of them.

We are proposing to conduct an exploratory study to understand the digestive tolerability of resistant maltodextrin (Fibersol-2) in young healthy and diarrheal childrenaged 1-3 yearsfor30 children at homeand 30 children at hospital in phase 1. Additionally, we propose to conduct a placebo-controlled, randomized, double blind clinical trial to examine whether Fibersol-2 with water can reduce the duration of diarrhea and stool output in children of 1-3 years old in the tertiary level hospital.If the child vomits out within 10 minutes of oral intake, we will give him again similar amount of fibersol-2 for consumption after an hour of rejection; if the child vomits again within 10 minutes of next intake, we will stop giving him fibersol-2anymore. We will also assessthe digestive tolerability of the study children.In addition to gathering information from hospital, our field research staff will visit the households of study children to collect information about their health status by administering field tested questionnaire.

Our study staff members will follow-up the study participants round the clock (24 hrs) during their hospital stay. To ensure the patient care they maintain a roster duty with staff members consisting 2 Project Research Physicians, 4 Study Nurse, 2 Field Research Assistants and 4 Field Organizer. Project physician will be responsible for consenting as well as all clinical assessment including the assessment of the diarrheal patients and provide treatment, besides these they will be given treatment for other illness if needed, our study nurses are responsible for recording vital signs and will feed the Fibersol-2 to the study participants in front of their legal guardians at hospital and community level with appropriate dose and time. They will also be closely monitoring the participants after oral intake of fibersol-2; if any adverse event is observed they will call the study physician immediately. Our Field Research assistant and Field Organizer will be screening the community control as well as will record other socio-demographic information and anthropometry as required. They will also assist the study nurse in monitoring the study participants at household level.

Flow chart of study design:

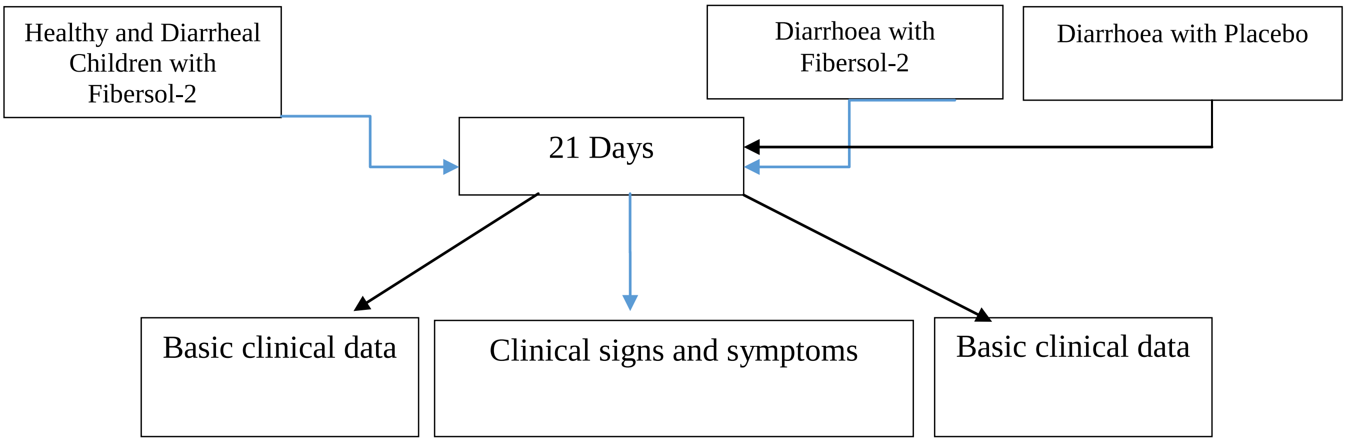

Study design of exploratory study

We will conduct the study in two phases – in phase I the safety and tolerability issues will be examined among healthy **and diarrheal** children (n=30**+30=60**), and if Fibersol-2 is found to be safe and tolerable, we will approach the DSMB to seek permission to extend the studyfor phase IIas the clinical trial only in diarrheal children. The evaluable clinical signs include abdominal pain, rumbling, bloating, flatulence, and stool consistency.

Eligibility for assessing digestive tolerability

Eligible age for the study: 1-3 years
Genders eligible for the study: Both
Include only healthy children: Yes

Eligibility criteria for the exploratory study

Inclusion criteria for healthy children:
(i)Healthy children, (ii) Aged between 1-3 years, and (iii) Received written consent from parents

Exclusion criteria:
(i)History of food allergy, (ii) Antibiotic or any medication that impacts the gut transit during the two weeks before the study, (iii) Chronic gastrointestinal disease, (iv) Gastroenteritis in the two weeks before the study, (v) Constipation (less than 3 stools per week) or diarrhea (three or more than 3 stools per day), (vi)child in a situation and could interfere with the optimal participation to the study or constitute a particular risk of non-compliance, (vii) currently participating in another clinical trial, and (viii) Parents refused to give written consent.

Inclusion criteria for diarrheal children:
Eligible age for the study: 1-3 years
Genders eligible for the study: Both
Include only diarrheal children: Yes
and (iii) Received written consent from parents
Exclusion criteria:

(i)History of food allergy, (ii) Antibiotic or any medication that impacts the gut transit during the two weeks before the study, (iii) Chronic gastrointestinal disease, (iv) Gastroenteritis in the two weeks before the study, (v) Constipation (less than 3 stools per week) (vi)child in a situation and could interfere with the optimal participation to the study or constitute a particular risk of non-compliance, (vii) currently participating in another clinical trial, and (viii) Parents refused to give written consent.

Methods for exploratory study

Study arm: 2 arms study which consists of 15 healthy children in each arm with Fibersol-2 dosing 2.5 g twice daily and 5g twice daily respectively

1. Study group 1, 15healthy children will receive Fibersol-2, 2.5g twice dailywith 50 ml drinking water
2. Study group 2, 15healthy children will receive Fibersol-2, 5g with 50 ml drinking watertwice daily

Study arm: 2 arm study which consists of 15 diarrheal children in each arm with Fibersol-2 dosing 2.5 g twice daily and 5 g twice daily respectively

1. Study group 1, 15 diarrheal children will receive Fibersol-2, 2.5g twice daily with 50 ml drinking water
2. Study group 2, 15 diarrheal will receive Fibersol-2, 5g with 50 ml drinking water twice daily

Study duration:

1. Pre-study observation: 7 days[During the pre-study observation period our study staff members will collect clinical information as well as associated co-morbidity from control group children to see whether they are eligible for enrolment].
2. Main study period : 7 days
3. Post-study observation: 7 days [Our research staff will visit households as well as hospital to collect 1-day recall data regarding health status; caregivers will be informed that the research staff will visit for consecutive 7 days]
   Total observation period 21 days

Study design for clinical efficacy trial

The proposed clinical efficacy trial will be a placebo-controlled, randomized; double-blind 2 arm trial to assess the efficacy of low osmolarity ORS plus Fibersol-2 compared with low osmolarity ORS in the treatment of acute watery diarrhea in children.

Eligibility for assessing for clinical efficacy trial

Eligible age for the study: 1-3 years
Genders eligible for the study: Both
Include only acute diarrheal children: Yes

Eligibility criteria for the clinical efficacy trial

Inclusion criteria:

(i)Children with acute watery diarrhea, 3 or more watery stool in any 24-hour period of <7 days duration with none or some dehydration (ii) Aged between 1-3 years, and (iii) Received written consent from parents

Exclusion criteria:

(i)Children with bloody diarrhea, severe diseases (severe sepsis, meningitis, severe pneumonia with respiratory distress requiring intensive care and ancillary support such as oxygen inhalation, orophryngeal suction etc., (ii) Child in a situation and could interfere with the optimal participation to the study or constitute a particular risk of non-compliance, (iii) Currently participating in another clinical trial, and (iv) Parents refused to give written consent.

Methods for clinical efficacy trial

A placebo-controlled, randomized; double-blind 2 arm trial to assess the efficacy of fibersol-2 in children with acute watery diarrhea. Children with acute watery diarrhea, 3 or more watery stool in any 24-hour period of <7 days duration with no dehydration.

Study group 1, 46 will receive Fibersol-2 disolved in 50 ml drinking water, twice daily (suitable dose from the tolerability and acceptability trial)

Study group 2, 46 will receive Placebo (regular maltodextrin) disolved in 50 ml drinking water twice daily (same dose as Fibersol-2)

An experienced researcher at icddr, b, not involved in the study, will prepare the randomization list using the randomization table. The name of random allocation will be indicated on a slip of paper, kept inside the sealed envelope. The sealed envelopes will be supplied to the researcher to supply the intervention package. The children with no dehydration or some dehydration will be randomized immediately after admission.

Baseline information

Children fulfilling the eligibility criteria will stay in the hospital throughout the study period until discharged. Research physician will take a detailed medical history of the enrolled children to determine the duration of and type of diarrhoea and its frequency; duration and frequency of vomiting; and presence of other symptoms such as fever, feeding difficulties, and treatment received for the illness before admission; and perform a thorough physical examination including assessment of dehydration according to the guidelines used in icddr,b and also the nutritional status will be measured and recorded.

Case management

Dehydration will be assessed according to the modified WHO guidelines followed in the hospital. In children with some dehydration, the fluid deficit will be corrected with ORS in an amount 10 ml/kg/hour for the first hours, then 5 ml/kg/hr until the deficit is corrected. In addition, ongoing stool losses will be replaced with ORS 5-10 ml/kg after each watery stool. For high purging children, the ORS intake will be adjusted according to the ongoing stool loss. ORS therapy will continue until diarrhoea stops. Mothers will be advised to continue breastfeeding.

Measurements

Fluids intakes (IV, ORS, and water)

ORS will be given to the after measuring with a calibrated cylinder and the amount intake will be recorded every 6 hours, in case of any leftover that will be deducted from previous order. Water intake will also be measured in a similar way. When needed IV fluid will be infused through a calibrated soluset, the amount infused will be recorded every 6 hours if the child requires IV fluid therapy.

Output (stool, urine and vomitus)

Stool will be collected in a bucket of known weight beneath the cholera cot with a central hole and will be measured every 6 hours with an electronic scale of a precision of 1 gram. Urine will be collected by pediatric urine collector (PUC bag) and measured with a calibrated cylinder in ml. Vomitus will be collected in a pre-weighed bowl and measured with an electronic scale. The children will be offered a defined food of known calorie after measuring with an electronic scale of precision 1 g. Any leftover will be measured and subtracted from the amount offered and amount ingested will be recorded every 6 hours. Nude body weight will be measured at admission, after rehydration, and every 6 hours until recovery from diarrhoea and then at the end of every 24 hours and at discharge. Clinical evaluation will be performed every morning and evening. Resolution of diarrhea will be defined as the passage of two consecutive soft/formed stools or no stool for 24 hours. Therapeutic success will be defined as the cessation of diarrhea within 7 days of inclusion in the study treatment. Duration of diarrhea will be calculated in hours from the time of randomization to the last watery or loose stool within 7 days. Children will be considered withdrawn from the study if their parents or legal guardian withdraw consent, or the child is withdrawn from study for treatment of any complications; data (intakes and outputs) of such children up to the time of withdrawal will be included in the analysis (intent to treat analysis). Data of the children who failed to recover within seven days (study period) will also be included in the analysis for a maximum of seven days; those will be labelled as therapeutic failures and will be treated in the hospital until recovery.

Primary outcome measures will be; duration of diarrhea, proportion of patients recovered within 72 hours, and daily stool output.

Definitions

Abdominal distension occurs when substances, such as air (gas) or fluid, accumulate in the abdomen causing its outward expansion beyond the normal girth of the stomach and waist. It will be evaluated by regular measurement of abdominal girth.No set parameters for abdominal distension. To measure the abdominal girth before the IP and repeat measurement daily. If there is change that is increase in diameter of girth measurement,then we will define distension. Recurrent abdominal pain or discomfort at least 3days/month in the last 3 months. [28, 29]

Abdominal pain: Abdominal pain is felt in the part of the trunk below the ribs and above the pelvis. Abdominal pain comes from organs within the abdomen or organs adjacent to the abdomen. It will be measured by asking mother`s perception whether the child had the history of cry, irritability which was associated with abdominal pain. [28]

Rumbling: A stomach rumble, also known as a bowel sound or peristaltic sound, is a rumbling, growling or gurgling noise produced by movement of the contents of the gastro-intestinal tract as they are propelled through the small intestine by a series of muscle contractions called peristalsis. It will be measured both by taking history from the parents and by abdominal auscultation.

Bloating: Bloating is the presence of abnormal general swelling, or increased in diameter of the abdominal area. Recurrent feeling of bloating or visible distension at least 3 days/month in the last 3 months [28]. It will also be evaluated by regular measurement of abdominal girth.
Flatulence: "flatus expelled through the anus" or the "quality or state of being flatulent", which is defined in turn as "marked by or affected with gases generated in the intestine or stomach; likely to cause digestive flatulence". It will be measured by taking history from the parents.
Stool consistency: Appearance of the stool; such as formed, mucoid, or liquid.This will be measured by direct visualization by the parents as well treating physicians.

Sample Size Calculation and Outcome (Primary and Secondary) Variable(s)

Clearly mention your assumptions. List the power and precision desired. Describe the optimal conditions to attain the sample size. Justify the sample size that is deemed sufficient to achieve the specific aims.

Based on the results of a clinical trial of ORS solution in children with acute watery diarrhea with an anticipation of a 25% reduction in 48-hour stool output after in children after receiving Fibersol-2 disolved with drinking water. Considering 5% level of significance and 90% power and 10% drop out the sample size is estimated to be 46 in each group [14]. However, for the tolerability and acceptability study we have selected 30+30=60 children suggested by the respected members of the RRC and ERC respectively.

For the tolerability and acceptability studyin healthy Study group 1, 15 healthy children will receive Fibersol-2, 2.5 mg twice daily
Study group 2, 15 healthy children will receive Fibersol-2, 5mg twice daily

For the tolerability and acceptability studyin children with diarrhea Study group 1, 15 diarrheal children will receive Fibersol-2, 2.5g twice daily
Study group 2, 15 diarrheal children will receive Fibersol-2, 5g twice daily

For RCT in diarrheal children:
Study group 1, 46 will receive Fibersol-2 disolved in 50 ml drinking water, twice daily (suitable dose from the tolerability and acceptability trial)

Study group 2, 46 will placebo (regular maltodextrin) disolved in 50 ml drinking water, twice daily (same dose as in Fibersol-2)

Data Analysis

Describe plans for data analysis, including stratification by sex, gender and diversity. Indicate whether data will be analysed by the investigators themselves or by other professionals. Specify what statistical software packages will be used and if the study is blinded, when the code will be opened. For clinical trials, indicate if interim data analysis will be required to determine further course of the study.

Statistical methods

All data will be entered into microcomputer and analyzed using the software ‘Statistical Package for Social Science’; version20.0, Chicago, IL. Continuous variables will be compared between groups with student’s t-test and nonparametric test. Categorical variables will be compared by chi-squaretest. Kaplan-Meir survival curves will be constructed for the duration of diarrhoea and for comparison with log rank test; a p value <0.05 will be considered as statistically significant. To see

whether there are significant differences among the groups, we will perform ANOVA to systematically examine variability within groups being compared.

Data Safety Monitoring Plan (DSMP)

All clinical investigations (research protocols testing biomedical and/or behavioural intervention(s)) should include the Data and Safety Monitoring Plan (DSMP). The purpose of DSMP is to provide a framework for appropriate oversight and monitoring of the conduct of clinical trials to ensure the safety of participants and the validity and integrity of the data. It involves involvement of all investigators in periodic assessments of data quality and timeliness, participant recruitment, accrual and retention, participant risk versus benefit, performance of trial sites, and other factors that can affect study outcome.

The research protocol testing the clinical efficacy will include the Data and Safety Monitoring Plan (DSMP) by forming a monitoring board by an independent Data Safety and Monitoring Board (DSMB) that will be formed by the ERC of icddr, b comprising of its members, such as scientists, paediatricians, epidemiologists, lawyers, and others. The trial will be registered with the Clinical Trial Registry. The purpose of DSMP is to provide a framework for appropriate oversight and monitoring of the conduct of clinical trials to ensure the safety of participants and the validity and integrity of the data. It will involve the participation of all investigators in periodic assessments of data quality and timeliness, study participant recruitment, accrual and retention, participant risk versus benefit, performance of trial site, and other factors that can affect study outcome. A DSMB will be constituted to oversee the implementation of the study activities.

Ethical Assurance for Protection of Human rights

Describe the justifications for conducting this research in human participants. If the study needs observations on sick individuals, provide sufficient reasons for using them. Indicate how participants' rights will be protected, and if there would be benefit or risk to each participants of the study. Discuss the ethical issues related to biomedical and social research for employing special procedures, such as invasive procedures in sick children, use of isotopes or any other hazardous materials, or social questionnaires relating to individual privacy. Discuss procedures safeguarding participants from injuries resulting from study procedures and/or interventions, whether physical, financial or social in nature. [Please see Guidelines]

Ethical consideration

Written informed consent will be obtained from parents/legal guardian of each of the participating children. The study subjects will be recruited once the protocol will be approved by the Research Review Committee (RRC) and Ethical Review Committee (ERC) of icddr,b with clinical trial registration.

Use of Animals

Describe if and the type and species of animals to be used in the study. Justify with reasons the use of particular animal species in the research and the compliance of the animal ethical guidelines for conducting the proposed procedures.

Not Applicable

Collaborative Arrangements

Describe if this study involves any scientific, administrative, fiscal, or programmatic arrangements with other national or international organizations or individuals. Indicate the nature and extent of collaboration and include a letter of agreement between the applicant or his/her organization and the collaborating organization.

This study will be implemented in collaboration with the Matsutani Chemical Industry Co., Ltd.The participating institutions and investigators are known to each other. icddr,b will be the administering institution and has substantial experience in the implementation of collaborative reserach projects. icddr,b will lead this collaborative research. All of the required expertise for the clinical trial in health facility is present with a highly trained with long experience in clinical trial programs.

Facilities Available

Describe the availability of physical facilities at site of conduction of the study. If applicable, describe the use of Biosafety Level 2 and/or 3 laboratory facilities. For clinical and laboratory-based studies, indicate the provision of hospital and other types of adequate patient care and laboratory support services. Identify the laboratory facilities and major equipment that will be required for the study. For field studies, describe the field area including its size, population, and means of communications plus field management plans specifying gender considerations for community and for research team members.

We propose to conduct this study in a community of rural Bangladesh (Mirzapur) located nearly 60 Km northwest of Dhaka, the capital city. The economy of the community is agro-based. The community has good metallic roads and mothers can reach the facility, often without male escorts, easily using the locally available transports. The tertiary level sentinel health facility is one of the oldest and largest tertiary level facilities in rural Bangladesh. It has 750 beds. We proposed to conduct this study in rural Bangladesh due to rural people represents more than 80% of our total population. Beside this rural people is less possibility to move away than urban people for their caregiver's day to day activity. Our community population are stable and mothers/caretakers in most instances are available at households for follow-up visits because they less often get involved in any paid job. Besides this, rural families less frequently move away from present place of residence than urban population which might interfere with any longitudinal follow-up of visits of children at household level.

Enrolment of the study children and data collection will take place in the sentinel health facility - a tertiary level facility popularly known as Kumudini Hospital located in the middle of the study community. Interviews will be conducted in the same facility. A quiet, private room will be used for the one-on-one interviews. All materials will be kept in a secure location on site.

Literature Cited

Identify all cited references to published literature in the text by number in parentheses. List all cited references sequentially as they appear in the text. For unpublished references, provide complete information in the text and do not include them in the list of Literature Cited. There is no page limit for this section, however, exercise judgment in assessing the "standard" length.

1. Hillemeier, C., *An overview of the effects of dietary fiber on gastrointestinal transit.* Pediatrics, 1995. **96**(5 Pt 2): p. 997-9.
2. Kishimoto, Y., et al., *The maximum single dose of resistant maltodextrin that does not cause diarrhea in humans.* J Nutr Sci Vitaminol (Tokyo), 2013. **59**(4): p. 352-7.
3. Livesey, G., *Tolerance of low-digestible carbohydrates: a general view.* Br J Nutr, 2001. **85 Suppl 1**: p. S7-16.
4. Bonnema, A.L., et al., *Gastrointestinal tolerance of chicory inulin products.* J Am Diet Assoc, 2010. **110**(6): p. 865-8.
5. Castillejo, G., et al., *A controlled, randomized, double-blind trial to evaluate the effect of a supplement of cocoa husk that is rich in dietary fiber on colonic transit in constipated pediatric patients.* Pediatrics, 2006. **118**(3): p. e641-8.
6. King, D.E., A.G. Mainous, 3rd, and C.A. Lambourne, *Trends in dietary fiber intake in the United States, 1999-2008.* J Acad Nutr Diet, 2012. **112**(5): p. 642-8.
7. McGill, C.R., V.L. Fulgoni, 3rd, and L. Devareddy, *Ten-year trends in fiber and whole grain intakes and food sources for the United States population: National Health and Nutrition Examination Survey 2001-2010.* Nutrients, 2015. **7**(2): p. 1119-30.
8. Fastinger, N.D., et al., *A novel resistant maltodextrin alters gastrointestinal tolerance factors, fecal characteristics, and fecal microbiota in healthy adult humans.* J Am Coll Nutr, 2008. **27**(2): p. 356-66.
9. Ye, Z., et al., *Soluble dietary fiber (Fibersol-2) decreased hunger and increased satiety hormones in humans when ingested with a meal.* Nutr Res, 2015. **35**(5): p. 393-400.
10. Guimaraes, E.V., E.M. Goulart, and F.J. Penna, *Dietary fiber intake, stool frequency and colonic transit time in chronic functional constipation in children.* Braz J Med Biol Res, 2001. **34**(9): p. 1147-53.
11. Gibson, G.R. and M.B. Roberfroid, *Dietary modulation of the human colonic microbiota: introducing the concept of prebiotics.* J Nutr, 1995. **125**(6): p. 1401-12.
12. Buddington, R.K., *The use of nondigestible oligosaccharides to manage the gastrointestinal ecosystem. Microbial Ecol Health Dis (http://www.microbecolhealthdis.net/index.php/mehd/article/view/7992).* 2001.

13. Fuller, R. and G.R. Gibson, *Modification of the Intestinal Microflora Using Probiotics and Prebiotics*. Scand J Gastroenterol, 1997. **32 Suppl 222**: p. 28-31.
14. Alam, N.H., et al., *Efficacy of partially hydrolyzed guar gum (PHGG) supplemented modified oral rehydration solution in the treatment of severely malnourished children with watery diarrhoea: a randomised double-blind controlled trial*. J Health Popul Nutr, 2015. 34: p. 3.
15. Nomura, M.N., Y. Abe, H. ,*Effects of long-term administration of indigestible dextrin as soluble dietary fiber on lipid and glucose metabolism [1992] (http://agris.fao.org/agris-search/search.do?recordID=JP9400816)*. 1992.
16. Kazuhiro Okuma, I.M., *Indigestible Fractions of Starch Hydrolysates and Their Determination Method (https://www.jstage.jst.go.jp/article/jag1999/49/4/49_4_479/_article)*. 2002.
17. Kazuhiro OHKUMA, I.M., Yasuo KATTA, Yoshio HANNO, *Pyrolysis of Starch and Its Digestibility by Enzymes -Characterization of Indigestible Dextrin-. (https://www.jstage.jst.go.jp/article/jag1972/37/2/37_2_107/_article/cited-by)*. 1990.
18. Hahn, S., S. Kim, and P. Garner, *Reduced osmolarity oral rehydration solution for treating dehydration caused by acute diarrhoea in children*. Cochrane Database Syst Rev, 2002(1): p. Cd002847.
19. Pulungsih, S.P., et al., *Standard WHO-ORS versus reduced-osmolarity ORS in the management of cholera patients*. J Health Popul Nutr, 2006. **24**(1): p. 107-12.
20. Alam, N.H., et al., *Symptomatic hyponatremia during treatment of dehydrating diarrheal disease with reduced osmolarity oral rehydration solution*. Jama, 2006. 296(5): p. 567-73.
21. *Multicenter, randomized, double-blind clinical trial to evaluate the efficacy and safety of a reduced osmolarity oral rehydration salts solution in children with acute watery diarrhea*. Pediatrics, 2001. **107**(4): p. 613-8.

22. "Dietary Reference Intakes for Energy, Carbohydrate, fibre, Fat, Fatty Acids, Cholesterol, Protein, and Amino Acids (Macronutrients) (2005), Chapter 7: Dietary, Functional and Total fibre" (PDF). US Department of Agriculture, National Agricultural Library and National Academy of Sciences, Institute of Medicine, Food and Nutrition Board.Eastwood M, Kritchevsky D (2005). "Dietary fiber: how did we get where we are?". Annu Rev Nutr. **25**: 1–8. *doi:10.1146/annurev.nutr.25.121304.131658. PMID 16011456.*

Anderson JW, Baird P, Davis RH, et al. (2009). "Health benefits of dietary fiber". Nutr Rev. **67** (4): 188–205. *doi:10.1111/j.1753-4887.2009.00189.x. PMID 19335713.*

Nugent, Anne P (2005). *"Health properties of resistant starch"*. Nutrition Bulletin. **30** (1): 27–54. *doi:10.1111/j.1467-3010.2005.00481.x.*

Simpson, H; Campbell, BJ (2015). *"Review article: dietary fibre-microbiota interactions."*. Aliment Pharmacol Ther. **42** (2): 158–79. *doi:10.1111/apt.13248.*

27. Simpson, H; Campbell, BJ; Rhodes, JM (2014). . Dig Dis. 32 Suppl1: 13–25. *doi:10.1159/000367821.*

28. Appendix A: Rome III Daignostic criteria for FGIDS *www.romecriteria.org/assets/pdf/19_RomeIII_apA_885-898***.**

29. Brian E. Lacy, Fermín Mearin, Lin Chang, William D. Chey, Anthony J. Lembo, Magnus Simren**,** Robin Spiller**.** Bowel Disorders**.** DOI: http://dx.doi.org/10.1053/j.gastro.2016.02.031
