# Supplementary material for: Digestive tolerability and acceptability of Fibersol-2 in healthy and diarrheal children 1–3 years old at a rural facility, Bangladesh: Results from a four arm exploratory study
Source: PLoS One. 2022 Sep 19;17(9):e0274302. doi: 10.1371/journal.pone.0274302 (PMC9484693; doi:10.1371/journal.pone.0274302)
Supplement: S1 Protocol — (PDF) [file pone.0274302.s005.pdf]

Study ID No.: | | | |

K	W	M	C	H

CASE REPORT FORM

(Healthy Children)

Protocol Number: PR-16091

Tolerability and Acceptability of Fibersol-2 (Resistant Maltodextrin) in healthy and diarrheal children followed by a randomized clinical trial to evaluate the efficacy of Fibersol-2 in diarrheal children 1-3 years old

Principal Investigator

Dr. Mohammod Jobayer Chisti, MBBS, MMed, PhD, icddr,b

Sponsor

Matsutani Chemical Industry Company.

INRORMED CONSENT

Date of Informed Consent signed: ☐☐ DD ☐☐☐ MON ☐☐ YY

Time of Informed Consent signed: ☐☐ HH ☐☐ MM

To be signed upon completion of all of the CRF for this patient:

I certify that the information supplied herein is a complete and accurate record of the patient's data, that the study was carried out according to the protocol and that the patient's written informed consent was obtained from his representative, parent or guardian.

Principal Investigator's Signature: **Date:**

Principal Investigator's Name (PLEASE PRINT): **Dr. Mohammod Jobayer Chisti**

Study ID No.: | | | |

K	W	M	C	H

CASE REPORT FORM (CRF) GUIDELINES

1. Please use a black ball pen to complete the CRF. **Press firmly.**
2. Use capital letters to fill the CRF.
3. Mark all choice fields (e.g.Yes/No boxes) with a "X"
4. Use 24 hour clock whenever it is asked to specify time, e.g. 9:00 pm to be captured as 2100
5. Use leading zeros when appropriate e.g.If patients weight is 10.5 Kg then enter it as 010.5 Kg
6. For unknown values place "UK" in the box. eg. Date: U K | D E C | 1 4

   Time: 1 9 U K

7. **If an error is made**
   - Do not obliterate or overwrite entry
   - Do not use correction fluid
   - Cross out with a single diagonal line ~~56~~ 66SAS
   - Re-enter clearly as near as practical to original entry 10/04/13
   - Initial and date beside the data correction
8. Do not give added information which is not requested.
9. Please ensure that reasons for any concomitant therapy or treatments clearly match medical history terms and comment if there is no change from baseline or that the matching event is reported as an adverse event.
10. For out of range height, weight, blood pressure or laboratory values please indicate if value is Clinically Significant (CS) and should be reported as an adverse event or if the value is Not Clinically Significant (NCS).

Study ID No.: | | | |

K	W	M	C	H

PRE-TREATMENT: ADMISSION

[PLEASE CAPTURE VALUE/NAME, OR CHECK APPROPRIATE RESPONSE IN COMPLETING FORM]

Date of Visit: DD MON YY   Time of Visit: HH : MM

GENERAL EXAMINATION DURING INITIAL REPORTING :

Vital signs

**Variables**	**Age** [months]	**Height** [cm]	**Weight(kg)** [morning only]	**Pulse Rate**(/Min)	**Resp. Rate** (/Min)	**Axillary Temp**(^{0}C)	**B.P.Systolic** (mmHg) [morning only]	**B.P Diastolic** (mmHg) [morning only]
Is any reading exceeds the normal range?			☐ No ☐ Yes	☐ No ☐ Yes	☐ No ☐ Yes	☐ No ☐ Yes	☐ No ☐ Yes	☐ No ☐ Yes
IF YES, is the deviation Clinically Significant?*			☐ No ☐ Yes	☐ No ☐ Yes	☐ No ☐ Yes	☐ No ☐ Yes	☐ No ☐ Yes	☐ No ☐ Yes

**[If Clinically Significant, please capture relevant details in Medical History or Adverse Event page as applicable]*

Study ID No.: | | | |

K	W	M	C	H

STATUS OF DEHYDRATION

Dehydration Status of child as per score provided below<br>(0 =signs of no dehydration 1 =signs of Some dehydration 2 =signs of Severe *)<br>**If Dehydration is severe please exclude the subject the study*	☐

Formula milk provided and ingested? ☐ No ☐ Yes<br>If Yes,Volume ☐☐☐☐ ml<br>Is the child on Breast fed? ☐ No ☐ Yes<br>Frequency, if yes ☐☐ per day<br>Is child Currently on Solid Food? ☐ No ☐ Yes	

Stool Consistency *(Check One)*	1 =Solid 2 =Paste 3 =Loose/watery 4 =Loose with blood<br>☐

[Guide: 1. Solid=Formed having its own shape; 2. Paste = soft; 3. Loose =Watery and can be poured from one container to another; 4. Loose with blood present = Blood and mucus with or without faecal matter present]

Study ID No.: | | | |

K	W	M	C	H

Other Symptoms like:

Details	Mother's Opinion		Interviewer's Opinion	
	NO	YES	NO	YES
Is Abdominal Pain present?	☐	☐	☐	☐
Is Abdominal Distension present?	☐	☐	☐	☐
Is Fever present?	☐	☐	☐	☐
Is Lethargy present?	☐	☐	☐	☐
Is Vomiting present?<br>If yes, number of vomiting per day ☐☐	☐	☐	☐	☐
Are other Abnormalities present?<br>If yes,<br>specify________________________________	☐	☐	☐	☐
History of mucus in stools	☐	☐	☐	☐
History of blood in stools	☐	☐	☐	☐
History of rice watery stools	☐	☐	☐	☐

STATUS OF OVERALL FOOD / MILK INTAKE

Mention food / Milk intake status of child as per scores provided below:<br>(1 =Normal, 2 =Mildly decreased, 3 =Moderately decreased, 4 =Severely decreased , 5 = Refused to eat	☐

Study ID No.: | | | |

K	W	M	C	H

SIGNIFICANT MEDICAL HISTORY (include jaundice, LRTI etc):

If none, check here ☐

Body System	NO	YES	If " YES", specify finding or diagnosis (if Known)
Ears, Nose, Throat:	☐	☐	
Opthalmological:	☐	☐	
Neurological:	☐	☐	
Cardiovascular:	☐	☐	
Respiratory:	☐	☐	
Gastrointestinal:	☐	☐	
Urogenital:	☐	☐	
Musculoskeletal:	☐	☐	
Dermatological:	☐	☐	
Endocrine:	☐	☐	
Hematological:	☐	☐	
Hepatic:	☐	☐	
Metabolic:	☐	☐	
Allergies and Drug Sensitivity:	☐	☐	

Study ID No.: ☐☐☐

K	W	M	C	H

ENROLMENT CRITERIA CHECKLIST

INCLUSION CRITERIA

If any criterion is checked "NO," Subject cannot be enrolled into the study.	NO	YES
1. Male and Female child aged 12 to 36 months	☐	☐
2. Guardian is willing and able to report to the investigator and provide information on stool frequency, stool consistency, vomiting frequency, food intake during the follow up phase	☐	☐
3. Written informed consent must be obtained prior to enrollment into this study	☐	☐
4. The subject had a physical examination that revealed no clinically significant abnormalities (altered mentation, convulsion, clinical suspicion, sepsis, meningitis, pneumonia,chronic gastrointestinal disease,acute gastroenteritis or other systemic infection other than those expected for subjects with acute infectious diarrhea)	☐	☐

EXCLUSION CRITERIA

If any criterion is checked "YES," Subject cannot be enrolled into the study	NO	YES
1. Suspected dysentery or constipation(less than 3 stools per week)	☐	☐
2. Severe dehydration during triage or screening	☐	☐
3. Vomiting severity that is likely to make administration and retention of test product impossible	☐	☐
4. Severe malnutrition (defined as weight-for-height of less than -3 SD below median, as per WHO Standards)	☐	☐
5. Child received antibiotics, or anti-motility drugs two weeks prior to screening	☐	☐
6. History of hypersensitivity or allergy to milk or egg	☐	☐
7. History of vaccination against rotavirus	☐	☐
8.Currently participating in another clinical trial or child in a situation and could interfere with the optimal participation to the study or constitute a particular risk of non-compliance	☐	☐

ELIGIBILITY

Is patient eligible for entry into study? ☐ No ☐ Yes

If Yes:

Date of Randomization: ☐☐ (DD) ☐☐☐ (MON) ☐☐ (YY) Time of Randomizatio: ☐☐ (HH) ☐☐ (MM)

Randomization Number: ☐☐☐

CRF 8

Study ID No.: | | | |

K	W	M	C	H

STUDY DAY 1

**Date of Visit** (DD MON YY)	**Time of Visit** (HH :MM)
	:

STUDY PRODUCT ADMINISTRATION SCHEDULE

Dose	**Date** (DD MON YY)	**Time** (HH :MM)	Volume Administered (ml)	Volume Remaining (ml)
**1**		:		

STUDY PRODUCT ADMINISTRATION SCHEDULE

Dose	**Date** (DD MON YY)	**Time** (HH :MM)	Volume Administered (ml)	Volume Remaining (ml)
**2**		:		

VITAL SIGNS

**Variables Time measured**	**Pulse Rate**(/Min)	**Respiratory Rate** (/Min)	**Axillary Temp** (°C)	**B.P. Systolic** (mmHg) [morning only]	**B.P. Diastolic** (mmHg) [morning only]
Is any reading outside of normal range?	☐ No ☐ Yes	☐ No ☐ Yes	☐ No ☐ Yes	☐ No ☐ Yes	☐ No ☐ Yes
IF YES, is the deviation Clinically Significant?*	☐ No ☐ Yes	☐ No ☐ Yes	☐ No ☐ Yes	☐ No ☐ Yes	☐ No ☐ Yes

**If Clinically Significant please fill up the Adverse Event Form as applicable.*

CRF 9

Other Symptoms like as:

Study ID No.: | | | |

K	W	M	C	H

Details	NO	YES
Is Abdominal Pain present?	☐	☐
Is Abdominal Distension present?	☐	☐
Is Fever present?	☐	☐
Is Lethargy present?	☐	☐
Is Vomiting present?<br>If Yes, number of vomiting per day ☐☐	☐	☐
Are other Abnormalities present?<br>If Yes, specify____	☐	☐
History of mucus in stools	☐	☐
History of blood in stools	☐	☐
History of rice watery stools	☐	☐

If the baby is breastfed: NO ☐ YES ☐ If YES, How many times (per day) ☐☐

If the baby is having formula milk: NO ☐ YES ☐ If YES, Amount per day ☐☐☐☐

STATUS OF OVERALL FOOD/ MILK INTAKE *(1 Hour before day ends)*

Mention food/Milk intake status of child as per scores provided below :<br>(1=Normal, 2=Mildly decreased, 3=Moderately decreased, 4=Severely decreased, 5=Refused to eat)	☐

STATUS OF DEHYDRATION *(1 Hour before day ends)*

Dehydration Status of child as per score provided below :<br>(0 =no signs of dehydration 1 =signs of Some dehydration 2 =signs of Severe dehydration*)<br>**If Dehydration is severe please exclude the subject from the study*	☐

ADVERSE EVENTS AND CONCOMITANT MEDICATIONS

Were there any changes in Adverse Events or Concomitant Medications since the last visit?<br>*If yes, please add new events/medications on appropriate log*	☐	**CRF 10**

Reaction of Child after intake of Study Product (Fibersol-2/Placebo):
For 1st dose of the day 01:

Study ID No.: | | | |

K	W	M	C	H

Amount of intake of Fibersol-2/Placebo in 30 mins: ☐.☐ **gm**

Description	No	Yes
Is Abdominal Distension present?	☐	☐
Is Abdominal Pain present?	☐	☐
Is Abdominal Rumbling present?	☐	☐
Is Abdominal Bloating present?	☐	☐
Child moved head away from the food?	☐	☐
Mouth clamped shut?	☐	☐
Teeth clenched?	☐	☐
Became agitated?	☐	☐
Spit out of the given drink?	☐	☐
Refused to swallow the given drink?	☐	☐

For 2nd dose of the day 01
Amount of intake of Fibersol-2/Placebo in 30 mins: ☐.☐ **gm**

Description	No	Yes
Is Abdominal Distension present?	☐	☐
Is Abdominal Pain present?	☐	☐
Is Abdominal Rumbling present?	☐	☐
Is Abdominal Bloating present?	☐	☐
Child moved head away from the food?	☐	☐
Mouth clamped shut?	☐	☐
Teeth clenched?	☐	☐
Became agitated?	☐	☐
Spit out of the given drink?	☐	☐
	☐	☐

Study ID No.: | | | |

CRF 11

K	W	M	C	H

Refused to swallow the given drink?		

Is vomiting present?<br>If yes, frequency per day? ☐☐<br><br>Amount of vomiting per day ☐☐☐ gm	**No** ☐ **Yes** ☐
In mother's opinion, the child likes Fibersol-2/placebo on not?	☐

*** Children were considered as refusing intake if they moved their head away from the food, cried, clamped the mouth shut or clenched the teeth, or became agitated, spit out the food or refused to swallow. The amount of food ingested was calculated by subtracting the left-over from the offered amount. Pre-weighed napkins were provided; any food that was regurgitated, vomited or spilled was swabbed, the napkin weighed and subtracted from the weight of the amount offered. Using a 7-point Hedonic Scale in which each point (1 = disliked extremely, 2 = disliked moderately, 3 = disliked, 4 = neither disliked nor liked, 5 = liked slightly, 6 = liked moderately, 7 = liked extremely) was depicted by a facial drawing, we asked mothers to rate the food's color, flavor, mouth feel, and overall acceptability.

Stool Consistency:	☐

* Stool Consistency: 1 =Solid 2 =Paste 3 =Loose/watery 4 =Loose with blood

Study ID No.: | | | |

CRF 12

K	W	M	C	H

STUDY DAY 2

Date of Visit (DD MON YY)	Time of Visit (HH :MM)
	:

STUDY PRODUCT ADMINISTRATION SCHEDULE

Dose	Date (DD MON YY)	Time (HH :MM)	Volume Administered (ml)	Volume Remaining (ml)
**1**		:		

STUDY PRODUCT ADMINISTRATION SCHEDULE

Dose	Date (DD MON YY)	Time (HH :MM)	Volume Administered (ml)	Volume Remaining (ml)
**2**		:		

VITAL SIGNS

**Variables Time measured**	**Pulse Rate**(/Min)	**Respiratory Rate** (/Min)	**Axillary Temp** (°C)	**B.P. Systolic** (mmHg) [morning only]	**B.P. Diastolic** (mmHg) [morning only]
Is any reading outside of normal range?	No<br>Yes	No<br>Yes	No<br>Yes	No<br>Yes	No<br>Yes
IF YES, is the deviation Clinically Significant?*	No<br>Yes	No<br>Yes	No<br>Yes	No<br>Yes	No<br>Yes

**If Clinically Significant please fill up the Adverse Event Form as applicable.*

CRF 13

Other Symptoms like:

	NO	YES

Study ID No.: | | | |

K	W	M	C	H

Details		
Is Abdominal Pain present?	☐	☐
Is Abdominal Distension present?	☐	☐
Is Fever present?	☐	☐
Is Lethargy present?	☐	☐
Is Vomiting present?<br>If Yes, number of vomiting per day ☐☐	☐	☐
Are other Abnormalities present?<br>If Yes,<br>specify____	☐	☐
History of mucus in stools	☐	☐
History of blood in stools	☐	☐
History of rice watery stools	☐	☐

If the baby is breastfed NO ☐ YES ☐ If YES, How many times (per day) ☐☐

If the baby is having formula milk: NO☐ YES ☐ If YES, Amount per day ☐☐☐☐ ml

STATUS OF OVERALL FOOD/ MILK INTAKE *(1 Hour before day ends)*

Mention food/Milk intake status of child as per scores provided below :<br>(1=Normal, 2=Mildly decreased, 3=Moderately decreased, 4=Severely decreased, 5=Refused to eat)	☐

STATUS OF DEHYDRATION *(1 Hour before day ends)*

Dehydration Status of child as per score provided below :<br>(0 =no signs of dehydration 1 =signs of Some dehydration 2 =signs of Severe*)<br>**If Dehydration is severe please exclude the subject from the study*	☐

CRF 14

ADVERSE EVENTS AND CONCOMITANT MEDICATIONS

Were there any changes in Adverse Events or Concomitant Medications since the last visit?<br>*If yes, please add new events/medications on appropriate log*	☐ No	☐ Yes

Reaction of Child after intake of Study Product (Fibersol-2/Placebo):
For 1st dose of the day 02
Amount of intake of Fibersol-2/Placebo in 30 mins: ☐.☐ **gm**

Study ID No.: | | | |

K	W	M	C	H

Description	No	Yes
Is Abdominal Distension present?	☐	☐
Is Abdominal Pain present?	☐	☐
Is Abdominal Rumbling present?	☐	☐
Is Abdominal Bloating present?	☐	☐
Child moved head away from the food?	☐	☐
Mouth clamped shut?	☐	☐
Teeth clenched?	☐	☐
Became agitated?	☐	☐
Spit out of the given drink?	☐	☐
Refused to swallow the given drink?	☐	☐

For 2nd dose of the day 02
Amount of intake of Fibersol-2/Placebo in 30 mins: ☐ . ☐ gm

Description	No	Yes
Is Abdominal Distension present?	☐	☐
Is Abdominal Pain present?	☐	☐
Is Abdominal Rumbling present?	☐	☐
Is Abdominal Bloating present?	☐	☐
Child moved head away from the food?	☐	☐
Mouth clamped shut?	☐	☐
Teeth clenched?	☐	**CRF 15**
Became agitated?	☐	☐
Spit out of the given drink?	☐	☐
Refused to swallow the given drink?	☐	☐

Study ID No.: | | | |

K	W	M	C	H

Is vomiting present?<br>If yes, frequency per day? ☐☐<br><br>Amount of vomiting per day ☐☐☐ gm	**No** ☐ **Yes** ☐
In mother's opinion, the child likes Fibersol-2/placebo on not?	☐

*** Children were considered as refusing intake if they moved their head away from the food, cried, clamped the mouth shut or clenched the teeth, or became agitated, spit out the food or refused to swallow. The amount of food ingested was calculated by subtracting the left-over from the offered amount. Pre-weighed napkins were provided; any food that was regurgitated, vomited or spilled was swabbed, the napkin weighed and subtracted from the weight of the amount offered. Using a 7-point Hedonic Scale in which each point (1 = disliked extremely, 2 = disliked moderately, 3 = disliked, 4 = neither disliked nor liked, 5 = liked slightly, 6 = liked moderately, 7 = liked extremely) was depicted by a facial drawing, we asked mothers to rate the food's color, flavor, mouth feel, and overall acceptability.

Stool Consistency:	☐

* Stool Consistency: 1 =Solid 2 =Paste 3 =Loose/watery 4 =Loose with blood

CRF 16

STUDY DAY 3

**Date of Visit** (DD MON YY)	**Time of Visit** (HH :MM)
☐☐ ☐☐☐ ☐☐	☐☐ : ☐☐

Study ID No.: [| |]

K	W	M	C	H

STUDY PRODUCT ADMINISTRATION SCHEDULE

Dose	Date (DD MON YY)	Time (HH :MM)	Volume Administered (ml)	Volume Remaining (ml)
**1**	☐☐ ☐☐☐ ☐☐	☐☐ : ☐☐	☐☐	☐☐

STUDY PRODUCT ADMINISTRATION SCHEDULE

Dose	Date (DD MON YY)	Time (HH :MM)	Volume Administered (ml)	Volume Remaining (ml)
**2**	☐☐ ☐☐☐ ☐☐	☐☐ : ☐☐	☐☐	☐☐

VITAL SIGNS

**Variables Time measured**	**Pulse Rate**(/Min)	**Respiratory Rate** (/Min)	**Axillary Temp** (°C)	**B.P. Systolic** (mmHg) [morning only]	**B.P. Diastolic** (mmHg) [morning only]
Is any reading outside of normal range?	☐ No ☐ Yes	☐ No ☐ Yes	☐ No ☐ Yes	☐ No ☐ Yes	☐ No ☐ Yes
IF YES, is the deviation Clinically Significant?*	☐ No ☐ Yes	☐ No ☐ Yes	☐ No ☐ Yes	☐ No ☐ Yes	☐ No ☐ Yes

Notes: *If Clinically Significant please fill up the Adverse Event Form as applicable.*

CRF 17

Other Symptoms like as:

Details	NO	YES
Is Abdominal Pain present?	☐	☐

Study ID No.: | | | |

K	W	M	C	H

Is Abdominal Distension present?	☐	☐
Is Fever present?	☐	☐
Is Lethargy present?	☐	☐
Is Vomiting present?<br>If Yes, number of vomiting per day ☐☐	☐	☐
Are other Abnormalities present?<br>If Yes,<br>specify____	☐	☐
History of mucus in stools	☐	☐
History of blood in stools	☐	☐
History of rice watery stools	☐	☐

If the baby is breastfed NO ☐ YES ☐ If YES, How many times (per day) ☐☐

If the baby is having formula milk: NO ☐ YES ☐ If YES, Amount per day ☐☐☐☐ ml

STATUS OF OVERALL FOOD/ MILK INTAKE *(1 Hour before day ends)*

Mention food/Milk intake status of child as per scores provided below :<br>(1=Normal, 2=Mildly decreased, 3=Moderately decreased, 4=Severely decreased, 5=Refused to eat)	☐

STATUS OF DEHYDRATION *(1 Hour before day ends)*

Dehydration Status of child as per score provided below :<br>(0 =no signs of dehydration 1 =signs of Some dehydration 2 =signs of Severe dehydration*)<br>**If Dehydration is severe please exclude the subject from the study*	☐

ADVERSE EVENTS AND CONCOMITANT MEDICATIONS

CRF 18

Were there any changes in Adverse Events or Concomitant Medications since the last visit?<br>*If yes, please add new events/medications on appropriate log*	☐ No	☐ Yes

Reaction of Child after intake of Study Product (Fibersol-2/Placebo):
For 1st dose of the day 03
Amount of intake of Fibersol-2/Placebo in 30 mins: ☐ . ☐ **gm**

**Description**	No	Yes

Study ID No.: | | | |

K	W	M	C	H

Is Abdominal Distension present?	☐	☐
Is Abdominal Pain present?	☐	☐
Is Abdominal Rumbling present?	☐	☐
Is Abdominal Bloating present?	☐	☐
Child moved head away from the food?	☐	☐
Mouth clamped shut?	☐	☐
Teeth clenched?	☐	☐
Became agitated?	☐	☐
Spit out of the given drink?	☐	☐
Refused to swallow the given drink?	☐	☐

For 2nd dose of the day 03
Amount of intake of Fibersol-2/Placebo in 30 mins: ☐ . ☐ gm

Description	No	Yes
Is Abdominal Distension present?	☐	☐
Is Abdominal Pain present?	☐	☐
Is Abdominal Rumbling present?	☐	☐
Is Abdominal Bloating present?	☐	☐
Child moved head away from the food?	☐	☐
Mouth clamped shut?	☐	☐
Teeth clenched?	☐	☐
Became agitated?	☐	☐
Spit out of the given drink?	☐	☐
Refused to swallow the given drink?	☐	☐

CRF 19

Is vomiting present?<br>If yes, frequency per day? ☐☐	**No** ☐ **Yes** ☐

Study ID No.: | | | |

K	W	M	C	H

Amount of vomiting per day gm	
In mother's opinion, the child likes Fibersol-2/placebo on not?	☐

*** Children were considered as refusing intake if they moved their head away from the food, cried, clamped the mouth shut or clenched the teeth, or became agitated, spit out the food or refused to swallow. The amount of food ingested was calculated by subtracting the left-over from the offered amount. Pre-weighed napkins were provided; any food that was regurgitated, vomited or spilled was swabbed, the napkin weighed and subtracted from the weight of the amount offered. Using a 7-point Hedonic Scale in which each point (1 = disliked extremely, 2 = disliked moderately, 3 = disliked, 4 = neither disliked nor liked, 5 = liked slightly, 6 = liked moderately, 7 = liked extremely) was depicted by a facial drawing, we asked mothers to rate the food's color, flavor, mouth feel, and overall acceptability.

Stool Consistency :	☐

* Stool Consistency: 1 =Solid 2 =Paste 3 =Loose/watery 4 =Loose with blood

CRF 20

STUDY DAY 4

**Date of Visit** (DD MON YY)	**Time of Visit** (HH :MM)
☐☐ ☐☐☐ ☐☐	☐☐ : ☐☐

Study ID No.: | | | |

K	W	M	C	H

STUDY PRODUCT ADMINISTRATION SCHEDULE

Dose	Date (DD MON YY)	Time (HH :MM)	Volume Administered (ml)	Volume Remaining (ml)
**1**		:		

STUDY PRODUCT ADMINISTRATION SCHEDULE

Dose	Date (DD MON YY)	Time (HH :MM)	Volume Administered (ml)	Volume Remaining (ml)
**2**		:		

VITAL SIGNS

**Variables Time measured**	**Pulse Rate**(/Min)	**Respiratory Rate** (/Min)	**Axillary Temp** (°C)	**B.P. Systolic** (mmHg) [morning only]	**B.P. Diastolic** (mmHg) [morning only]
Is any reading outside of normal range?	☐ No ☐ Yes	☐ No ☐ Yes	☐ No ☐ Yes	☐ No ☐ Yes	☐ No ☐ Yes
IF YES, is the deviation Clinically Significant?*	☐ No ☐ Yes	☐ No ☐ Yes	☐ No ☐ Yes	☐ No ☐ Yes	☐ No ☐ Yes

**If Clinically Significant please fill up the Adverse Event Form as applicable.*

CRF 21

Other Symptoms like as:

**Details**	**NO**	**YES**
Is Abdominal Pain present?	☐	☐

Study ID No.: | | | |

K	W	M	C	H

Is Abdominal Distension present?	☐	☐
Is Fever present?	☐	☐
Is Lethargy present?	☐	☐
Is Vomiting present?<br>If Yes, number of vomiting per day ☐☐	☐	☐
Are other Abnormalities present?<br>If Yes,<br>specify____	☐	☐
History of mucus in stools	☐	☐
History of blood in stools	☐	☐
History of rice watery stools	☐	☐

If the baby is breastfed NO ☐ YES ☐ If YES, How many times (per day) ☐☐

If the baby is having formula milk: NO ☐ YES ☐ If YES, Amount per day ☐☐☐☐

STATUS OF OVERALL FOOD/ MILK INTAKE *(1 Hour before day ends)*

Mention food/Milk intake status of child as per scores provided below :<br>(1=Normal, 2=Mildly decreased, 3=Moderately decreased, 4=Severely decreased, 5=Refused to eat)	☐

STATUS OF DEHYDRATION *(1 Hour before day ends)*

Dehydration Status of child as per score provided below :<br>(0 =no signs of dehydration 1 =signs of Some dehydration 2 =signs of Severe dehydration*)<br>**If Dehydration is severe please exclude the subject from the study*	☐

ADVERSE EVENTS AND CONCOMITANT MEDICATIONS **CRF 22**

Were there any changes in Adverse Events or Concomitant Medications since the last visit?<br>*If yes, please add new events/medications on appropriate log*	☐ No	☐ Yes

Reaction of Child after intake of Study Product (Fibersol-2/Placebo):
For 1st dose of the day 04
Amount of intake of Fibersol-2/Placebo in 30 mins: ☐ . ☐ **gm**

**Description**	No	Yes
	☐	☐

Study ID No.: | | | |

K	W	M	C	H

Is Abdominal Distension present?		
Is Abdominal Pain present?		
Is Abdominal Rumbling present?		
Is Abdominal Bloating present?		
Child moved head away from the food?	☐	☐
Mouth clamped shut?	☐	☐
Teeth clenched?	☐	☐
Became agitated?	☐	☐
Spit out of the given drink?	☐	☐
Refused to swallow the given drink?	☐	☐

For 2nd dose of the day 04
Amount of intake of Fibersol-2/Placebo in 30 mins: ☐ . ☐ gm

Description	No	Yes
Is Abdominal Distension present?	☐	☐
Is Abdominal Pain present?	☐	☐
Is Abdominal Rumbling present?	☐	☐
Is Abdominal Bloating present?	☐	☐
Child moved head away from the food?	☐	☐
Mouth clamped shut?	☐	☐
Teeth clenched?	☐	☐
Became agitated?	☐	☐
Spit out of the given drink?	☐	☐
Refused to swallow the given drink?	☐	☐

CRF 23

Is vomiting present?		

Study ID No.: | | | |

K	W	M	C	H

If yes, frequency per day? বার<br>Amount of vomiting per day ☐☐☐ gm	**No** ☐ **Yes** ☐
In mother's opinion, the child likes Fibersol-2/placebo on not?	☐

*** Children were considered as refusing intake if they moved their head away from the food, cried, clamped the mouth shut or clenched the teeth, or became agitated, spit out the food or refused to swallow. The amount of food ingested was calculated by subtracting the left-over from the offered amount. Pre-weighed napkins were provided; any food that was regurgitated, vomited or spilled was swabbed, the napkin weighed and subtracted from the weight of the amount offered. Using a 7-point Hedonic Scale in which each point (1 = disliked extremely, 2 = disliked moderately, 3 = disliked, 4 = neither disliked nor liked, 5 = liked slightly, 6 = liked moderately, 7 = liked extremely) was depicted by a facial drawing, we asked mothers to rate the food's color, flavor, mouth feel, and overall acceptability.

Stool Consistency :	☐

* Stool Consistency: 1 =Solid 2 =Paste 3 =Loose/watery 4 =Loose with blood

CRF 24

STUDY DAY 5

**Date of Visit** (DD MON YY)	**Time of Visit** (HH :MM)
☐☐ ☐☐☐ ☐☐	☐☐ : ☐☐

STUDY PRODUCT ADMINISTRATION SCHEDULE

Study ID No.:

K	W	M	C	H

Dose	Date (DD MON YY)	Time (HH :MM)	Volume Administered	Volume Remaining
Dose	Date	Time	Volume	Volume
2		:		

STUDY PRODUCT ADMINISTRATION SCHEDULE

VITAL SIGNS

**Variables Time measured**	**Pulse Rate**(/Min)	**Respiratory Rate** (/Min)	**Axillary Temp** (°C)	**B.P. Systolic** (mmHg) [morning only]	**B.P. Diastolic** (mmHg) [morning only]
Is any reading outside of normal range?	No / Yes	No / Yes	No / Yes	No / Yes	No / Yes
IF YES, is the deviation Clinically Significant?*	No / Yes	No / Yes	No / Yes	No / Yes	No / Yes

**If Clinically Significant please fill up the Adverse Event Form as applicable.*

CRF 25

Other Symptoms like as:

**Details**	**NO**	**YES**
Is Abdominal Pain present?	☐	☐
Is Abdominal Distension present?	☐	☐
Is Fever present?	☐	☐
Is Lethargy present?	☐	☐
Is Vomiting present?	☐	☐

Study ID No.: | | | |

K	W	M	C	H

If Yes, number of vomiting per day		
Are other Abnormalities present?<br>If Yes,<br>specify___________________________	☐	☐
History of mucus in stools	☐	☐
History of blood in stools	☐	☐
History of rice watery stools	☐	☐

If the baby is breastfed NO ☐ YES ☐ If YES, How many times (per day) ☐☐

If the baby is having formula milk: NO ☐ YES ☐ If YES, Amount per day ☐☐☐☐

STATUS OF OVERALL FOOD/ MILK INTAKE *(1 Hour before day ends)*

Mention food/Milk intake status of child as per scores provided below :<br>(1=Normal, 2=Mildly decreased, 3=Moderately decreased, 4=Severely decreased, 5=Refused to eat)	☐

STATUS OF DEHYDRATION *(1 Hour before day ends)*

Dehydration Status of child as per score provided below :<br>(0 =no signs of dehydration 1 =signs of Some dehydration 2 =signs of Severe dehydration*)<br>**If Dehydration is severe please exclude the subject from the study*	☐

ADVERSE EVENTS AND CONCOMITANT MEDICATIONS

CRF 26

Were there any changes in Adverse Events or Concomitant Medications since the last visit?<br>*If yes, please add new events/medications on appropriate log*	☐ No	☐ Yes

Reaction of Child after intake of Study Product (Fibersol-2/Placebo):
For 1st dose of the day 05
Amount of intake of Fibersol-2/Placebo in 30 mins: ☐ . ☐ gm

Description	No	Yes
Is Abdominal Distension present?	☐	☐
Is Abdominal Pain present?	☐	☐
Is Abdominal Rumbling present?	☐	☐
Is Abdominal Bloating present?	☐	☐

Study ID No.: | | | |

K	W	M	C	H

Child moved head away from the food?	☐	☐
Mouth clamped shut?	☐	☐
Teeth clenched?	☐	☐
Became agitated?	☐	☐
Spit out of the given drink?	☐	☐
Refused to swallow the given drink?	☐	☐

For 2nd dose of the day 05
Amount of intake of Fibersol-2/Placebo in 30 mins: ☐ . ☐ gm

Description	No	Yes
Is Abdominal Distension present?	☐	☐
Is Abdominal Pain present?	☐	☐
Is Abdominal Rumbling present?	☐	☐
Is Abdominal Bloating present?	☐	☐
Child moved head away from the food?	☐	☐
Mouth clamped shut?	☐	☐
Teeth clenched?	☐	☐
Became agitated?	☐	**CRF 27**
Spit out of the given drink?	☐	☐
Refused to swallow the given drink?	☐	☐

Is vomiting present?<br>If yes, frequency per day? ☐☐<br>Amount of vomiting per day ☐☐☐ gm	**No** ☐ **Yes** ☐
In mother's opinion, the child likes Fibersol-2/placebo on not?	☐

*** Children were considered as refusing intake if they moved their head away from the food, cried, clamped the mouth shut or clenched the teeth, or became agitated, spit out the food or refused to swallow. The amount of food ingested was calculated by

Study ID No.: | | | |

K	W	M	C	H

subtracting the left-over from the offered amount. Pre-weighed napkins were provided; any food that was regurgitated, vomited or spilled was swabbed, the napkin weighed and subtracted from the weight of the amount offered. Using a 7-point Hedonic Scale in which each point (1 = disliked extremely, 2 = disliked moderately, 3 = disliked, 4 = neither disliked nor liked, 5 = liked slightly, 6 = liked moderately, 7 = liked extremely) was depicted by a facial drawing, we asked mothers to rate the food's color, flavor, mouth feel, and overall acceptability.

Stool Consistency:	☐

* Stool Consistency: 1 =Solid 2 =Paste 3 =Loose/watery 4 =Loose with blood

CRF 28

STUDY DAY 6

**Date of Visit** (DD MON YY)	**Time of Visit** (HH :MM)
☐☐ ☐☐☐ ☐☐	☐☐ : ☐☐

STUDY PRODUCT ADMINISTRATION SCHEDULE

Study ID No.: | | | |

K	W	M	C	H

Dose	Date (DD MON YY)	Time (HH :MM)	Volume Administered (ml)	Volume Remaining (ml)
2		:		

STUDY PRODUCT ADMINISTRATION SCHEDULE

VITAL SIGNS

**Variables** **Time measured**	**Pulse Rate**(/Min)	**Respiratory Rate** (/Min)	**Axillary Temp** (°C)	**B.P. Systolic** (mmHg) [morning only]	**B.P. Diastolic** (mmHg) [morning only]
Is any reading outside of normal range?	☐ No ☐ Yes	☐ No ☐ Yes	☐ No ☐ Yes	☐ No ☐ Yes	☐ No ☐ Yes
IF YES, is the deviation Clinically Significant?*	☐ No ☐ Yes	☐ No ☐ Yes	☐ No ☐ Yes	☐ No ☐ Yes	☐ No ☐ Yes

**If Clinically Significant please fill up the Adverse Event Form as applicable.*

CRF 29

Other Symptoms like:

**Details**	**NO**	**YES**
Is Abdominal Pain present?	☐	☐
Is Abdominal Distension present?	☐	☐
Is Fever present?	☐	☐
Is Lethargy present?	☐	☐
Is Vomiting present? If Yes, number of vomiting per day	☐	☐

Study ID No.: | | | |

K	W	M	C	H

Are other Abnormalities present?<br>If Yes,<br>specify________________________	☐	☐
History of mucus in stools	☐	☐
History of blood in stools	☐	☐
History of rice watery stools	☐	☐

If the baby is breastfed NO ☐ YES ☐ If YES, How many times (per day) ☐☐

If the baby is having formula milk: NO ☐ YES ☐ If YES, Amount per day ☐☐☐☐ ml

STATUS OF OVERALL FOOD/ MILK INTAKE *(1 Hour before day ends)*

Mention food/Milk intake status of child as per scores provided below :<br>(1=Normal, 2=Mildly decreased, 3=Moderately decreased, 4=Severely decreased, 5=Refused to eat)	☐

STATUS OF DEHYDRATION *(1 Hour before day ends)*

Dehydration Status of child as per score provided below :<br>(0 =no signs of dehydration 1 =signs of Some dehydration 2 =signs of Severe dehydration*)<br>**If Dehydration is severe please exclude the subject from the study*	☐

ADVERSE EVENTS AND CONCOMITANT MEDICATIONS

CRF 30

Were there any changes in Adverse Events or Concomitant Medications since the last visit?<br>*If yes, please add new events/medications on appropriate log*	☐ No	☐ Yes

Reaction of Child after intake of Study Product (Fibersol-2/Placebo):
For 1st dose of the day 06
Amount of intake of Fibersol-2/Placebo in 30 mins: ☐ . ☐ **gm**

**Description**	No	Yes
Is Abdominal Distension present?	☐	☐
Is Abdominal Pain present?	☐	☐
Is Abdominal Rumbling present?	☐	☐
Is Abdominal Bloating present?	☐	☐

Study ID No.: | | | |

K	W	M	C	H

Child moved head away from the food?	☐	☐
Mouth clamped shut?	☐	☐
Teeth clenched?	☐	☐
Became agitated?	☐	☐
Spit out of the given drink?	☐	☐
Refused to swallow the given drink?	☐	☐

For 2nd dose of the day 06
Amount of intake of Fibersol-2/Placebo in 30 mins: ☐ . ☐ gm

Description	No	Yes
Is Abdominal Distension present?	☐	☐
Is Abdominal Pain present?	☐	☐
Is Abdominal Rumbling present?	☐	☐
Is Abdominal Bloating present?	☐	☐
Child moved head away from the food?	☐	☐
Mouth clamped shut?	☐	☐
Teeth clenched?	☐	**CRF 31**
Became agitated?	☐	☐
Spit out of the given drink?	☐	☐
Refused to swallow the given drink?	☐	☐

Is vomiting present?<br>If yes, frequency per day? ☐☐<br>Amount of vomiting per day ☐☐☐ gm	**No** ☐ **Yes** ☐
In mother's opinion, the child likes Fibersol-2/placebo on not?	☐

*** Children were considered as refusing intake if they moved their head away from the food, cried, clamped the mouth shut or clenched the teeth, or became agitated, spit out the food or refused to swallow. The amount of food ingested was calculated by subtracting the left-over from the offered amount. Pre-weighed napkins were provided; any food that was regurgitated, vomited or spilled was swabbed, the napkin weighed and subtracted from the weight of the amount offered. Using a 7-point Hedonic Scale in which each point (1 = disliked extremely, 2 = disliked moderately, 3 = disliked, 4 = neither disliked nor liked, 5 = liked slightly,

Study ID No.: | | | |

K	W	M	C	H

6 = liked moderately, 7 = liked extremely) was depicted by a facial drawing, we asked mothers to rate the food's color, flavor, mouth feel, and overall acceptability.

Stool Consistency:	☐

* Stool Consistency: 1 =Solid 2 =Paste 3 =Loose/watery 4 =Loose with blood

CRF 32

STUDY DAY 7

**Date of Visit** (DD MON YY)	**Time of Visit** (HH :MM)
☐☐ ☐☐☐ ☐☐	☐☐ : ☐☐

STUDY PRODUCT ADMINISTRATION SCHEDULE

Dose	**Date** (DD MON YY)	**Time** (HH :MM)	Volume Administered (ml)	Volume Remaining (ml)
**1**	☐☐ ☐☐☐ ☐☐	☐☐ : ☐☐	☐☐	☐☐

Study ID No.: [ ][ ][ ]

K	W	M	C	H

STUDY PRODUCT ADMINISTRATION SCHEDULE

Dose	**Date** (DD MON YY)	**Time** (HH :MM)	Volume Administered (ml)	Volume Remaining (ml)
**2**	[ ][ ] [ ][ ][ ] [ ][ ]	[ ][ ] : [ ][ ]	[ ][ ]	[ ][ ]

VITAL SIGNS

**Variables Time measured**	**Pulse Rate**(/Min)	**Respiratory Rate** (/Min)	**Axillary Temp** (°C)	**B.P. Systolic** (mmHg) [morning only]	**B.P. Diastolic** (mmHg) [morning only]
Is any reading outside of normal range?	☐ No ☐ Yes	☐ No ☐ Yes	☐ No ☐ Yes	☐ No ☐ Yes	☐ No ☐ Yes
IF YES, is the deviation Clinically Significant?*	☐ No ☐ Yes	☐ No ☐ Yes	☐ No ☐ Yes	☐ No ☐ Yes	☐ No ☐ Yes

***If Clinically Significant please fill up the Adverse Event Form as applicable.**

CRF 33

Other Symptoms like:

**Details**	**NO**	**YES**
Is Abdominal Pain present?	☐	☐
Is Abdominal Distension present?	☐	☐
Is Fever present?	☐	☐
Is Lethargy present?	☐	☐
Is Vomiting present?<br>If Yes, number of vomiting per day [ ][ ]	☐	☐

Study ID No.: | | | |

K	W	M	C	H

Are other Abnormalities present?<br>If Yes,<br>specify____________________	☐	☐
History of mucus in stools	☐	☐
History of blood in stools	☐	☐
History of rice watery stools	☐	☐

If the baby is breastfed NO ☐ YES ☐ If YES, How many times (per day) ☐☐

If the baby is having formula milk: NO ☐ YES ☐ If YES, Amount per day ☐☐☐☐ ml

STATUS OF OVERALL FOOD/ MILK INTAKE *(1 Hour before day ends)*

Mention food/Milk intake status of child as per scores provided below :<br>(1=Normal, 2=Mildly decreased, 3=Moderately decreased, 4=Severely decreased, 5=Refused to eat)	☐

STATUS OF DEHYDRATION *(1 Hour before day ends)*

Dehydration Status of child as per score provided below :<br>(0 =signs of no dehydration 1 =signs of Some dehydration 2 =signs of Severe dehydration*)<br>**If Dehydration is severe please exclude the subject from the study*	☐

ADVERSE EVENTS AND CONCOMITANT MEDICATIONS

CRF 34

Were there any changes in Adverse Events or Concomitant Medications since the last visit?<br>*If yes, please add new events/medications on appropriate log*	☐ No	☐ Yes

Reaction of Child after intake of Study Product (Fibersol-2/Placebo):
For 1st dose of the day 07
Amount of intake of Fibersol-2/Placebo in 30 mins: ☐ . ☐ **gm**

**Description**	No	Yes
Is Abdominal Distension present?	☐	☐
Is Abdominal Pain present?	☐	☐
Is Abdominal Rumbling present?	☐	☐
Is Abdominal Bloating present?	☐	☐

Study ID No.: | | | |

K	W	M	C	H

Child moved head away from the food?	☐	☐
Mouth clamped shut?	☐	☐
Teeth clenched?	☐	☐
Became agitated?	☐	☐
Spit out of the given drink?	☐	☐
Refused to swallow the given drink?	☐	☐

For 2nd dose of the day 07
Amount of intake of Fibersol-2/Placebo in 30 mins: ☐ . ☐ gm

Description	No	Yes
Is Abdominal Distension present?	☐	☐
Is Abdominal Pain present?	☐	☐
Is Abdominal Rumbling present?	☐	☐
Is Abdominal Bloating present?	☐	☐
Child moved head away from the food?	☐	☐
Mouth clamped shut?	☐	☐
Teeth clenched?	☐	☐
Became agitated?	☐	**CRF 35**
Spit out of the given drink?	☐	☐
Refused to swallow the given drink?	☐	☐

Is vomiting present?<br>If yes, frequency per day? ☐☐<br>Amount of vomiting per day ☐☐☐ gm	**No** ☐ **Yes** ☐
In mother's opinion, the child likes Fibersol-2/placebo on not?	☐

*** Children were considered as refusing intake if they moved their head away from the food, cried, clamped the mouth shut or clenched the teeth, or became agitated, spit out the food or refused to swallow. The amount of food ingested was calculated by

Study ID No.: | | | |

K	W	M	C	H

subtracting the left-over from the offered amount. Pre-weighed napkins were provided; any food that was regurgitated, vomited or spilled was swabbed, the napkin weighed and subtracted from the weight of the amount offered. Using a 7-point Hedonic Scale in which each point (1 = disliked extremely, 2 = disliked moderately, 3 = disliked, 4 = neither disliked nor liked, 5 = liked slightly, 6 = liked moderately, 7 = liked extremely) was depicted by a facial drawing, we asked mothers to rate the food's color, flavor, mouth feel, and overall acceptability.

Stool Consistency :	☐

* Stool Consistency: 1 =Solid 2 =Paste 3 =Loose/watery 4 =Loose with blood

CRF 36

DAY 8: FOLLOW- UP QUESTIONNAIRES

Date of Follow up Visit: ☐☐ ☐☐☐ ☐☐
DD MON YY

Was telephonic contact made with the parent/legal guardian? ☐ No ☐ Yes

Other symtoms like as:

**Details**	**NO**	**YES**
Is Abdominal Distension present?	☐	☐
Is Abdominal Pain present?	☐	☐

Study ID No.: | | | |

K	W	M	C	H

Is Abdominal Rumbling present?	☐	☐
Is Abdominal Bloating present?	☐	☐
Is Fever present?	☐	☐
Is Lethargy present?	☐	☐
Is Vomiting present?<br>If Yes, number of vomiting per day ☐☐	☐	☐
Are other Abnormalities present?<br>If Yes, pecify________________________________	☐	☐
History of mucus in stools	☐	☐
History of blood in stools	☐	☐
History of rice watery stools	☐	☐

STATUS OF OVERALL FOOD / MILK INTAKE

☐ **(Milk 1-5 as per score provided below)**

Score	Food / Milk Intake	Frequency of Milk Intake per day
1	Normal	☐
2	Mildly decreased	
3	Moderately decreased	
4	Severely decreased	
5	Refused to eat	
In case of milk Intake, Please mention ☐ Breast fed ☐ Formula Milk ☐ Both		

If the baby passes any stool in 24 hours? NO ☐ **YES** ☐ **if YES then how many times** ☐☐

Stool consistancy ☐

CRF 37

Stool Consistancy: (1=Hard, 2=Soft, 3=Rice watery stool, 4=Stool mixed with blood and 9=NA	

STATUS OF DEHYDRATION *(1 Hour before day ends)*

Dehydration Status of child as per score provided below :<br>(0 =signs of no dehydration 1 =signs of Some dehydration 2 =signs of Severe dehydration*)<br><br>**If Dehydration is severe please exclude the subject from the study*	☐

DAY 9: FOLLOW- UP QUESTIONNAIRES

Date of Follow up Visit: ☐☐ (DD) ☐☐☐ (MON) ☐☐ (YY)

Was telephonic contact made with the parent/legal guardian? ☐ No ☐ Yes

Other symtoms like as:

Study ID No.: [| |]

K	W	M	C	H

Details	NO	YES
Is Abdominal Distension present?	☐	☐
Is Abdominal Pain present?	☐	☐
Is Abdominal Rumbling present?	☐	☐
Is Abdominal Bloating present?	☐	☐
Is Fever present?	☐	☐
Is Lethargy present?	☐	☐
Is Vomiting present?<br>If Yes, number of vomiting per day [|]	☐	☐
Are other Abnormalities present?<br>If Yes, specify________________________________	☐	☐
History of mucus in stools	☐	☐
History of blood in stools	☐	☐
History of rice watery stools	☐	☐

STATUS OF OVERALL FOOD / MILK INTAKE
☐ **(Milk 1-5 as per score provided below)**

Score	Food / Milk Intake	Frequency of Milk Intake per day
1	Normal	☐
2	Mildly decreased	
3	Moderately decreased	
4	Severely decreased	
5	Refused to eat	

In case of milk Intake, Please mention ☐ Breast fed ☐ Formula Milk ☐ Both

If the baby passes any stool in 24 hours? NO ☐ **YES** ☐ **if YES then how many times** [|]
Stool consistancy ☐

Stool Consistancy: (1=Hard, 2=Soft, 3=Rice watery stool, 4=Stool mixed with blood and 9= NA ☐

STATUS OF DEHYDRATION *(1 Hour before day ends)*

CRF 38

Dehydration Status of child as per score provided below : ☐
(0 =no signs of dehydration 1 =signs of Some dehydration 2 =signs of Severe dehydration *)
**If Dehydration is severe please exclude the subject from the study*

DAY 10: FOLLOW- UP QUESTIONNAIRES

Date of Follow up Visit: [|] DD [| |] MON [|] YY

Study ID No.: ☐☐☐

K	W	M	C	H

Was telephonic contact made with the parent/legal guardian?	☐ No ☐ Yes

Other symtoms like as:

Details	NO	YES
Is Abdominal Distension present?	☐	☐
Is Abdominal Pain present?	☐	☐
Is Abdominal Rumbling present?	☐	☐
Is Abdominal Bloating present?	☐	☐
Is Fever present?	☐	☐
Is Lethargy present?	☐	☐
Is Vomiting present?<br>If Yes, number of vomiting per day ☐☐	☐	☐
Are other Abnormalities present?<br>If Yes, specify____	☐	☐
History of mucus in stools	☐	☐
History of blood in stools	☐	☐
History of rice watery stools	☐	☐

STATUS OF OVERALL FOOD / MILK INTAKE

☐ **(Milk 1-5 as per score provided below)**

Score	Food / Milk Intake	Frequency of Milk Intake per day
1	Normal	☐
2	Mildly decreased	
3	Moderately decreased	
4	Severely decreased	
5	Refused to eat	
In case of milk Intake, Please mention	☐ Breast fed ☐ Formula Milk ☐ Both	

If the baby passes any stool in 24 hours? NO ☐ **YES** ☐ **if YES then how many times** ☐☐
Stool consistancy ☐

Stool Consistancy: (1=Hard, 2=Soft, 3=Rice watery stool, 4=Stool mixed with blood and 9=NA	

Study ID No.: ☐☐☐

K	W	M	C	H

STATUS OF DEHYDRATION *(1 Hour before day ends)*

Dehydration Status of child as per score provided below :<br>(0 =signs of no dehydration 1 =signs of Some dehydration 2 =signs of Severe dehydration*)<br>**If Dehydration is severe please exclude the subject from the study*	**CRF 39**

DAY 11: FOLLOW- UP QUESTIONNAIRES

Date of Follow up Visit: ☐☐ ☐☐☐ ☐☐ (DD MON YY)

Was telephonic contact made with the parent/legal guardian? ☐ No ☐ Yes

Other symtoms like as:

Details	NO	YES
Is Abdominal Distension present?	☐	☐
Is Abdominal Pain present?	☐	☐
Is Abdominal Rumbling present?	☐	☐
Is Abdominal Bloating present?	☐	☐
Is Fever present?	☐	☐
Is Lethargy present?	☐	☐
Is Vomiting present?<br>If Yes, number of vomiting per day ☐☐	☐	☐
Are other Abnormalities present?<br>If Yes, specify________________________________	☐	☐
History of mucus in stools	☐	☐
History of blood in stools	☐	☐
History of rice watery stools	☐	☐

STATUS OF OVERALL FOOD / MILK INTAKE

☐ **(Milk 1-5 as per score provided below)**

Score	Food / Milk Intake	Frequency of Milk Intake per day
1	Normal	☐
2	Mildly decreased	
3	Moderately decreased	
4	Severely decreased	
5	Refused to eat	

Study ID No.: [| |]

K	W	M	C	H

In case of milk Intake, Please mention	☐ Breast fed ☐ Formula Milk ☐ Both

If the baby passes any stool in 24 hours? NO ☐ YES ☐ if YES then how many times [|]
Stool consistancy ☐

Stool Consistancy: (1=Hard, 2=Soft, 3=Rice watery stool, 4=Stool mixed with blood and 9=NA	

STATUS OF DEHYDRATION *(1 Hour before day ends)*

Dehydration Status of child as per score provided below :<br>(0 =signs of no dehydration 1 =signs of Some dehydration 2 =signs of Severe dehydration*)<br>**If Dehydration is severe please exclude the subject from the study*	**CRF 40**

DAY 12: FOLLOW- UP QUESTIONNAIRES

Date of Follow up Visit:	DD	MON	YY
Was telephonic contact made with the parent / legal guardian?	☐ No	☐ Yes	

Other symtoms like as:

Details	NO	YES
Is Abdominal Distension present?	☐	☐
Is Abdominal Pain present?	☐	☐
Is Abdominal Rumbling present?	☐	☐
Is Abdominal Bloating present?	☐	☐
Is Fever present?	☐	☐
Is Lethargy present?	☐	☐
Is Vomiting present?<br>If Yes, number of vomiting per day [|]	☐	☐

Study ID No.: | | | |

K	W	M	C	H

Are other Abnormalities present?<br>If Yes, specify________________________________	☐	☐
History of mucus in stools	☐	☐
History of blood in stools	☐	☐
History of rice watery stools	☐	☐

STATUS OF OVERALL FOOD / MILK INTAKE

☐ **(Milk 1-5 as per score provided below)**

Score	Food / Milk Intake	Frequency of Milk Intake per day
1	Normal	☐
2	Mildly decreased	
3	Moderately decreased	
4	Severely decreased	
5	Refused to eat	

In case of milk Intake, Please mention ☐ Breast fed ☐ Formula Milk ☐ Both

If the baby passes any stool in 24 hours? NO ☐ **YES** ☐ **if YES then how many times** ☐☐

Stool consistancy ☐

Stool Consistancy: (1=Hard, 2=Soft, 3=Rice watery stool, 4=Stool mixed with blood and 9=NA	

STATUS OF DEHYDRATION *(1 Hour before day ends)*

Dehydration Status of child as per score provided below :<br>(0 =signs of no dehydration 1 =signs of Some dehydration 2 =signs of Severe dehydration*)<br>**If Dehydration is severe please exclude the subject from the study*	**CRF 41**

DAY 13: FOLLOW- UP QUESTIONNAIRES

Date of Follow up Visit:	☐☐ DD ☐☐☐ MON ☐☐ YY
Was telephonic contact made with the parent / legal guardian?	☐ No ☐ Yes

Other symtoms like as:

**Details**	**NO**	**YES**
Is Abdominal Distension present?	☐	☐
Is Abdominal Pain present?	☐	☐
Is Abdominal Rumbling present?	☐	☐

Study ID No.: [ ][ ][ ]

K	W	M	C	H

Is Abdominal Bloating present?	☐	☐
Is Fever present?	☐	☐
Is Lethargy present?	☐	☐
Is Vomiting present?<br>If Yes, number of vomiting per day [ ][ ]	☐	☐
Are other Abnormalities present?<br>If Yes, specify______________________________	☐	☐
History of mucus in stools	☐	☐
History of blood in stools	☐	☐
History of rice watery stools	☐	☐

STATUS OF OVERALL FOOD / MILK INTAKE

☐ **(Milk 1-5 as per score provided below)**

Score	Food / Milk Intake	Frequency of Milk Intake per day
1	Normal	☐
2	Mildly decreased	
3	Moderately decreased	
4	Severely decreased	
5	Refused to eat	
In case of milk Intake, Please mention		☐ Breast fed ☐ Formula Milk ☐ Both

If the baby passes any stool in 24 hours? NO ☐ **YES** ☐ **if YES then how many times** [ ][ ]
Stool consistancy ☐

Stool Consistancy: (1=Hard, 2=Soft, 3=Rice watery stool, 4=Stool mixed with blood and 9=NA	

STATUS OF DEHYDRATION *(1 Hour before day ends)*

Dehydration Status of child as per score provided below :<br>(0 =signs of no dehydration 1 =signs of Some dehydration 2 =signs of Severe dehydration *)<br>**If Dehydration is severe please exclude the subject from the study*	**CRF 42**

DAY 14: FOLLOW- UP QUESTIONNAIRES

Date of Follow up Visit: [ ][ ] DD [ ][ ][ ] MON [ ][ ] YY

Was telephonic contact made with the parent/legal guardian? ☐ No ☐ Yes

Vital sign

**Variables**	**Weight (Kg)** [morning only]	**Pulse Rate**(/Min)	**Respiratory Rate** (/Min)	**Axillary Temp** (°C)	**B.P. Systolic** (mmHg) [morning only]	**B.P. Diastolic** (mmHg) [morning only]

Study ID No.: | | | |

K	W	M	C	H

Is any reading outside of normal range?	☐ No ☐ Yes	☐ No ☐ Yes	☐ No ☐ Yes	☐ No ☐ Yes	☐ No ☐ Yes	☐ No ☐ Yes
IF YES, is the deviation Clinically Significant?*	☐ No ☐ Yes	☐ No ☐ Yes	☐ No ☐ Yes	☐ No ☐ Yes	☐ No ☐ Yes	☐ No ☐ Yes

**If Clinically Significant please fill up the Adverse Event Form as applicable.*

Other symtoms like:

Details	NO	YES
Is Abdominal Distension present?	☐	☐
Is Abdominal Pain present?	☐	☐
Is Abdominal Rumbling present?	☐	☐
Is Abdominal Bloating present?	☐	☐
Is Fever present?	☐	☐
Is Lethargy present?	☐	☐
Is Vomiting present?<br>If Yes, number of vomiting per day ☐☐	☐	☐
Are other Abnormalities present?<br>If Yes, specify________________________________	☐	☐
History of mucus in stools	☐	☐
History of blood in stools	☐	☐
History of rice watery stools	☐	☐

CRF 43

STATUS OF OVERALL FOOD / MILK INTAKE

☐ **(Milk 1-5 as per score provided below)**

Score	Food / Milk Intake	Frequency of Milk Intake per day
1	Normal	☐
2	Mildly decreased	
3	Moderately decreased	
4	Severely decreased	

Study ID No.: | | | |

K	W	M	C	H

5	Refused to eat

In case of milk Intake, Please mention ☐ Breast fed ☐ Formula Milk ☐ Both

If the baby passes any stool in 24 hours? NO ☐ **YES** ☐ **if YES then how many times** ☐☐

Stool consistancy ☐

Stool Consistancy: 1=Hard, 2=Soft, 3=Rice watery stool, 4=Stool mixed with blood and 9=NA	

STATUS OF DEHYDRATION *(1 Hour before day ends)*

Dehydration Status of child as per score provided below :<br>(0 =signs of no dehydration 1 =signs of Some dehydration 2 =signs of Severe dehydration*)<br>**If Dehydration is severe please exclude the subject from the study*	☐

CRF 44

FINAL EVALUATION FORM (COMPLETION/DISCONTINUATION FORM)

Last day of Participation:

Study Day ☐☐ Date ☐☐ ☐☐☐ ☐☐
DD MON YY

Last day of Test Article:

Study Day ☐☐ Date ☐☐ ☐☐☐ ☐☐
DD MON YY

Study ID No.: | | | |

K	W	M	C	H

Did child complete the study ☐ No ☐ Yes

If Yes, Date of study Completion ☐☐ DD ☐☐☐ MON ☐☐ YY

If child did not complete the study, what is the reason for termination? *(Check one box only)*

- ☐ Consent withdrawn
- ☐ Lost to follow-up *(appointment failure)*
- ☐ Withdrawn for discovery of pre-existing violation of entry criteria *(specify)*_______________
- ☐ Withdrawn for protocol noncompliance *(specify)*____________________________________
- ☐ Withdrawn due to lack of improvement requiring additional hospitalization *(Specify)* ____
- ☐ Withdrawn for adverse sign of symptom.

If adverse sign or symptom is serious or death occurs, notify Medical Monitor and Sponsor immediately.

Death: Date of Death: ☐☐ DD ☐☐☐ MON ☐☐ YY

Cause(s) of Death: ____

Confirmed by Postmorterm : ☐ No ☐ Yes

Investigator's Signature ____________________ Date of Signature ☐☐ DD ☐☐☐ MON ☐☐ YY

CRF 45

PROTOCOL DEVIATION LOG

CRF Page No	Visit and CRF Date Item	Description of Deviation	Notified to EC
			☐ No<br>☐ Yes
			☐ No<br>☐ Yes
			☐ No<br>☐ Yes
			☐ No<br>☐ Yes

Study ID No.ঃ | | | |

K	W	M	C	H

			☐ No<br>☐ Yes
			☐ No<br>☐ Yes
			☐ No<br>☐ Yes
			☐ No<br>☐ Yes
			☐ No<br>☐ Yes
			☐ No<br>☐ Yes

CRF 1

সুস্থ্য শিশুর রির্পোট ফর্ম

প্রটোকল নাম্বার : PR-১৬০৯১

প্রোটোকল টাইটেলঃ ১-৩ বছরের সুস্থ্য ও ডায়রিয়া আক্রান্ত শিশুদের ক্ষেত্রে ফাইবারসল-২ (প্রতিরোধী ম্যালটোডেক্সট্রিন) এর সহনীয়তা এবং গ্রহনযোগ্যতা নিরুপনের পরবর্তীতে রেনডোমাইজড ক্লিনিক্যাল ট্রায়ালের দ্বারা ফাইবারসল-২ এর কার্যক্ষমতা যাচাই।

প্রধান গবেষক
ডাঃ মোহাম্মদ জোবায়ের চিশতি, এমবিবিএস, এম মেড, পি এইচ ডি, আই সি ডি ডি আর বি

Study ID No.ঃ | | | |

K	W	M	C	H

পৃষ্ঠপোষক
Matsutani Chemical Industry Company.

লিখিত সম্মতি পত্র

লিখিত সম্মতিপত্রের স্বাক্ষরের তারিখ: [ ][ ] [ ][ ][ ] [ ][ ]
দিন মাস বছর

লিখিত সম্মতিপত্রের স্বাক্ষরের সময়: [ ][ ] : [ ][ ]
ঘন্টা মিনিট

সম্মতিপত্রে দেওয়া স্বাক্ষর এই রোগীর সকল CRFএর জন্য:

আমি নিশ্চিত করছি যে এখানে সংগ্রহীত সকল তথ্য উপাত্ত গবেষণার প্রটোকল অনুসারে সংগ্রহ করা হয়েছে এবং লিখিত সম্মতিপত্র পিতামাতা অথবা অভিভাবক দিয়েছেন।

প্রধান গবেষকের স্বাক্ষর ঃ **তারিখ ঃ**

প্রধান গবেষকের নাম (মুদ্রন করুন): ডাঃ মোহাম্মদ জোবায়ের চিশতি

CRF 2

কেস রির্পোট ফর্মের (CRF) নির্দেশিকা ঃ
১.কালো কলম দ্বারা CRF পূরন করতে হবে।
২.CRF.পূরন করতে বড় হাতের অক্ষর ব্যবহার করুন।
৩.সকল ঘর চিহ্নিত করতে যেমন হ্যাঁ এবং না এর চিহ্ন এর সাহায্যে পূরন করতে হবে।
৪. ২৪ ঘন্টায় ঘড়ির সময় ব্যবহার করতে হবে। যেমন রাত ৯ টা হলে ২১:০০ টা লিখতে হবে।
৫.আগে শূন্য বসাতে হবে লক্ষন প্রযোজ্য যেমন রোগীর ওজন ১০.৫ কেজি হলে ০১০.৫ কেজি লিখতে হবে।

৬.কোন তথ্য জানা না থাকলে বক্সে UK লিখতে হবে : [U][K] [D][E][C] [1][4]
দিন মাস বছর

Time: [১][৯] [U][K]
ঘন্টা মিনিট

৭.যদি কোন ভূল হয়ে থাকে ?
- লেখাটি মুছে ফেলা যাবেনা ঘষাঘষি করে কাটা যাবেনা।
- ফ্লুইড ব্যবহার করা যাবেনা।
- এক টানে কাটতে হবে। 66SAS
- পুনরায় লিখতে হলে পরিস্কার ভাবে আগের লেখার কাছাকাছি লিখতে হবে ১০/০৪/১৩

Study ID No.ঃ | | | |

K	W	M	C	H

~~56~~

CRF 3

অন্তর্ভূক্ত হওয়ার পূর্বে শিশুর শারীরিক অবস্থা
[অনুগ্রহ পূর্বক গ্রাম/ নাম, অথবা যথাযথ উত্তর নিয়ে ফর্ম পূরন করুন]

পরিদর্শনের তারিখঃ: দিন মাস বছর   পরিদর্শনের সময়: ঘন্টা : মিনিট

রির্পোটি করার সময় সাধারন পরীক্ষা :
অত্যাবশ্যকীয় লক্ষণ

সূচক	বয়স [মাস]	উচ্চতা [সে.মি]	ওজন (কেজি) [শুধু মাত্র সকালে]	নাড়ীর গতি (প্রতি মিনিটে)	শ্বাসের গতি (প্রতি মিনিট)	তাপমাত্রা (বগলের)	রক্তের চাপ.Systolic (mmHg)	রক্তের চাপ Diastolic (mmHg)

Study ID No.ঃ | | | |

K	W	M	C	H

						(০সে.)	[শুধু মাত্র সকালে]	[শুধু মাত্র সকালে]
যদি কোন reading স্বাভাবিক পরিসীমা অতিক্রম করে?			☐ না ☐ হ্যাঁ	☐ না ☐ হ্যাঁ	☐ না ☐ হ্যাঁ	☐ না ☐ হ্যাঁ	☐ না ☐ হ্যাঁ	☐ না ☐ হ্যাঁ
যদি হ্যাঁ হয় তবে, reading clinically Significant কিনা?*			☐ না ☐ হ্যাঁ	☐ না ☐ হ্যাঁ	☐ না ☐ হ্যাঁ	☐ না ☐ হ্যাঁ	☐ না ☐ হ্যাঁ	☐ না ☐ হ্যাঁ

**যদি reading Clinically Significant হয় তবে প্রয়োজন হলে অনুগ্রহ করে Adverse Event Form পূরণ করুন]*

CRF 4

পানি শূন্যতার অবস্থা

নিচে উল্লেখিত তালিকা অনুযায়ী শিশুর পানি শূন্যতা :<br>(০=পানি শূন্যতা নাই ১=কিছুটা পানি শূন্যতা ২= মারাত্বক পানি শূন্যতা *)<br><br>* যদি মারাত্বক পানি শূন্যতা হয় তাহলে শিশুকে গবেষণা থেকে বাদ দিন	☐

প্রস্তুতকৃত দুধ শিশুকে দেওয়া হয়ে ছিল কিনা? ☐ না ☐ হ্যাঁ<br>যদি হ্যাঁ হয়,পরিমান ☐☐☐☐ মি.লি.<br>শিশুটি কি বুকের দুধ খায় ? ☐ না ☐ হ্যাঁ	

Study ID No.ঃ | | | |

K	W	M	C	H

যদি হ্যাঁ হয়,কত বার প্রতিদিন<br>শিশুটি কি বর্তমানে শক্ত খাবার খায়? ☐ না ☐ হ্যাঁ	

মলের ধরণ *(একটি উওর প্রযোজ্য হবে)*	১ = শক্ত	২ = নরম	৩ = পাতলা/ পানির মত	৪ = রক্তযুক্ত পাতলা পায়খানা
	☐			

[নির্দেশিকা: ১. শক্ত =নিজস্ব ধরনের শক্ত; ২. নরম = নরম এবং রোগীর শারিরীকগঠনের সাথে মানানসই; ৩. পাতলা = পানির মত এবং পরিমানে অনেক বেশি; ৪. রক্তযুক্ত পাতলা পায়খানা = রক্ত এবং মিউকাস থাকবে সাথে মল থাকতে পারে না ও থাকতে পারে]

CRF 5

অন্যান্য লক্ষন সমূহ

বর্ণনা	মায়ের মতামত		ইন্টারভিউয়ারের মতামত	
	না	হ্যাঁ	না	হ্যাঁ
পেটে ব্যথা আছে কিনা?	☐	☐	☐	☐
পেটে ফাঁপা আছে কিনা?	☐	☐	☐	☐
জ্বর আছে কিনা?	☐	☐	☐	☐
শারিরীক দুর্বলতা আছে কিনা?	☐	☐	☐	☐

Study ID No.ঃ | | | |

K	W	M	C	H

বমি আছে কিনা?<br>যদি হ্যাঁ হয়, প্রতি দিন কত বার বমি হয়? ☐☐	☐	☐	☐	☐
অন্যান্য অস্বাভাবিকতার উপস্থিতি<br>যদি হ্যাঁ হয়, নির্দিষ্ট করুন________________	☐	☐	☐	☐
পায়খানার সাথে মলের *উপস্থিতির ইতিহাস*	☐	☐	☐	☐
পায়খানার সাথে রক্তের *উপস্থিতির ইতিহাস*	☐	☐	☐	☐
চাল ধোয়া পানির মত পায়খানার ইতিহাস	☐	☐	☐	☐

সামগ্রিকভাবে খাবার এবং দুধ গ্রহনের পরিমান

খাওয়ার অবস্থা উল্লেখিত স্কোর অনুযায়ী<br>(১= স্বাভাবিক, ২= কিছুটা কম, ৩=মধ্যম কম, ৪= খুব বেশি কম,<br>৫= খাবার গ্রহনে অস্বীকৃতি)	☐

CRF 6

তাৎপর্যপূর্ণ চিকিৎসাজনিত ইতিহাস (জন্ডিস, LRTI ইত্যাদি অন্তর্ভূক্তি):
যদি না হয়, তবে এখানে চেক করুন ☐

শরীর সর্ম্পকিত	না	হ্যাঁ	যদি হ্যাঁ হয়,<br>রোগটি নির্দিষ্ট করুন(যদি জানা থাকে)
নাক,কান,গলা :	☐	☐	
চক্ষু সংক্রান্ত :	☐	☐	
স্নায়ুতান্ত্রিক :	☐	☐	
কার্ডিওভাসকুলার :	☐	☐	

Study ID No.ঃ | | | |

K	W	M	C	H

শ্বসন :	☐	☐	
গ্যাস্ট্রো–ইনটেসস্টাইনাল :	☐	☐	
মূত্র সংক্রান্ত এবং যৌনাঙ্গ সংক্রান্ত:	☐	☐	
মাংসপেশী এবং কঙ্কাল :	☐	☐	
ডার্মাটোলজিক্যাল:	☐	☐	
হরমোন সংক্রান্ত	☐	☐	
রক্তসংক্রান্ত :	☐	☐	
যকৃত সংক্রান্ত :	☐	☐	
বিপাকীয়:	☐	☐	
এলার্জি এবং ঔষধ সংবেদনশীলতা:	☐	☐	

CRF 7

অন্তর্ভূক্তি হওয়ার নিয়মাবলীর চেকলিষ্ট
অন্তর্ভুক্তির যোগ্যতা সমূহ:

অন্তর্ভূক্তি যোগ্যতার যে কোন একটি না হলে শিশুটি গবেষনায় অন্তর্ভূক্তি করা যাবেনা	না	হ্যাঁ
১.ছেলে এবং মেয়ে শিশুর বয়স ১২-৩৬ মাসের মধ্যে কিনা?	☐	☐
২. অভিভাবকগন স্বেচ্ছায় মলের পরিমান, মলের ধরন, বমির পরিমান, খাবার গ্রহন সংক্রান্ত তথ্য পরিদর্শনের সময় জানাতে রাজি আছে কিনা?	☐	☐
৩. গবেষণার অন্তর্ভুর্ক্তি হলে অবশ্যই লিখিত সম্মতি দিতে হবে?	☐	☐
৪. শিশুর শারিরীক পরীক্ষায় কোন অস্বাভাবিকতা নাই। (মারাত্বক অপুষ্টি, খিচুনি, ক্লিনিক্যাল সাসপিশিয়ান, স্যাপসিস, ম্যানেনজাইটিস, নিউমোনিয়া, দীর্ঘস্থায়ী পরিপাকতন্ত্রের রোগ, পরিপাকতন্ত্রের প্রদাহ অথবা অন্য কোন সংক্রমন যা একুয়েট ডায়রিয়ার সাথে সম্পর্কিত)	☐	☐

অন্তর্ভুর্ক্তি না হওয়ার কারনগুলো

	না	হ্যাঁ

Study ID No.ঃ | | | |

K	W	M	C	H

যদি অন্তর্ভুর্ক্তি না হওয়ার কারনগুলোর মধ্যে একটি যদি হ্যাঁ হয় তবে শিশুদের গবেষণায় অন্তর্ভুর্ক্তি করা হবে না		
১.সন্দেহজনক আমাশয় অথবা কোষ্ঠকাঠিন্য(সপ্তাহে তিন বারের কম পায়খানা হলে)	☐	☐
২. খুঁজে বের করার সময় মারাত্বক পানি শূন্যতা আছে কিনা	☐	☐
৩. মারাত্বক বমি করার জন্য ভর্তি সংক্রান্ত এবং অন্যান্য পরীক্ষা অসম্ভব কিনা	☐	☐
৪. মারাত্বক অপুষ্টি (WHO এর মান অনুযায়ী উচ্চতা ও ওজন যদি মিডিয়াম থেকে ৩ SD এর কম হয়)	☐	☐
৫. শিশুটি এন্টিবায়টিক বা সক্রিয়তা নষ্ঠ কারী কোন ঔষধ গবেষণার ২ সপ্তাহে পূর্বে খেয়েছে কিনা	☐	☐
৬. অতি সংবেদনশীলতা বা দুধ বা এলার্জি আছে কিনা	☐	☐
৭. রোটা ভাইরাস এর প্রতিশেধক নিয়েছে কিনা	☐	☐
৮. শিশুটি বর্তমানে অন্য কোন গবেষণায় অন্তর্ভুক্ত আছে কিনা অথবা শিশুটি গবেষণায় অন্তর্ভুক্ত হলে গবেষণায় নিয়ম নীতি ভংগ হওয়ার সম্ভাবনা আছে কিনা	☐	☐

অন্তর্ভুক্তি

রোগীটি গবেষণার অন্তর্ভুক্ত হওয়ার যোগ্য কিনা ? ☐ না ☐ হ্যাঁ

যদি হ্যাঁ হয় :

রেনডোমাইজেশন এর তারিখ : ☐☐ ☐☐☐ ☐☐ রেনডোমাইজেশন এর সময় : ☐☐ ___ **CRF 8**

দিন মাস বছর ঘন্টা মিনিট

রেনডোমাইজেশন এর নাম্বার: ☐☐☐

গবেষণার ১ম দিন

**পরিদর্শনের তারিখ**<br>(দিন মাস বছর)	**পরিদর্শনের সময়**<br>(ঘন্টা : মিনিট)
☐☐ ☐☐☐ ☐☐	☐☐ : ☐☐

গবেষণার জিনিসপত্র/ ঔষধ দেওয়ার সূচী

ডোজ	**তারিখ**<br>(দিন মাস বছর)	**সময়**<br>(ঘন্টা : মিনিট)	দেওয়ার পরিমান (মিলি)	দেওয়ার বাকী আছে (মিলি)
১	☐☐ ☐☐☐ ☐☐	☐☐ : ☐☐	☐☐	☐☐

Study ID No.ঃ | | | |

K	W	M	C	H

গবেষণার জিনিসপত্র/ ঔষধ দেওয়ার সূচী

ডোজ	**তারিখ** (দিন মাস বছর)	**সময়** (ঘন্টা : মিনিট)	দেওয়ার পরিমান (মিলি)	দেওয়ার বাকী আছে (মিলি)
২	☐☐ ☐☐☐ ☐☐	☐☐ : ☐☐	☐☐	☐☐

অত্যাবশ্যকীয় লক্ষণ

**Variables** সময় গণনা	**নাড়ীর গতি** (প্রতি মিনিটে)	**শ্বাসের গতি** (প্রতি মিনিট)	**তাপমাত্রা (বগলের)** (০সে.)	**ব্লাড প্রেসার. Systolic** (mmHg) [শুধু মাত্র সকালে]	**ব্লাড প্রেসার. Diastolic** (mmHg) [শুধু মাত্র সকালে]
যদি কোন reading স্বাভাবিক পরিসীমা অতিক্রম করে?	☐ না<br>☐ হ্যাঁ	☐ না<br>☐ হ্যাঁ	☐ না<br>☐ হ্যাঁ	☐ না<br>☐ হ্যাঁ	☐ না<br>☐ হ্যাঁ
যদি হ্যাঁ হয় তবে, reading clinically Significant কিনা?*	☐ না<br>☐ হ্যাঁ	☐ না<br>☐ হ্যাঁ	☐ না<br>☐ হ্যাঁ	☐ না<br>☐ হ্যাঁ	☐ না<br>☐ হ্যাঁ

**যদি reading Clinically Significant হয় তবে প্রয়োজন হলে অনুগ্রহ করে Adverse Event Form পূরণ করুন]*

CRF 9

অন্যান্য লক্ষন সমূহ

বর্ণনা	না	হ্যাঁ
পেটে ব্যাথা আছে কিনা?	☐	☐
পেটে ফাঁপা আছে কিনা?	☐	☐
জ্বর আছে কিনা?	☐	☐
শারিরীক দুর্বলতা আছে কিনা?	☐	☐
বমি আছে কিনা?<br>যদি হ্যাঁ হয়, প্রতি দিন কত বার বমি হয় ? ☐☐	☐	☐
অন্যান্য অস্বাভাবিকতা উপস্থিতি যদি হ্যাঁ হয়, নির্দিষ্ট করুন____________________	☐	☐
পায়খানার সাথে মলের *উপস্থিতির ইতিহাস*	☐	☐
	☐	☐
	☐	☐

Study ID No.ঃ | | | |

K	W	M	C	H

পায়খানার সাথে রক্তের *উপস্থিতির ইতিহাস*		
চাল ধোয়া পানির মত পায়খানার ইতিহাস		

শিশুটি বুকের দুধ খায় কিনা না ☐ হ্যাঁ ☐ **যদি হ্যাঁ হয়, প্রতিদিন কত বার খায় ?** ☐☐

শিশুটি তৈরি করা দুধ খায় কিনা না ☐ হ্যাঁ ☐ **যদি হ্যাঁ হয়, দিনে কত পরিমান (মি.লি)** ☐☐☐☐

সামগ্রিকভাবে খাবার এবং দুধ গ্রহনের পরিমান *(দিন শেষ হওয়ার ১ ঘন্টা পূর্বে)*

শশিুর খাবার / দুধ খাওয়ার অবস্থা নচিে উল্লখেতি স্কোর অনুযায়ী<br>(১=স্বাভাবিক, ২=কিছুটা কম, ৩=মধ্যম কম, ৪=খুব বেশি কম, ৫=খাবার গ্রহনে অস্বীকৃতি)	☐

পানি শূন্যতার পরিমান *(দিন শেষ হওয়ার ১ ঘন্টা পূর্বে)*

নিচে উল্লেখিত তালিকা অনুযায়ী শিশুর পানি শূন্যতা :<br>(০=পানি শূন্যতা নাই ১=কিছুটা পানি শূন্যতা ২= মারাত্বক পানি শূন্যতা *)<br>* যদি মারাত্বক পানি শূন্যতা হয় তাহলে শিশুকে গবেষণা থেকে বাদ দিন	☐

প্রতিকূল ঘটনায় প্রযোজ্য ঔষধ সমূহ

গত পরিদর্শনের সময় দেওয়া ঔষধ সমূহ/ **প্রতিকূল ঘটনার** পরিবর্তন হয়েছে কিনা?<br>*যদি হ্যাঁ হয় তবে প্রযোজ্য লিষ্ট থেকে নতুন ঔষধ যোগ করুন*	☐ [illegible]	☐ [illegible]

CRF 10

পানীয় (ফাইবারসল-২/প্লাসিবু) গ্রহনের পর শিশুর প্রতিক্রিয়া :
১ম দিনের ১ম ডোজ
৩০ মিনিটে ফাইবারসল-২/প্লাসিবু গ্রহনের পরিমান : ☐ . ☐ গ্রাম

বর্ণনা	না	হ্যাঁ
পেট ফুলে গিয়েছে কিনা?	☐	☐
পেটে ব্যাথা আছে কিনা?	☐	☐
পেট ডাকে কিনা?	☐	☐
পেটে ফাঁপা আছে কিনা?	☐	☐
শিশুটি খাবার থেকে মুখ ঘুরিয়ে নেয় কিনা ?	☐	☐
	☐	☐

Study ID No.ঃ | | | |

K	W	M	C	H

শিশুটি মুখ বন্ধ করে রাখে কিনা ?		
শিশুটি মাড়ি কামড়ে রাখে কিনা ?	☐	☐
শিশুটি বিরক্ত হয়েছে কিনা ?	☐	☐
শিশুটি মুখ থেকে **ফাইবারসল-২/প্লাসিবু** ফেলে দেয় কিনা ?	☐	☐
শিশুটি **ফাইবারসল-২/প্লাসিবু** গিলতে অস্বীকার করে কিনা ?	☐	☐

১ম দিনের ২য় ডোজ

৩০ মিনিটে ফাইবারসল-২/প্লাসিবু গ্রহনের পরিমান : ☐ . ☐ গ্রাম

বর্ণনা	না	হ্যাঁ
পেট ফুলে গিয়েছে কিনা?	☐	☐
পেটে ব্যাথা আছে কিনা?	☐	☐
পেট ডাকে কিনা?	☐	☐
পেটে ফাঁপা আছে কিনা?	☐	☐
শিশুটি খাবার থেকে মুখ ঘুরিয়ে নেয় কিনা ?	☐	☐
শিশুটি মুখ বন্ধ করে রাখে কিনা ?	☐	☐
শিশুটি মাড়ি কামড়ে রাখে কিনা ?	☐	**CRF 11**
শিশুটি বিরক্ত হয়েছে কিনা ?	☐	☐
শিশুটি মুখ থেকে **ফাইবারসল-২/প্লাসিবু** ফেলে দেয় কিনা ?	☐	☐
শিশুটি **ফাইবারসল-২/প্লাসিবু** গিলতে অস্বীকার করে কিনা ?	☐	☐

বমি আছে কিনা?<br>যদি হ্যাঁ হয়, প্রতি দিন কত বার বমি হয় ? ☐☐ বার<br>প্রতিদিন কি পরিমান বমি করেছে ☐☐☐ গ্রাম	না ☐ হ্যাঁ ☐
মায়ের দৃষ্টিতে শিশুটি **ফাইবারসল-২/প্লাসিবু পছন্দ করেছে কিনা ?**	☐

*** শিশুটি পানীয় নিতে অস্বীকার করেছে বলে বিবেচনা করবো যদি সে পানীয় থেকে মুখ ফিরিয়ে নেয়, কান্নাকাটি করে ,মুখ জোর করে বন্ধ করে রাখে অথবা দাত খিচিয়ে রাখে অথবা বিরক্ত হয়ে যায়, মুখ থেকে ফেলে দেয় কিংবা গিলতে অস্বীকার করে। আগে থেকে ওজন নেয়া নেপকিন সরবরাহ করা হবে। কোনো খাবার যদি উগড়ে ফেলে বা বমি করে ফেলে দেয়া হয়, তা মুছে নেপকিনের ওজন নেয়া হবে এবং সরবরাহকৃত পরিমান থেকে বাদ দেয়া হবে। ৭ পয়েন্টের হেডোনিক স্কেল ব্যবহার করা হবে যেখানে প্রতিটি পয়েন্ট মুখভঙ্গির অঙ্কন দিয়ে নির্ধারন করা হবে। আমরা মায়েদের থেকে খাবারের রং,গন্ধ, স্বাদ এবং সার্বিক গ্রহনযোগ্যতা সর্ম্পকে মতামত চেয়েছি। (১=খুবই অপছন্দ, ২=কিছুটা অপছন্দ, ৩= অপছন্দ, ৪= পছন্দ-অপছন্দ কিছুই না, ৫=কিছুটা পছন্দ, ৬=মোটামুটি পছন্দ, ৭= খুবই পছন্দ)।

পায়খানার ধরণ :	☐

Study ID No.ঃ | | | |

K	W	M	C	H

* **পায়খানার ধরণ** *(১= শক্ত, ২= নরম, ৩ = পাতলা / পানির মত, ৪ = পাতলা পায়খানার সাথে রক্ত)*

CRF 12

গবেষণার ২য় দিন

পরিদর্শনের তারিখ (দিন মাস বছর)	পরিদর্শনের সময় (ঘন্টা : মিনিট)
	:

গবেষণার জিনিসপত্র/ ঔষধ দেওয়ার সূচী

ডোজ	তারিখ (দিন মাস বছর)	সময় (ঘন্টা : মিনিট)	দেওয়ার পরিমান (মিলি)	দেওয়ার বাকী আছে (মিলি)
১		:		

Study ID No.ঃ | | | |

K	W	M	C	H

গবেষণার জিনিসপত্র/ ঔষধ দেওয়ার সূচী

ডোজ	তারিখ (দিন মাস বছর)	সময় (ঘন্টা : মিনিট)	দেওয়ার পরিমান (মিলি)	দেওয়ার বাকী আছে (মিলি)
২	☐☐ ☐☐☐ ☐☐	☐☐ : ☐☐	☐☐	☐☐

অত্যাবশ্যকীয় লক্ষণ

**Variables** সময় গণনা	**নাড়ীর গতি** (প্রতি মিনিটে)	**শ্বাসের গতি** (প্রতি মিনিট)	**তাপমাত্রা (বগলের)** ($^{0সে.}$)	**ব্লাড প্রেসার. Systolic** (mmHg) [শুধু মাত্র সকালে]	**ব্লাড প্রেসার. Diastolic** (mmHg) [শুধু মাত্র সকালে]
যদি কোন reading স্বাভাবিক পরিসীমা অতিক্রম করে?	☐ না ☐ হ্যাঁ	☐ না ☐ হ্যাঁ	☐ না ☐ হ্যাঁ	☐ না ☐ হ্যাঁ	☐ না ☐ হ্যাঁ
যদি হ্যাঁ হয় তবে, reading clinically Significant কিনা?*	☐ না ☐ হ্যাঁ	☐ না ☐ হ্যাঁ	☐ না ☐ হ্যাঁ	☐ না ☐ হ্যাঁ	☐ না ☐ হ্যাঁ

**যদি reading Clinically Significant হয় তবে প্রয়োজন হলে অনুগ্রহ করে Adverse Event Form পূরণ করুন]*

CRF 13

অন্যান্য লক্ষন সমূহ

বর্ণনা	না	হ্যাঁ
পেটে ব্যাথা আছে কিনা?	☐	☐
পেটে ফাঁপা আছে কিনা?	☐	☐
জ্বর আছে কিনা?	☐	☐
শারিরীক দুর্বলতা আছে কিনা?	☐	☐
বমি আছে কিনা? যদি হ্যাঁ হয়, প্রতি দিন কত বার বমি হয় ? ☐☐	☐	☐
অন্যান্য অস্বাভাবিকতা উপস্থিতি যদি হ্যাঁ হয়, নির্দিষ্ট করুন________________________________	☐	☐
পায়খানার সাথে মলের *উপস্থিতির ইতিহাস*	☐	☐
	☐	☐
	☐	☐

Study ID No.ঃ | | | |

K	W	M	C	H

পায়খানার সাথে রক্তের *উপস্থিতির ইতিহাস*		
চাল ধোয়া পানির মত পায়খানার ইতিহাস		

শিশুটি বুকের দুধ খায় কিনা না ☐ হ্যাঁ ☐ **যদি হ্যাঁ হয়, প্রতিদিন কত বার খায় ?** ☐☐

শিশুটি তৈরি করা দুধ খায় কিনা না ☐ হ্যাঁ ☐ **যদি হ্যাঁ হয়, দিনে কত পরিমান (মি.লি)** ☐☐☐☐

সামগ্রিকভাবে খাবার এবং দুধ গ্রহনের পরিমান *(দিন শেষ হওয়ার ১ ঘন্টা পূর্বে)*

শশিুর খাবার / দুধ খাওয়ার অবস্থা নচিে উল্লখেতি স্কোর অনুযায়ী<br>(১=স্বাভাবিক, ২=কিছুটা কম, ৩=মধ্যম কম, ৪=খুব বেশি কম, ৫=খাবার গ্রহনে অস্বীকৃতি)	☐

পানি শূন্যতার পরিমান *(দিন শেষ হওয়ার ১ ঘন্টা পূর্বে)*

নিচে উল্লেখিত তালিকা অনুযায়ী শিশুর পানি শূন্যতা :<br>(০=পানি শূন্যতা নাই ১=কিছুটা পানি শূন্যতা ২= মারাত্বক পানি শূন্যতা *)<br>* যদি মারাত্বক পানি শূন্যতা হয় তাহলে শিশুকে গবেষণা থেকে বাদ দিন	☐

প্রতিকূল ঘটনায় প্রযোজ্য ঔষধ সমূহ

গত পরিদর্শনের সময় দেওয়া ঔষধ সমূহ/ **প্রতিকূল ঘটনার** পরিবর্তন হয়েছে কিনা?<br>*যদি হ্যাঁ হয় তবে প্রযোজ্য লিষ্ট থেকে নতুন ঔষধ যোগ করুন*	☐ **CRF 14**

পানীয় (ফাইবারসল-২/প্লাসিবু) গ্রহনের পর শিশুর প্রতিক্রিয়া :

২য় দিনের ১ম ডোজ

৩০ মিনিটে ফাইবারসল-২/প্লাসিবু গ্রহনের পরিমান : ☐ . ☐ গ্রাম

বর্ণনা	না	হ্যাঁ
পেট ফুলে গিয়েছে কিনা?	☐	☐
পেটে ব্যাথা আছে কিনা?	☐	☐
পেট ডাকে কিনা?	☐	☐
পেটে ফাঁপা আছে কিনা?	☐	☐
শিশুটি খাবার থেকে মুখ ঘুরিয়ে নেয় কিনা ?	☐	☐
	☐	☐

Study ID No.ঃ | | | |

K	W	M	C	H

শিশুটি মুখ বন্ধ করে রাখে কিনা ?		
শিশুটি মাড়ি কামড়ে রাখে কিনা ?	☐	☐
শিশুটি বিরক্ত হয়েছে কিনা ?	☐	☐
শিশুটি মুখ থেকে **ফাইবারসল-২/প্লাসিবু** ফেলে দেয় কিনা ?	☐	☐
শিশুটি **ফাইবারসল-২/প্লাসিবু** গিলতে অস্বীকার করে কিনা ?	☐	☐

২য় দিনের ২য় ডোজ

৩০ মিনিটে ফাইবারসল-২/প্লাসিবু গ্রহনের পরিমান : ☐ . ☐ গ্রাম

বর্ণনা	না	হ্যাঁ
পেট ফুলে গিয়েছে কিনা?	☐	☐
পেটে ব্যাথা আছে কিনা?	☐	☐
পেট ডাকে কিনা?	☐	☐
পেটে ফাঁপা আছে কিনা?	☐	☐
শিশুটি খাবার থেকে মুখ ঘুরিয়ে নেয় কিনা ?	☐	☐
শিশুটি মুখ বন্ধ করে রাখে কিনা ?	☐	☐
শিশুটি মাড়ি কামড়ে রাখে কিনা ?	☐	☐
শিশুটি বিরক্ত হয়েছে কিনা ?	☐	☐
শিশুটি মুখ থেকে **ফাইবারসল-২/প্লাসিবু** ফেলে দেয় কিনা ?	☐	☐
শিশুটি **ফাইবারসল-২/প্লাসিবু** গিলতে অস্বীকার করে কিনা ?	☐	☐

CRF 15

বমি আছে কিনা?<br>যদি হ্যাঁ হয়, প্রতি দিন কত বার বমি হয় ? ☐☐ বার<br>প্রতিদিন কি পরিমান বমি করেছে ☐☐☐ গ্রাম	না ☐ হ্যাঁ ☐
মায়ের দৃষ্টিতে শিশুটি **ফাইবারসল-২/প্লাসিবু পছন্দ করেছে কিনা ?**	☐

*** শিশুটি পানীয় নিতে অস্বীকার করেছে বলে বিবেচনা করবো যদি সে পানীয় থেকে মুখ ফিরিয়ে নেয়, কান্নাকাটি করে ,মুখ জোর করে বন্ধ করে রাখে অথবা দাত খিচিয়ে রাখে অথবা বিরক্ত হয়ে যায়, মুখ থেকে ফেলে দেয় কিংবা গিলতে অস্বীকার করে। আগে থেকে ওজন নেয়া নেপকিন সরবরাহ করা হবে। কোনো খাবার যদি উগড়ে ফেলে বা বমি করে ফেলে দেয়া হয়, তা মুছে নেপকিনের ওজন নেয়া হবে এবং সরবরাহকৃত পরিমান থেকে বাদ দেয়া হবে। ৭ পয়েন্টের হেডোনিক স্কেল ব্যবহার করা হবে যেখানে প্রতিটি পয়েন্ট মুখভঙ্গির অঙ্কন দিয়ে নির্ধারন করা হবে। আমরা মায়েদের থেকে খাবারের রং,গন্ধ, স্বাদ এবং সার্বিক গ্রহনযোগ্যতা সম্পর্কে মতামত চেয়েছি। (১=খুবই অপছন্দ, ২=কিছুটা অপছন্দ, ৩= অপছন্দ, ৪= পছন্দ-অপছন্দ কিছুই না, ৫=কিছুটা পছন্দ, ৬=মোটামুটি পছন্দ, ৭= খুবই পছন্দ)।

Study ID No.ঃ | | | |

K	W	M	C	H

পায়খানার ধরণ :	☐

* **পায়খানার ধরণ** *(১= শক্ত, ২= নরম, ৩ = পাতলা / পানির মত, ৪ = পাতলা পায়খানার সাথে রক্ত)*

CRF 16

গবেষণার ৩য় দিন

**পরিদর্শনের তারিখ** (দিন মাস বছর)	**পরিদর্শনের সময়** (ঘন্টা : মিনিট)
	:

গবেষণার জিনিসপত্র/ ঔষধ দেওয়ার সূচী

ডোজ	**তারিখ** (দিন মাস বছর)	**সময়** (ঘন্টা : মিনিট)	দেওয়ার পরিমান (মিলি)	দেওয়ার বাকী আছে (মিলি)
১		:		

Study ID No.ঃ | | | |

K	W	M	C	H

গবেষণার জিনিসপত্র/ ঔষধ দেওয়ার সূচী

ডোজ	তারিখ (দিন মাস বছর)	সময় (ঘন্টা : মিনিট)	দেওয়ার পরিমান (মিলি)	দেওয়ার বাকী আছে (মিলি)
২		:		

অত্যাবশ্যকীয় লক্ষণ

**Variables** সময় গণনা	নাড়ীর গতি (প্রতি মিনিটে)	শ্বাসের গতি (প্রতি মিনিট)	তাপমাত্রা (বগলের) (^{0}সে.)	ব্লাড প্রেসার. Systolic (mmHg) [শুধু মাত্র সকালে]	ব্লাড প্রেসার. **Diastolic** (mmHg) [শুধু মাত্র সকালে]
যদি কোন reading স্বাভাবিক পরিসীমা অতিক্রম করে?	☐ না ☐ হ্যাঁ	☐ না ☐ হ্যাঁ	☐ না ☐ হ্যাঁ	☐ না ☐ হ্যাঁ	☐ না ☐ হ্যাঁ
যদি হ্যাঁ হয় তবে, reading clinically Significant কিনা?*	☐ না ☐ হ্যাঁ	☐ না ☐ হ্যাঁ	☐ না ☐ হ্যাঁ	☐ না ☐ হ্যাঁ	☐ না ☐ হ্যাঁ

**যদি reading Clinically Significant হয় তবে প্রয়োজন হলে অনুগ্রহ করে Adverse Event Form পূরণ করুন]*

CRF 17

অন্যান্য লক্ষন সমূহ

বর্ণনা	না	হ্যাঁ
পেটে ব্যাথা আছে কিনা?	☐	☐
পেটে ফাঁপা আছে কিনা?	☐	☐
জ্বর আছে কিনা?	☐	☐
শারিরীক দুর্বলতা আছে কিনা?	☐	☐
বমি আছে কিনা? যদি হ্যাঁ হয়, প্রতি দিন কত বার বমি হয় ? ☐☐	☐	☐
অন্যান্য অস্বাভাবিকতা উপস্থিতি যদি হ্যাঁ হয়, নির্দিষ্ট করুন________________	☐	☐
পায়খানার সাথে মলের *উপস্থিতির ইতিহাস*	☐	☐
	☐	☐
	☐	☐

Study ID No.ঃ | | | |

K	W	M	C	H

পায়খানার সাথে রক্তের *উপস্থিতির ইতিহাস*		
চাল ধোয়া পানির মত পায়খানার ইতিহাস		

শিশুটি বুকের দুধ খায় কিনা না ☐ হ্যাঁ ☐ **যদি হ্যাঁ হয়, প্রতিদিন কত বার খায় ?** ☐☐

শিশুটি তৈরি করা দুধ খায় কিনা না ☐ হ্যাঁ ☐ **যদি হ্যাঁ হয়, দিনে কত পরিমান (মি.লি)** ☐☐☐☐

সামগ্রিকভাবে খাবার এবং দুধ গ্রহনের পরিমান *(দিন শেষ হওয়ার ১ ঘন্টা পূর্বে)*

শশিুর খাবার / দুধ খাওয়ার অবস্থা নচিে উল্লখেতি স্কেোর অনুযায়ী<br>(১=স্বাভাবিক, ২=কিছুটা কম, ৩=মধ্যম কম, ৪=খুব বেশি কম, ৫=খাবার গ্রহনে অস্বীকৃতি)	☐

পানি শূন্যতার পরিমান *(দিন শেষ হওয়ার ১ ঘন্টা পূর্বে)*

নিচে উল্লেখিত তালিকা অনুযায়ী শিশুর পানি শূন্যতা :<br>(০=পানি শূন্যতা নাই ১=কিছুটা পানি শূন্যতা ২= মারাত্বক পানি শূন্যতা *)<br>* যদি মারাত্বক পানি শূন্যতা হয় তাহলে শিশুকে গবেষণা থেকে বাদ দিন	☐

প্রতিকূল ঘটনায় প্রযোজ্য ঔষধ সমূহ

গত পরিদর্শনের সময় দেওয়া ঔষধ সমূহ/ **প্রতিকূল ঘটনার** পরিবর্তন হয়েছে কিনা?<br>*যদি হ্যাঁ হয় তবে প্রযোজ্য লিষ্ট থেকে নতুন ঔষধ যোগ করুন*	☐ **CRF 18**

পানীয় (ফাইবারসল-২/প্লাসিবু) গ্রহনের পর শিশুর প্রতিক্রিয়া :
৩য় দিনের ১ম ডোজ
৩০ মিনিটে ফাইবারসল-২/প্লাসিবু গ্রহনের পরিমান : ☐ . ☐ গ্রাম

বর্ণনা	না	হ্যাঁ
পেট ফুলে গিয়েছে কিনা?	☐	☐
পেটে ব্যাথা আছে কিনা?	☐	☐
পেট ডাকে কিনা?	☐	☐
পেটে ফাঁপা আছে কিনা?	☐	☐
শিশুটি খাবার থেকে মুখ ঘুরিয়ে নেয় কিনা ?	☐	☐
	☐	☐

Study ID No.ঃ | | | |

K	W	M	C	H

শিশুটি মুখ বন্ধ করে রাখে কিনা ?		
শিশুটি মাড়ি কামড়ে রাখে কিনা ?	☐	☐
শিশুটি বিরক্ত হয়েছে কিনা ?	☐	☐
শিশুটি মুখ থেকে **ফাইবারসল-২/প্লাসিবু** ফেলে দেয় কিনা ?	☐	☐
শিশুটি **ফাইবারসল-২/প্লাসিবু** গিলতে অস্বীকার করে কিনা ?	☐	☐

৩য় দিনের ২য় ডোজ
৩০ মিনিটে ফাইবারসল-২/প্লাসিবু গ্রহনের পরিমান : ☐ . ☐ গ্রাম

বর্ণনা	না	হ্যাঁ
পেট ফুলে গিয়েছে কিনা?	☐	☐
পেটে ব্যাথা আছে কিনা?	☐	☐
পেট ডাকে কিনা?	☐	☐
পেটে ফাঁপা আছে কিনা?	☐	☐
শিশুটি খাবার থেকে মুখ ঘুরিয়ে নেয় কিনা ?	☐	☐
শিশুটি মুখ বন্ধ করে রাখে কিনা ?	☐	☐
শিশুটি মাড়ি কামড়ে রাখে কিনা ?	☐	☐
শিশুটি বিরক্ত হয়েছে কিনা ?	☐	☐
শিশুটি মুখ থেকে **ফাইবারসল-২/প্লাসিবু** ফেলে দেয় কিনা ?	☐	☐
শিশুটি **ফাইবারসল-২/প্লাসিবু** গিলতে অস্বীকার করে কিনা ?	☐	☐

CRF 19

বমি আছে কিনা?<br>যদি হ্যাঁ হয়, প্রতি দিন কত বার বমি হয় ? ☐☐ বার<br>প্রতিদিন কি পরিমান বমি করেছে ☐☐☐ গ্রাম	না ☐ হ্যাঁ ☐
মায়ের দৃষ্টিতে শিশুটি **ফাইবারসল-২/প্লাসিবু পছন্দ করেছে কিনা ?**	☐

*** শিশুটি পানীয় নিতে অস্বীকার করেছে বলে বিবেচনা করবো যদি সে পানীয় থেকে মুখ ফিরিয়ে নেয়, কান্নাকাটি করে ,মুখ জোর করে বন্ধ করে রাখে অথবা দাত খিচিয়ে রাখে অথবা বিরক্ত হয়ে যায়, মুখ থেকে ফেলে দেয় কিংবা গিলতে অস্বীকার করে। আগে থেকে ওজন নেয়া নেপকিন সরবরাহ করা হবে। কোনো খাবার যদি উগড়ে ফেলে বা বমি করে ফেলে দেয়া হয়, তা মুছে নেপকিনের ওজন নেয়া হবে এবং সরবরাহকৃত পরিমান থেকে বাদ দেয়া হবে। ৭ পয়েন্টের হেডোনিক স্কেল ব্যবহার করা হবে যেখানে প্রতিটি পয়েন্ট মুখভঙ্গির অঙ্কন দিয়ে নির্ধারন করা হবে। আমরা মায়েদের থেকে খাবারের রং,গন্ধ, স্বাদ এবং সার্বিক গ্রহনযোগ্যতা সর্ম্পকে মতামত চেয়েছি। (১=খুবই অপছন্দ, ২=কিছুটা অপছন্দ, ৩= অপছন্দ, ৪= পছন্দ-অপছন্দ কিছুই না, ৫=কিছুটা পছন্দ, ৬=মোটামুটি পছন্দ, ৭= খুবই পছন্দ)।

Study ID No.ঃ | | | |

K	W	M	C	H

পায়খানার ধরণ :	☐

* **পায়খানার ধরণ** *(১= শক্ত, ২= নরম, ৩ = পাতলা / পানির মত, ৪ = পাতলা পায়খানার সাথে রক্ত)*

CRF 20

গবেষণার ৪র্থ দিন

পরিদর্শনের তারিখ (দিন মাস বছর)	পরিদর্শনের সময় (ঘন্টা : মিনিট)
☐☐ ☐☐☐ ☐☐	☐☐ : ☐☐

গবেষণার জিনিসপত্র/ ঔষধ দেওয়ার সূচী

ডোজ	তারিখ (দিন মাস বছর)	সময় (ঘন্টা : মিনিট)	দেওয়ার পরিমান (মিলি)	দেওয়ার বাকী আছে (মিলি)

Study ID No.ঃ | | | |

K	W	M	C	H

১		:			

গবেষণার জিনিসপত্র/ ঔষধ দেওয়ার সূচী

ডোজ	তারিখ (দিন মাস বছর)	সময় (ঘন্টা : মিনিট)	দেওয়ার পরিমান (মিলি)	দেওয়ার বাকী আছে (মিলি)
২		:		

অত্যাবশ্যকীয় লক্ষণ

**Variables** সময় গণনা	নাড়ীর গতি (প্রতি মিনিটে)	শ্বাসের গতি (প্রতি মিনিট)	তাপমাত্রা (বগলের) ($^{0সে.}$)	ব্লাড প্রেসার. Systolic (mmHg) [শুধু মাত্র সকালে]	ব্লাড প্রেসার. **Diastolic** (mmHg) [শুধু মাত্র সকালে]
যদি কোন reading স্বাভাবিক পরিসীমা অতিক্রম করে?	☐ না ☐ হ্যাঁ	☐ না ☐ হ্যাঁ	☐ না ☐ হ্যাঁ	☐ না ☐ হ্যাঁ	☐ না ☐ হ্যাঁ
যদি হ্যাঁ হয় তবে, reading clinically Significant কিনা?*	☐ না ☐ হ্যাঁ	☐ না ☐ হ্যাঁ	☐ না ☐ হ্যাঁ	☐ না ☐ হ্যাঁ	☐ না ☐ হ্যাঁ

**যদি reading Clinically Significant হয় তবে প্রয়োজন হলে অনুগ্রহ করে Adverse Event Form পূরণ করুন]*

CRF 21

অন্যান্য লক্ষন সমূহ

বর্ণনা	না	হ্যাঁ
পেটে ব্যাথা আছে কিনা?	☐	☐
পেটে ফাঁপা আছে কিনা?	☐	☐
জ্বর আছে কিনা?	☐	☐
শারিরীক দুর্বলতা আছে কিনা?	☐	☐
বমি আছে কিনা? যদি হ্যাঁ হয়, প্রতি দিন কত বার বমি হয় ? ☐☐	☐	☐

Study ID No.ঃ ☐☐☐

K	W	M	C	H

অন্যান্য অস্বাভাবিকতা উপস্থিতি যদি হ্যাঁ হয়, নির্দিষ্ট করুন________________________________	☐	☐
পায়খানার সাথে মলের *উপস্থিতির ইতিহাস*		☐
পায়খানার সাথে রক্তের *উপস্থিতির ইতিহাস*		
চাল ধোয়া পানির মত পায়খানার ইতিহাস		

শিশুটি বুকের দুধ খায় কিনা না ☐ হ্যাঁ ☐ **যদি হ্যাঁ হয়, প্রতিদিন কত বার খায় ?** ☐☐

শিশুটি তৈরি করা দুধ খায় কিনা না ☐ হ্যাঁ ☐ **যদি হ্যাঁ হয়, দিনে কত পরিমান (মি.লি)** ☐☐☐☐

সামগ্রিকভাবে খাবার এবং দুধ গ্রহনের পরিমান *(দিন শেষ হওয়ার ১ ঘন্টা পূর্বে)*

শশিুর খাবার / দুধ খাওয়ার অবস্থা নচিে উল্লখেতি স্কোর অনুযায়ী<br>(১=স্বাভাবিক, ২=কিছুটা কম, ৩=মধ্যম কম, ৪=খুব বেশি কম, ৫=খাবার গ্রহনে অস্বীকৃতি)	☐

পানি শূন্যতার পরিমান *(দিন শেষ হওয়ার ১ ঘন্টা পূর্বে)*

নিচে উল্লেখিত তালিকা অনুযায়ী শিশুর পানি শূন্যতা :<br>(০=পানি শূন্যতা নাই ১=কিছুটা পানি শূন্যতা ২= মারাত্মক পানি শূন্যতা *)<br>* যদি মারাত্মক পানি শূন্যতা হয় তাহলে শিশুকে গবেষণা থেকে বাদ দিন	☐

প্রতিকূল ঘটনায় প্রযোজ্য ঔষধ সমূহ

গত পরিদর্শনের সময় দেওয়া ঔষধ সমূহ/ **প্রতিকূল ঘটনার** পরিবর্তন হয়েছে কিনা?<br>*যদি হ্যাঁ হয় তবে প্রযোজ্য লিষ্ট থেকে নতুন ঔষধ যোগ করুন*	**CRF 22**

পানীয় (ফাইবারসল-২/প্লাসিবু) গ্রহনের পর শিশুর প্রতিক্রিয়া :
৪র্থ দিনের ১ম ডোজ
৩০ মিনিটে ফাইবারসল-২/প্লাসিবু গ্রহনের পরিমান : ☐ . ☐ গ্রাম

বর্ণনা	না	হ্যাঁ
পেট ফুলে গিয়েছে কিনা?	☐	☐
পেটে ব্যাথা আছে কিনা?	☐	☐
পেট ডাকে কিনা?	☐	☐
পেটে ফাঁপা আছে কিনা?	☐	☐

Study ID No.ঃ | | | |

K	W	M	C	H

শিশুটি খাবার থেকে মুখ ঘুরিয়ে নেয় কিনা ?	☐	☐
শিশুটি মুখ বন্ধ করে রাখে কিনা ?	☐	☐
শিশুটি মাড়ি কামড়ে রাখে কিনা ?	☐	☐
শিশুটি বিরক্ত হয়েছে কিনা ?	☐	☐
শিশুটি মুখ থেকে **ফাইবারসল-২/প্লাসিবু** ফেলে দেয় কিনা ?	☐	☐
শিশুটি **ফাইবারসল-২/প্লাসিবু** গিলতে অস্বীকার করে কিনা ?	☐	☐

৪র্থ দিনের ২য় ডোজ

৩০ মিনিটে ফাইবারসল-২/প্লাসিবু গ্রহনের পরিমান : ☐ . ☐ গ্রাম

বর্ণনা	না	হ্যাঁ
পেট ফুলে গিয়েছে কিনা?	☐	☐
পেটে ব্যাথা আছে কিনা?	☐	☐
পেট ডাকে কিনা?	☐	☐
পেটে ফাঁপা আছে কিনা?	☐	☐
শিশুটি খাবার থেকে মুখ ঘুরিয়ে নেয় কিনা ?	☐	☐
শিশুটি মুখ বন্ধ করে রাখে কিনা ?	☐	☐
শিশুটি মাড়ি কামড়ে রাখে কিনা ?	☐	☐
শিশুটি বিরক্ত হয়েছে কিনা ?	☐	☐ C[illegible]F 23
শিশুটি মুখ থেকে **ফাইবারসল-২/প্লাসিবু** ফেলে দেয় কিনা ?	☐	☐
শিশুটি **ফাইবারসল-২/প্লাসিবু** গিলতে অস্বীকার করে কিনা ?	☐	☐

বমি আছে কিনা?<br>যদি হ্যাঁ হয়, প্রতি দিন কত বার বমি হয় ? ☐☐ বার<br>প্রতিদিন কি পরিমান বমি করেছে ☐☐☐ গ্রাম	না ☐ হ্যাঁ ☐
মায়ের দৃষ্টিতে শিশুটি **ফাইবারসল-২/প্লাসিবু পছন্দ করেছে কিনা ?**	☐

*** শিশুটি পানীয় নিতে অস্বীকার করেছে বলে বিবেচনা করবো যদি সে পানীয় থেকে মুখ ফিরিয়ে নেয়, কান্নাকাটি করে ,মুখ জোর করে বন্ধ করে রাখে অথবা দাত খিচিয়ে রাখে অথবা বিরক্ত হয়ে যায়, মুখ থেকে ফেলে দেয় কিংবা গিলতে অস্বীকার করে। আগে থেকে ওজন নেয়া নেপকিন সরবরাহ করা হবে। কোনো খাবার যদি উগড়ে ফেলে বা বমি করে ফেলে দেয়া হয়, তা মুছে নেপকিনের ওজন নেয়া হবে এবং সরবরাহকৃত পরিমান থেকে বাদ দেয়া হবে। ৭ পয়েন্টের হেডোনিক স্কেল ব্যবহার করা হবে যেখানে প্রতিটি পয়েন্ট মুখভঙ্গির অঙ্কন দিয়ে নির্ধারন করা হবে। আমরা মায়েদের থেকে খাবারের রং,গন্ধ, স্বাদ এবং সার্বিক গ্রহনযোগ্যতা

Study ID No.ঃ | | | |

**K**	**W**	**M**	**C**	**H**

সম্পর্কে মতামত চেয়েছি। (১=খুবই অপছন্দ, ২=কিছুটা অপছন্দ, ৩= অপছন্দ, ৪= পছন্দ-অপছন্দ কিছুই না, ৫=কিছুটা পছন্দ, ৬=মোটামুটি পছন্দ, ৭= খুবই পছন্দ)।

পায়খানার ধরণ :	☐

* **পায়খানার ধরণ** *(১= শক্ত, ২= নরম, ৩ = পাতলা / পানির মত, ৪ = পাতলা পায়খানার সাথে রক্ত)*

CRF 24

গবেষণার ৫ম দিন

পরিদর্শনের তারিখ (দিন মাস বছর)	পরিদর্শনের সময় (ঘন্টা : মিনিট)
☐☐ ☐☐☐ ☐☐	☐☐ : ☐☐

গবেষণার জিনিসপত্র/ ঔষধ দেওয়ার সূচী

Study ID No.ঃ | | | |

K	W	M	C	H

ডোজ	তারিখ (দিন মাস বছর)	সময় (ঘন্টা : মিনিট)	দেওয়ার পরিমান (মিলি)	দেওয়ার বাকী আছে (মিলি)
১		:		

গবেষণার জিনিসপত্র/ ঔষধ দেওয়ার সূচী

ডোজ	তারিখ (দিন মাস বছর)	সময় (ঘন্টা : মিনিট)	দেওয়ার পরিমান (মিলি)	দেওয়ার বাকী আছে (মিলি)
২		:		

অত্যাবশ্যকীয় লক্ষণ

**Variables** সময় গণনা	নাড়ীর গতি (প্রতি মিনিটে)	শ্বাসের গতি(প্রতি মিনিট)	তাপমাত্রা (বগলের) (০সে.)	ব্লাড প্রেসার. **Systolic** (mmHg) [শুধু মাত্র সকালে]	ব্লাড প্রেসার. **Diastolic** (mmHg) [শুধু মাত্র সকালে]
যদি কোন reading স্বাভাবিক পরিসীমা অতিক্রম করে?	☐ না ☐ হ্যাঁ	☐ না ☐ হ্যাঁ	☐ না ☐ হ্যাঁ	☐ না ☐ হ্যাঁ	☐ না ☐ হ্যাঁ
যদি হ্যাঁ হয় তবে, reading clinically Significant কিনা?*	☐ না ☐ হ্যাঁ	☐ না ☐ হ্যাঁ	☐ না ☐ হ্যাঁ	☐ না ☐ হ্যাঁ	☐ না ☐

CRF 25

**যদি reading Clinically Significant হয় তবে প্রয়োজন হলে অনুগ্রহ করে Adverse Event Form পূরণ করুন]*

অন্যান্য লক্ষন সমূহ

বর্ণনা	না	হ্যাঁ
পেটে ব্যাথা আছে কিনা?	☐	☐
পেটে ফাঁপা আছে কিনা?	☐	☐
জ্বর আছে কিনা?	☐	☐
শারিরীক দুর্বলতা আছে কিনা?	☐	☐
বমি আছে কিনা?	☐	☐

Study ID No.ঃ | | | |

K	W	M	C	H

যদি হ্যাঁ হয়, প্রতি দিন কত বার বমি হয় ?		
অন্যান্য অস্বাভাবিকতা উপস্থিতি যদি হ্যাঁ হয়, নির্দিষ্ট করুন________________________________	☐	☐
পায়খানার সাথে মলের *উপস্থিতির ইতিহাস*		☐
পায়খানার সাথে রক্তের *উপস্থিতির ইতিহাস*		
চাল ধোয়া পানির মত পায়খানার ইতিহাস		

শিশুটি বুকের দুধ খায় কিনা না ☐ হ্যাঁ ☐ **যদি হ্যাঁ হয়, প্রতিদিন কত বার খায় ?** ☐☐

শিশুটি তৈরি করা দুধ খায় কিনা না ☐ হ্যাঁ ☐ **যদি হ্যাঁ হয়, দিনে কত পরিমান (মি.লি)** ☐☐☐☐

সামগ্রিকভাবে খাবার এবং দুধ গ্রহনের পরিমান *(দিন শেষ হওয়ার ১ ঘন্টা পূর্বে)*

শশিুর খাবার / দুধ খাওয়ার অবস্থা নচিে উল্লখেতি স্কোর অনুযায়ী<br>(১=স্বাভাবিক, ২=কিছুটা কম, ৩=মধ্যম কম, ৪=খুব বেশি কম, ৫=খাবার গ্রহনে অস্বীকৃতি)	☐

পানি শূন্যতার পরিমান *(দিন শেষ হওয়ার ১ ঘন্টা পূর্বে)*

নিচে উল্লেখিত তালিকা অনুযায়ী শিশুর পানি শূন্যতা :<br>(০=পানি শূন্যতা নাই ১=কিছুটা পানি শূন্যতা ২= মারাত্বক পানি শূন্যতা *)<br>* যদি মারাত্বক পানি শূন্যতা হয় তাহলে শিশুকে গবেষণা থেকে বাদ দিন	☐

প্রতিকূল ঘটনায় প্রযোজ্য ঔষধ সমূহ

গত পরিদর্শনের সময় দেওয়া ঔষধ সমূহ/ **প্রতিকূল ঘটনার** পরিবর্তন হয়েছে কিনা?<br><br>*যদি হ্যাঁ হয় তবে প্রযোজ্য লিষ্ট থেকে নতুন ঔষধ যোগ করুন*	☐ না	☐ হ্যাঁ

CRF 26

পানীয় (ফাইবারসল-২/প্লাসিবু) গ্রহনের পর শিশুর প্রতিক্রিয়া :
৫ম দিনের ১ম ডোজ
৩০ মিনিটে ফাইবারসল-২/প্লাসিবু গ্রহনের পরিমান : ☐ . ☐ গ্রাম

বর্ণনা	না	হ্যাঁ
পেট ফুলে গিয়েছে কিনা?	☐	☐
পেটে ব্যাথা আছে কিনা?	☐	☐
পেট ডাকে কিনা?	☐	☐
	☐	☐

Study ID No.ঃ | | | |

K	W	M	C	H

পেটে ফাঁপা আছে কিনা?		
শিশুটি খাবার থেকে মুখ ঘুরিয়ে নেয় কিনা ?	☐	☐
শিশুটি মুখ বন্ধ করে রাখে কিনা ?	☐	☐
শিশুটি মাড়ি কামড়ে রাখে কিনা ?	☐	☐
শিশুটি বিরক্ত হয়েছে কিনা ?	☐	☐
শিশুটি মুখ থেকে **ফাইবারসল-২/প্লাসিবু** ফেলে দেয় কিনা ?	☐	☐
শিশুটি **ফাইবারসল-২/প্লাসিবু** গিলতে অস্বীকার করে কিনা ?	☐	☐

৫ম দিনের ২য় ডোজ

৩০ মিনিটে ফাইবারসল-২/প্লাসিবু গ্রহনের পরিমান : ☐ . ☐ গ্রাম

বর্ণনা	না	হ্যাঁ
পেট ফুলে গিয়েছে কিনা?	☐	☐
পেটে ব্যাথা আছে কিনা?	☐	☐
পেট ডাকে কিনা?	☐	☐
পেটে ফাঁপা আছে কিনা?	☐	☐
শিশুটি খাবার থেকে মুখ ঘুরিয়ে নেয় কিনা ?	☐	☐
শিশুটি মুখ বন্ধ করে রাখে কিনা ?	☐	☐
শিশুটি মাড়ি কামড়ে রাখে কিনা ?	☐	☐
শিশুটি বিরক্ত হয়েছে কিনা ?	☐	**CRF 27**
শিশুটি মুখ থেকে **ফাইবারসল-২/প্লাসিবু** ফেলে দেয় কিনা ?	☐	☐
শিশুটি **ফাইবারসল-২/প্লাসিবু** গিলতে অস্বীকার করে কিনা ?	☐	☐

বমি আছে কিনা?<br>যদি হ্যাঁ হয়, প্রতি দিন কত বার বমি হয় ? ☐☐ বার<br>প্রতিদিন কি পরিমান বমি করেছে ☐☐☐ গ্রাম	না ☐ হ্যাঁ ☐
মায়ের দৃষ্টিতে শিশুটি **ফাইবারসল-২/প্লাসিবু পছন্দ করেছে কিনা ?**	☐

*** শিশুটি পানীয় নিতে অস্বীকার করেছে বলে বিবেচনা করবো যদি সে পানীয় থেকে মুখ ফিরিয়ে নেয়, কান্নাকাটি করে ,মুখ জোর করে বন্ধ করে রাখে অথবা দাত খিচিয়ে রাখে অথবা বিরক্ত হয়ে যায়, মুখ থেকে ফেলে দেয় কিংবা গিলতে অস্বীকার করে। আগে থেকে ওজন নেয়া নেপকিন সরবরাহ করা হবে। কোনো খাবার যদি উগড়ে ফেলে বা বমি করে ফেলে দেয়া হয়, তা মুছে নেপকিনের ওজন নেয়া হবে এবং সরবরাহকৃত পরিমান থেকে বাদ দেয়া হবে। ৭ পয়েন্টের হেডোনিক

Study ID No.ঃ | | | |

K	W	M	C	H

স্কেল ব্যবহার করা হবে যেখানে প্রতিটি পয়েন্ট মুখভঙ্গির অঙ্কন দিয়ে নির্ধারন করা হবে। আমরা মায়েদের থেকে খাবারের রং,গন্ধ, স্বাদ এবং সার্বিক গ্রহনযোগ্যতা সম্পর্কে মতামত চেয়েছি। (১=খুবই অপছন্দ, ২=কিছুটা অপছন্দ, ৩= অপছন্দ, ৪= পছন্দ-অপছন্দ কিছুই না, ৫=কিছুটা পছন্দ, ৬=মোটামুটি পছন্দ, ৭= খুবই পছন্দ)।

পায়খানার ধরণ :	☐

* **পায়খানার ধরণ** *(১= শক্ত, ২= নরম, ৩ = পাতলা / পানির মত, ৪ = পাতলা পায়খানার সাথে রক্ত)*

CRF 28

গবেষণার ৬ষ্ঠ দিন

**পরিদর্শনের তারিখ**<br>(দিন মাস বছর)	**পরিদর্শনের সময়**<br>(ঘন্টা : মিনিট)
	:

গবেষণার জিনিসপত্র/ ঔষধ দেওয়ার সূচী

ডোজ	**তারিখ**<br>(দিন মাস বছর)	**সময়**<br>(ঘন্টা : মিনিট)	দেওয়ার পরিমান<br>(মিলি)	দেওয়ার বাকী আছে<br>(মিলি)

Study ID No.ঃ ☐☐☐

K	W	M	C	H

গবেষণার জিনিসপত্র/ ঔষধ দেওয়ার সূচী

ডোজ	তারিখ (দিন মাস বছর)	সময় (ঘন্টা : মিনিট)	দেওয়ার পরিমান (মিলি)	দেওয়ার বাকী আছে (মিলি)
১	☐☐ ☐☐☐ ☐☐	☐☐ : ☐☐	☐☐	☐☐
২	☐☐ ☐☐☐ ☐☐	☐☐ : ☐☐	☐☐	☐☐

অত্যাবশ্যকীয় লক্ষণ

**Variables** সময় গণনা	নাড়ীর গতি (প্রতি মিনিটে)	শ্বাসের গতি (প্রতি মিনিট)	তাপমাত্রা (বগলের) (০সে.)	ব্লাড প্রেসার. Systolic (mmHg) [শুধু মাত্র সকালে]	ব্লাড প্রেসার. **Diastolic** (mmHg) [শুধু মাত্র সকালে]
যদি কোন reading স্বাভাবিক পরিসীমা অতিক্রম করে?	☐ না ☐ হ্যাঁ	☐ না ☐ হ্যাঁ	☐ না ☐ হ্যাঁ	☐ না ☐ হ্যাঁ	☐ না ☐ হ্যাঁ
যদি হ্যাঁ হয় তবে, reading clinically Significant কিনা?*	☐ না ☐ হ্যাঁ	☐ না ☐ হ্যাঁ	☐ না ☐ হ্যাঁ	☐ না ☐ হ্যাঁ	☐ না ☐ হ্যাঁ

**যদি reading Clinically Significant হয় তবে প্রয়োজন হলে অনুগ্রহ করে Adverse Event Form পূরণ করুন]*

CRF 29

অন্যান্য লক্ষন সমূহ

বর্ণনা	না	হ্যাঁ
পেটে ব্যাথা আছে কিনা?	☐	☐
পেটে ফাঁপা আছে কিনা?	☐	☐
জ্বর আছে কিনা?	☐	☐
শারিরীক দুর্বলতা আছে কিনা?	☐	☐
বমি আছে কিনা?<br>যদি হ্যাঁ হয়, প্রতি দিন কত বার বমি হয় ? ☐☐	☐	☐

Study ID No.ঃ | | | |

K	W	M	C	H

অন্যান্য অস্বাভাবিকতা উপস্থিতি যদি হ্যাঁ হয়, নির্দিষ্ট করুন_______________	☐	☐
পায়খানার সাথে মলের *উপস্থিতির ইতিহাস*		☐
পায়খানার সাথে রক্তের *উপস্থিতির ইতিহাস*		
চাল ধোয়া পানির মত পায়খানার ইতিহাস		

শিশুটি বুকের দুধ খায় কিনা না ☐ হ্যাঁ ☐ **যদি হ্যাঁ হয়, প্রতিদিন কত বার খায় ?** ☐☐

শিশুটি তৈরি করা দুধ খায় কিনা না ☐ হ্যাঁ ☐ **যদি হ্যাঁ হয়, দিনে কত পরিমান (মি.লি)** ☐☐☐☐

সামগ্রিকভাবে খাবার এবং দুধ গ্রহনের পরিমান *(দিন শেষ হওয়ার ১ ঘন্টা পূর্বে)*

শিশুর খাবার / দুধ খাওয়ার অবস্থা নিচে উল্লেখিত স্কোর অনুযায়ী<br>(১=স্বাভাবিক, ২=কিছুটা কম, ৩=মধ্যম কম, ৪=খুব বেশি কম, ৫=খাবার গ্রহনে অস্বীকৃতি)	☐

পানি শূন্যতার পরিমান *(দিন শেষ হওয়ার ১ ঘন্টা পূর্বে)*

নিচে উল্লেখিত তালিকা অনুযায়ী শিশুর পানি শূন্যতা :<br>(০=পানি শূন্যতা নাই ১=কিছুটা পানি শূন্যতা ২= মারাত্মক পানি শূন্যতা *)<br>* যদি মারাত্মক পানি শূন্যতা হয় তাহলে শিশুকে গবেষণা থেকে বাদ দিন	☐

প্রতিকূল ঘটনায় প্রযোজ্য ঔষধ সমূহ

CRF 30

গত পরিদর্শনের সময় দেওয়া ঔষধ সমূহ/ **প্রতিকূল ঘটনার** পরিবর্তন হয়েছে কিনা?<br>*যদি হ্যাঁ হয় তবে প্রযোজ্য লিষ্ট থেকে নতুন ঔষধ যোগ করুন*	☐ না	☐ হ্যাঁ

পানীয় (ফাইবারসল-২/প্লাসিবু) গ্রহনের পর শিশুর প্রতিক্রিয়া :
৬ষ্ঠ দিনের ১ম ডোজ
৩০ মিনিটে ফাইবারসল-২/প্লাসিবু গ্রহনের পরিমান : ☐ . ☐ গ্রাম

বর্ণনা	না	হ্যাঁ
পেট ফুলে গিয়েছে কিনা?	☐	☐
পেটে ব্যাথা আছে কিনা?	☐	☐
পেট ডাকে কিনা?	☐	☐
পেটে ফাঁপা আছে কিনা?	☐	☐
শিশুটি খাবার থেকে মুখ ঘুরিয়ে নেয় কিনা ?	☐	☐
	☐	☐

Study ID No.ঃ | | | |

K	W	M	C	H

শিশুটি মুখ বন্ধ করে রাখে কিনা ?		
শিশুটি মাড়ি কামড়ে রাখে কিনা ?	☐	☐
শিশুটি বিরক্ত হয়েছে কিনা ?	☐	☐
শিশুটি মুখ থেকে **ফাইবারসল-২/প্লাসিবু** ফেলে দেয় কিনা ?	☐	☐
শিশুটি **ফাইবারসল-২/প্লাসিবু** গিলতে অস্বীকার করে কিনা ?	☐	☐

৬ষ্ঠ দিনের ২য় ডোজ
৩০ মিনিটে ফাইবারসল-২/প্লাসিবু গ্রহনের পরিমান : ☐ . ☐ গ্রাম

বর্ণনা	না	হ্যাঁ
পেট ফুলে গিয়েছে কিনা?	☐	☐
পেটে ব্যাথা আছে কিনা?	☐	☐
পেট ডাকে কিনা?	☐	☐
পেটে ফাঁপা আছে কিনা?	☐	☐
শিশুটি খাবার থেকে মুখ ঘুরিয়ে নেয় কিনা ?	☐	☐
শিশুটি মুখ বন্ধ করে রাখে কিনা ?	☐	☐
শিশুটি মাড়ি কামড়ে রাখে কিনা ?	☐	☐
শিশুটি বিরক্ত হয়েছে কিনা ?	☐	☐ CRF 31
শিশুটি মুখ থেকে **ফাইবারসল-২/প্লাসিবু** ফেলে দেয় কিনা ?	☐	☐
শিশুটি **ফাইবারসল-২/প্লাসিবু** গিলতে অস্বীকার করে কিনা ?	☐	☐

বমি আছে কিনা?<br>যদি হ্যাঁ হয়, প্রতি দিন কত বার বমি হয় ? ☐☐ বার<br>প্রতিদিন কি পরিমান বমি করেছে ☐☐☐ গ্রাম	না ☐ হ্যাঁ ☐
মায়ের দৃষ্টিতে শিশুটি **ফাইবারসল-২/প্লাসিবু পছন্দ করেছে কিনা ?**	☐

*** শিশুটি পানীয় নিতে অস্বীকার করেছে বলে বিবেচনা করবো যদি সে পানীয় থেকে মুখ ফিরিয়ে নেয়, কান্নাকাটি করে ,মুখ জোর করে বন্ধ করে রাখে অথবা দাত খিচিয়ে রাখে অথবা বিরক্ত হয়ে যায়, মুখ থেকে ফেলে দেয় কিংবা গিলতে অস্বীকার করে। আগে থেকে ওজন নেয়া নেপকিন সরবরাহ করা হবে। কোনো খাবার যদি উগড়ে ফেলে বা বমি করে ফেলে দেয়া হয়, তা মুছে নেপকিনের ওজন নেয়া হবে এবং সরবরাহকৃত পরিমান থেকে বাদ দেয়া হবে। ৭ পয়েন্টের হেডোনিক স্কেল ব্যবহার করা হবে যেখানে প্রতিটি পয়েন্ট মুখভঙ্গির অঙ্কন দিয়ে নির্ধারন করা হবে। আমরা মায়েদের থেকে খাবারের রং,গন্ধ, স্বাদ এবং সার্বিক গ্রহনযোগ্যতা সম্পর্কে মতামত চেয়েছি। (১=খুবই অপছন্দ, ২=কিছুটা অপছন্দ, ৩= অপছন্দ, ৪= পছন্দ-অপছন্দ কিছুই না, ৫=কিছুটা পছন্দ, ৬=মোটামুটি পছন্দ, ৭= খুবই পছন্দ)।

Study ID No.ঃ | | | |

K	W	M	C	H

পায়খানার ধরণ :	☐

* **পায়খানার ধরণ** *(১= শক্ত, ২= নরম, ৩ = পাতলা / পানির মত, ৪ = পাতলা পায়খানার সাথে রক্ত)*

CRF 32

গবেষণার ৭ম দিন

**পরিদর্শনের তারিখ** (দিন মাস বছর)	**পরিদর্শনের সময়** (ঘন্টা : মিনিট)
	:

গবেষণার জিনিসপত্র/ ঔষধ দেওয়ার সূচী

ডোজ	**তারিখ** (দিন মাস বছর)	**সময়** (ঘন্টা : মিনিট)	দেওয়ার পরিমান (মিলি)	দেওয়ার বাকী আছে (মিলি)

Study ID No.ঃ | | | |

K	W	M	C	H

গবেষণার জিনিসপত্র/ ঔষধ দেওয়ার সূচী

ডোজ	তারিখ (দিন মাস বছর)	সময় (ঘন্টা : মিনিট)	দেওয়ার পরিমান (মিলি)	দেওয়ার বাকী আছে (মিলি)
১		:		
২		:		

অত্যাবশ্যকীয় লক্ষণ

**Variables** সময় গণনা	নাড়ীর গতি (প্রতি মিনিটে)	শ্বাসের গতি (প্রতি মিনিট)	তাপমাত্রা (বগলের) (০সে.)	ব্লাড প্রেসার. Systolic (mmHg) [শুধু মাত্র সকালে]	ব্লাড প্রেসার. **Diastolic** (mmHg) [শুধু মাত্র সকালে]
যদি কোন reading স্বাভাবিক পরিসীমা অতিক্রম করে?	☐ না ☐ হ্যাঁ	☐ না ☐ হ্যাঁ	☐ না ☐ হ্যাঁ	☐ না ☐ হ্যাঁ	☐ না ☐ হ্যাঁ
যদি হ্যাঁ হয় তবে, reading clinically Significant কিনা?*	☐ না ☐ হ্যাঁ	☐ না ☐ হ্যাঁ	☐ না ☐ হ্যাঁ	☐ না ☐ হ্যাঁ	☐ না ☐ হ্যাঁ

**যদি reading Clinically Significant হয় তবে প্রয়োজন হলে অনুগ্রহ করে Adverse Event Form পূরণ করুন]*

CRF 33

অন্যান্য লক্ষন সমূহ

বর্ণনা	না	হ্যাঁ
পেটে ব্যাথা আছে কিনা?	☐	☐
পেটে ফাঁপা আছে কিনা?	☐	☐
জ্বর আছে কিনা?	☐	☐
শারিরীক দুর্বলতা আছে কিনা?	☐	☐
বমি আছে কিনা? যদি হ্যাঁ হয়, প্রতি দিন কত বার বমি হয় ?	☐	☐

Study ID No.ঃ | | | |

K	W	M	C	H

অন্যান্য অস্বাভাবিকতা উপস্থিতি যদি হ্যাঁ হয়, নির্দিষ্ট করুন______________	☐	☐
পায়খানার সাথে মলের *উপস্থিতির ইতিহাস*		☐
পায়খানার সাথে রক্তের *উপস্থিতির ইতিহাস*		
চাল ধোয়া পানির মত পায়খানার ইতিহাস		

শিশুটি বুকের দুধ খায় কিনা না ☐ হ্যাঁ ☐ **যদি হ্যাঁ হয়, প্রতিদিন কত বার খায় ?** ☐☐

শিশুটি তৈরি করা দুধ খায় কিনা না ☐ হ্যাঁ ☐ **যদি হ্যাঁ হয়, দিনে কত পরিমান (মি.লি)** ☐☐☐☐

সামগ্রিকভাবে খাবার এবং দুধ গ্রহনের পরিমান *(দিন শেষ হওয়ার ১ ঘন্টা পূর্বে)*

শিশুর খাবার / দুধ খাওয়ার অবস্থা নিচে উল্লেখিত স্কোর অনুযায়ী<br>(১=স্বাভাবিক, ২=কিছুটা কম, ৩=মধ্যম কম, ৪=খুব বেশি কম, ৫=খাবার গ্রহনে অস্বীকৃতি)	☐

পানি শূন্যতার পরিমান *(দিন শেষ হওয়ার ১ ঘন্টা পূর্বে)*

নিচে উল্লেখিত তালিকা অনুযায়ী শিশুর পানি শূন্যতা :<br>(০=পানি শূন্যতা নাই ১=কিছুটা পানি শূন্যতা ২= মারাত্বক পানি শূন্যতা *)<br>* যদি মারাত্বক পানি শূন্যতা হয় তাহলে শিশুকে গবেষণা থেকে বাদ দিন	☐

প্রতিকূল ঘটনায় প্রযোজ্য ঔষধ সমূহ

CRF 34

গত পরিদর্শনের সময় দেওয়া ঔষধ সমূহ/ **প্রতিকূল ঘটনার** পরিবর্তন হয়েছে কিনা?<br>*যদি হ্যাঁ হয় তবে প্রযোজ্য লিষ্ট থেকে নতুন ঔষধ যোগ করুন*	☐ না	☐ হ্যাঁ

পানীয় (ফাইবারসল-২/প্লাসিবু) গ্রহনের পর শিশুর প্রতিক্রিয়া :
৭ম দিনের ১ম ডোজ
৩০ মিনিটে ফাইবারসল-২/প্লাসিবু গ্রহনের পরিমান : ☐ . ☐ গ্রাম

বর্ণনা	না	হ্যাঁ
পেট ফুলে গিয়েছে কিনা?	☐	☐
পেটে ব্যাথা আছে কিনা?	☐	☐
পেট ডাকে কিনা?	☐	☐
পেটে ফাঁপা আছে কিনা?	☐	☐
শিশুটি খাবার থেকে মুখ ঘুরিয়ে নেয় কিনা ?	☐	☐
	☐	☐

Study ID No.ঃ | | | |

K	W	M	C	H

শিশুটি মুখ বন্ধ করে রাখে কিনা ?		
শিশুটি মাড়ি কামড়ে রাখে কিনা ?	☐	☐
শিশুটি বিরক্ত হয়েছে কিনা ?	☐	☐
শিশুটি মুখ থেকে **ফাইবারসল-২/প্লাসিবু** ফেলে দেয় কিনা ?	☐	☐
শিশুটি **ফাইবারসল-২/প্লাসিবু** গিলতে অস্বীকার করে কিনা ?	☐	☐

৭ম দিনের ২য় ডোজ

৩০ মিনিটে ফাইবারসল-২/প্লাসিবু গ্রহনের পরিমান : ☐ . ☐ গ্রাম

বর্ণনা	না	হ্যাঁ
পেট ফুলে গিয়েছে কিনা?	☐	☐
পেটে ব্যাথা আছে কিনা?	☐	☐
পেট ডাকে কিনা?	☐	☐
পেটে ফাঁপা আছে কিনা?	☐	☐
শিশুটি খাবার থেকে মুখ ঘুরিয়ে নেয় কিনা ?	☐	☐
শিশুটি মুখ বন্ধ করে রাখে কিনা ?	☐	☐
শিশুটি মাড়ি কামড়ে রাখে কিনা ?	☐	☐
শিশুটি বিরক্ত হয়েছে কিনা ?	☐	**CRF 35**
শিশুটি মুখ থেকে **ফাইবারসল-২/প্লাসিবু** ফেলে দেয় কিনা ?	☐	☐
শিশুটি **ফাইবারসল-২/প্লাসিবু** গিলতে অস্বীকার করে কিনা ?	☐	☐

বমি আছে কিনা?<br>যদি হ্যাঁ হয়, প্রতি দিন কত বার বমি হয় ? ☐☐ বার<br>প্রতিদিন কি পরিমান বমি করেছে ☐☐☐ গ্রাম	না ☐ হ্যাঁ ☐
মায়ের দৃষ্টিতে শিশুটি **ফাইবারসল-২/প্লাসিবু পছন্দ করেছে কিনা ?**	☐

*** শিশুটি পানীয় নিতে অস্বীকার করেছে বলে বিবেচনা করবো যদি সে পানীয় থেকে মুখ ফিরিয়ে নেয়, কান্নাকাটি করে ,মুখ জোর করে বন্ধ করে রাখে অথবা দাত খিচিয়ে রাখে অথবা বিরক্ত হয়ে যায়, মুখ থেকে ফেলে দেয় কিংবা গিলতে অস্বীকার করে। আগে থেকে ওজন নেয়া নেপকিন সরবরাহ করা হবে। কোনো খাবার যদি উগড়ে ফেলে বা বমি করে ফেলে দেয়া হয়, তা মুছে নেপকিনের ওজন নেয়া হবে এবং সরবরাহকৃত পরিমান থেকে বাদ দেয়া হবে। ৭ পয়েন্টের হেডোনিক স্কেল ব্যবহার করা হবে যেখানে প্রতিটি পয়েন্ট মুখভঙ্গির অঙ্কন দিয়ে নির্ধারন করা হবে। আমরা মায়েদের থেকে খাবারের রং,গন্ধ, স্বাদ এবং সার্বিক গ্রহনযোগ্যতা সর্ম্পকে মতামত চেয়েছি। (১=খুবই অপছন্দ, ২=কিছুটা অপছন্দ, ৩= অপছন্দ, ৪= পছন্দ-অপছন্দ কিছুই না, ৫=কিছুটা পছন্দ, ৬=মোটামুটি পছন্দ, ৭= খুবই পছন্দ)।

Study ID No.ঃ | | | |

K	W	M	C	H

পায়খানার ধরণ :	☐

* **পায়খানার ধরণ** *(১= শক্ত, ২= নরম, ৩ = পাতলা / পানির মত, ৪ = পাতলা পায়খানার সাথে রক্ত)*

CRF 36

৮ম দিন : ফলোআপ প্রশ্নপত্র

ফলোআপ পরিদর্শনের তারিখ :	দিন	মাস	বছর
পিতামাতা / বৈধ অভিভাবককে টেলিফোনে পাওয়া যাবে কিনা ?	☐ না	☐ হ্যাঁ	

অন্যান্য রোগের ইতিহাস

বর্ণনা	না	হ্যাঁ
পেট ফুলে গিয়েছে কিনা?	☐	☐
পেটে ব্যাথা আছে কিনা?	☐	☐
	☐	☐

Study ID No.ঃ | | | |

K	W	M	C	H

পেট ডাকে কিনা?		
পেটে ফাঁপা আছে কিনা?		
জ্বর আছে কিনা?	☐	☐
শারিরীক দুর্বলতা আছে কিনা?	☐	☐
বমি আছে কিনা?<br>যদি হ্যাঁ হয়, প্রতি দিন কত বার বমি হয়? ☐☐	☐	☐
অন্যান্য অস্বাভাবিকতা উপস্থিতি<br>যদি হ্যাঁ হয়, নির্দিষ্ট করুন________	☐	☐
পায়খানার সাথে মলের *উপস্থিতির ইতিহাস*	☐	☐
পায়খানার সাথে রক্তের *উপস্থিতির ইতিহাস*	☐	☐
চাল ধোয়া পানির মত পায়খানার ইতিহাস	☐	☐

সামগ্রিকভাবে খাবার এবং দুধ গ্রহন

☐ **(দুধ ১-৫ প্রতি স্কোর হিসাবে নিচে দেওয়া হল)**

**স্কোর**	**খাবার/ দুধ গ্রহন**	**প্রতিদিন দুধ গ্রহনের পরিমান**
১	স্বাভাবিক	☐
২	হালকা কম	
৩	মাঝারী কম	
৪	খুব কম	
৫	খাবার গ্রহনে অস্বীকৃতি	
যদি দুধ খায় অনুগ্রহ করে নির্দিষ্ট করুন?	☐ বুকের দুধ খায় ☐ প্রস্তুতকৃত দুধ খায় ☐ উভয়ই	

শিশুটি গত ২৪ ঘন্টায় পায়খানা করেছে কিনা ? না ☐ হ্যাঁ ☐ যদি হ্যাঁ হয়, কত বার ☐☐

পায়খানার ধরণ ☐

CRF 37

* **পায়খানার ধরণ** *(১= শক্ত, ২= নরম, ৩ = পাতলা / পানির মত, ৪ = পাতলা পায়খানার সাথে রক্ত ৯=প্রযোজ্য নয়)*

পানি শূন্যতার পরিমান *(দিন শেষ হওয়ার ১ ঘন্টা পূর্বে)*

উল্লেখিত তালিকা অনুযায়ী শিশুর পানি শূন্যতা :(০=পানি শূন্যতা নাই, ১=কিছুটা পানি শূন্যতা, ২= মারাত্বক পানি শূন্যতা)<br>* যদি মারাত্বক পানি শূন্যতা হয় তাহলে শিশুকে গবেষণা থেকে বাদ দিন	☐

৯ম দিন : ফলোআপ প্রশ্নপত্র

ফলোআপ পরিদর্শনের তারিখ : ☐☐ দিন ☐☐☐ মাস ☐☐ বছর

পিতামাতা / বৈধ অভিভাবককে টেলিফোনে পাওয়া যাবে কিনা ? ☐ না ☐ হ্যাঁ

Study ID No.ঃ | | | |

| K | W | M | C | H |

অন্যান্য রোগের ইতিহাস

বর্ণনা	না	হ্যাঁ
পেট ফুলে গিয়েছে কিনা?	☐	☐
পেটে ব্যাথা আছে কিনা?	☐	☐
পেট ডাকে কিনা?	☐	☐
পেটে ফাঁপা আছে কিনা?	☐	☐
জ্বর আছে কিনা?	☐	☐
শারিরীক দুর্বলতা আছে কিনা?	☐	☐
বমি আছে কিনা?<br>যদি হ্যাঁ হয়, প্রতি দিন কত বার বমি হয়? ☐☐	☐	☐
অন্যান্য অস্বাভাবিকতা উপস্থিতি<br>যদি হ্যাঁ হয়, নির্দিষ্ট করুন____________	☐	☐
পায়খানার সাথে মলের *উপস্থিতির ইতিহাস*	☐	☐
পায়খানার সাথে রক্তের *উপস্থিতির ইতিহাস*	☐	☐
চাল ধোয়া পানির মত পায়খানার ইতিহাস	☐	☐

সামগ্রিকভাবে খাবার এবং দুধ গ্রহন

☐ **(দুধ ১-৫ প্রতি স্কোর হিসাবে নিচে দেওয়া হল)**

স্কোর	খাবার/ দুধ গ্রহন	প্রতিদিন দুধ গ্রহনের পরিমান
১	স্বাভাবিক	☐
২	হালকা কম	
৩	মাঝারী কম	
৪	খুব কম	
৫	খাবার গ্রহনে অস্বীকৃতি	
যদি দুধ খায় অনুগ্রহ করে নির্দিষ্ট করুন?		☐ বুকের দুধ খায় ☐ প্রস্তুতকৃত দুধ খায় ☐ উভয়ই

☐☐

শিশুটি গত ২৪ ঘন্টায় পায়খানা করেছে কিনা ? না ☐ হ্যাঁ ☐ যদি হ্যাঁ হয়, কত বার

পায়খানার ধরণ ☐

* **পায়খানার ধরণ** *(১= শক্ত, ২= নরম, ৩ = পাতলা / পানির মত, ৪ = পাতলা পায়খানার সাথে রক্ত ৯=প্রযোজ্য নয়)*

Study ID No.ঃ | | | |

K	W	M	C	H

CRF 38

পানি শূন্যতার পরিমান *(দিন শেষ হওয়ার ১ ঘন্টা পূর্বে)*

উল্লেখিত তালিকা অনুযায়ী শিশুর পানি শূন্যতা :(০=পানি শূন্যতা নাই, ১=কিছুটা পানি শূন্যতা, ২= মারাত্বক পানি শূন্যতা)<br>* যদি মারাত্বক পানি শূন্যতা হয় তাহলে শিশুকে গবেষণা থেকে বাদ দিন	☐

১০ম দিন : ফলোআপ প্রশ্নপত্র

ফলোআপ পরিদর্শনের তারিখ :	☐☐ দিন ☐☐☐ মাস ☐☐ বছর
পিতামাতা / বৈধ অভিভাবককে টেলিফোনে পাওয়া যাবে কিনা ?	☐ না ☐ হ্যাঁ

অন্যান্য রোগের ইতিহাস

বর্ণনা	না	হ্যাঁ
পেট ফুলে গিয়েছে কিনা?	☐	☐
পেটে ব্যাথা আছে কিনা?	☐	☐
পেট ডাকে কিনা?	☐	☐
পেটে ফাঁপা আছে কিনা?	☐	☐
জ্বর আছে কিনা?	☐	☐
শারিরীক দুর্বলতা আছে কিনা?	☐	☐
বমি আছে কিনা?<br>যদি হ্যাঁ হয়, প্রতি দিন কত বার বমি হয়? ☐☐	☐	☐
অন্যান্য অস্বাভাবিকতা উপস্থিতি<br>যদি হ্যাঁ হয়, নির্দিষ্ট করুন________________________	☐	☐
পায়খানার সাথে মলের *উপস্থিতির ইতিহাস*	☐	☐
পায়খানার সাথে রক্তের *উপস্থিতির ইতিহাস*	☐	☐
চাল ধোয়া পানির মত পায়খানার ইতিহাস	☐	☐

সামগ্রিকভাবে খাবার এবং দুধ গ্রহন

☐ **(দুধ ১-৫ প্রতি স্কোর হিসাবে নিচে দেওয়া হল)**

স্কোর	খাবার/ দুধ গ্রহন	প্রতিদিন দুধ গ্রহনের পরিমান
১	স্বাভাবিক	
২	হালকা কম	
৩	মাঝারী কম	
৪	খুব কম	☐

Study ID No.ঃ | | | |

K	W	M	C	H

৫	খাবার গ্রহনে অস্বীকৃতি	

যদি দুধ খায় অনুগ্রহ করে নির্দিষ্ট করুন? ☐ বুকের দুধ খায় ☐ প্রস্তুতকৃত দুধ খায় ☐ উভয়ই

শিশুটি গত ২৪ ঘন্টায় পায়খানা করেছে কিনা ? না ☐ হ্যাঁ ☐ যদি হ্যাঁ হয়, কত বার ☐☐

পায়খানার ধরণ ☐

* **পায়খানার ধরণ** *(১= শক্ত, ২= নরম, ৩ = পাতলা / পানির মত, ৪ = পাতলা পায়খানার সাথে রক্ত ৯=প্রযোজ্য নয়)*

পানি শূন্যতার পরিমান *(দিন শেষ হওয়ার ১ ঘন্টা পূর্বে)*

CRF 39

উল্লেখিত তালিকা অনুযায়ী শিশুর পানি শূন্যতা :(০=পানি শূন্যতা নাই, ১=কিছুটা পানি শূন্যতা, ২= মারাত্বক পানি শূন্যতা)<br>* যদি মারাত্বক পানি শূন্যতা হয় তাহলে শিশুকে গবেষণা থেকে বাদ দিন	☐

১১তম দিন : ফলোআপ প্রশ্নপত্র

ফলোআপ পরিদর্শনের তারিখ : ☐☐ দিন ☐☐☐ মাস ☐☐ বছর

পিতামাতা / বৈধ অভিভাবককে টেলিফোনে পাওয়া যাবে কিনা ? ☐ না ☐ হ্যাঁ

অন্যান্য রোগের ইতিহাস

বর্ণনা	না	হ্যাঁ
পেট ফুলে গিয়েছে কিনা?	☐	☐
পেটে ব্যাথা আছে কিনা?	☐	☐
পেট ডাকে কিনা?	☐	☐
পেটে ফাঁপা আছে কিনা?	☐	☐
জ্বর আছে কিনা?	☐	☐
শারিরীক দুর্বলতা আছে কিনা?	☐	☐
বমি আছে কিনা?<br>যদি হ্যাঁ হয়, প্রতি দিন কত বার বমি হয়? ☐☐	☐	☐
অন্যান্য অস্বাভাবিকতা উপস্থিতি<br>যদি হ্যাঁ হয়, নির্দিষ্ট করুন________________	☐	☐
পায়খানার সাথে মলের *উপস্থিতির ইতিহাস*	☐	☐
পায়খানার সাথে রক্তের *উপস্থিতির ইতিহাস*	☐	☐
চাল ধোয়া পানির মত পায়খানার ইতিহাস	☐	☐

Study ID No.: | | | |

K	W	M	C	H

সামগ্রিকভাবে খাবার এবং দুধ গ্রহন

☐ (দুধ ১-৫ প্রতি স্কোর হিসাবে নিচে দেওয়া হল)

স্কোর	খাবার/ দুধ গ্রহন	প্রতিদিন দুধ গ্রহনের পরিমান
১	স্বাভাবিক	☐
২	হালকা কম	
৩	মাঝারী কম	
৪	খুব কম	
৫	খাবার গ্রহনে অস্বীকৃতি	
যদি দুধ খায় অনুগ্রহ করে নির্দিষ্ট করুন?		☐ বুকের দুধ খায় ☐ প্রস্তুতকৃত দুধ খায় ☐ উভয়ই

শিশুটি গত ২৪ ঘন্টায় পায়খানা করেছে কিনা ? না ☐ হ্যাঁ ☐ যদি হ্যাঁ হয়, কত বার ☐☐

পায়খানার ধরণ ☐

* **পায়খানার ধরণ** *(১= শক্ত, ২= নরম, ৩ = পাতলা / পানির মত, ৪ = পাতলা পায়খানার সাথে রক্ত ৯=প্রযোজ্য নয়)*

পানি শূন্যতার পরিমান *(দিন শেষ হওয়ার ১ ঘন্টা পূর্বে)*

CRF 40

উল্লেখিত তালিকা অনুযায়ী শিশুর পানি শূন্যতা :(০=পানি শূন্যতা নাই, ১=কিছুটা পানি শূন্যতা, ২= মারাত্বক পানি শূন্যতা)<br>* যদি মারাত্বক পানি শূন্যতা হয় তাহলে শিশুকে গবেষণা থেকে বাদ দিন	☐

১২তম দিন : ফলোআপ প্রশ্নপত্র

ফলোআপ পরিদর্শনের তারিখ : ☐☐ দিন ☐☐☐ মাস ☐☐ বছর

পিতামাতা / বৈধ অভিভাবককে টেলিফোনে পাওয়া যাবে কিনা ? ☐ না ☐ হ্যাঁ

অন্যান্য রোগের ইতিহাস

বর্ণনা	না	হ্যাঁ
পেট ফুলে গিয়েছে কিনা?	☐	☐
পেটে ব্যাথা আছে কিনা?	☐	☐
পেট ডাকে কিনা?	☐	☐
পেটে ফাঁপা আছে কিনা?	☐	☐
জ্বর আছে কিনা?	☐	☐
শারিরীক দুর্বলতা আছে কিনা?	☐	☐
বমি আছে কিনা?<br>যদি হ্যাঁ হয়, প্রতি দিন কত বার বমি হয়? ☐☐	☐	☐

Study ID No.ঃ | | | |

K	W	M	C	H

অন্যান্য অস্বাভাবিকতা উপস্থিতি<br>যদি হ্যাঁ হয়, নির্দিষ্ট করুন________________________	☐	☐
পায়খানার সাথে মলের *উপস্থিতির ইতিহাস*	☐	☐
পায়খানার সাথে রক্তের *উপস্থিতির ইতিহাস*	☐	☐
চাল ধোয়া পানির মত পায়খানার ইতিহাস	☐	☐

সামগ্রিকভাবে খাবার এবং দুধ গ্রহন

☐ **(দুধ ১-৫ প্রতি স্কোর হিসাবে নিচে দেওয়া হল)**

স্কোর	খাবার/ দুধ গ্রহন	প্রতিদিন দুধ গ্রহনের পরিমান
১	স্বাভাবিক	☐
২	হালকা কম	
৩	মাঝারী কম	
৪	খুব কম	
৫	খাবার গ্রহনে অস্বীকৃতি	
যদি দুধ খায় অনুগ্রহ করে নির্দিষ্ট করুন?		☐ বুকের দুধ খায় ☐ প্রস্তুতকৃত দুধ খায় ☐ উভয়ই

শিশুটি গত ২৪ ঘন্টায় পায়খানা করেছে কিনা ? না ☐ হ্যাঁ ☐ যদি হ্যাঁ হয়, কত বার ☐☐

পায়খানার ধরণ ☐

* **পায়খানার ধরণ** *(১= শক্ত, ২= নরম, ৩ = পাতলা / পানির মত, ৪ = পাতলা পায়খানার সাথে রক্ত ৯=প্রযোজ্য নয়)*

পানি শূন্যতার পরিমান *(দিন শেষ হওয়ার ১ ঘন্টা পূর্বে)*

CRF 41

উল্লেখিত তালিকা অনুযায়ী শিশুর পানি শূন্যতা :(০=পানি শূন্যতা নাই, ১=কিছুটা পানি শূন্যতা, ২= মারাত্মক পানি শূন্যতা)<br>* যদি মারাত্মক পানি শূন্যতা হয় তাহলে শিশুকে গবেষণা থেকে বাদ দিন	☐

১৩তম দিন : ফলোআপ প্রশ্নপত্র

ফলোআপ পরিদর্শনের তারিখ : ☐☐ দিন ☐☐☐ মাস ☐☐ বছর

পিতামাতা / বৈধ অভিভাবককে টেলিফোনে পাওয়া যাবে কিনা ? ☐ না ☐ হ্যাঁ

অন্যান্য রোগের ইতিহাস

বর্ণনা	না	হ্যাঁ
পেট ফুলে গিয়েছে কিনা?	☐	☐
পেটে ব্যাথা আছে কিনা?	☐	☐
পেট ডাকে কিনা?	☐	☐
পেটে ফাঁপা আছে কিনা?	☐	☐

Study ID No.ঃ | | | |

K	W	M	C	H

জ্বর আছে কিনা?	☐	☐
শারিরীক দুর্বলতা আছে কিনা?	☐	☐
বমি আছে কিনা? ☐☐<br>যদি হ্যাঁ হয়, প্রতি দিন কত বার বমি হয়?	☐	☐
অন্যান্য অস্বাভাবিকতা উপস্থিতি<br>যদি হ্যাঁ হয়, নির্দিষ্ট করুন________________________	☐	☐
পায়খানার সাথে মলের *উপস্থিতির ইতিহাস*	☐	☐
পায়খানার সাথে রক্তের *উপস্থিতির ইতিহাস*	☐	☐
চাল ধোয়া পানির মত পায়খানার ইতিহাস	☐	☐

সামগ্রিকভাবে খাবার এবং দুধ গ্রহন

☐ **(দুধ ১-৫ প্রতি স্কোর হিসাবে নিচে দেওয়া হল)**

স্কোর	খাবার/ দুধ গ্রহন	প্রতিদিন দুধ গ্রহনের পরিমান
১	স্বাভাবিক	
২	হালকা কম	
৩	মাঝারী কম	☐
৪	খুব কম	
৫	খাবার গ্রহনে অস্বীকৃতি	
যদি দুধ খায় অনুগ্রহ করে নির্দিষ্ট করুন?		☐ বুকের দুধ খায় ☐ প্রস্তুতকৃত দুধ খায় ☐ উভয়ই

শিশুটি গত ২৪ ঘন্টায় পায়খানা করেছে কিনা ? না ☐ হ্যাঁ ☐ যদি হ্যাঁ হয়, কত বার ☐☐

পায়খানার ধরণ ☐

* **পায়খানার ধরণ** *(১= শক্ত, ২= নরম, ৩ = পাতলা / পানির মত, ৪ = পাতলা পায়খানার সাথে রক্ত ৯=প্রযোজ্য নয়)*

CRF 42

পানি শূন্যতার পরিমান *(দিন শেষ হওয়ার ১ ঘন্টা পূর্বে)*

উল্লেখিত তালিকা অনুযায়ী শিশুর পানি শূন্যতা :(০=পানি শূন্যতা নাই, ১=কিছুটা পানি শূন্যতা, ২= মারাত্বক পানি শূন্যতা)<br>* যদি মারাত্বক পানি শূন্যতা হয় তাহলে শিশুকে গবেষণা থেকে বাদ দিন	☐

১৪ তম দিন : ফলোআপ প্রশ্নপত্র

ফলোআপ পরিদর্শনের তারিখ : ☐☐ দিন ☐☐☐ মাস ☐☐ বছর

পিতামাতা / বৈধ অভিভাবককে টেলিফোনে পাওয়া যাবে কিনা ? ☐ না ☐ হ্যাঁ

অত্যাবশ্যকীয় লক্ষণ

**Variables** সময় গণনা	**ওজন (কেজি)** [শুধু মাত্র সকালে]	**নাড়ীর গতি** (প্রতি মিনিটে)	**শ্বাসের গতি** (প্রতি মিনিট)	**বগলের (তাপমাত্রা)** (০সে.)	**ব্লাড প্রেসার. Systolic** (mmHg) [শুধু মাত্র সকালে]	**ব্লাড প্রেসার. Diastolic** (mmHg) [শুধু মাত্র সকালে]

Study ID No.ঃ | | | |

K	W	M	C	H

যদি কোন reading স্বাভাবিক পরিসীমা অতিক্রম করে?	☐ না ☐ হ্যাঁ	☐ না ☐ হ্যাঁ	☐ না ☐ হ্যাঁ	☐ না ☐ হ্যাঁ	☐ না ☐ হ্যাঁ	☐ না ☐ হ্যাঁ
যদি হ্যাঁ হয় তবে, reading clinically Significant কিনা?*	☐ না ☐ হ্যাঁ	☐ না ☐ হ্যাঁ	☐ না ☐ হ্যাঁ	☐ না ☐ হ্যাঁ	☐ না ☐ হ্যাঁ	☐ না ☐ হ্যাঁ
**যদি reading Clinically Significant হয় তবে প্রয়োজন হলে অনুগ্রহ করে Adverse Event Form পূরণ করুন]*						

অন্যান্য রোগের ইতিহাস

বর্ণনা	না	হ্যাঁ
পেট ফুলে গিয়েছে কিনা?	☐	☐
পেটে ব্যাথা আছে কিনা?	☐	☐
পেট ডাকে কিনা?	☐	☐
পেটে ফাঁপা আছে কিনা?	☐	☐
জ্বর আছে কিনা?	☐	☐
শারিরীক দুর্বলতা আছে কিনা?	☐	☐
বমি আছে কিনা?<br>যদি হ্যাঁ হয়, প্রতি দিন কত বার বমি হয়? ☐☐	☐	☐
অন্যান্য অস্বাভাবিকতা উপস্থিতি<br>যদি হ্যাঁ হয়, নির্দিষ্ট করুন______________________________	☐	☐
পায়খানার সাথে মলের *উপস্থিতির ইতিহাস*	☐	☐
পায়খানার সাথে রক্তের *উপস্থিতির ইতিহাস*	☐	☐
চাল ধোয়া পানির মত পায়খানার ইতিহাস	☐	☐

CRF 43

সামগ্রিকভাবে খাবার এবং দুধ গ্রহন

☐ **(দুধ ১-৫ প্রতি স্কোর হিসাবে নিচে দেওয়া হল)**

স্কোর	খাবার/ দুধ গ্রহন	প্রতিদিন দুধ গ্রহনের পরিমান
১	স্বাভাবিক	
২	হালকা কম	

Study ID No.ঃ | | | |

K	W	M	C	H

৩	মাঝারী কম	
৪	খুব কম	
৫	খাবার গ্রহনে অস্বীকৃতি	
যদি দুধ খায় অনুগ্রহ করে নির্দিষ্ট করুন? ☐ বুকের দুধ খায় ☐ প্রস্তুতকৃত দুধ খায় ☐ উভয়ই		

শিশুটি গত ২৪ ঘন্টায় পায়খানা করেছে কিনা ? না ☐ হ্যাঁ ☐ যদি হ্যাঁ হয়, কত বার ☐☐
পায়খানার ধরণ ☐

* **পায়খানার ধরণ** *(১= শক্ত, ২= নরম, ৩ = পাতলা / পানির মত, ৪ = পাতলা পায়খানার সাথে রক্ত ৯=প্রযোজ্য নয়)*

পানি শূন্যতার পরিমান *(দিন শেষ হওয়ার ১ ঘন্টা পূর্বে)*

উল্লেখিত তালিকা অনুযায়ী শিশুর পানি শূন্যতা :(০=পানি শূন্যতা নাই, ১=কিছুটা পানি শূন্যতা, ২= মারাত্বক পানি শূন্যতা)<br>* যদি মারাত্বক পানি শূন্যতা হয় তাহলে শিশুকে গবেষণা থেকে বাদ দিন	☐

Study ID No.ঃ [| |]

CRF 44

K	W	M	C	H

চুড়ান্ত মূল্যায়ন ফর্ম

অংশগ্রহন শেষ হওয়ার তারিখ:

গবেষণার দিন [|]   তারিখ [|] [| |] [|]

দিন   মাস   বছর

গবেষণার জিনিসপত্র/ ঔষধ গ্রহনের শেষ দিন:

গবেষণার দিন [|]   তারিখ : [|] [| |] [|]

দিন   মাস   বছর

শিশুটি কি গবেষণা সমাপ্ত করেছে ☐ না ☐ হ্যাঁ

যদি হ্যাঁ হয়, সমাপ্ত হওয়ার তারিখ [|] [| |] [|]

দিন   মাস   বছর

যদি শিশুটি গবেষনা সমাপ্ত না করে তাহলে প্রত্যাহারের কারন *?(একটি মাত্র বক্সে টিক চিহ্ন দিন)*

- ☐ সম্মতি প্রত্যাহার
- ☐ ফলোআপ সম্মব হয়নি *(appointment ব্যর্থ)*
- ☐ প্রত্যাহার করা হবে যদি পূর্বে উল্লেখিত অন্তভর্ক্তি শর্তাবলী ভঙ্গ হয় *(নির্দিষ্ট করুন)*________________
- ☐ প্রটোকল অনুযায়ী প্রত্যাহার *(নির্দিষ্ট করুন)*____
- ☐ প্রয়োজনীয় উন্নতির অভাবে হাসপাতালে অতিরিক্ত ভর্তি থাকা *(নির্দিষ্ট করুন)*

____

- ☐ প্রতিকূলতার জন্য প্রত্যাহার .

যদি প্রতিকূল অবস্থার কোন লক্ষন মারাত্বক হয় বা মৃত্যু হয় তবে চিকিৎসকে অবহিত করুন এবং তাৎক্ষনিক ভাবে ব্যবস্থা নিন.

মৃত্যু: মৃত্যুর তারিখ : [|] [| |] [|]

দিন   মাস   বছর

মৃত্যুর কারনগুলো : ____

ময়নাতদন্ত দ্বারা নিশ্চিত : ☐ না ☐ হ্যাঁ

গবেষকের স্বাক্ষর ________________________   স্বাক্ষরের তারিখ : [|] [| |] [|]

দিন   মাস   বছর

Study ID No.ঃ | | | |

| K | W | M | C | H |

CRF 45

বিচ্যুতিকরন সূচি

CRF পৃষ্ঠা নম্বর	পরিদর্শন এবং CRF তারিখ পদ	বিচ্যুতির বর্ণনা	EC- কে জানানো
			☐ না<br>☐ হ্যাঁ
			☐ না<br>☐ হ্যাঁ
			☐ না<br>☐ হ্যাঁ
			☐ না<br>☐ হ্যাঁ
			☐ না<br>☐ হ্যাঁ
			☐ না<br>☐ হ্যাঁ
			☐ না<br>☐ হ্যাঁ
			☐ না<br>☐ হ্যাঁ
			☐ না<br>☐ হ্যাঁ
			☐ না<br>☐ হ্যাঁ

Study ID No.:

K	W	M	C	H

Hospital ID No

CASE REPORT FORM

(Diarrhoeal Children)

Protocol Number: PR-16091

Protocol Title: Tolerability and Acceptability of Fibersol-2 (Resistant Maltodextrin) in healthy and diarrheal children followed by a randomized clinical trial to evaluate the efficacy of Fibersol-2 in diarrheal children 1-3 years old .

Principal Investigator

Dr. Mohammod Jobayer Chisti, MBBS, MMed, PhD, icddr,b

Sponsor

Matsutani Chemical Industry Company, Kagawa, Japan

INRORMED CONSENT

Date of Informed Consent signed: DD MON YY

Time of Informed Consent signed: HH : MM

To be signed upon completion of all of the CRF for this patient:

I certify that the information supplied herein is a complete and accurate record of the patient's data, that the study was carried out according to the protocol and that the patient's written informed consent was obtained from his representative, parent or guardian.

Principal Investigator's Signature: **Date:**

Principal Investigator's Name (PLEASE PRINT): **Dr. Mohammod Jobayer Chisti**

Study ID No.:

K	W	M	C	H

Hospital ID No

CASE REPORT FORM (CRF) GUIDELINES

1. Please use a black ball pen to complete the CRF. **Press firmly.**
2. Use capital letters fill the CRF.
3. Mark all choice fields (e.g.Yes/No boxes) with a "X"
4. Use 24-hour clock whenever it is asked to specify time, e.g. 9:00 pm to be captured as 21:00
5. Use leading zeros when appropriate e.g. If patients weight is 10.5 Kg then enter it as 010.5 Kg
6. For unknown values place "UK" in the box. e.g. Date:

DD		MON			YY	
U	K	D	E	C	1	4

Time:

HH		MM	
1	9	U	K

7. **If an error is made**
   - Do not obliterate or overwrite entry
   - Do not use correction fluid
   - Cross out with a single diagonal ~~56~~ 66SAS
   - Re-enter clearly as near as practical to original entry 10/04/13
   - Initial and date beside the data correction
8. Do not give added information which are not requested.
9. Please ensure that reasons for any concomitant therapy or treatments clearly match medical history terms and comment if there is no change from baseline or that the matching event term is reported as an adverse event.
10. For out of range height, weight, blood pressure or laboratory values please indicate if value is Clinically Significant (CS) and should be reported as an adverse event or if the value is Not Clinically Significant (NCS).

CRF 3

Study ID No.: [ ][ ][ ]

K	W	M	C	H

Hospital ID No

PRE-TREATMENT: ADMISSION

[PLEASE CAPTURE VALUE/NAME, OR CHECK APPROPRIATE RESPONSE IN COMPLETING FORM]

Date of Visit: DD MON YY

Time of Visit: HH : MM

GENERAL EXAMINATION DURING INITIAL REPORTING:
Vital signs

**Variables**	**Age** [months]	**Height** [cm]	**Weight(kg)** [morning only]	**Pulse Rate** (/Min)	**Resp. Rate** (/Min)	**Axillary Temp**(^{0}C)	**B.P.Systolic** (mmHg) [morning only]	**B.P Diastolic** (mmHg) [morning only]
Is any reading exceeding the normal range?			No / Yes	No / Yes	No / Yes	No / Yes	No / Yes	No / Yes
IF YES, is the deviation clinically significant?*			No / Yes	No / Yes	No / Yes	No / Yes	No / Yes	No / Yes

**[If Clinically Significant, please capture relevant details in Medical History or Adverse Event page as applicable]*

CRF 4

Study ID No.: ☐☐☐

K	W	M	C	H

Hospital ID No ☐☐☐☐☐☐☐☐☐☐

STATUS OF DEHYDRATION

Dehydration Status of child as per score provided below:<br>(0 =signs of dehydration 1=signs of Some dehydration 2 =signs of Severe dehydration*)<br>**If Dehydration is severe please exclude the subject from the study.*	☐

Correction by I/V fluid: ☐☐☐ ml Correction by ORS : ☐☐☐ ml

Whether any Zinc Supplement given? ☐ No ☐ Yes

Date of Zinc Intake: ☐☐ DD ☐☐☐ MON ☐☐ YY

Time of Zinc Intake: ☐☐ HH : ☐☐ MM

Formula milk provided and ingested? ☐ No ☐ Yes

If Yes,Volume ☐☐☐☐ ml

Is the child breastfed? ☐ No ☐ Yes

If Yes, Frequency ☐☐ per day

Is child Currently on Solid Food? ☐ No ☐ Yes

HISTORY OF DIARRHOEA

Date of Diarrhea Onset: ☐☐ DD ☐☐☐ MM ☐☐ YY Time of Diarrhea Onset: ☐☐ HH : ☐☐ MM

Diarrhea Duration ☐☐☐ (Hours)
[From onset to 1st dose]

Stool Frequency ☐☐ (per day)
[24 Hours prior to 1st dose]

Stool Consistency *(Check One)*	1 =Solid 2 =Paste 3 =Loose/watery 4 =Loose with blood<br>☐

[Guide: 1. Solid=Formed having its own shape; 2. Paste = soft; 3. Loose =watery and can be poured from one container to another; 4. Loose with blood present =Blood and mucus with or without faecal matter present]

Study ID No.: | | | |

K	W	M	C	H

Hospital ID No

HISTORY ASSOCIATED WITH DIARRHOEA

Details	Mother's Opinion		Interviewer's Opinion	
	NO	YES	NO	YES
Is Abdominal Pain present?	☐	☐	☐	☐
Is Abdominal Distension present?	☐	☐	☐	☐
Is Fever present?	☐	☐	☐	☐
Is Lethargy present?	☐	☐	☐	☐
Is Vomiting present?<br>If yes, number of vomiting per day ☐☐	☐	☐	☐	☐
Are other Abnormalities present?<br>If yes, specify____	☐	☐	☐	☐
History of mucus in stools	☐	☐	☐	☐
History of blood in stools	☐	☐	☐	☐
History of rice watery stools	☐	☐	☐	☐

STATUS OF OVERALL FOOD/MILK INTAKE

Mention food/Milk intake status of child as per scores provided below:<br>(1 = Normal, 2 =Mildly decreased, 3 =Moderately decreased, 4 =Severely decreased , 5 =Refused to eat	☐

Study ID No.:

K	W	M	C	H

Hospital ID No

SIGNIFICANT MEDICAL HISTORY (include jaundice, LRTI etc):

If none, check here ☐

Body System	NO	YES	If " YES", specify finding or diagnosis (if Known)
Ears, Nose, Throat:	☐	☐	
Opthalmological:	☐	☐	
Neurological:	☐	☐	
Cardiovascular:	☐	☐	
Respiratory:	☐	☐	
Gastrointestinal:	☐	☐	
Urogenital:	☐	☐	
Musculoskeletal:	☐	☐	
Dermatological:	☐	☐	
Endocrine:	☐	☐	
Hematological:	☐	☐	
Hepatic:	☐	☐	
Metabolic:	☐	☐	
Allergies and Drug Sensitivity:	☐	☐	

Study ID No.: | | | |

K	W	M	C	H

Hospital ID No

ENROLMENT CRITERIA CHECKLIST

INCLUSION CRITERIA

If any criterion is checked "NO," Subject cannot be enrolled into the study.	NO	YES
1. Male and Female Children aged 12 to 36 months	☐	☐
2. Acute diarrhoea with a duration < 72 hour without associated co-morbid conditions, e.g. acute respiratory tract infection, sepsis, gross electrolyte imbalances etc.	☐	☐
3. Guardian is willing to have the child admitted to the hospital as an inpatient for 72 hours and to return to the clinic with the child on Day 7 for a final evaluation	☐	☐
4. Guardian is willing and able to report to the Investigator information on stool frequency, stool consistency, vomiting frequency, food intake and ORS intake during the follow up phase	☐	☐
5. Written informed consent must be obtained prior to admission into this study	☐	☐
6. The subject has a physical examination that reveals no clinically significant abnormalities (altered mentation, convulsion, clinical suspicion of sepsis, meningitis, pneumonia,chronic gastrointestinal disease,acute gastroenteritis or any systemic infection other than those expected for subjects with acute infectious diarrhea	☐	☐

Study ID No.: | | | |

K	W	M	C	H

Hospital ID No

EXCLUSION CRITERIA

If any criterion is checked "YES," Subject cannot be enrolled into the study	NO	YES
1. Suspected dysentery and constipation(less than 3 stools per week)	☐	☐
2. Diarrhoea Symptoms duration > 72 hours at screening	☐	☐
3. Severe dehydration during triage or screening	☐	☐
4. Vomiting severity that is likely to make administration and retention of test product impossible	☐	☐
5. Severe malnutrition (defined as weight for height of less than -3 SD below median, as per WHO Standards)	☐	☐
6. Child received antibiotics, or anti-motility drugs two weeks prior to screening	☐	☐
7. History of hypersensitivity or allergy to milk or egg	☐	☐
8. History of vaccination against rotavirus	☐	☐
9.Currently participating in another clinical trial or child in a situation and could interfere with the optimal participation to the study or constitute a particular risk of non-compliance	☐	☐

ELIGIBILITY

Is patient eligible for entry into study? ☐ No ☐ Yes

If Yes:

Date of Randomization: |__|__| |__|__|__| |__|__| (DD MON YY) Time of Randomization: |__|__| : |__|__| (HH MM)

Randomization Number: |__|__|__|

Study ID No.: | K | W | M | C | H |

Hospital ID No

STUDY DAY 1

Date of Visit (DD MON YY)	Time of Visit (HH :MM)
	:

STUDY PRODUCT ADMINISTRATION SCHEDULE

Dose	Date (DD MON YY)	Time (HH:MM)	Volume Administered (ml)	Volume Remaining (ml)
1		:		

ZINC ADMINISTRATION SCHEDULE [Zinc to be administered with food at least 3 hours after test article administration]

Dose	Date (DD MON YY)	Time (HH:MM)	Volume Administered (ml)	Volume Remaining (ml)
1		:	.	

STUDY PRODUCT ADMINISTRATION SCHEDULE

Dose	Date (DD MON YY)	Time (HH:MM)	Volume Administered (ml)	Volume Remaining (ml)
2		:		

ZINC ADMINISTRATION SCHEDULE [Zinc to be administered with food at least 3 hours after test article administration]

Dose	Date (DD MON YY)	Time (HH:MM)	Volume Administered (ml)	Volume Remaining (ml)
2		:	.	

Study ID No.: | K | W | M | C | H |

CRF 10

Hospital ID No

VITAL SIGNS

**Variables Time measured (Measure at end of each 8-hour period)**	**Weight (Kg)** [morning only]	**Pulse Rate** (/Min	**Respiratory Rate** (/Min)	**Axillary Temp** (°C)	**B.P. Systolic** (mmHg) [morning only]	**B.P. Diastolic** (mmHg) [morning only]
1st 8-Hour Period						
2nd 8-Hour Period						
3rd 8-Hour Period						
Is any reading outside of normal range?	☐ No ☐ Yes	☐ No ☐ Yes	☐ No ☐ Yes	☐ No ☐ Yes	☐ No ☐ Yes	☐ No ☐ Yes
IF YES, is the deviation Clinically Significant? *	☐ No ☐ Yes	☐ No ☐ Yes	☐ No ☐ Yes	☐ No ☐ Yes	☐ No ☐ Yes	☐ No ☐ Yes

**If Clinically Significant please fill up the Adverse Event Form as applicable.*

HISTORY ASSOCIATED WITH DIARRHOEA

**Details**	**NO**	**YES**
Is Abdominal Pain present?	☐	☐
Is Abdominal Distension present?	☐	☐
Is Fever present?	☐	☐
Is Lethargy present?	☐	☐
Is Vomiting present?<br>If Yes, number of vomiting per day ☐☐	☐	☐
Are other Abnormalities present?<br>If Yes, specify____	☐	☐
History of mucus in stools	☐	☐
History of blood in stools	☐	☐
History of rice watery stools	☐	☐

Study ID No.: | | | |

K	W	M	C	H

Hospital ID No

FOOD AND ORS INTAKE

**Date** (DD MON YY)	**Time of Intake 8-Hourly** (HH:MM)	**Food Intake** (g)	**ORS/Rice Saline Intake** (ml)	**Formula Milk** (ml)	**Was the subject breast fed**	**If Yes frequency**
	:				No / Yes	
	:				No / Yes	
	:				No / Yes	

STOOL AND URINE OUTPUT

**Date** (DD MON YY)	**Time of Measurement 8-Hourly** (HH MM)	**Has watery stool been passed in this 8-hour period**	**Weight of stool** (g)	**Stool frequency**	**Stool consistency ***	**Urine output (ml)**
	:	No Yes				
	:	No Yes				
	:	No Yes				

**Stool consistency (1=Solid, 2 Paste, 3 =Loose/watery, 4 = Loose with blood)*

STATUS OF OVERALL FOOD/ MILK INTAKE *(1 Hour before day ends)*

Mention food/Milk intake status of child as per scores provided below:<br>(1=Normal, 2=Mildly decreased, 3=Moderately decreased, 4=Severely decreased, 5=Refused to eat)	

STATUS OF DEHYDRATION *(1 Hour before day ends)*

Dehydration Status of child as per score provided below:<br>(0= no signs of dehydration 1=signs of Some dehydration 2 =signs of Severe dehydration*)<br>**If Dehydration is severe please exclude the subject from the study*	

ADVERSE EVENTS CONCOMITANT MEDICATION

Were there any changes in Adverse Events or Concomitant Medications since the last visit?<br><br>*If yes, please add new events/medications on appropriate log*	No	Yes

Study ID No.: ☐☐☐

K	W	M	C	H

Hospital ID No ☐☐☐☐☐☐☐☐☐☐

Reaction of Child after intake of Study Product (Fibersol-2/Placebo):
For 1st dose of the day 01
Amount of intake of Fibersol-2/Placebo in 30 mins: ☐ . ☐ **gm**

Description	No	Yes
Is Abdominal Distension present?	☐	☐
Is Abdominal Pain present?	☐	☐
Is Abdominal Rumbling present?	☐	☐
Is Abdominal Bloating present?	☐	☐
Child moved head away from the food?	☐	☐
Mouth clamped shut?	☐	☐
Teeth clenched?	☐	☐
Became agitated?	☐	☐
Spit out of the given drink?	☐	☐
Refused to swallow the given drink?	☐	☐

For 2nd dose of the day 01
Amount of intake of Fibersol-2/Placebo in 30 mins: ☐ . ☐ **gm**

Description	No	Yes
Is Abdominal Distension present?	☐	☐
Is Abdominal Pain present?	☐	☐
Is Abdominal Rumbling present?	☐	☐
Is Abdominal Bloating present?	☐	☐
Child moved head away from the food?	☐	☐
Mouth clamped shut?	☐	☐
Teeth clenched?	☐	☐
Became agitated?	☐	☐
Spit out of the given drink?	☐	☐
Refused to swallow the given drink?	☐	☐

Study ID No.: | | | |

K	W	M	C	H

Hospital ID No

Is vomiting present?<br>If yes, frequency per day? ☐☐<br><br>Amount of vomiting per day ☐☐☐ gm	**No** ☐ **Yes** ☐
In mother's opinion, the child likes Fibersol-2/placebo on not?	☐

*** Children were considered as refusing intake if they moved their head away from the food, cried, clamped the mouth shut or clenched the teeth, or became agitated, spit out the food or refused to swallow. The amount of food ingested was calculated by subtracting the left-over from the offered amount. Pre-weighed napkins were provided; any food that was regurgitated, vomited or spilled was swabbed, the napkin weighed and subtracted from the weight of the amount offered. Using a 7-point Hedonic Scale in which each point (1 = disliked extremely, 2 = disliked moderately, 3 = disliked, 4 = neither disliked nor liked, 5 = liked slightly, 6 = liked moderately, 7 = liked extremely) was depicted by a facial drawing, we asked mothers to rate the food's color, flavor, mouth feel, and overall acceptability.

Stool Consistency:	☐

* Stool Consistency: 1 =Solid 2 =Paste 3 =Loose/watery 4 =Loose with blood

Study ID No.: K W M C H

Hospital ID No

STUDY DAY 2

Date of Visit (DD MON YY)	Time of Visit (HH:MM)
	:

STUDY PRODUCT ADMINISTRATION SCHEDULE

Dose	Date (DD MON YY)	Time (HH:MM)	Volume Administered (ml)	Volume Remaining (ml)
**1**		:		

ZINC ADMINISTRATION SCHEDULE *[Zinc to be administered with food at least 3 hours after test article administration]*

Dose	Date (DD MON YY)	Time (HH: MM)	Volume Administered (ml)	Volume Remaining (ml)
**1**		:	.	

STUDY PRODUCT ADMINISTRATION SCHEDULE

Dose	Date (DD MON YY)	Time (HH:MM)	Volume Administered (ml)	Volume Remaining (ml)
**2**		:		

ZINC ADMINISTRATION SCHEDULE *[Zinc to be administered with food at least 3 hours after test article administration]*

Dose	Date (DD MON YY)	Time (HH:MM)	Volume Administered (ml)	Volume Remaining (ml)
**2**		:	.	

Study ID No.: | | | |

K	W	M	C	H

Hospital ID No

VITAL SIGNS

Variables Time measured (Measure at end of each 8-hour period)	Weight (Kg) [morning only]	Pulse Rate (/Min	Respiratory Rate (/Min)	Axillary Temp (°C)	B.P. Systolic (mmHg) [morning only]	B.P. Diastolic (mmHg) [morning only]
1st 8-Hour Period						
2nd 8-Hour Period						
3rd 8-Hour Period						
Is any reading outside of normal range?	☐ No ☐ Yes	☐ No ☐ Yes	☐ No ☐ Yes	☐ No ☐ Yes	☐ No ☐ Yes	☐ No ☐ Yes
IF YES, is the deviation Clinically Significant? *	☐ No ☐ Yes	☐ No ☐ Yes	☐ No ☐ Yes	☐ No ☐ Yes	☐ No ☐ Yes	☐ No ☐ Yes

**If Clinically Significant please fill up the Adverse Event Form as applicable.*

HISTORY ASSOCIATED WITH DIARRHOEA

Details	NO	YES
Is Abdominal Pain present?	☐	☐
Is Abdominal Distension present?	☐	☐
Is Fever present?	☐	☐
Is Lethargy present?	☐	☐
Is Vomiting present?<br>If Yes, number of vomiting per day ☐☐	☐	☐
Are other Abnormalities present?<br>If Yes, specify______________________________	☐	☐
History of mucus in stools	☐	☐
History of blood in stools	☐	☐
History of rice watery stools	☐	☐

Study ID No.: | | | |

K	W	M	C	H

Hospital ID No

FOOD AND ORS INTAKE

**Date** (DD MON YY)	**Time of Intake 8-Hourly** (HH:MM)	**Food Intake** (g)	**ORS/Rice Saline Intake** (ml)	**Formula Milk** (ml)	**Was the subject breast fed**	**If Yes frequency**
	:				No<br>Yes	
	:				No<br>Yes	
	:				No<br>Yes	

STOOL AND URINE OUTPUT

**Date** (DD MON YY)	**Time of Measurement 8-Hourly** (HH:MM)	**Has watery stool been passed in this 8-hour period**	**Weight of stool** (g)	**Stool frequency**	**Stool consistency***	**Urine output (ml)**
	:	No Yes				
	:	No Yes				
	:	No Yes				

**Stool consistency (1=Solid, 2=Paste, 3 =Loose/watery, 4 =Loose with blood)*

STATUS OF OVERALL FOOD/ MILK INTAKE *(1 Hour before day ends)*

Mention food/Milk intake status of child as per scores provided below:<br>(1=Normal, 2=Mildly decreased, 3=Moderately decreased, 4=Severely decreased, 5=Refused to eat)	

STATUS OF DEHYDRATION *(1 Hour before day ends)*

Dehydration Status of child as per score provided below:<br>(0 =no signs of dehydration 1=signs of Some dehydration 2=signs of Severe dehydration*)<br>**If Dehydration is severe please exclude the subject from the study*	

ADVERSE EVENTS AND CONCOMITANT MEDICATIONS

Were there any changes in Adverse Events or Concomitant Medications since the last visit?<br>*If yes, please add new events/medications on appropriate log*	No	Yes

Study ID No.: | | | |

K	W	M	C	H

Hospital ID No

Reaction of Child after intake of Study Product (Fibersol-2/Placebo):
For 1st dose of the day 02
Amount of intake of Fibersol-2/Placebo in 30 mins: ☐ . ☐ **gm**

Description	No	Yes
Is Abdominal Distension present?	☐	☐
Is Abdominal Pain present?	☐	☐
Is Abdominal Rumbling present?	☐	☐
Is Abdominal Bloating present?	☐	☐
Child moved head away from the food?	☐	☐
Mouth clamped shut?	☐	☐
Mouth clenched?	☐	☐
Became agitated?	☐	☐
Spit out of the given drink?	☐	☐
Refused to swallow the given drink?	☐	☐

For 2nd dose of the day 02
Amount of intake of Fibersol-2/Placebo in 30 mins: ☐ . ☐ **gm**

Description	No	Yes
Is Abdominal Distension present?	☐	☐
Is Abdominal Pain present?	☐	☐
Is Abdominal Rumbling present?	☐	☐
Is Abdominal Bloating present?	☐	☐
Child moved head away from the food?	☐	☐
Mouth clamped shut?	☐	☐
Mouth clenched?	☐	☐
Became agitated?	☐	☐
Spit out of the given drink?	☐	☐
Refused to swallow the given drink?	☐	☐

Study ID No.: | | | |

K	W	M	C	H

Hospital ID No

Is vomiting present?<br>If yes, frequency per day? ☐☐<br><br>Amount of vomiting per day ☐☐☐ gm	**No** ☐ **Yes** ☐
In mother's opinion, the child likes Fibersol-2/placebo on not?	☐

*** Children were considered as refusing intake if they moved their head away from the food, cried, clamped the mouth shut or clenched the teeth, or became agitated, spit out the food or refused to swallow. The amount of food ingested was calculated by subtracting the left-over from the offered amount. Pre-weighed napkins were provided; any food that was regurgitated, vomited or spilled was swabbed, the napkin weighed and subtracted from the weight of the amount offered. Using a 7-point Hedonic Scale in which each point (1 = disliked extremely, 2 = disliked moderately, 3 = disliked, 4 = neither disliked nor liked, 5 = liked slightly, 6 = liked moderately, 7 = liked extremely) was depicted by a facial drawing, we asked mothers to rate the food's color, flavor, mouth feel, and overall acceptability.

Stool Consistency:	☐

* Stool Consistency: 1 =Solid 2 =Paste 3 =Loose/watery 4 =Loose with blood

Study ID No.: | | | |

K	W	M	C	H

Hospital ID No

STUDY DAY 3

**Date of Visit** (DD MON YY)	**Time of Visit** (HH:MM)
	:

STUDY PRODUCT ADMINISTRATION SCHEDULE

Dose	**Date** (DD MON YY)	**Time** (HH:MM)	Volume Administered (ml)	Volume Remaining (ml)
**1**		:		

ZINC ADMINISTRATION SCHEDULE *[Zinc to be administered with food at least 3 hours after test article administration]*

Dose	**Date** (DD MON YY)	**Time** (HH: MM)	Volume Administered (ml)	Volume Remaining (ml)
**1**		:	.	

STUDY PRODUCT ADMINISTRATION SCHEDULE

Dose	**Date** (DD MON YY)	**Time** (HH:MM)	Volume Administered (ml)	Volume Remaining (ml)
**2**		:		

ZINC ADMINISTRATION SCHEDULE *[Zinc to be 19administered with food at least 3 hours after test article administration]*

Dose	**Date** (DD MON YY)	**Time** (HH:MM)	Volume Administered (ml)	Volume Remaining (ml)
**2**		:	.	

Study ID No.: [| |]

K	W	M	C	H

Hospital ID No

VITAL SIGNS

Variables Time measured (Measure at end of each 8-hour period)	Weight (Kg) [morning only]	Pulse Rate (/Min	Respiratory Rate (/Min)	Axillary Temp (°C)	B.P. Systolic (mmHg) [morning only]	B.P. Diastolic (mmHg) [morning only]
1st 8-Hour Period						
2nd 8-Hour Period						
3rd 8-Hour Period						
Is any reading outside of normal range?	☐ No ☐ Yes	☐ No ☐ Yes	☐ No ☐ Yes	☐ No ☐ Yes	☐ No ☐ Yes	☐ No ☐ Yes
IF YES, is the deviation Clinically Significant? *	☐ No ☐ Yes	☐ No ☐ Yes	☐ No ☐ Yes	☐ No ☐ Yes	☐ No ☐ Yes	☐ No ☐ Yes

**If Clinically Significant please fill up the Adverse Event Form as applicable.*

HISTORY ASSOCIATED WITH DIARRHOEA

Details	NO	YES
Is Abdominal Pain present?	☐	☐
Is Abdominal Distension present?	☐	☐
Is Fever present?	☐	☐
Is Lethargy present?	☐	☐
Is Vomiting present?<br>If Yes, number of vomiting per day [|]	☐	☐
Are other Abnormalities present?<br>If Yes, specify____	☐	☐
History of mucus in stools	☐	☐
History of blood in stools	☐	☐
History of rice watery stools	☐	☐

Study ID No.: ☐☐☐

K	W	M	C	H

Hospital ID No ☐☐☐☐☐☐☐☐☐☐

FOOD AND ORS INTAKE

Date (DD MON YY)	Time of Intake 8-Hourly (HH:MM)	Food Intake (g)	ORS/Rice Saline Intake (ml)	Formula Milk (ml)	Was the subject breast fed	If Yes frequency
☐☐ ☐☐☐ ☐☐	☐☐ : ☐☐	☐☐☐☐	☐☐☐☐	☐☐☐☐	☐ No ☐ Yes	☐☐
☐☐ ☐☐☐ ☐☐	☐☐ : ☐☐	☐☐☐☐	☐☐☐☐	☐☐☐☐	☐ No ☐ Yes	☐☐
☐☐ ☐☐☐ ☐☐	☐☐ : ☐☐	☐☐☐☐	☐☐☐☐	☐☐☐☐	☐ No ☐ Yes	☐☐

STOOL AND URINE OUTPUT

Date (DD MON YY)	Time of Measurement 8-Hourly (HH:MM)	Has watery stool been passed in this 8-hour period	Weight of stool (g)	Stool frequency	Stool consistency*	Urine output (ml)
☐☐ ☐☐☐ ☐☐	☐☐ : ☐☐	☐No ☐Yes	☐☐☐☐	☐☐	☐	☐☐☐☐
☐☐ ☐☐☐ ☐☐	☐☐ : ☐☐	☐No ☐Yes	☐☐☐☐	☐☐	☐	☐☐☐☐
☐☐ ☐☐☐ ☐☐	☐☐ : ☐☐	☐No ☐Yes	☐☐☐☐	☐☐	☐	☐☐☐☐

**Stool consistency (1= Solid, 2=Paste, 3 =Loose/watery, 4 =Loose with blood)*

STATUS OF OVERALL FOOD/ MILK INTAKE *(1 Hour before day ends)*

Mention food/Milk intake status of child as per scores provided below:<br>(1=Normal, 2=Mildly decreased, 3=Moderately decreased, 4=Severely decreased, 5=Refused to eat)	☐

STATUS OF DEHYDRATION *(1 Hour before day ends)*

Dehydration Status of child as per score provided below:<br>(0 =no signs of dehydration 1=signs of Some dehydration 2=signs of Severe dehydration*)<br>**If Dehydration is severe please exclude the subject from the study*	☐

ADVERSE EVENTS AND CONCOMITANT MEDICATIONS

Were there any changes in Adverse Events or Concomitant Medications since the last visit?<br>*If yes, please add new events/medications on appropriate log*	☐ No	☐ Yes

Study ID No.: | | | |

K	W	M	C	H

Hospital ID No

Reaction of Child after intake of Study Product (Fibersol-2/Placebo):
For 1st dose of the day 03
Amount of intake of Fibersol-2/Placebo in 30 mins: ☐ . ☐ gm

Description	No	Yes
Is Abdominal Distension present?	☐	☐
Is Abdominal Pain present?	☐	☐
Is Abdominal Rumbling present?	☐	☐
Is Abdominal Bloating present?	☐	☐
Child moved head away from the food?	☐	☐
Mouth clamped shut?	☐	☐
Mouth clenched?	☐	☐
Became agitated?	☐	☐
Spit out of the given drink?	☐	☐
Refused to swallow the given drink?	☐	☐

For 2nd dose of the day 03
Amount of intake of Fibersol-2/Placebo in 30 mins: ☐ . ☐ gm

Description	No	Yes
Is Abdominal Distension present?	☐	☐
Is Abdominal Pain present?	☐	☐
Is Abdominal Rumbling present?	☐	☐
Is Abdominal Bloating present?	☐	☐
Child moved head away from the food?	☐	☐
Mouth clamped shut?	☐	☐
Mouth clenched?	☐	☐
Became agitated?	☐	☐
Spit out of the given drink?	☐	☐
Refused to swallow the given drink?	☐	☐

Study ID No.: | | | |

K	W	M	C	H

Hospital ID No

Is vomiting present?<br>If yes, frequency per day? ☐☐<br>Amount of vomiting per day ☐☐☐ gm	**No** ☐ **Yes** ☐
In mother's opinion, the child likes Fibersol-2/placebo on not?	☐

*** Children were considered as refusing intake if they moved their head away from the food, cried, clamped the mouth shut or clenched the teeth, or became agitated, spit out the food or refused to swallow. The amount of food ingested was calculated by subtracting the left-over from the offered amount. Pre-weighed napkins were provided; any food that was regurgitated, vomited or spilled was swabbed, the napkin weighed and subtracted from the weight of the amount offered. Using a 7-point Hedonic Scale in which each point (1 = disliked extremely, 2 = disliked moderately, 3 = disliked, 4 = neither disliked nor liked, 5 = liked slightly, 6 = liked moderately, 7 = liked extremely) was depicted by a facial drawing, we asked mothers to rate the food's color, flavor, mouth feel, and overall acceptability.

Stool consistency:	☐

* Stool consistency: 1 =Solid 2 =Paste 3 = Loose/watery 4 =Loose with blood

Study ID No.: ☐☐☐

K	W	M	C	H

Hospital ID No ☐☐☐☐☐☐☐☐☐☐

STUDY DAY 4

Date of Visit (DD MON YY)	Time of Visit (HH: MM)
☐☐ ☐☐☐ ☐☐	☐☐ : ☐☐

STUDY PRODUCT ADMINISTRATION SCHEDULE

Dose	Date (DD MON YY)	Time (HH:MM)	Volume Administered (ml)	Volume Remaining (ml)
**1**	☐☐ ☐☐☐ ☐☐	☐☐ : ☐☐	☐☐	☐☐

ZINC ADMINISTRATION SCHEDULE *[Zinc to be administered with food at least 3 hours after test article administration]*

Dose	Date (DD MON YY)	Time (HH: MM)	Volume Administered (ml)	Volume Remaining (ml)
**1**	☐☐ ☐☐☐ ☐☐	☐☐ : ☐☐	☐☐.☐	☐☐

STUDY PRODUCT ADMINISTRATION SCHEDULE

Dose	Date (DD MON YY)	Time (HH:MM)	Volume Administered (ml)	Volume Remaining (ml)
**2**	☐☐ ☐☐☐ ☐☐	☐☐ : ☐☐	☐☐	☐☐

ZINC ADMINISTRATION SCHEDULE *[Zinc to be administered with food at least 3 hours after test article administration]*

Dose	Date (DD MON YY)	Time (HH:MM)	Volume Administered (ml)	Volume Remaining (ml)
**2**	☐☐ ☐☐☐ ☐☐	☐☐ : ☐☐	☐☐.☐	☐☐

Study ID No.: | K | W | M | C | H | | | |

Hospital ID No | | | | | | | | | | |

VITAL SIGNS

Variables Time measured (Measure at end of each 8-hour period)	Weight (Kg) [morning only]	Pulse Rate (/Min	Respiratory Rate (/Min)	Axillary Temp (°C)	B.P. Systolic (mmHg) [morning only]	B.P. Diastolic (mmHg) [morning only]
1st 8-Hour Period						
2nd 8-Hour Period						
3rd 8-Hour Period						
Is any reading outside of normal range?	☐ No ☐ Yes	☐ No ☐ Yes	☐ No ☐ Yes	☐ No ☐ Yes	☐ No ☐ Yes	☐ No ☐ Yes
IF YES, is the deviation Clinically Significant? *	☐ No ☐ Yes	☐ No ☐ Yes	☐ No ☐ Yes	☐ No ☐ Yes	☐ No ☐ Yes	☐ No ☐ Yes

**If Clinically Significant please fill up the Adverse Event Form as applicable.*

HISTORY ASSOCIATED WITH DIARRHOEA

Details	NO	YES
Is Abdominal Pain present?	☐	☐
Is Abdominal Distension present?	☐	☐
Is Fever present?	☐	☐
Is Lethargy present?	☐	☐
Is Vomiting present?<br>If Yes, number of vomiting per day ☐☐	☐	☐
Are other Abnormalities present?<br>If Yes, specify____	☐	☐
History of mucus in stools	☐	☐
History of blood in stools	☐	☐
History of rice watery stools	☐	☐

Study ID No.: K W M C H

Hospital ID No

FOOD AND ORS INTAKE

**Date** (DD MON YY)	**Time of Intake 8-Hourly** (HH :MM)	**Food Intake** (g)	**ORS/Rice Saline Intake** (ml)	**Formula Milk** (ml)	**Was the subject breast fed**	**If Yes frequency**
	:				No Yes	
	:				No Yes	
	:				No Yes	

STOOL AND URINE OUTPUT

**Date** (DD MON YY)	**Time of Measurement 8-Hourly** (HH:MM)	**Has watery stool been passed in this 8-hour period**	**Weight of stool** (g)	**Stool frequency**	**Stool consistency***	**Urine output (ml)**
	:	No Yes				
	:	No Yes				
	:	No Yes				

**Stool consistency (1= Solid, 2= Paste, 3 =Loose/watery, 4=Loose with blood)*

STATUS OF OVERALL FOOD/ MILK INTAKE *(1 Hour before day ends)*

Mention food/Milk intake status of child as per scores provided below:<br>(1=Normal, 2=Mildly decreased, 3=Moderately decreased, 4=Severely decreased, 5=Refused to eat)	

STATUS OF DEHYDRATION *(1 Hour before day ends)*

Dehydration Status of child as per score provided below:<br>(0 =no signs of dehydration 1= signs of Some dehydration 2 =signs of Severe dehydration*)<br>**If Dehydration is severe please exclude the subject from the study*	

ADVERSE EVENTS AND CONCOMITANT MEDICATIONS

Were there any changes in Adverse Events or Concomitant Medications since the last visit?<br>*If yes, please add new events/medications on appropriate log*	No	Yes

Study ID No.: | | | |

K	W	M	C	H

Hospital ID No

Reaction of Child after intake of Study Product (Fibersol-2/Placebo):
For 1st dose of the day 04
Amount of intake of Fibersol-2/Placebo in 30 mins: ☐ . ☐ gm

Description	No	Yes
Is Abdominal Distension present?	☐	☐
Is Abdominal Pain present?	☐	☐
Is Abdominal Rumbling present?	☐	☐
Is Abdominal Bloating present?	☐	☐
Child moved head away from the food?	☐	☐
Mouth clamped shut?	☐	☐
Teeth clenched?	☐	☐
Became agitated?	☐	☐
Spit out of the given drink?	☐	☐
Refused to swallow the given drink?	☐	☐

For 2nd dose of the day 04
Amount of intake of Fibersol-2/Placebo in 30 mins: ☐ . ☐ gm

Description	No	Yes
Is Abdominal Distension present?	☐	☐
Is Abdominal Pain present?	☐	☐
Is Abdominal Rumbling present?	☐	☐
Is Abdominal Bloating present?	☐	☐
Child moved head away from the food?	☐	☐
Mouth clamped shut?	☐	☐
Teeth clenched?	☐	☐
Became agitated?	☐	☐
Spit out of the given drink?	☐	☐
Refused to swallow the given drink?	☐	☐

Study ID No.: | | | |

K	W	M	C	H

•

Hospital ID No

Is vomiting present?<br>If yes, frequency per day? ☐☐<br><br>Amount of vomiting per day ☐☐☐ gm	**No** ☐ **Yes** ☐
In mother's opinion, the child likes Fibersol-2/placebo on not?	☐

*** Children were considered as refusing intake if they moved their head away from the food, cried, clamped the mouth shut or clenched the teeth, or became agitated, spit out the food or refused to swallow. The amount of food ingested was calculated by subtracting the left-over from the offered amount. Pre-weighed napkins were provided; any food that was regurgitated, vomited or spilled was swabbed, the napkin weighed and subtracted from the weight of the amount offered. Using a 7-point Hedonic Scale in which each point (1 = disliked extremely, 2 = disliked moderately, 3 = disliked, 4 = neither disliked nor liked, 5 = liked slightly, 6 = liked moderately, 7 = liked extremely) was depicted by a facial drawing, we asked mothers to rate the food's color, flavor, mouth feel, and overall acceptability.

Stool Consistency:	☐

* Stool Consistency: 1 =Solid 2= Paste 3 =Loose/watery 4 =Loose with blood

Study ID No.: | | | |

K	W	M	C	H

Hospital ID No

STUDY DAY 5

**Date of Visit** (DD MON YY)	**Time of Visit** (HH: MM)
	:

STUDY PRODUCT ADMINISTRATION SCHEDULE

Dose	**Date** (DD MON YY)	**Time** (HH :MM)	Volume Administered (ml)	Volume Remaining (ml)
**1**		:		

ZINC ADMINISTRATION SCHEDULE *[Zinc to be administered with food at least 3 hours after test article administration]*

Dose	**Date** (DD MON YY)	**Time** (HH: MM)	Volume Administered (ml)	Volume Remaining (ml)
**1**		:	.	

STUDY PRODUCT ADMINISTRATION SCHEDULE

Dose	**Date** (DD MON YY)	**Time** (HH :MM)	Volume Administered (ml)	Volume Remaining (ml)
**2**		:		

ZINC ADMINISTRATION SCHEDULE *[Zinc to be administered with food at least 3 hours after test article administration]*

Dose	**Date** (DD MON YY)	**Time** (HH :MM)	Volume Administered (ml)	Volume Remaining (ml)
**2**		:	.	

Study ID No.: | | | |

K	W	M	C	H

Hospital ID No

VITAL SIGNS

**Variables Time measured (Measure at end of each 8-hours period)**	**Weight (Kg)** [morning only]	**Pulse Rate** (/Min	**Respiratory Rate** (/Min)	**Axillary Temp** (°C)	**B.P. Systolic** (mmHg) [morning only]	**B.P. Diastolic** (mmHg) [morning only]
1st 8-Hour Period						
2nd 8-Hour Period						
3rd 8-Hour Period						
Is any reading outside of normal range?	☐ No ☐ Yes	☐ No ☐ Yes	☐ No ☐ Yes	☐ No ☐ Yes	☐ No ☐ Yes	☐ No ☐ Yes
IF YES, is the deviation clinically Significant?*	☐ No ☐ Yes	☐ No ☐ Yes	☐ No ☐ Yes	☐ No ☐ Yes	☐ No ☐ Yes	☐ No ☐ Yes

**If Clinically Significant please fill up the Adverse Event Form as applicable.*

HISTORY ASSOCIATED WITH DIARRHOEA

**Details**	**NO**	**YES**
Is Abdominal Pain present?	☐	☐
Is Abdominal Distension present?	☐	☐
Is Fever present?	☐	☐
Is Lethargy present?	☐	☐
Is Vomiting present?<br>If Yes, number of vomiting per day ☐☐	☐	☐
Are other Abnormalities present?<br>If Yes, specify________________________________	☐	☐
History of mucus in stools	☐	☐
History of blood in stools	☐	☐
History of rice watery stools	☐	☐

Study ID No.: K W M C H

Hospital ID No

FOOD AND ORS INTAKE

**Date** (DD MON YY)	**Time of Intake 8-Hourly** (HH :MM)	**Food Intake** (g)	**ORS/Rice Saline Intake** (ml)	**Formula Milk** (ml)	**Was the subject breast fed**	**If Yes frequency**
	:				No / Yes	
	:				No / Yes	
	:				No / Yes	

STOOL AND URINE OUTPUT

**Date** (DD MON YY)	**Time of Measurement 8-Hourly** (HH :MM)	**Has watery stool been passed in this 8-hour period**	**Weight of stool** (g)	**Stool frequency**	**Stool consistency***	**Urine output (ml)**
	:	No Yes				
	:	No Yes				
	:	No Yes				

**Stool consistency (1= Solid, 2=Paste, 3 =Loose/watery, 4 =Loose with blood)*

STATUS OF OVERALL FOOD/ MILK INTAKE *(1 Hour before day ends)*

Mention food/Milk intake status of child as per scores provided below :<br>(1=Normal, 2=Mildly decreased, 3=Moderately decreased, 4=Severely decreased, 5=Refused to eat)	

STATUS OF DEHYDRATION *(1 Hour before day ends)*

Dehydration Status of child as per score provided below :<br>(0 =no signs of dehydration 1 =signs of Some dehydration 2 =signs of Severe dehydration*)<br>**If Dehydration is severe please exclude the subject from the study*	

ADVERSE EVENTS AND CONCOMITANT MEDICATIONS

Were there any changes in Adverse Events or Concomitant Medications since the last visit?<br>*If yes, please add new events/medications on appropriate log*	No	Yes

Study ID No.: | | | |

K	W	M	C	H

Hospital ID No

Reaction of Child after intake of Study Product (Fibersol-2/Placebo):
For 1st dose of the day 05
Amount of intake of Fibersol-2/Placebo in 30 mins: ☐ . ☐ gm

Description	No	Yes
Is Abdominal Distension present?	☐	☐
Is Abdominal Pain present?	☐	☐
Is Abdominal Rumbling present?	☐	☐
Is Abdominal Bloating present?	☐	☐
Child moved head away from the food?	☐	☐
Mouth clamped shut?	☐	☐
Teeth clenched?	☐	☐
Became agitated?	☐	☐
Spit out of the given drink?	☐	☐
Refused to swallow the given drink?	☐	☐

For 2nd dose of the day 05
Amount of intake of Fibersol-2/Placebo in 30 mins: ☐ . ☐ gm

Description	No	Yes
Is Abdominal Distension present?	☐	☐
Is Abdominal Pain present?	☐	☐
Is Abdominal Rumbling present?	☐	☐
Is Abdominal Bloating present?	☐	☐
Child moved head away from the food?	☐	☐
Mouth clamped shut?	☐	☐
Teeth clenched?	☐	☐
Became agitated?	☐	☐
Spit out of the given drink?	☐	☐
Refused to swallow the given drink?	☐	☐

Study ID No.: | | | |

K	W	M	C	H

Hospital ID No

Is vomiting present?<br>If yes, frequency per day? ☐☐<br><br>Amount of vomiting per day ☐☐☐ gm	**No** ☐ **Yes** ☐
In mother's opinion, the child likes Fibersol-2/placebo on not?	☐

*** Children were considered as refusing intake if they moved their head away from the food, cried, clamped the mouth shut or clenched the teeth, or became agitated, spit out the food or refused to swallow. The amount of food ingested was calculated by subtracting the left-over from the offered amount. Pre-weighed napkins were provided; any food that was regurgitated, vomited or spilled was swabbed, the napkin weighed and subtracted from the weight of the amount offered. Using a 7-point Hedonic Scale in which each point (1 = disliked extremely, 2 = disliked moderately, 3 = disliked, 4 = neither disliked nor liked, 5 = liked slightly, 6 = liked moderately, 7 = liked extremely) was depicted by a facial drawing, we asked mothers to rate the food's color, flavor, mouth feel, and overall acceptability.

Stool Consistency:	☐

* Stool Consistency: 1 =Solid 2 =Paste 3 =Loose/watery 4 =Loose with blood

Study ID No.: | | | |

K	W	M	C	H

Hospital ID No

STUDY DAY 6

Date of Visit (DD MON YY)	Time of Visit (HH: MM)
	:

STUDY PRODUCT ADMINISTRATION SCHEDULE

Dose	Date (DD MON YY)	Time (HH :MM)	Volume Administered (ml)	Volume Remaining (ml)
1		:		

ZINC ADMINISTRATION SCHEDULE *[Zinc to be administerd with food at least 3 hours after test article administration]*

Dose	Date (DD MON YY)	Time (HH: MM)	Volume Administered (ml)	Volume Remaining (ml)
1		:	.	

STUDY PRODUCT ADMINISTRATION SCHEDULE

Dose	Date (DD MON YY)	Time (HH :MM)	Volume Administered (ml)	Volume Remaining (ml)
2		:		

ZINC ADMINISTRATION SCHEDULE *[Zinc to be administered with food at least 3 hours after test article administration]*

Dose	Date (DD MON YY)	Time (HH :MM)	Volume Administered (ml)	Volume Remaining (ml)
2		:	.	

Study ID No.: [| |]
| K | W | M | C | H |

Hospital ID No [| | | | | | | | | |]

VITAL SIGNS

Variables Time measured (Measure at end of each 8-hours period)	Weight (Kg) [morning only]	Pulse Rate (/Min)	Respiratory Rate (/Min)	Axillary Temp (°C)	B.P. Systolic (mmHg) [morning only]	B.P. Diastolic (mmHg) [morning only]
1st 8-Hour Period						
2nd 8-Hour Period						
3rd 8-Hour Period						
Is any reading outside of normal range?	☐ No ☐ Yes	☐ No ☐ Yes	☐ No ☐ Yes	☐ No ☐ Yes	☐ No ☐ Yes	☐ No ☐ Yes
IF YES, is the deviation Cclinically Significant?*	☐ No ☐ Yes	☐ No ☐ Yes	☐ No ☐ Yes	☐ No ☐ Yes	☐ No ☐ Yes	☐ No ☐ Yes

**If Clinically Significant please fill up the Adverse Event Form as applicable.*

HISTORY ASSOCIATED WITH DIARRHOEA

Details	NO	YES
Is Abdominal Pain present?	☐	☐
Is Abdominal Distension present?	☐	☐
Is Fever present?	☐	☐
Is Lethargy present?	☐	☐
Is Vomiting present?<br>If Yes, number of vomiting per day [|]	☐	☐
Are other Abnormalities present?<br>If Yes, specify____________________	☐	☐
History of mucus in stools	☐	☐
History of blood in stools	☐	☐
History of rice watery stools	☐	☐

Study ID No.: K W M C H

Hospital ID No

FOOD AND ORS INTAKE

**Date** (DD MON YY)	**Time of Intake 8-Hourly** (HH :MM)	**Food Intake** (g)	**ORS/Rice Saline Intake** (ml)	**Formula Milk** (ml)	**Was the subject breast fed**	**If Yes frequency**
	:				No / Yes	
	:				No / Yes	
	:				No / Yes	

STOOL AND URINE OUTPUT

**Date** (DD MON YY)	**Time of Measurement 8-Hourly** (HH :MM)	**Has watery stool been passed in this 8-hour period**	**Weight of stool** (g)	**Stool frequency**	**Stool consistency***	**Urine output (ml)**
	:	No Yes				
	:	No Yes				
	:	No Yes				

**Stool consistency (1= Solid, 2=Paste, 3 =Loose/watery, 4 =Loose with blood)*

STATUS OF OVERALL FOOD/ MILK INTAKE *(1 Hour before day ends)*

Mention food/Milk intake status of child as per scores provided below :<br>(1=Normal, 2=Mildly decreased, 3=Moderately decreased, 4=Severely decreased, 5=Refused to eat)	

STATUS OF DEHYDRATION *(1 Hour before day ends)*

Dehydration Status of child as per score provided below :<br>(0 =no signs of dehydration 1 =signs of Some dehydration 2 =signs of Severe dehydration*)<br>**If Dehydration is severe please exclude the subject from the study*	

ADVERSE EVENTS AND CONCOMITANT MEDICATIONS

Were there any changes in Adverse Events or Concomitant Medications since the last visit?<br>*If yes, please add new events/medications on appropriate log*	No	Yes

Study ID No.: ☐☐☐

K	W	M	C	H

Hospital ID No ☐☐☐☐☐☐☐☐☐☐

Reaction of Child after intake of Study Product (Fibersol-2/Placebo):
For 1st dose of the day 06
Amount of intake of Fibersol-2/Placebo in 30 mins: ☐ . ☐ **gm**

Description	No	Yes
Is Abdominal Distension present?	☐	☐
Is Abdominal Pain present?	☐	☐
Is Abdominal Rumbling present?	☐	☐
Is Abdominal Bloating present?	☐	☐
Child moved head away from the food?	☐	☐
Mouth clamped shut?	☐	☐
Teeth clenched?	☐	☐
Became agitated?	☐	☐
Spit out of the given drink?	☐	☐
Refused to swallow the given drink?	☐	☐

For 2nd dose of the day 06
Amount of intake of Fibersol-2/Placebo in 30 mins: ☐ . ☐ **gm**

Description	No	Yes
Is Abdominal Distension present?	☐	☐
Is Abdominal Pain present?	☐	☐
Is Abdominal Rumbling present?	☐	☐
Is Abdominal Bloating present?	☐	☐
Child moved head away from the food?	☐	☐
Mouth clamped shut?	☐	☐
Teeth clenched?	☐	☐
Became agitated?	☐	☐
Spit out of the given drink?	☐	☐
Refused to swallow the given drink?	☐	☐

Study ID No.: | | | |

K	W	M	C	H

Hospital ID No | | | | | | | | | | |

Is vomiting present?<br>If yes, frequency per day? [ ][ ]<br><br>Amount of vomiting per day [ ][ ][ ] gm	**No** ☐ **Yes** ☐
In mother's opinion, the child likes Fibersol-2/placebo on not?	☐

*** Children were considered as refusing intake if they moved their head away from the food, cried, clamped the mouth shut or clenched the teeth, or became agitated, spit out the food or refused to swallow. The amount of food ingested was calculated by subtracting the left-over from the offered amount. Pre-weighed napkins were provided; any food that was regurgitated, vomited or spilled was swabbed, the napkin weighed and subtracted from the weight of the amount offered. Using a 7-point Hedonic Scale in which each point (1 = disliked extremely, 2 = disliked moderately, 3 = disliked, 4 = neither disliked nor liked, 5 = liked slightly, 6 = liked moderately, 7 = liked extremely) was depicted by a facial drawing, we asked mothers to rate the food's color, flavor, mouth feel, and overall acceptability.

Stool Consistency:	☐

* Stool Consistency: 1 =Solid 2 =Paste 3 =Loose/watery 4 =Loose with blood

Study ID No.: K W M C H

Hospital ID No

STUDY DAY 7

Date of Visit (DD MON YY)	Time of Visit (HH: MM)
	:

STUDY PRODUCT ADMINISTRATION SCHEDULE

Dose	Date (DD MON YY)	Time (HH :MM)	Volume Administered (ml)	Volume Remaining (ml)
**1**		:		

ZINC ADMINISTRATION SCHEDULE *[Zinc to be administered with food at least 3 hours after test article administration]*

Dose	Date (DD MON YY)	Time (HH: MM)	Volume Administered (ml)	Volume Remaining (ml)
**1**		:	.	

STUDY PRODUCT ADMINISTRATION SCHEDULE

Dose	Date (DD MON YY)	Time (HH :MM)	Volume Administered (ml)	Volume Remaining (ml)
**2**		:		

ZINC ADMINISTRATION SCHEDULE *[Zinc to be administered with food at least 3 hours after test article administration]*

Dose	Date (DD MON YY)	Time (HH :MM)	Volume Administered (ml)	Volume Remaining (ml)
**2**		:	.	

Study ID No.: | | | |

K	W	M	C	H

Hospital ID No

VITAL SIGNS

Variables Time measured (Measure at end of each 8-hours period)	Weight (Kg) [morning only]	Pulse Rate (/Min	Respiratory Rate (/Min)	Axillary Temp (°C)	B.P. Systolic (mmHg) [morning only]	B.P. Diastolic (mmHg) [morning only]
1st 8-Hour Period						
2nd 8-Hour Period						
3rd 8-Hour Period						
Is any reading outside of normal range?	☐ No ☐ Yes	☐ No ☐ Yes	☐ No ☐ Yes	☐ No ☐ Yes	☐ No ☐ Yes	☐ No ☐ Yes
IF YES, is the deviation Cclinically Significant?*	☐ No ☐ Yes	☐ No ☐ Yes	☐ No ☐ Yes	☐ No ☐ Yes	☐ No ☐ Yes	☐ No ☐ Yes

**If Clinically Significant please fill up the Adverse Event Form as applicable.*

HISTORY ASSOCIATED WITH DIARRHOEA

Details	NO	YES
Is Abdominal Pain present?	☐	☐
Is Abdominal Distension present?	☐	☐
Is Fever present?	☐	☐
Is Lethargy present?	☐	☐
Is Vomiting present?<br>If Yes, number of vomiting per day ☐☐	☐	☐
Are other Abnormalities present?<br>If Yes, specify______________________________	☐	☐
History of mucus in stools	☐	☐
History of blood in stools	☐	☐
History of rice watery stools	☐	☐

Study ID No.: ☐☐☐

K	W	M	C	H

Hospital ID No ☐☐☐☐☐☐☐☐☐☐

FOOD AND ORS INTAKE

Date (DD MON YY)	Time of Intake 8-Hourly (HH :MM)	Food Intake (g)	ORS/Rice Saline Intake (ml)	Formula Milk (ml)	Was the subject breast fed	If Yes frequency
☐☐ ☐☐☐ ☐☐	☐☐ : ☐☐	☐☐☐☐	☐☐☐☐	☐☐☐☐	☐ No ☐ Yes	☐☐
☐☐ ☐☐☐ ☐☐	☐☐ : ☐☐	☐☐☐☐	☐☐☐☐	☐☐☐☐	☐ No ☐ Yes	☐☐
☐☐ ☐☐☐ ☐☐	☐☐ : ☐☐	☐☐☐☐	☐☐☐☐	☐☐☐☐	☐ No ☐ Yes	☐☐

STOOL AND URINE OUTPUT

Date (DD MON YY)	Time of Measurement 8-Hourly (HH :MM)	Has watery stool been passed in this 8-hour period	Weight of stool (g)	Stool frequency	Stool consistency *	Urine output (ml)
☐☐ ☐☐☐ ☐☐	☐☐ : ☐☐	☐No ☐Yes	☐☐☐☐	☐☐	☐	☐☐☐☐
☐☐ ☐☐☐ ☐☐	☐☐ : ☐☐	☐No ☐Yes	☐☐☐☐	☐☐	☐	☐☐☐☐
☐☐ ☐☐☐ ☐☐	☐☐ : ☐☐	☐No ☐Yes	☐☐☐☐	☐☐	☐	☐☐☐☐

**Stool consistency (1= Solid, 2=Paste, 3 =Loose/watery, 4 =Loose with blood)*

STATUS OF OVERALL FOOD/ MILK INTAKE *(1 Hour before day ends)*

Mention food/Milk intake status of child as per scores provided below :<br>(1=Normal, 2=Mildly decreased, 3=Moderately decreased, 4=Severely decreased, 5=Refused to eat)	☐

STATUS OF DEHYDRATION *(1 Hour before day ends)*

Dehydration Status of child as per score provided below :<br>(0=no signs of dehydration 1 =signs of Some dehydration 2 =signs of Severe dehydration*)<br>**If Dehydration is severe please exclude the subject from the study*	☐

ADVERSE EVENTS AND CONCOMITANT MEDICATIONS

Were there any changes in Adverse Events or Concomitant Medications since the last visit?<br>*If yes, please add new events/medications on appropriate log*	☐ No	☐ Yes

Study ID No.: | | | |

K	W	M	C	H

Hospital ID No

Reaction of Child after intake of Study Product (Fibersol-2/Placebo):
For 1st dose of the day 07
Amount of intake of Fibersol-2/Placebo in 30 mins: ☐ . ☐ **gm**

Description	No	Yes
Is Abdominal Distension present?	☐	☐
Is Abdominal Pain present?	☐	☐
Is Abdominal Rumbling present?	☐	☐
Is Abdominal Bloating present?	☐	☐
Child moved head away from the food?	☐	☐
Mouth clamped shut?	☐	☐
Teeth clenched?	☐	☐
Became agitated?	☐	☐
Spit out of the given drink?	☐	☐
Refused to swallow the given drink?	☐	☐

For 2nd dose of the day 07
Amount of intake of Fibersol-2/Placebo in 30 mins: ☐ . ☐ **gm**

Description	No	Yes
Is Abdominal Distension present?	☐	☐
Is Abdominal Pain present?	☐	☐
Is Abdominal Rumbling present?	☐	☐
Is Abdominal Bloating present?	☐	☐
Child moved head away from the food?	☐	☐
Mouth clamped shut?	☐	☐
Teeth clenched?	☐	☐
Became agitated?	☐	☐
Spit out of the given drink?	☐	☐
Refused to swallow the given drink?	☐	☐

Study ID No.: | | | |

K	W	M	C	H

Hospital ID No

Is vomiting present?<br>If yes, frequency per day? ☐☐<br><br>Amount of vomiting per day ☐☐☐ gm	**No** ☐ **Yes** ☐
In mother's opinion, the child likes Fibersol-2/placebo on not?	☐

*** Children were considered as refusing intake if they moved their head away from the food, cried, clamped the mouth shut or clenched the teeth, or became agitated, spit out the food or refused to swallow. The amount of food ingested was calculated by subtracting the left-over from the offered amount. Pre-weighed napkins were provided; any food that was regurgitated, vomited or spilled was swabbed, the napkin weighed and subtracted from the weight of the amount offered. Using a 7-point Hedonic Scale in which each point (1 = disliked extremely, 2 = disliked moderately, 3 = disliked, 4 = neither disliked nor liked, 5 = liked slightly, 6 = liked moderately, 7 = liked extremely) was depicted by a facial drawing, we asked mothers to rate the food's color, flavor, mouth feel, and overall acceptability.

Stool Consistency:	☐

* Stool Consistency: 1 =Solid 2 =Paste 3 =Loose/watery 4 =Loose with blood

Study ID No.: ☐☐☐

K	W	M	C	H

Hospital ID No ☐☐☐☐☐☐☐☐☐☐

DAY 8: FOLLOW- UP QUESTIONNAIRES
Within 24 hours of discharge

Date of Follow up Visit: ☐☐ ☐☐☐ ☐☐
DD MON YY

Was telephonic contact made with the parent/legal guardian? ☐ No ☐ Yes

HISTORY ASSOCIATED WITH DIARRHOEA

Details	NO	YES
Is Abdominal Distension present?	☐	☐
Is Abdominal Pain present?	☐	☐
Is Abdominal Rumbling present?	☐	☐
Is Abdominal Bloating present?	☐	☐
Is Fever present?	☐	☐
Is Lethargy present?	☐	☐
Is Vomiting present?<br>If Yes, number of vomiting per day ☐☐	☐	☐
Are other Abnormalities present?<br>If Yes, specify________________________________	☐	☐
History of mucus in stools	☐	☐
History of blood in stools	☐	☐
History of rice watery stools	☐	☐

Study ID No.: | | | |

K	W	M	C	H

Hospital ID No

CRF 45

STATUS OF OVERALL FOOD / MILK INTAKE

☐ **(Milk 1-5 as per score provided below)**

Score	Food / Milk Intake	Frequency of Milk Intake per day
1	Normal	☐
2	Mildly decreased	
3	Moderately decreased	
4	Severely decreased	
5	Refused to eat	
In case of milk Intake, Please mention		☐ Breast fed ☐ Formula Milk ☐ Both

STATUS OF STOOL OUTPUT

Time Period	Has stool passed in this 8-hour period?	Stool frequency	Stool Consistency*	ORS No. of Cups
1st 8 Hours	☐ No<br>☐ Yes	☐☐	☐	☐☐
2nd 8 Hours	☐ No<br>☐ Yes	☐☐	☐	☐☐
3rd 8 Hours	☐ No<br>☐ Yes	☐☐	☐	☐☐

**Stool Consistency (1= Solid, 2 =Paste, 3 = Loose / watery, 4 = Loose with blood, 9 =Not applicable)*

STATUS OF DEHYDRATION *(1 Hour before day ends)*

Dehydration Status of child as per score provided below :<br>(0=no signs of dehydration 1 =signs of Some dehydration 2 =signs of Severe dehydration*)<br>**If Dehydration is severe please exclude the subject from the study*	☐

ADVERSE EVENTS CONCOMITANT MEDICATION

Were there any changes in Adverse Events or Concomitant Medications since the last visit?<br>*If yes, please add new events/medications on appropriate log*	☐ No	☐ Yes

Study ID No.: [ ][ ][ ]
| K | W | M | C | H |

Hospital ID No [ ][ ][ ][ ][ ][ ][ ][ ][ ][ ]

CRF 46

DAY 9: FOLLOW- UP QUESTIONNAIRES
Within 48 hours of discharge

Date of Follow up Visit : [ ][ ] DD [ ][ ][ ] MON [ ][ ] YY

Was telephonic contact made with the parent/legal guardian? ☐ No ☐ Yes

HISTORY ASSOCIATED WITH DIARRHOEA

	Mother's Opinion		Interviewer's Opinion	
**Details**	**NO**	**YES**	**NO**	**YES**
Is Abdominal Distension present?	☐	☐	☐	☐
Is Abdominal Pain present?	☐	☐	☐	☐
Is Abdominal Rumbling present?	☐	☐	☐	☐
Is Abdominal Bloating present?	☐	☐	☐	☐
Is Fever present?	☐	☐	☐	☐
Is Lethargy present?	☐	☐	☐	☐
Is Vomiting present?<br>If Yes, number of vomiting per day [ ][ ]	☐	☐	☐	☐
Are other Abnormalities present?<br>If Yes, specify_____________	☐	☐	☐	☐
History of mucus in stools	☐	☐	☐	☐

History of blood in stools	☐	☐	☐	☐
History of rice watery stools	☐	☐	☐	☐

STATUS OF OVERALL FOOD / MILK INTAKE

☐ **(Milk 1-5 as per score provided below)**

Score	Food / Milk Intake	Frequency of Milk Intake per day
1	Normal	☐
2	Mildly decreased	
3	Moderately decreased	
4	Severely decreased	
5	Refused to eat	

In case of milk Intake, Please mention ☐ Breast fed ☐ Formula Milk ☐ Both

STATUS OF STOOL OUTPUT

Time Period	Has stool passed in this 8-hour period?	Stool frequency	Stool Consistency*	ORS No. of Cups
1st 8 Hours	☐ No ☐ Yes	☐☐	☐	☐☐
2nd 8 Hours	☐ No ☐ Yes	☐☐	☐	☐☐
3rd 8 Hours	☐ No ☐ Yes	☐☐	☐	☐☐

**Stool Consistency (1=Solid, 2 =Paste, 3 =Loose / watery, 4 =Loose with blood, 9 =Not applicable)*

STATUS OF DEHYDRATION *(1 Hour before day ends)*

Dehydration Status of child as per score provided below :<br>(0 =no signs of dehydration 1 =signs of Some dehydration 2 =signs of Severe dehydration*)<br>**If Dehydration is severe please exclude the subject from the study*	☐

ADVERSE EVENTS CONCOMITANT MEDICATION

Were there any changes in Adverse Events or Concomitant Medications since the last visit?<br>*If yes, please add new events/medications on appropriate log*	☐ No	☐ Yes

Study ID No.: ☐☐☐

K	W	M	C	H

Hospital ID No ☐☐☐☐☐☐☐☐☐☐

CRF 48

DAY 10: FOLLOW- UP QUESTIONNAIRES
Within 72 hours of discharge

Date of Follow up Visit: ☐☐ ☐☐☐ ☐☐
DD MON YY

Was telephonic contact made with the parent/legal guardian? ☐ No ☐ Yes

HISTORY ASSOCIATED WITH DIARRHOEA

Details	NO	YES
Is Abdominal Distension present?	☐	☐
Is Abdominal Pain present?	☐	☐
Is Abdominal Rumbling present?	☐	☐
Is Abdominal Bloating present?	☐	☐
Is Fever present?	☐	☐
Is Lethargy present?	☐	☐
Is Vomiting present?<br>If Yes, number of vomiting per day ☐☐	☐	☐
Are other Abnormalities present?<br>If Yes, specify____	☐	☐
History of mucus in stools	☐	☐
History of blood in stools	☐	☐
History of rice watery stools	☐	☐

Study ID No.: | | | |

K	W	M	C	H

Hospital ID No | | | | | | | | | | |

CRF 49

STATUS OF OVERALL FOOD / MILK INTAKE

☐ **(Milk 1-5 as per score provided below)**

Score	Food / Milk Intake	Frequency of Milk Intake per day
1	Normal	☐
2	Mildly decreased	
3	Moderately decreased	
4	Severely decreased	
5	Refused to eat	
In case of milk Intake, Please mention		☐ Breast fed ☐ Formula Milk ☐ Both

STATUS OF STOOL OUTPUT

Time Period	Has stool passed in this 8-hour period?	Stool frequency	Stool Consistency*	ORS No. of Cups
1st 8-Hours	☐ No<br>☐ Yes	☐☐	☐	☐☐
2nd 8-Hours	☐ No<br>☐ Yes	☐☐	☐	☐☐
3rd 8-Hours	☐ No<br>☐ Yes	☐☐	☐	☐☐
*Stool Consistency (1=Solid, 2 =Paste, 3 =Loose / watery, 4 =Loose with blood, 9 =Not applicable)				

STATUS OF DEHYDRATION *(1 Hour before day ends)*

Dehydration Status of child as per score provided below :<br>(0 =signs of no dehydration 1 =signs of Some dehydration 2 =signs of Severe dehydration*)<br><br>**If Dehydration is severe please exclude the subject from the study*	☐

ADVERSE EVENTS CONCOMITANT MEDICATION

Were there any changes in Adverse Events or Concomitant Medications since the last visit?<br><br>*If yes, please add new events/medications on appropriate log*	☐ No	☐ Yes

Study ID No.: | | | |

K	W	M	C	H

Hospital ID No

CRF 50

DAY 11: FOLLOW- UP QUESTIONNAIRES
Within 96 hours of discharge

Date of Follow up Visit: ☐☐ ☐☐☐ ☐☐ ☐ ☐
DD MON YY

Was telephonic contact made with the parent/legal guardian? ☐ No ☐ Yes

HISTORY ASSOCIATED WITH DIARRHOEA

Details	NO	YES
Is Abdominal Distension present?	☐	☐
Is Abdominal Pain present?	☐	☐
Is Abdominal Rumbling present?	☐	☐
Is Abdominal Bloating present?	☐	☐
Is Fever present?	☐	☐
Is Lethargy present?	☐	☐
Is Vomiting present?<br>If Yes, number of vomiting per day ☐☐	☐	☐
Are other Abnormalities present?<br>If Yes, specify____	☐	☐
History of mucus in stools	☐	☐
History of blood in stools	☐	☐
History of rice watery stools	☐	☐

Study ID No.: | | | |

K	W	M	C	H

Hospital ID No

CRF 51

STATUS OF OVERALL FOOD / MILK INTAKE

☐ **(Milk 1-5 as per score provided below)**

Score	Food / Milk Intake	Frequency of Milk Intake per day
1	Normal	☐
2	Mildly decreased	
3	Moderately decreased	
4	Severely decreased	
5	Refused to eat	

In case of milk Intake, Please mention ☐ Breast fed ☐ Formula Milk ☐ Both

STATUS OF STOOL OUTPUT

Time Period	Has stool passed in this 8-hour period?	Stool frequency	Stool Consistency*	ORS No. of Cups
1st 8-Hours	☐ No ☐ Yes	☐☐	☐	☐☐
2nd 8-Hours	☐ No ☐ Yes	☐☐	☐	☐☐
3rd 8-Hours	☐ No ☐ Yes	☐☐	☐	☐☐

**Stool Consistency (1=Solid, 2 =Paste, 3 =Loose / watery, 4 =Loose with blood, 9 =Not applicable)*

STATUS OF DEHYDRATION *(1 Hour before day ends)*

Dehydration Status of child as per score provided below :<br>(0 =no signs of dehydration 1=signs of Some dehydration 2 =signs of Severe dehydration*)<br><br>**If Dehydration is severe please exclude the subject from the study*	☐

ADVERSE EVENTS CONCOMITANT MEDICATION

Were there any changes in Adverse Events or Concomitant Medications since the last visit?<br><br>*If yes, please add new events/medications on appropriate log*	☐ No	☐ Yes

Study ID No.: | | | |

K	W	M	C	H

Hospital ID No | | | | | | | | | | |

CRF 52

DAY 12: FOLLOW- UP QUESTIONNAIRES
Within 120 hours of discharge

Date of Follow up Visit: ☐☐ ☐☐☐ ☐☐
DD MON YY

Was telephonic contact made with the parent/legal guardian? ☐ No ☐ Yes

HISTORY ASSOCIATED WITH DIARRHOEA

Details	NO	YES
Is Abdominal Distension present?	☐	☐
Is Abdominal Pain present?	☐	☐
Is Abdominal Rumbling present?	☐	☐
Is Abdominal Bloating present?	☐	☐
Is Fever present?	☐	☐
Is Lethargy present?	☐	☐
Is Vomiting present?<br>If Yes, number of vomiting per day ☐☐	☐	☐
Are other Abnormalities present?<br>If Yes, specify____	☐	☐
History of mucus in stools	☐	☐
History of blood in stools	☐	☐
History of rice watery stools	☐	☐

Study ID No.: ☐☐☐

K	W	M	C	H

Hospital ID No ☐☐☐☐☐☐☐☐☐☐

CRF 53

STATUS OF OVERALL FOOD / MILK INTAKE

☐ **(Milk 1-5 as per score provided below)**

Score	Food / Milk Intake	Frequency of Milk Intake per day
1	Normal	☐
2	Mildly decreased	
3	Moderately decreased	
4	Severely decreased	
5	Refused to eat	

In case of milk Intake, Please mention ☐ Breast fed ☐ Formula Milk ☐ Both

STATUS OF STOOL OUTPUT

Time Period	Has stool passed in this 8-hour period?	Stool frequency	Stool Consistency*	ORS No. of Cups
1st 8-Hours	☐ No ☐ Yes	☐☐	☐	☐☐
2nd 8-Hours	☐ No ☐ Yes	☐☐	☐	☐☐
3rd 8-Hours	☐ No ☐ Yes	☐☐	☐	☐☐

**Stool Consistency (1=Solid, 2 =Paste, 3 =Loose / watery, 4 =Loose with blood, 9 =Not applicable)*

STATUS OF DEHYDRATION *(1 Hour before day ends)*

Dehydration Status of child as per score provided below :<br>(0 =signs of no dehydration 1 =signs of Some dehydration 2 =signs of Severe dehydration*)<br><br>**If Dehydration is severe please exclude the subject from the study*	☐

ADVERSE EVENTS CONCOMITANT MEDICATION

Were there any changes in Adverse Events or Concomitant Medications since the last visit?	☐ No	☐ Yes

Study ID No.:

K	W	M	C	H

Hospital ID No

If yes, please add new events/medications on appropriate log		

CRF 54

DAY 13: FOLLOW- UP QUESTIONNAIRES
Within 144 hours of discharge

Date of Follow up Visit: DD MON YY

Was telephonic contact made with the parent/legal guardian? ☐ No ☐ Yes

HISTORY ASSOCIATED WITH DIARRHOEA

Details	NO	YES
Is Abdominal Distension present?	☐	☐
Is Abdominal Pain present?	☐	☐
Is Abdominal Rumbling present?	☐	☐
Is Abdominal Bloating present?	☐	☐
Is Fever present?	☐	☐
Is Lethargy present?	☐	☐
Is Vomiting present?<br>If Yes, number of vomiting per day	☐	☐
Are other Abnormalities present?<br>If Yes, specify_______________________	☐	☐
History of mucus in stools	☐	☐

Study ID No.: | | | |

K	W	M	C	H

Hospital ID No

CRF 55

History of blood in stools	☐	☐
History of rice watery stools	☐	☐

STATUS OF OVERALL FOOD / MILK INTAKE

☐ **(Milk 1-5 as per score provided below)**

Score	Food / Milk Intake	Frequency of Milk Intake per day
1	Normal	☐
2	Mildly decreased	
3	Moderately decreased	
4	Severely decreased	
5	Refused to eat	
In case of milk Intake, Please mention		☐ Breast fed ☐ Formula Milk ☐ Both

STATUS OF STOOL OUTPUT

Time Period	Has stool passed in this 8-hour period?	Stool frequency	Stool Consistency*	ORS No. of Cups
1st 8-Hours	☐ No<br>☐ Yes	☐☐	☐	☐☐
2nd 8-Hours	☐ No<br>☐ Yes	☐☐	☐	☐☐
3rd 8-Hours	☐ No<br>☐ Yes	☐☐	☐	☐☐

**Stool Consistency (1= Solid, 2 =Paste, 3 =Loose / watery, 4 =Loose with blood, 9 =Not applicable)*

STATUS OF DEHYDRATION *(1 Hour before day ends)*

Dehydration Status of child as per score provided below :<br>(0 =no signs of dehydration 1 =signs of Some dehydration 2 =signs of Severe dehydration*)<br><br>**If Dehydration is severe please exclude the subject from the study*	☐

ADVERSE EVENTS CONCOMITANT MEDICATION

Were there any changes in Adverse Events or Concomitant Medications since the last visit?<br><br>*If yes, please add new events/medications on appropriate log*	☐ No	☐ Yes

Study ID No.:

K	W	M	C	H

Hospital ID No

CRF 56

DAY 14: FOLLOW- UP QUESTIONNAIRES
Within 168 hours of discharge

Date of Follow up Visit: ☐☐ ☐☐☐ ☐☐
DD MON YY

Was telephonic contact made with the parent/legal guardian? ☐ No ☐ Yes

VITAL SIGNS

**Variables Time measured (Measure at end of each 8-hours period)**	**Weight (Kg)** [morning only]	**Pulse Rate** (/Min)	**Respiratory Rate** (/Min)	**Axillary Temp** (°C)	**B.P. Systolic** (mmHg) [morning only]	**B.P. Diastolic** (mmHg) [morning only]
1st 8-Hour Period						
2nd 8-Hour Period						
3rd 8-Hour Period						
Is any reading outside of normal range?	☐ No ☐ Yes	☐ No ☐ Yes	☐ No ☐ Yes	☐ No ☐ Yes	☐ No ☐ Yes	☐ No ☐ Yes
IF YES, is the deviation Clinically Significant?*	☐ No ☐ Yes	☐ No ☐ Yes	☐ No ☐ Yes	☐ No ☐ Yes	☐ No ☐ Yes	☐ No ☐ Yes

**If Clinically Significant please fill up the Adverse Event Form as applicable.*

Study ID No.: | K | W | M | C | H |

Hospital ID No

CRF 57

HISTORY ASSOCIATED WITH DIARRHOEA

Details	NO	YES
Is Abdominal Distension present?	☐	☐
Is Abdominal Pain present?	☐	☐
Is Abdominal Rumbling present?	☐	☐
Is Abdominal Bloating present?	☐	☐
Is Fever present?	☐	☐
Is Lethargy present?	☐	☐
Is Vomiting present?<br>If Yes, number of vomiting per day	☐	☐
Are other Abnormalities present?<br>If Yes, specify____	☐	☐
History of mucus in stools	☐	☐
History of blood in stools	☐	☐
History of rice watery stools	☐	☐

STATUS OF OVERALL FOOD / MILK INTAKE

☐ **(Milk 1-5 as per score provided below)**

Score	Food / Milk Intake	Frequency of Milk Intake per day
1	Normal	
2	Mildly decreased	☐
3	Moderately decreased	

4	Severely decreased
5	Refused to eat

In case of milk Intake, Please mention ☐ Breast fed ☐ Formula Milk ☐ Both

STATUS OF STOOL OUTPUT

Time Period	Has stool passed in this 8 hour period?	Stool frequency	Stool Consistency*	ORS No. of Cups
1st 8-Hours	☐ No<br>☐ Yes	☐☐	☐	☐☐
2nd 8-Hours	☐ No<br>☐ Yes	☐☐	☐	☐☐
3rd 8-Hours	☐ No<br>☐ Yes	☐☐	☐	☐☐

**Stool Consistency (1=Solid, 2 =Paste, 3 =Loose / watery, 4 =Loose with blood, 9 =Not applicable)*

STATUS OF DEHYDRATION *(1 Hour before day ends)*

Dehydration Status of child as per score provided below :<br>(0=no signs of dehydration 1=signs of Some dehydration 2 =signs of Severe dehydration*)<br><br>**If Dehydration is severe please exclude the subject from the study*	☐

ADVERSE EVENTS CONCOMITANT MEDICATION

Were there any change in Adverse Events or Concomitant Medications since the last visit?<br><br>*If yes, please add new events/medications on appropriate log*	☐ No	☐ Yes

CESSATION OF DIARRHOEA

Did Diarrhoea cease during staying at hospital? ☐ No ☐ Yes

If Yes, Date of Cessation ☐☐ (DD) ☐☐☐ (MON) ☐☐ (YY) Time of Cessation : ☐☐ (HH) : ☐☐ (MM)

Study ID No.:

K	W	M	C	H

Hospital ID No

If No, Date of Cessation at home : Time of Cessation : :

DD MON YY HH MM

CRF 59

FINAL EVALUATION FROM (COMPLETION/DISCONTINUATION FORM)

Last day of Participation:

Study Day Date

DD MON YY

Last day of Test Article:

Study Day Date

DD MON YY

Discharge from Hospital:

Date :

Did child complete the study No Yes

If Yes, Date of study Completion

If child did not complete the study, what is the reason for termination*?(Check one box only)*

Consent withdrawn

Lost to follow-up *(appointment failure)*

Withdrawn for discovery of pre-existing violation of entry criteria *(specify)*_______________

Withdrawn for protocol noncompliance *(specify)*_______________________________________

Withdrawn due to lack of improvement requiring additional hospitalization *(Specify)*

____

Withdrawn for adverse sign of symptom.

If adverse sign ymptom is serious or death occurs, notify Medical Monitor and Sponsor immediately.

Death: Date of Death:

Cause(s) of Death: ____

Confirmed by Postmorterm: No Yes

Investigator's Signature ____________________ Date of Signature

Study ID No.: | | | |

K	W	M	C	H

Hospital ID No

PROTOCOL DEVIATION LOG

CRF Page No	Visit and CRF Date Item	Description of Deviation	Notified to EC
			☐ No<br>☐ Yes
			☐ No<br>☐ Yes
			☐ No<br>☐ Yes
			☐ No<br>☐ Yes
			☐ No<br>☐ Yes
			☐ No<br>☐ Yes
			☐ No<br>☐ Yes
			☐ No<br>☐ Yes
			☐ No<br>☐ Yes
			☐ No<br>☐ Yes

Study ID No.ঃ | | | |

K	W	M	C	H

Hospital ID No

CRF 1

ডায়রিয়া আক্রান্ত শিশুর রির্পোট ফর্ম

প্রটোকল নাম্বার : PR-১৬০৯১

প্রোটোকল টাইটেলঃ ১-৩ বছরের সুস্থ্য ও ডায়রিয়া আক্রান্ত শিশুদের ক্ষেত্রে ফাইবারসল-২ (প্রতিরোধী ম্যালটোডেক্সট্রিন) এর সহনীয়তা এবং গ্রহনযোগ্যতা নিরুপনের পরবর্তীতে রেনডোমাইজড ক্লিনিক্যাল ট্রায়ালের দ্বারা ফাইবারসল-২ এর কার্যক্ষমতা যাচাই।

প্রধান গবেষক
ডাঃ মোহাম্মদ জোবায়ের চিশতি, এমবিবিএস, এম মেড, পি এইচ ডি, আই সি ডি ডি আর বি

পৃষ্ঠপোষক
Matsutani Chemical Industry Company.

লিখিত সম্মতি পত্র

লিখিত সম্মতিপত্রের স্বাক্ষরের তারিখ: | | | দিন | | | | মাস | | | বছর

লিখিত সম্মতিপত্রের স্বাক্ষরের সময়: | | | ঘন্টা : | | | মিনিট

সম্মতিপত্রে দেওয়া স্বাক্ষর এই রোগীর সকল CRFএর জন্য:

আমি নিশ্চিত করছি যে এখানে সংগ্রহীত সকল তথ্য উপাত্ত গবেষণার প্রটোকল অনুসারে সংগ্রহ করা হয়েছে এবং লিখিত সম্মতিপত্র পিতামাতা অথবা অভিভাবক দিয়েছেন।

প্রধান গবেষকের স্বাক্ষর ঃ ____________________ **তারিখ ঃ** ____________________

প্রধান গবেষকের নাম (মুদ্রন করুন): ডাঃ মোহাম্মদ জোবায়ের চিশতি

Study ID No.ঃ | | | |

K	W	M	C	H

Hospital ID No

CRF 2

কেস রির্পোট ফর্মের (CRF) নির্দেশিকা ঃ

১.কালো কলম দ্বারা CRF পূরন করতে হবে। দৃঢ়ভাবে চাপ দিয়ে লিখুন ।

২.CRF.পূরন করতে বড় হাতের অক্ষর ব্যবহার করুন।

৩.সকল ঘর চিহ্নিত করতে (যেমন হ্যাঁ এবং না) X চিহ্ন এর সাহায্যে পূরন করতে হবে।

৪.২৪ ঘন্টায় ঘড়ির সময় ব্যবহার করতে হবে। যেমন রাত ৯ টা হলে ২১:০০ টা লিখতে হবে।

৫. আগে শূন্য বসাতে হবে যেখানে প্রযোজ্য যেমন রোগীর ওজন ১০.৫ কেজি হলে ০১০.৫ কেজি লিখতে হবে।

৬.কোন তথ্য জানা না থাকলে বক্সে UK লিখতে হবে :

U	K	D	E	C	1	4
দিন		মাস			বছর	

Time:

১	৯	U	K
ঘন্টা		মিনিট	

৭.যদি কোন ভুল হয়ে থাকে ?

- লেখাটি মুছে ফেলা যাবেনা ঘষাঘষি করে কাটা যাবেনা ।
- ফ্লুইড ব্যবহার করা যাবেনা।
- এক টানে কাটতে হবে। ~~56~~ 66SAS
- পূনরায় লিখতে হলে পরিস্কার ভাবে আগের লেখার কাছাকাছি লিখতে হবে ১০/০৪/১৩
- স্বাক্ষর এবং তারিখ দিতে হবে কোন সংশোধন করলে তার কাছাকাছি।

৮.প্রয়োজন নয় এমন তথ্য দেওয়া যাবেনা।

৯.দয়া করে নিশ্চিত করুন যে সমসাময়িক চিকিৎসা পরিস্কার ভাবে চিকিৎসা গত ভাষার সাথে মিলে যায় এবং লক্ষ্য করুন মূল ধারার কোন পরিবর্তন না হয় এবং মিলে যাওয়া ঘটনাকে প্রতিকূল ঘটনা হিসাবে বর্ণনা করুন।

১০.স্বাভাবিক পরিসরের বাইরের উচ্চতা ওজন রক্তচাপ অথবা ল্যাবরেটরী তথ্য দয়া করে নির্দেশ করুন যে মাপটি চিকিৎসাগতভাবে তাৎপর্যপূর্ন এবং অবশ্যই প্রতিকূল ঘটনা হিসাবে লিপিবন্ধ করতে হবে যদি কোন তথ্য চিকিৎসাগতভাবে তাৎপর্যপূন না হয়।

Study ID No.ঃ [ ][ ][ ]

K	W	M	C	H

Hospital ID No [ ][ ][ ][ ][ ][ ][ ][ ][ ][ ]

CRF 3

ভর্তি হওয়ার/অন্তর্ভূক্ত হওয়ার পূবের্র চিকিৎসা

[অনুগ্রহ পূর্বক গ্রাম/ নাম, অথবা যথাযথ উত্তর নিয়ে ফর্ম পূরন করুন]

পরিদর্শনের তারিখঃ: [ ][ ] [ ][ ][ ] [ ][ ] পরিদর্শনের সময়: [ ][ ] : [ ][ ]

দিন মাস বছর ঘন্টা মিনিট

রিপোর্টি করার সময় সাধারন পরীক্ষা :

অত্যাবশ্যকীয় লক্ষণ

সূচক	বয়স [মাস]	উচ্চতা [সে.মি]	ওজন (কেজি) [শুধু মাত্র সকালে]	নাড়ীর গতি (প্রতি মিনিটে)	শ্বাসের গতি (প্রতি মিনিট)	তাপমাত্রা (বগলের) (০সে.)	রক্তের চাপ.Systolic (mmHg) [শুধু মাত্র সকালে]	রক্তের চাপ Diastolic (mmHg) [শুধু মাত্র সকালে]
যদি কোন reading স্বাভাবিক পরিসীমা অতিক্রম করে?			☐ না ☐ হ্যাঁ	☐ না ☐ হ্যাঁ	☐ না ☐ হ্যাঁ	☐ না ☐ হ্যাঁ	☐ না ☐ হ্যাঁ	☐ না ☐ হ্যাঁ
যদি হ্যাঁ হয় তবে, reading clinically Significant কিনা?*			☐ না ☐ হ্যাঁ	☐ না ☐ হ্যাঁ	☐ না ☐ হ্যাঁ	☐ না ☐ হ্যাঁ	☐ না ☐ হ্যাঁ	☐ না ☐ হ্যাঁ

***যদি reading Clinically Significant হয় তবে প্রয়োজন হলে অনুগ্রহ করে Adverse Event Form পূরণ করুন]*

Study ID No.ঃ | | | |

K	W	M	C	H

Hospital ID No | | | | | | | | | | |

CRF 4

পানি শূন্যতার অবস্থা

নিচে উল্লেখিত তালিকা অনুযায়ী শিশুর পানি শূন্যতা :<br>(০=পানি শূন্যতা নাই ১=কিছুটা পানি শূন্যতা ২= মারাত্বক পানি শূন্যতা *)<br><br>* যদি মারাত্বক পানি শূন্যতা হয় তাহলে শিশুকে গবেষণা থেকে বাদ দিন	☐

I/V দ্বারা পূর্ণতা : ☐☐☐ মি .লি. খারার স্যালাইন দ্বারা পূর্ণতা: ☐☐☐ মি .লি.

জিংক এর পরিপূরক কিছু দেওয়া হয়েছিল কিনা ? ☐ না ☐ হ্যাঁ

জিংক দেওয়ার তারিখ : ☐☐ দিন ☐☐☐ মাস ☐☐ বছর

জিংক দেওয়ার সময়: ☐☐ ঘন্টা ☐☐ মিনিট

প্রস্তুতকৃত দুধ শিশুকে দেওয় হয়ে ছিল কিনা? ☐ না ☐ হ্যাঁ

যদি হ্যাঁ হয়,পরিমান ☐☐☐☐ মি.লি.

শিশুটি কি বুকের দুধ খায় ? ☐ না ☐ হ্যাঁ

কত বার ☐☐ প্রতিদিন

শিশুটি কি বর্তমানে শক্ত খাবার খায়? ☐ না ☐ হ্যাঁ

ডায়রিয়া ইতিহাস

ডায়রিয়া শুরু হওয়ার তারিখ: ☐☐ দিন ☐☐☐ মাস ☐☐ বছর ডায়রিয়া শুরু হওয়ার সময় : ☐☐ ঘন্টা : ☐☐ মিনিট

ডায়রিয়ার সময়কাল ☐☐☐ (ঘন্টায়)
[১ম ডোজ থেকে]

কতবার পায়খানা করেছে ☐☐ (প্রতিদিন)
[১ম ডো জের ২৪ ঘন্টা আগে]

মলের ধরণ *(একটি উওর প্রযোজ্য হবে)*	১ = শক্ত	২ = নরম	৩ = পাতলা/ পানির মত	৪ = রক্তযুক্ত পাতলা পায়খানা
			☐	

[নির্দেশিকা: ১. শক্ত =নিজস্ব ধরনের শক্ত; ২. নরম = নরম; ৩. পাতলা = পানির মত এবং পরিমানে অনেক বেশি; ৪. রক্তযুক্ত পাতলা পায়খানা = রক্ত এবং মিউকাস থাকবে সাথে মল থাকতে পারে না ও থাকতে পারে]

Study ID No.ঃ | | | |

K	W	M	C	H

Hospital ID No

CRF 5

ডায়রিয়া সাথে সম্পর্কিত লক্ষন সমূহ

বর্ণনা	মায়ের মতামত		ইন্টারভিউয়ারের মতামত	
	না	হ্যাঁ	না	হ্যাঁ
পেটে ব্যথা আছে কিনা?	☐	☐	☐	☐
পেটে ফাঁপা আছে কিনা?	☐	☐	☐	☐
জ্বর আছে কিনা?	☐	☐	☐	☐
শারিরীক দুর্বলতা আছে কিনা?	☐	☐	☐	☐
বমি আছে কিনা?<br>যদি হ্যাঁ হয়, প্রতি দিন কত বার বমি হয়? ☐☐	☐	☐	☐	☐
অন্যান্য অস্বাভাবিকতার উপস্থিতি<br>যদি হ্যাঁ হয়, নির্দিষ্ট করুন________________	☐	☐	☐	☐
পায়খানার সাথে মলের *উপস্থিতির ইতিহাস*	☐	☐	☐	☐
পায়খানার সাথে রক্তের *উপস্থিতির ইতিহাস*	☐	☐	☐	☐
চাল ধোয়া পানির মত পায়খানার ইতিহাস	☐	☐	☐	☐

সামগ্রিকভাবে খাবার এবং দুধ গ্রহনের পরিমান

খাওয়ার অবস্থা উল্লেখিত স্কোর অনুযায়ী<br>(১= স্বাভাবিক, ২= কিছুটা কম, ৩=মধ্যম কম, ৪= খুব বেশি কম,<br>৫= খাবার গ্রহনে অস্বীকৃতি)	☐

Study ID No.ঃ | | | |

K	W	M	C	H

.

Hospital ID No

CRF 6

তাৎপর্যপূর্ণ চিকিৎসাজনিত ইতিহাস (জন্ডিস, LRTI ইত্যাদি অন্তর্ভূক্তি):
যদি না হয়, তবে এখানে চেক করুন ☐

শরীর সর্ম্পকিত	না	হ্যাঁ	যদি হ্যাঁ হয়, রোগটি নির্দিষ্ট করুন(যদি জানা থাকে)
নাক,কান,গলা :	☐	☐	
চক্ষু সংক্রান্ত :	☐	☐	
স্নায়ুতান্ত্রিক :	☐	☐	
কার্ডিওভাসকুলার :	☐	☐	
শ্বসন :	☐	☐	
গ্যাস্ট্রো–ইনটেসস্টাইনাল :	☐	☐	
মূত্র সংক্রান্ত এবং যৌনাঙ্গ সংক্রান্ত:	☐	☐	
মাংসপেশী এবং কঙ্কাল :	☐	☐	
ডার্মাটোলজিক্যাল:	☐	☐	
হরমোন সংক্রান্ত	☐	☐	
রক্তসংক্রান্ত :	☐	☐	
যকৃত সংক্রান্ত :	☐	☐	
বিপাকীয়:	☐	☐	
এলার্জি এবং ঔষধ সংবেদনশীলতা:	☐	☐	

Study ID No.ঃ | | | |

K	W	M	C	H

Hospital ID No

CRF 7

অন্তভূর্ক্তি হওয়ার নিয়মাবলীর চেকলিষ্ট

অন্তর্ভুক্তির যোগ্যতা সমূহঃ

**অন্তভূর্ক্তি যোগ্যতার যে কোন একটি না হলে শিশুটি গবেষনায় অন্তভূর্ক্তি করা যাবেনা**	না	হ্যাঁ
১. ছেলে এবং মেয়ে শিশুর বয়স ১২-৩৬ মাসের মধ্যে কিনা?	☐	☐
২. ৭২ ঘন্টার কম স্থিতিকালের ডায়রিয়া জীবনের ঝুঁকি ছাড়া, যেমন- মারাত্বক শ্বসননালী সংক্রমন, রক্তে ব্যাক্টেরিয়ার সংক্রমন, রক্তে ইলেক্টলাইট এর অসামঞ্জস্যতা?	☐	☐
৩ অভিভাবক ৭২ ঘন্টার মধ্যে শিশুটিকে হাসপাতালে ভর্তি করিয়ে ছিলেন কিনা এবং ৭ দিন শেষে চুড়ান্ত পর্যবেক্ষনের জন্য হাসপাতালে নিয়ে আসতে রাজি আছে কিনা?	☐	☐
৪. অভিভাবকগন স্বেচ্ছায় মলের পরিমান, মলের ধরন, বমির পরিমান, খাবার গ্রহন সংক্রান্ত তথ্য পরিদর্শনের সময় জানাতে রাজি আছে কিনা?	☐	☐
৫. গবেষণার অন্তভুর্ক্তি হলে অবশ্যই লিখিত সম্মতি দিতে হবে?	☐	☐
৬. শিশুর শারিরীক পরীক্ষায় কোন অস্বাভাবিকতা নাই। (মারাত্বক অপুষ্টি, খিচুনি, ক্লিনিক্যাল সাসপিশিয়ান, স্যাপসিস, ম্যানেনজাইটিস, নিউমোনিয়া, দীর্ঘস্থায়ী পরিপাকতন্ত্রের রোগ, পরিপাকতন্ত্রের প্রদাহ অথবা অন্য কোন সংক্রমন যা একুয়েট ডায়রিয়ার সাথে সম্পর্কিত)	☐	☐

Study ID No.ঃ | | | |

K	W	M	C	H

Hospital ID No | | | | | | | | | | |

CRF 8

অন্তভুর্ক্তি না হওয়ার কারনগুলো

যদি অন্তভুর্ক্তি না হওয়ার কারনগুলোর মধ্যে একটি যদি হ্যাঁ হয় তবে শিশুদের গবেষণায় অন্তভুর্ক্তি করা হবে না	না	হ্যাঁ
১. সন্দেহজনক আমাশয় অথবা কোষ্ঠকাঠিন্য(সপ্তাহে তিন বারের কম পায়খানা হলে)	☐	☐
২. ডায়রিয়ার লক্ষন খুঁজে পেতে ৭২ ঘন্টার বেশি সময় লাগলে	☐	☐
৩. খুঁজে বের করার সময় মারাত্বক পানি শূন্যতা আছে কিনা	☐	☐
৪. মারাত্বক বমি করার জন্য ভর্তি সংক্রান্ত এবং অন্যান্য পরীক্ষা অসম্ভব কিনা	☐	☐
৫. মারাত্বক অপুষ্টি (WHO এর মান অনুযায়ী উচ্চতা ও ওজন যদি মিডিয়াম থেকে ৩ SD এর কম হয়)	☐	☐
৬. শিশুটি এন্টিবায়টিক বা সক্রিয়তা নষ্ঠকারী কোন ঔষধ গবেষণার ২ সপ্তাহে পূর্বে খেয়েছে কিনা	☐	☐
৭. অতি সংবেদনশীলতা বা দুধ বা ড়িএলার্জি আছে কিনা	☐	☐
৮. রোটা ভাইরাস এর প্রতিশেধক নিয়েছে কিনা	☐	☐
৯. শিশুটি বর্তমানে অন্য কোন গবেষণায় অন্তর্ভুক্ত আছে কিনা অথবা শিশুটি গবেষণায় অন্তর্ভুক্ত হলে গবেষণায় নিয়ম নীতি ভংগ হওয়ার সম্ভাবনা আছে কিনা	☐	☐

অন্তর্ভুক্তি

রোগীটি গবেষণার অন্তর্ভুক্ত হওয়ার যোগ্য কিনা ? ☐ না ☐ হ্যাঁ

যদি হ্যাঁ হয় :

রেনডোমাইজেশন এর তারিখ : ☐☐ দিন ☐☐☐ মাস ☐☐ বছর রেনডোমাইজেশন এর সময়: ☐☐ ঘন্টা ☐☐ মিনিট

রেনডোমাইজেশন এর নাম্বার : ☐☐☐

Study ID No.ঃ | | | |

K	W	M	C	H

Hospital ID No

CRF 9

গবেষণার ১ম দিন

পরিদর্শনের তারিখ (দিন মাস বছর)	পরিদর্শনের সময় (ঘন্টা : মিনিট)
	:

গবেষণার জিনিসপত্র/ ঔষধ দেওয়ার সূচী

ডোজ	তারিখ (দিন মাস বছর)	সময় (ঘন্টা : মিনিট)	দেওয়ার পরিমান (মিলি)	দেওয়ার বাকী আছে (মিলি)
১		:		

জিংক দেওয়ার সূচী *[test article administration এর কমপক্ষে ৩ ঘন্টা পর খাবারের সাথে জিংক দিতে হবে]*

ডোজ	তারিখ (দিন মাস বছর)	সময় (ঘন্টা : মিনিট)	দেওয়ার পরিমান (মিলি)	দেওয়ার বাকী আছে (মিলি)
১		:	.	

গবেষণার জিনিসপত্র/ ঔষধ দেওয়ার সূচী

ডোজ	তারিখ (দিন মাস বছর)	সময় (ঘন্টা : মিনিট)	দেওয়ার পরিমান (মিলি)	দেওয়ার বাকী আছে (মিলি)
২		:		

জিংক দেওয়ার সূচী *[test article administration এর কমপক্ষে ৩ ঘন্টা পর খাবারের সাথে জিংক দিতে হবে]*

ডোজ	তারিখ (দিন মাস বছর)	সময় (ঘন্টা : মিনিট)	দেওয়ার পরিমান (মিলি)	দেওয়ার বাকী আছে (মিলি)
২		:	.	

Study ID No.ঃ | | | |

K	W	M	C	H

Hospital ID No

CRF 10

অত্যাবশ্যকীয় লক্ষণ

**Variables** সময় গণনা (প্রতি ৮ ঘন্টা পর পর গণনা করুন)	**ওজন (কেজি)** [শুধু মাত্র সকালে]	নাড়ীর গতি (প্রতি মিনিটে)	**শ্বাসের গতি**(প্রতি মিনিট)	**তাপমাত্রা (বগলের)** ($^{0সে.}$)	**ব্লাড প্রেসার. Systolic** (mmHg) [শুধু মাত্র সকালে]	**ব্লাড প্রেসার. Diastolic** (mmHg) [শুধু মাত্র সকালে]
১ম আট ঘন্টা সময়						
২য় আট ঘন্টা সময়						
৩য় আট ঘন্টা সময়						
যদি কোন reading স্বাভাবিক পরিসীমা অতিক্রম করে?	☐ না ☐ হ্যাঁ	☐ না ☐ হ্যাঁ	☐ না ☐ হ্যাঁ	☐ না ☐ হ্যাঁ	☐ না ☐ হ্যাঁ	☐ না ☐ হ্যাঁ
যদি হ্যাঁ হয় তবে, reading clinically Significant কিনা?*	☐ না ☐ হ্যাঁ	☐ না ☐ হ্যাঁ	☐ না ☐ হ্যাঁ	☐ না ☐ হ্যাঁ	☐ না ☐ হ্যাঁ	☐ না ☐ হ্যাঁ
**যদি reading Clinically Significant হয় তবে প্রয়োজন হলে অনুগ্রহ করে Adverse Event Form পূরণ করুন]*						

ডায়রিয়া সাথে সম্পর্কিত লক্ষন সমূহ

বর্ণনা	না	হ্যাঁ
পেটে ব্যাথা আছে কিনা?	☐	☐
পেটে ফাঁপা আছে কিনা?	☐	☐
জ্বর আছে কিনা?	☐	☐
শারিরীক দুর্বলতা আছে কিনা?	☐	☐
বমি আছে কিনা? যদি হ্যাঁ হয়, প্রতি দিন কত বার বমি হয় ? ☐☐	☐	☐
অন্যান্য অস্বাভাবিকতা উপস্থিতি যদি হ্যাঁ হয়, নির্দিষ্ট করুন________________	☐	☐
পায়খানার সাথে মলের *উপস্থিতির ইতিহাস*	☐	☐
পায়খানার সাথে রক্তের *উপস্থিতির ইতিহাস*	☐	☐
চাল ধোয়া পানির মত পায়খানার ইতিহাস	☐	☐

Study ID No.ঃ | | | |

K	W	M	C	H

Hospital ID No

CRF 11

খাবার এবং স্যালাইন গ্রহন

তারিখ (দিন মাস বছর)	গ্রহনের ৮ ঘন্টা সময় (ঘন্টা মিনিট)	খাবার গ্রহন (গ্রাম)	ORS/Rice-স্যালাইন গ্রহন (মি.লি)	তৈরি করা দুধ (মি.লি)	(শিশুটি) বুকের দুধ খায় কিনা	যদি হ্যাঁ হয় কত বার
	:				না / হ্যাঁ	
	:				না / হ্যাঁ	
	:				না / হ্যাঁ	

প্রস্রাব এবং মল ত্যাগের পরিমান

তারিখ (দিন মাস বছর)	পরিমাপ ৮ ঘন্টায় (ঘন্টা/ মিনিট)	এই ৮ ঘন্টায় পানির মত পায়খানা করেছে	পায়খানার পরিমান (গ্রাম)	পায়খানা কত বার	পায়খানার ধরণ*	প্রস্রাবের পরিমান (মি.লি)
	:	না হ্যাঁ				
	:	না হ্যাঁ				
	:	না হ্যাঁ				

* **পায়খানার ধরণ** *(১= শক্ত, ২= নরম, ৩ = পাতলা / পানির মত, ৪ = পাতলা পায়খানার সাথে রক্ত)*

সামগ্রিকভাবে খাবার এবং দুধ গ্রহনের পরিমান *(দিন শেষ হওয়ার ১ ঘন্টা পূর্বে)*

শশিুর খাবার / দুধ খাওয়ার অবস্থা নচিে উল্লখেতি স্কোর অনুযায়ী<br>(১=স্বাভাবিক, ২=কিছুটা কম, ৩=মধ্যম কম, ৪=খুব বেশি কম, ৫=খাবার গ্রহনে অস্বীকৃতি)	

পানি শূন্যতার পরিমান *(দিন শেষ হওয়ার ১ ঘন্টা পূর্বে)*

নিচে উল্লেখিত তালিকা অনুযায়ী শিশুর পানি শূন্যতা :<br>(০=পানি শূন্যতা নাই ১=কিছুটা পানি শূন্যতা ২= মারাত্বক পানি শূন্যতা *)<br>* যদি মারাত্বক পানি শূন্যতা হয় তাহলে শিশুকে গবেষণা থেকে বাদ দিন	

প্রতিকূল ঘটনায় প্রযোজ্য ঔষধ সমূহ

গত পরিদর্শনের সময় দেওয়া ঔষধ সমূহ পরিবর্তনের প্রয়োজন কিনা ?<br>*যদি হ্যাঁ হয় তবে প্রযোজ্য লিষ্ট থেকে নতুন ঔষধ যোগ করুন*	না	হ্যাঁ

Study ID No.ঃ | | | |

K	W	M	C	H

.

Hospital ID No

CRF 12

পানীয় (ফাইবারসল-২/প্লাসিবু) গ্রহনের পর শিশুর প্রতিক্রিয়া :

১ম দিনের ১ম ডোজ

৩০ মিনিটে ফাইবারসল-২/প্লাসিবু গ্রহনের পরিমান : ☐.☐ গ্রাম

বর্ণনা	না	হ্যাঁ
পেট ফুলে গিয়েছে কিনা?	☐	☐
পেটে ব্যাথা আছে কিনা?	☐	☐
পেট ডাকে কিনা?	☐	☐
পেটে ফাঁপা আছে কিনা?	☐	☐
শিশুটি খাবার থেকে মুখ ঘুরিয়ে নেয় কিনা ?	☐	☐
শিশুটি মুখ বন্ধ করে রাখে কিনা ?	☐	☐
শিশুটি মাড়ি কামড়ে রাখে কিনা ?	☐	☐
শিশুটি বিরক্ত হয়েছে কিনা ?	☐	☐
শিশুটি মুখ থেকে **ফাইবারসল-২/প্লাসিবু** ফেলে দেয় কিনা ?	☐	☐
শিশুটি **ফাইবারসল-২/প্লাসিবু** গিলতে অস্বীকার করে কিনা ?	☐	☐

১ম দিনের ২য় ডোজ

৩০ মিনিটে ফাইবারসল-২/প্লাসিবু গ্রহনের পরিমান : ☐.☐ গ্রাম

বর্ণনা	না	হ্যাঁ
পেট ফুলে গিয়েছে কিনা?	☐	☐
পেটে ব্যাথা আছে কিনা?	☐	☐
পেট ডাকে কিনা?	☐	☐
পেটে ফাঁপা আছে কিনা?	☐	☐
শিশুটি খাবার থেকে মুখ ঘুরিয়ে নেয় কিনা ?	☐	☐
শিশুটি মুখ বন্ধ করে রাখে কিনা ?	☐	☐
শিশুটি মাড়ি কামড়ে রাখে কিনা ?	☐	☐
	☐	☐

Study ID No.ঃ | | | |

K	W	M	C	H

Hospital ID No

CRF 13

শিশুটি বিরক্ত হয়েছে কিনা ?		
শিশুটি মুখ থেকে **ফাইবারসল-২/প্লাসিবু** ফেলে দেয় কিনা ?	☐	☐
শিশুটি **ফাইবারসল-২/প্লাসিবু** গিলতে অস্বীকার করে কিনা ?	☐	☐

বমি আছে কিনা?<br>যদি হ্যাঁ হয়, প্রতি দিন কত বার বমি হয় ? ☐☐ বার<br>প্রতিদিন কি পরিমান বমি করেছে ☐☐☐ গ্রাম	না ☐ হ্যাঁ ☐
মায়ের দৃষ্টিতে শিশুটি **ফাইবারসল-২/প্লাসিবু পছন্দ করেছে কিনা ?**	☐

*** শিশুটি পানীয় নিতে অস্বীকার করেছে বলে বিবেচনা করবো যদি সে পানীয় থেকে মুখ ফিরিয়ে নেয়, কান্নাকাটি করে ,মুখ জোর করে বন্ধ করে রাখে অথবা দাত খিচিয়ে রাখে অথবা বিরক্ত হয়ে যায়, মুখ থেকে ফেলে দেয় কিংবা গিলতে অস্বীকার করে। আগে থেকে ওজন নেয়া নেপকিন সরবরাহ করা হবে। কোনো খাবার যদি উগড়ে ফেলে বা বমি করে ফেলে দেয়া হয়, তা মুছে নেপকিনের ওজন নেয়া হবে এবং সরবরাহকৃত পরিমান থেকে বাদ দেয়া হবে। ৭ পয়েন্টের হেডোনিক স্কেল ব্যবহার করা হবে যেখানে প্রতিটি পয়েন্ট মুখভঙ্গির অঙ্কন দিয়ে নির্ধারন করা হবে। আমরা মায়েদের থেকে খাবারের রং,গন্ধ, স্বাদ এবং সার্বিক গ্রহনযোগ্যতা সর্ম্পকে মতামত চেয়েছি। (১=খুবই অপছন্দ, ২=কিছুটা অপছন্দ, ৩= অপছন্দ, ৪= পছন্দ-অপছন্দ কিছুই না, ৫=কিছুটা পছন্দ, ৬=মোটামুটি পছন্দ, ৭= খুবই পছন্দ)।

পায়খানার ধরণ :	☐

* **পায়খানার ধরণ** *(১= শক্ত, ২= নরম, ৩ = পাতলা / পানির মত, ৪ = পাতলা পায়খানার সাথে রক্ত)*

Study ID No.: | | | |

K	W	M	C	H

Hospital ID No

CRF 14

গবেষণার ২য় দিন

পরিদর্শনের তারিখ (দিন মাস বছর)	পরিদর্শনের সময় (ঘন্টা : মিনিট)
	:

গবেষণার জিনিসপত্র/ ঔষধ দেওয়ার সূচী

ডোজ	তারিখ (দিন মাস বছর)	সময় (ঘন্টা : মিনিট)	দেওয়ার পরিমান (মিলি)	দেওয়ার বাকী আছে (মিলি)
১		:		

জিংক দেওয়ার সূচী [*test article administration এর কমপক্ষে ৩ ঘন্টা পর খাবারের সাথে জিংক দিতে হবে*]

ডোজ	তারিখ (দিন মাস বছর)	সময় (ঘন্টা : মিনিট)	দেওয়ার পরিমান (মিলি)	দেওয়ার বাকী আছে (মিলি)
১		:	.	

গবেষণার জিনিসপত্র/ ঔষধ দেওয়ার সূচী

ডোজ	তারিখ (দিন মাস বছর)	সময় (ঘন্টা : মিনিট)	দেওয়ার পরিমান (মিলি)	দেওয়ার বাকী আছে (মিলি)
২		:		

জিংক দেওয়ার সূচী [*test article administration এর কমপক্ষে ৩ ঘন্টা পর খাবারের সাথে জিংক দিতে হবে*]

ডোজ	তারিখ (দিন মাস বছর)	সময় (ঘন্টা : মিনিট)	দেওয়ার পরিমান (মিলি)	দেওয়ার বাকী আছে (মিলি)
২		:	.	

Study ID No.ঃ | | | |

K	W	M	C	H

Hospital ID No

CRF 15

অত্যাবশ্যকীয় লক্ষণ

Variables সময় গণনা (প্রতি ৮ ঘন্টা পর পর গণনা করুন)	ওজন (কেজি) [শুধু মাত্র সকালে]	নাড়ীর গতি (প্রতি মিনিটে)	শ্বাসের গতি(প্রতি মিনিট)	তাপমাত্রা (বগলের) (^{0}সে.)	ব্লাড প্রেসার. Systolic (mmHg) [শুধু মাত্র সকালে]	ব্লাড প্রেসার. Diastolic (mmHg) [শুধু মাত্র সকালে]
১ম আট ঘন্টা সময়						
২য় আট ঘন্টা সময়						
৩য় আট ঘন্টা সময়						
যদি কোন reading স্বাভাবিক পরিসীমা অতিক্রম করে?	☐ না ☐ হ্যাঁ	☐ না ☐ হ্যাঁ	☐ না ☐ হ্যাঁ	☐ না ☐ হ্যাঁ	☐ না ☐ হ্যাঁ	☐ না ☐ হ্যাঁ
যদি হ্যাঁ হয় তবে, reading clinically Significant কিনা?*	☐ না ☐ হ্যাঁ	☐ না ☐ হ্যাঁ	☐ না ☐ হ্যাঁ	☐ না ☐ হ্যাঁ	☐ না ☐ হ্যাঁ	☐ না ☐ হ্যাঁ

**যদি reading Clinically Significant হয় তবে প্রয়োজন হলে অনুগ্রহ করে Adverse Event Form পূরণ করুন]*

ডায়রিয়া সাথে সম্পর্কিত লক্ষন সমূহ

বর্ণনা	না	হ্যাঁ
পেটে ব্যাথা আছে কিনা?	☐	☐
পেটে ফাঁপা আছে কিনা?	☐	☐
জ্বর আছে কিনা?	☐	☐
শারিরীক দুর্বলতা আছে কিনা?	☐	☐
বমি আছে কিনা?<br>যদি হ্যাঁ হয়, প্রতি দিন কত বার বমি হয় ? ☐☐	☐	☐
অন্যান্য অস্বাভাবিকতা উপস্থিতি যদি হ্যাঁ হয়, নির্দিষ্ট করুন______________________________	☐	☐
পায়খানার সাথে মলের *উপস্থিতির ইতিহাস*	☐	☐
পায়খানার সাথে রক্তের *উপস্থিতির ইতিহাস*	☐	☐
চাল ধোয়া পানির মত পায়খানার ইতিহাস	☐	☐

Study ID No.ঃ | | | |

K	W	M	C	H

.

Hospital ID No

CRF 16

খাবার এবং স্যালাইন গ্রহন

তারিখ (দিন মাস বছর)	গ্রহনের ৮ ঘন্টা সময় (ঘন্টা মিনিট)	খাবার গ্রহন (গ্রাম)	ORS/Rice- স্যালাইন গ্রহন (মি.লি)	তৈরি করা দুধ (মি.লি)	(শিশুটি) বুকের দুধ খায় কিনা	যদি হ্যাঁ হয় কত বার
	:				না হ্যাঁ	
	:				না হ্যাঁ	
	:				না হ্যাঁ	

প্রস্রাব এবং মল ত্যাগের পরিমান

তারিখ (দিন মাস বছর)	পরিমাপ ৮ ঘন্টায় (ঘন্টা/ মিনিট)	এই ৮ ঘন্টায় পানির মত পায়খানা করেছে	পায়খানার পরিমান (গ্রাম)	পায়খানা কত বার	পায়খানার ধরণ*	প্রস্রাবের পরিমান (মি.লি)
	:	না হ্যাঁ				
	:	না হ্যাঁ				
	:	না হ্যাঁ				

* **পায়খানার ধরণ** *(১= শক্ত, ২= নরম, ৩ = পাতলা / পানির মত, ৪ = পাতলা পায়খানার সাথে রক্ত)*

সামগ্রিকভাবে খাবার এবং দুধ গ্রহনের পরিমান *(দিন শেষ হওয়ার ১ ঘন্টা পূর্বে)*

শশিুর খাবার / দুধ খাওয়ার অবস্থা নচিে উল্লখেতি স্কোর অনুযায়ী<br>(১=স্বাভাবিক, ২=কিছুটা কম, ৩=মধ্যম কম, ৪=খুব বেশি কম, ৫=খাবার গ্রহনে অস্বীকৃতি)	

পানি শূন্যতার পরিমান *(দিন শেষ হওয়ার ১ ঘন্টা পূর্বে)*

নিচে উল্লেখিত তালিকা অনুযায়ী শিশুর পানি শূন্যতা :<br>(০=পানি শূন্যতা নাই ১=কিছুটা পানি শূন্যতা ২= মারাত্বক পানি শূন্যতা *)<br>* যদি মারাত্বক পানি শূন্যতা হয় তাহলে শিশুকে গবেষণা থেকে বাদ দিন	

প্রতিকূল ঘটনায় প্রযোজ্য ঔষধ সমূহ

গত পরিদর্শনের সময় দেওয়া ঔষধ সমূহ পরিবর্তনের প্রয়োজন কিনা ?<br>*যদি হ্যাঁ হয় তবে প্রযোজ্য লিষ্ট থেকে নতুন ঔষধ যোগ করুন*	না	হ্যাঁ

Study ID No.ঃ [ ][ ][ ]

K	W	M	C	H

Hospital ID No

CRF 17

পানীয় (ফাইবারসল-২/প্লাসিবু) গ্রহনের পর শিশুর প্রতিক্রিয়া :

২য় দিনের ১ম ডোজ

৩০ মিনিটে ফাইবারসল-২/প্লাসিবু গ্রহনের পরিমান : [ ].[ ] গ্রাম

বর্ণনা	না	হ্যাঁ
পেট ফুলে গিয়েছে কিনা?		
পেটে ব্যাথা আছে কিনা?		
পেট ডাকে কিনা?		
পেটে ফাঁপা আছে কিনা?		
শিশুটি খাবার থেকে মুখ ঘুরিয়ে নেয় কিনা ?		
শিশুটি মুখ বন্ধ করে রাখে কিনা ?		
শিশুটি মাড়ি কামড়ে রাখে কিনা ?		
শিশুটি বিরক্ত হয়েছে কিনা ?		
শিশুটি মুখ থেকে **ফাইবারসল-২/প্লাসিবু** ফেলে দেয় কিনা ?		
শিশুটি **ফাইবারসল-২/প্লাসিবু** গিলতে অস্বীকার করে কিনা ?		

২য় দিনের ২য় ডোজ

৩০ মিনিটে ফাইবারসল-২/প্লাসিবু গ্রহনের পরিমান : [ ].[ ] গ্রাম

বর্ণনা	না	হ্যাঁ
পেট ফুলে গিয়েছে কিনা?		
পেটে ব্যাথা আছে কিনা?		
পেট ডাকে কিনা?		
পেটে ফাঁপা আছে কিনা?		
শিশুটি খাবার থেকে মুখ ঘুরিয়ে নেয় কিনা ?		
শিশুটি মুখ বন্ধ করে রাখে কিনা ?		
শিশুটি মাড়ি কামড়ে রাখে কিনা ?		

Study ID No.ঃ | | | |

K	W	M	C	H

Hospital ID No | | | | | | | | | | |

CRF 18

শিশুটি বিরক্ত হয়েছে কিনা ?		
শিশুটি মুখ থেকে **ফাইবারসল-২/প্লাসিবু** ফেলে দেয় কিনা ?	☐	☐
শিশুটি **ফাইবারসল-২/প্লাসিবু** গিলতে অস্বীকার করে কিনা ?	☐	☐

বমি আছে কিনা?<br>যদি হ্যাঁ হয়, প্রতি দিন কত বার বমি হয় ? ☐☐ বার<br>প্রতিদিন কি পরিমান বমি করেছে ☐☐☐ গ্রাম	না ☐ হ্যাঁ ☐
মায়ের দৃষ্টিতে শিশুটি **ফাইবারসল-২/প্লাসিবু পছন্দ করেছে কিনা ?**	☐

*** শিশুটি পানীয় নিতে অস্বীকার করেছে বলে বিবেচনা করবো যদি সে পানীয় থেকে মুখ ফিরিয়ে নেয়, কান্নাকাটি করে ,মুখ জোর করে বন্ধ করে রাখে অথবা দাত খিচিয়ে রাখে অথবা বিরক্ত হয়ে যায়, মুখ থেকে ফেলে দেয় কিংবা গিলতে অস্বীকার করে। আগে থেকে ওজন নেয়া নেপকিন সরবরাহ করা হবে। কোনো খাবার যদি উগড়ে ফেলে বা বমি করে ফেলে দেয়া হয়, তা মুছে নেপকিনের ওজন নেয়া হবে এবং সরবরাহকৃত পরিমান থেকে বাদ দেয়া হবে। ৭ পয়েন্টের হেডোনিক স্কেল ব্যবহার করা হবে যেখানে প্রতিটি পয়েন্ট মুখভঙ্গির অঙ্কন দিয়ে নির্ধারন করা হবে। আমরা মায়েদের থেকে খাবারের রং,গন্ধ, স্বাদ এবং সার্বিক গ্রহনযোগ্যতা সর্ম্পকে মতামত চেয়েছি। (১=খুবই অপছন্দ, ২=কিছুটা অপছন্দ, ৩= অপছন্দ, ৪= পছন্দ-অপছন্দ কিছুই না, ৫=কিছুটা পছন্দ, ৬=মোটামুটি পছন্দ, ৭= খুবই পছন্দ)।

পায়খানার ধরণ :	☐

* **পায়খানার ধরণ** *(১= শক্ত, ২= নরম, ৩ = পাতলা / পানির মত, ৪ = পাতলা পায়খানার সাথে রক্ত)*

Study ID No.ঃ | | | |

K	W	M	C	H

Hospital ID No

CRF 19

গবেষণার ৩য় দিন

পরিদর্শনের তারিখ (দিন মাস বছর)	পরিদর্শনের সময় (ঘন্টা : মিনিট)
	:

গবেষণার জিনিসপত্র/ ঔষধ দেওয়ার সূচী

ডোজ	তারিখ (দিন মাস বছর)	সময় (ঘন্টা : মিনিট)	দেওয়ার পরিমান (মিলি)	দেওয়ার বাকী আছে (মিলি)
১		:		

জিংক দেওয়ার সূচী *[test article administration এর কমপক্ষে ৩ ঘন্টা পর খাবারের সাথে জিংক দিতে হবে]*

ডোজ	তারিখ (দিন মাস বছর)	সময় (ঘন্টা : মিনিট)	দেওয়ার পরিমান (মিলি)	দেওয়ার বাকী আছে (মিলি)
১		:	.	

গবেষণার জিনিসপত্র/ ঔষধ দেওয়ার সূচী

ডোজ	তারিখ (দিন মাস বছর)	সময় (ঘন্টা : মিনিট)	দেওয়ার পরিমান (মিলি)	দেওয়ার বাকী আছে (মিলি)
২		:		

জিংক দেওয়ার সূচী *[test article administration এর কমপক্ষে ৩ ঘন্টা পর খাবারের সাথে জিংক দিতে হবে]*

ডোজ	তারিখ (দিন মাস বছর)	সময় (ঘন্টা : মিনিট)	দেওয়ার পরিমান (মিলি)	দেওয়ার বাকী আছে (মিলি)

Study ID No.ঃ | | | |

K	W	M	C	H

Hospital ID No

২		:	.	

CRF 20

অত্যাবশ্যকীয় লক্ষণ

**Variables** সময় গণনা (প্রতি ৮ ঘন্টা পর পর গণনা করুন)	ওজন (কেজি) [শুধু মাত্র সকালে]	নাড়ীর গতি (প্রতি মিনিটে))	শ্বাসের গতি (প্রতি মিনিট)	তাপমাত্রা (বগলের) (^{0}সে.)	ব্লাড প্রেসার. **Systolic** (mmHg) [শুধু মাত্র সকালে]	ব্লাড প্রেসার. **Diastolic** (mmHg) [শুধু মাত্র সকালে]
১ম আট ঘন্টা সময়						
২য় আট ঘন্টা সময়						
৩য় আট ঘন্টা সময়						
যদি কোন reading স্বাভাবিক পরিসীমা অতিক্রম করে?	☐ না ☐ হ্যাঁ	☐ না ☐ হ্যাঁ	☐ না ☐ হ্যাঁ	☐ না ☐ হ্যাঁ	☐ না ☐ হ্যাঁ	☐ না ☐ হ্যাঁ
যদি হ্যাঁ হয় তবে, reading clinically Significant কিনা?*	☐ না ☐ হ্যাঁ	☐ না ☐ হ্যাঁ	☐ না ☐ হ্যাঁ	☐ না ☐ হ্যাঁ	☐ না ☐ হ্যাঁ	☐ না ☐ হ্যাঁ

***যদি reading Clinically Significant হয় তবে প্রয়োজন হলে অনুগ্রহ করে Adverse Event Form পূরণ করুন]*

ডায়রিয়া সাথে সম্পর্কিত লক্ষন সমূহ

বর্ণনা	না	হ্যাঁ
পেটে ব্যাথা আছে কিনা?	☐	☐
পেটে ফাঁপা আছে কিনা?	☐	☐
জ্বর আছে কিনা?	☐	☐
শারিরীক দুর্বলতা আছে কিনা?	☐	☐
বমি আছে কিনা?<br>যদি হ্যাঁ হয়, প্রতি দিন কত বার বমি হয় ? ☐☐	☐	☐

Study ID No.ঃ [| |]

K	W	M	C	H

Hospital ID No [| | | | | | | | |]

অন্যান্য অস্বাভাবিকতা উপস্থিতি যদি হ্যাঁ হয়, নির্দিষ্ট করুন______________________	☐	☐
পায়খানার সাথে মলের *উপস্থিতির ইতিহাস*		☐
পায়খানার সাথে রক্তের *উপস্থিতির ইতিহাস*		
চাল ধোয়া পানির মত পায়খানার ইতিহাস		

CRF 21

খাবার এবং স্যালাইন গ্রহন

তারিখ (দিন মাস বছর)	গ্রহনের ৮ ঘন্টা সময় (ঘন্টা মিনিট)	খাবার গ্রহন (গ্রাম)	ORS/Rice-স্যালাইন গ্রহন (মি.লি)	তৈরি করা দুধ (মি.লি)	(শিশুটি) বুকের দুধ খায় কিনা	যদি হ্যাঁ হয় কত বার
☐☐ ☐☐☐ ☐☐	☐☐ : ☐☐	☐☐☐☐	☐☐☐☐	☐☐☐☐	☐ না ☐ হ্যাঁ	☐☐
☐☐ ☐☐☐ ☐☐	☐☐ : ☐☐	☐☐☐☐	☐☐☐☐	☐☐☐☐	☐ না ☐ হ্যাঁ	☐☐
☐☐ ☐☐☐ ☐☐	☐☐ : ☐☐	☐☐☐☐	☐☐☐☐	☐☐☐☐	☐ না ☐ হ্যাঁ	☐☐

প্রস্রাব এবং মল ত্যাগের পরিমান

তারিখ (দিন মাস বছর)	পরিমাপ ৮ ঘন্টায় (ঘন্টা/ মিনিট)	এই ৮ ঘন্টায় পানির মত পায়খানা করেছে	পায়খানার পরিমান (গ্রাম)	পায়খানা কত বার	পায়খানার ধরণ*	প্রস্রাবের পরিমান (মি.লি)
☐☐ ☐☐☐ ☐☐	☐☐ : ☐☐	☐ না ☐ হ্যাঁ	☐☐☐☐	☐☐	☐	☐☐☐☐
☐☐ ☐☐☐ ☐☐	☐☐ : ☐☐	☐ না ☐ হ্যাঁ	☐☐☐☐	☐☐	☐	☐☐☐☐
☐☐ ☐☐☐ ☐☐	☐☐ : ☐☐	☐ না ☐ হ্যাঁ	☐☐☐☐	☐☐	☐	☐☐☐☐

* **পায়খানার ধরণ** *(১= শক্ত, ২= নরম, ৩ = পাতলা / পানির মত, ৪ = পাতলা পায়খানার সাথে রক্ত)*

সামগ্রিকভাবে খাবার এবং দুধ গ্রহনের পরিমান *(দিন শেষ হওয়ার ১ ঘন্টা পূর্বে)*

শিশুর খাবার / দুধ খাওয়ার অবস্থা নিচে উল্লিখিত স্কোর অনুযায়ী<br>(১=স্বাভাবিক, ২=কিছুটা কম, ৩=মধ্যম কম, ৪=খুব বেশি কম, ৫=খাবার গ্রহনে অস্বীকৃতি)	☐

পানি শূন্যতার পরিমান *(দিন শেষ হওয়ার ১ ঘন্টা পূর্বে)*

নিচে উল্লেখিত তালিকা অনুযায়ী শিশুর পানি শূন্যতা :	☐

Study ID No.ঃ | | | |

K	W	M	C	H

Hospital ID No | | | | | | | | | | |

(০=পানি শূন্যতা নাই ১=কিছুটা পানি শূন্যতা ২= মারাত্বক পানি শূন্যতা *)<br>* যদি মারাত্বক পানি শূন্যতা হয় তাহলে শিশুকে গবেষণা থেকে বাদ দিন	

প্রতিকূল ঘটনায় প্রযোজ্য ঔষধ সমূহ

গত পরিদর্শনের সময় দেওয়া ঔষধ সমূহ পরিবর্তনের প্রয়োজন কিনা ?<br>*যদি হ্যাঁ হয় তবে প্রযোজ্য লিষ্ট থেকে নতুন ঔষধ যোগ করুন*	☐ না	☐ হ্যাঁ

CRF 22

পানীয় (ফাইবারসল-২/প্লাসিবু) গ্রহনের পর শিশুর প্রতিক্রিয়া :

৩য় দিনের ১ম ডোজ

৩০ মিনিটে ফাইবারসল-২/প্লাসিবু গ্রহনের পরিমান : ☐.☐ গ্রাম

বর্ণনা	না	হ্যাঁ
পেট ফুলে গিয়েছে কিনা?	☐	☐
পেটে ব্যাথা আছে কিনা?	☐	☐
পেট ডাকে কিনা?	☐	☐
পেটে ফাঁপা আছে কিনা?	☐	☐
শিশুটি খাবার থেকে মুখ ঘুরিয়ে নেয় কিনা ?	☐	☐
শিশুটি মুখ বন্ধ করে রাখে কিনা ?	☐	☐
শিশুটি মাড়ি কামড়ে রাখে কিনা ?	☐	☐
শিশুটি বিরক্ত হয়েছে কিনা ?	☐	☐
শিশুটি মুখ থেকে **ফাইবারসল-২/প্লাসিবু** ফেলে দেয় কিনা ?	☐	☐
শিশুটি **ফাইবারসল-২/প্লাসিবু** গিলতে অস্বীকার করে কিনা ?	☐	☐

৩য় দিনের ২য় ডোজ

৩০ মিনিটে ফাইবারসল-২/প্লাসিবু গ্রহনের পরিমান : ☐.☐ গ্রাম

বর্ণনা	না	হ্যাঁ
পেট ফুলে গিয়েছে কিনা?	☐	☐
পেটে ব্যাথা আছে কিনা?	☐	☐
	☐	☐

Study ID No.: | | | |

K	W	M	C	H

Hospital ID No

CRF 23

পেট ডাকে কিনা?		
পেটে ফাঁপা আছে কিনা?		
শিশুটি খাবার থেকে মুখ ঘুরিয়ে নেয় কিনা ?	☐	☐
শিশুটি মুখ বন্ধ করে রাখে কিনা ?	☐	☐
শিশুটি মাড়ি কামড়ে রাখে কিনা ?	☐	☐
শিশুটি বিরক্ত হয়েছে কিনা ?	☐	☐
শিশুটি মুখ থেকে **ফাইবারসল-২/প্লাসিবু** ফেলে দেয় কিনা ?	☐	☐
শিশুটি **ফাইবারসল-২/প্লাসিবু** গিলতে অস্বীকার করে কিনা ?	☐	☐

বমি আছে কিনা?<br>যদি হ্যাঁ হয়, প্রতি দিন কত বার বমি হয় ? ☐☐ বার<br>প্রতিদিন কি পরিমান বমি করেছে ☐☐☐ গ্রাম	না ☐ হ্যাঁ ☐
মায়ের দৃষ্টিতে শিশুটি **ফাইবারসল-২/প্লাসিবু পছন্দ করেছে কিনা ?**	☐

*** শিশুটি পানীয় নিতে অস্বীকার করেছে বলে বিবেচনা করবো যদি সে পানীয় থেকে মুখ ফিরিয়ে নেয়, কান্নাকাটি করে ,মুখ জোর করে বন্ধ করে রাখে অথবা দাত খিচিয়ে রাখে অথবা বিরক্ত হয়ে যায়, মুখ থেকে ফেলে দেয় কিংবা গিলতে অস্বীকার করে। আগে থেকে ওজন নেয়া নেপকিন সরবরাহ করা হবে। কোনো খাবার যদি উগড়ে ফেলে বা বমি করে ফেলে দেয়া হয়, তা মুছে নেপকিনের ওজন নেয়া হবে এবং সরবরাহকৃত পরিমান থেকে বাদ দেয়া হবে। ৭ পয়েন্টের হেডোনিক স্কেল ব্যবহার করা হবে যেখানে প্রতিটি পয়েন্ট মুখভঙ্গির অঙ্কন দিয়ে নির্ধারন করা হবে। আমরা মায়েদের থেকে খাবারের রং,গন্ধ, স্বাদ এবং সার্বিক গ্রহনযোগ্যতা সর্ম্পকে মতামত চেয়েছি। (১=খুবই অপছন্দ, ২=কিছুটা অপছন্দ, ৩= অপছন্দ, ৪= পছন্দ-অপছন্দ কিছুই না, ৫=কিছুটা পছন্দ, ৬=মোটামুটি পছন্দ, ৭= খুবই পছন্দ)।

পায়খানার ধরণ :	☐

* **পায়খানার ধরণ** *(১= শক্ত, ২= নরম, ৩ = পাতলা / পানির মত, ৪ = পাতলা পায়খানার সাথে রক্ত)*

Study ID No.: | | | |

K	W	M	C	H

Hospital ID No

CRF 24

গবেষণার ৪র্থ দিন

পরিদর্শনের তারিখ (দিন মাস বছর)	পরিদর্শনের সময় (ঘন্টা : মিনিট)
	:

গবেষণার জিনিসপত্র/ ঔষধ দেওয়ার সূচী

ডোজ	তারিখ (দিন মাস বছর)	সময় (ঘন্টা : মিনিট)	দেওয়ার পরিমান (মিলি)	দেওয়ার বাকী আছে (মিলি)
১		:	.	

জিংক দেওয়ার সূচী [*test article administration* এর কমপক্ষে ৩ ঘন্টা পর খাবারের সাথে জিংক দিতে হবে]

ডোজ	তারিখ (দিন মাস বছর)	সময় (ঘন্টা : মিনিট)	দেওয়ার পরিমান (মিলি)	দেওয়ার বাকী আছে (মিলি)
১		:		

ডোজ	তারিখ (দিন মাস বছর)	সময় (ঘন্টা : মিনিট)	দেওয়ার পরিমান (মিলি)	দেওয়ার বাকী আছে (মিলি)

Study ID No.ঃ | | | |

K	W	M	C	H

Hospital ID No

২		:		

গবেষণার জিনিসপত্র/ ঔষধ দেওয়ার সূচী

ডোজ	তারিখ (দিন মাস বছর)	সময় (ঘন্টা : মিনিট)	দেওয়ার পরিমান (মিলি)	দেওয়ার বাকী আছে (মিলি)
২		:	.	

জিংক দেওয়ার সূচী *[test article administration এর কমপক্ষে ৩ ঘন্টা পর খাবারের সাথে জিংক দিতে হবে]*

CRF 25

অত্যাবশ্যকীয় লক্ষণ

**Variables** সময় গণনা (প্রতি ৮ ঘন্টা পর পর গণনা করুন)	ওজন (কেজি) [শুধু মাত্র সকালে]	নাড়ীর গতি (প্রতি মিনিটে))	শ্বাসের গতি(মিনিট)	তাপমাত্রা (বগলের) (^{0}সে.)	ব্লাড প্রেসার. **Systolic** (mmHg) [শুধু মাত্র সকালে]	ব্লাড প্রেসার. **Diastolic** (mmHg) [শুধু মাত্র সকালে]
১ম আট ঘন্টা সময়						
২য় আট ঘন্টা সময়						
৩য় আট ঘন্টা সময়						
যদি কোন reading স্বাভাবিক পরিসীমা অতিক্রম করে?	☐ না ☐ হ্যাঁ	☐ না ☐ হ্যাঁ	☐ না ☐ হ্যাঁ	☐ না ☐ হ্যাঁ	☐ না ☐ হ্যাঁ	☐ না ☐ হ্যাঁ
যদি হ্যাঁ হয় তবে, reading clinically Significant কিনা?*	☐ না ☐ হ্যাঁ	☐ না ☐ হ্যাঁ	☐ না ☐ হ্যাঁ	☐ না ☐ হ্যাঁ	☐ না ☐ হ্যাঁ	☐ না ☐ হ্যাঁ

**যদি reading Clinically Significant হয় তবে প্রয়োজন হলে অনুগ্রহ করে Adverse Event Form পূরণ করুন]*

ডায়রিয়া সাথে সম্পর্কিত লক্ষন সমূহ

বর্ণনা	না	হ্যাঁ
পেটে ব্যাথা আছে কিনা?	☐	☐
	☐	☐
	☐	☐
	☐	☐

Study ID No.ঃ | | | |

K	W	M	C	H

Hospital ID No | | | | | | | | | | |

পেটে ফাঁপা আছে কিনা?		
জ্বর আছে কিনা?		
শারিরীক দুর্বলতা আছে কিনা?		
বমি আছে কিনা?<br>যদি হ্যাঁ হয়, প্রতি দিন কত বার বমি হয় ? ☐☐		
অন্যান্য অস্বাভাবিকতা উপস্থিতি যদি হ্যাঁ হয়, নির্দিষ্ট করুন____________________	☐	☐
পায়খানার সাথে মলের *উপস্থিতির ইতিহাস*		☐
পায়খানার সাথে রক্তের *উপস্থিতির ইতিহাস*		
চাল ধোয়া পানির মত পায়খানার ইতিহাস		

CRF 26

খাবার এবং স্যালাইন গ্রহন

তারিখ (দিন মাস বছর)	গ্রহনের ৮ ঘন্টা সময় (ঘন্টা মিনিট)	খাবার গ্রহন (গ্রাম)	ORS/Rice-স্যালাইন গ্রহন (মি.লি)	তৈরি করা দুধ (মি.লি)	(শিশুটি) বুকের দুধ খায় কিনা	যদি হ্যাঁ হয় কত বার
☐☐ ☐☐☐ ☐☐	☐☐ : ☐☐	☐☐☐☐	☐☐☐☐	☐☐☐☐	☐ না<br>☐ হ্যাঁ	☐☐
☐☐ ☐☐☐ ☐☐	☐☐ : ☐☐	☐☐☐☐	☐☐☐☐	☐☐☐☐	☐ না<br>☐ হ্যাঁ	☐☐
☐☐ ☐☐☐ ☐☐	☐☐ : ☐☐	☐☐☐☐	☐☐☐☐	☐☐☐☐	☐ না<br>☐ হ্যাঁ	☐☐

প্রস্রাব এবং মল ত্যাগের পরিমান

তারিখ (দিন মাস বছর)	পরিমাপ ৮ ঘন্টায় (ঘন্টা/ মিনিট)	এই ৮ ঘন্টায় পানির মত পায়খানা করেছে	পায়খানার পরিমান (গ্রাম)	পায়খানা কত বার	পায়খানার ধরণ*	প্রস্রাবের পরিমান (মি.লি)
☐☐ ☐☐☐ ☐☐	☐☐ : ☐☐	☐ না ☐ হ্যাঁ	☐☐☐☐	☐☐	☐	☐☐☐☐
☐☐ ☐☐☐ ☐☐	☐☐ : ☐☐	☐ না ☐ হ্যাঁ	☐☐☐☐	☐☐	☐	☐☐☐☐
☐☐ ☐☐☐ ☐☐	☐☐ : ☐☐	☐ না ☐ হ্যাঁ	☐☐☐☐	☐☐	☐	☐☐☐☐

* **পায়খানার ধরণ** *(১= শক্ত, ২= নরম, ৩ = পাতলা / পানির মত, ৪ = পাতলা পায়খানার সাথে রক্ত)*

সামগ্রিকভাবে খাবার এবং দুধ গ্রহনের পরিমান *(দিন শেষ হওয়ার ১ ঘন্টা পূর্বে)*

Study ID No.ঃ [ ][ ][ ]

K	W	M	C	H

Hospital ID No [ ][ ][ ][ ][ ][ ][ ][ ][ ][ ]

শশিুর খাবার / দুধ খাওয়ার অবস্থা নচিে উল্লখেতি স্কোর অনুযায়ী<br>(১=স্বাভাবিক, ২=কিছুটা কম, ৩=মধ্যম কম, ৪=খুব বেশি কম, ৫=খাবার গ্রহনে অস্বীকৃতি)	☐

পানি শূন্যতার পরিমান *(দিন শেষ হওয়ার ১ ঘন্টা পূর্বে)*

নিচে উল্লেখিত তালিকা অনুযায়ী শিশুর পানি শূন্যতা :<br>(০=পানি শূন্যতা নাই ১=কিছুটা পানি শূন্যতা ২= মারাত্বক পানি শূন্যতা *)<br>* যদি মারাত্বক পানি শূন্যতা হয় তাহলে শিশুকে গবেষণা থেকে বাদ দিন	☐

প্রতিকূল ঘটনায় প্রযোজ্য ঔষধ সমূহ

গত পরিদর্শনের সময় দেওয়া ঔষধ সমূহ পরিবর্তনের প্রয়োজন কিনা ?<br>*যদি হ্যাঁ হয় তবে প্রযোজ্য লিষ্ট থেকে নতুন ঔষধ যোগ করুন*	☐ না	☐ হ্যাঁ

CRF 27

পানীয় (ফাইবারসল-২/প্লাসিবু) গ্রহনের পর শিশুর প্রতিক্রিয়া :

৪র্থ দিনের ১ম ডোজ

৩০ মিনিটে ফাইবারসল-২/প্লাসিবু গ্রহনের পরিমান : [ ].[ ] গ্রাম

বর্ণনা	না	হ্যাঁ
পেট ফুলে গিয়েছে কিনা?	☐	☐
পেটে ব্যাথা আছে কিনা?	☐	☐
পেট ডাকে কিনা?	☐	☐
পেটে ফাঁপা আছে কিনা?	☐	☐
শিশুটি খাবার থেকে মুখ ঘুরিয়ে নেয় কিনা ?	☐	☐
শিশুটি মুখ বন্ধ করে রাখে কিনা ?	☐	☐
শিশুটি মাড়ি কামড়ে রাখে কিনা ?	☐	☐
শিশুটি বিরক্ত হয়েছে কিনা ?	☐	☐
শিশুটি মুখ থেকে **ফাইবারসল-২/প্লাসিবু** ফেলে দেয় কিনা ?	☐	☐
শিশুটি **ফাইবারসল-২/প্লাসিবু** গিলতে অস্বীকার করে কিনা ?	☐	☐

৪র্থ দিনের ২য় ডোজ

৩০ মিনিটে ফাইবারসল-২/প্লাসিবু গ্রহনের পরিমান : [ ].[ ] গ্রাম

Study ID No.ঃ | | | |

K	W	M	C	H

Hospital ID No | | | | | | | | | | |

বর্ণনা	না	হ্যাঁ
পেট ফুলে গিয়েছে কিনা?	☐	☐
পেটে ব্যাথা আছে কিনা?	☐	☐
পেট ডাকে কিনা?	☐	☐
পেটে ফাঁপা আছে কিনা?	☐	☐
শিশুটি খাবার থেকে মুখ ঘুরিয়ে নেয় কিনা ?	☐	☐
শিশুটি মুখ বন্ধ করে রাখে কিনা ?	☐	☐
শিশুটি মাড়ি কামড়ে রাখে কিনা ?	☐	☐
শিশুটি বিরক্ত হয়েছে কিনা ?	☐	☐
শিশুটি মুখ থেকে **ফাইবারসল-২/প্লাসিবু** ফেলে দেয় কিনা ?	☐	☐
শিশুটি **ফাইবারসল-২/প্লাসিবু** গিলতে অস্বীকার করে কিনা ?	☐	☐

CRF 28

বমি আছে কিনা?<br>যদি হ্যাঁ হয়, প্রতি দিন কত বার বমি হয় ? ☐☐ বার<br>প্রতিদিন কি পরিমান বমি করেছে ☐☐☐ গ্রাম	না ☐ হ্যাঁ ☐
মায়ের দৃষ্টিতে শিশুটি **ফাইবারসল-২/প্লাসিবু পছন্দ করেছে কিনা ?**	☐

*** শিশুটি পানীয় নিতে অস্বীকার করেছে বলে বিবেচনা করবো যদি সে পানীয় থেকে মুখ ফিরিয়ে নেয়, কান্নাকাটি করে ,মুখ জোর করে বন্ধ করে রাখে অথবা দাত খিচিয়ে রাখে অথবা বিরক্ত হয়ে যায়, মুখ থেকে ফেলে দেয় কিংবা গিলতে অস্বীকার করে। আগে থেকে ওজন নেয়া নেপকিন সরবরাহ করা হবে। কোনো খাবার যদি উগড়ে ফেলে বা বমি করে ফেলে দেয়া হয়, তা মুছে নেপকিনের ওজন নেয়া হবে এবং সরবরাহকৃত পরিমান থেকে বাদ দেয়া হবে। ৭ পয়েন্টের হেডোনিক স্কেল ব্যবহার করা হবে যেখানে প্রতিটি পয়েন্ট মুখভঙ্গির অঙ্কন দিয়ে নির্ধারন করা হবে। আমরা মায়েদের থেকে খাবারের রং,গন্ধ, স্বাদ এবং সার্বিক গ্রহনযোগ্যতা সর্ম্পকে মতামত চেয়েছি। (১=খুবই অপছন্দ, ২=কিছুটা অপছন্দ, ৩= অপছন্দ, ৪= পছন্দ-অপছন্দ কিছুই না, ৫=কিছুটা পছন্দ, ৬=মোটামুটি পছন্দ, ৭= খুবই পছন্দ)।

পায়খানার ধরণ :	☐

* **পায়খানার ধরণ** *(১= শক্ত, ২= নরম, ৩ = পাতলা / পানির মত, ৪ = পাতলা পায়খানার সাথে রক্ত)*

Study ID No.ঃ | | | |

K	W	M	C	H

Hospital ID No

CRF 29

গবেষণার ৫ম দিন

পরিদর্শনের তারিখ (দিন মাস বছর)	পরিদর্শনের সময় (ঘন্টা : মিনিট)
	:

গবেষণার জিনিসপত্র/ ঔষধ দেওয়ার সূচী

ডোজ	তারিখ (দিন মাস বছর)	সময় (ঘন্টা : মিনিট)	দেওয়ার পরিমান (মিলি)	দেওয়ার বাকী আছে (মিলি)
১		:		

জিংক দেওয়ার সূচী *[test article administration এর কমপক্ষে ৩ ঘন্টা পর খাবারের সাথে জিংক দিতে হবে]*

ডোজ	তারিখ (দিন মাস বছর)	সময় (ঘন্টা : মিনিট)	দেওয়ার পরিমান (মিলি)	দেওয়ার বাকী আছে (মিলি)
১		:	.	

Study ID No.ঃ | | | |

K	W	M	C	H

Hospital ID No

গবেষণার জিনিসপত্র/ ঔষধ দেওয়ার সূচী

ডোজ	তারিখ (দিন মাস বছর)	সময় (ঘন্টা : মিনিট)	দেওয়ার পরিমান (মিলি)	দেওয়ার বাকী আছে (মিলি)
২		:		

জিংক দেওয়ার সূচী *[test article administration এর কমপক্ষে ৩ ঘন্টা পর খাবারের সাথে জিংক দিতে হবে]*

ডোজ	তারিখ (দিন মাস বছর)	সময় (ঘন্টা : মিনিট)	দেওয়ার পরিমান (মিলি)	দেওয়ার বাকী আছে (মিলি)
২		:	.	

CRF 30

অত্যাবশ্যকীয় লক্ষণ

**Variables** সময় গণনা (প্রতি ৮ ঘন্টা পর পর গণনা করুন)	ওজন (কেজি) [শুধু মাত্র সকালে]	নাড়ীর গতি (প্রতি মিনিটে)	শ্বাসের গতি (প্রতি মিনিট)	তাপমাত্রা (বগলের) ($^{0সে.}$)	ব্লাড প্রেসার. **Systolic** (mmHg) [শুধু মাত্র সকালে]	ব্লাড প্রেসার. **Diastolic** (mmHg) [শুধু মাত্র সকালে]
১ম আট ঘন্টা সময়						
২য় আট ঘন্টা সময়						
৩য় আট ঘন্টা সময়						
যদি কোন reading স্বাভাবিক পরিসীমা অতিক্রম করে?	☐ না ☐ হ্যাঁ	☐ না ☐ হ্যাঁ	☐ না ☐ হ্যাঁ	☐ না ☐ হ্যাঁ	☐ না ☐ হ্যাঁ	☐ না ☐ হ্যাঁ
যদি হ্যাঁ হয় তবে, reading clinically Significant কিনা?*	☐ না ☐ হ্যাঁ	☐ না ☐ হ্যাঁ	☐ না ☐ হ্যাঁ	☐ না ☐ হ্যাঁ	☐ না ☐ হ্যাঁ	☐ না ☐ হ্যাঁ

**যদি reading Clinically Significant হয় তবে প্রয়োজন হলে অনুগ্রহ করে Adverse Event Form পূরণ করুন]*

Study ID No.ঃ [| |]

K	W	M	C	H

Hospital ID No

ডায়রিয়া সাথে সম্পর্কিত লক্ষন সমূহ

বর্ণনা	না	হ্যাঁ
পেটে ব্যাথা আছে কিনা?	☐	☐
পেটে ফাঁপা আছে কিনা?	☐	☐
জ্বর আছে কিনা?	☐	☐
শারিরীক দুর্বলতা আছে কিনা?	☐	☐
বমি আছে কিনা?<br>যদি হ্যাঁ হয়, প্রতি দিন কত বার বমি হয় ? ☐☐	☐	☐
অন্যান্য অস্বাভাবিকতা উপস্থিতি যদি হ্যাঁ হয়, নির্দিষ্ট করুন________________________________	☐	☐
পায়খানার সাথে মলের *উপস্থিতির ইতিহাস*	☐	☐
পায়খানার সাথে রক্তের *উপস্থিতির ইতিহাস*	☐	☐
চাল ধোয়া পানির মত পায়খানার ইতিহাস	☐	☐

CRF 31

খাবার এবং স্যালাইন গ্রহন

তারিখ (দিন মাস বছর)	গ্রহনের ৮ ঘন্টা সময় (ঘন্টা মিনিট)	খাবার গ্রহন (গ্রাম)	ORS/Rice-স্যালাইন গ্রহন (মি.লি)	তৈরি করা দুধ (মি.লি)	(শিশুটি) বুকের দুধ খায় কিনা	যদি হ্যাঁ হয় কত বার
☐☐ ☐☐☐ ☐☐	☐☐ : ☐☐	☐☐☐☐	☐☐☐☐	☐☐☐☐	☐ না ☐ হ্যাঁ	☐☐
☐☐ ☐☐☐ ☐☐	☐☐ : ☐☐	☐☐☐☐	☐☐☐☐	☐☐☐☐	☐ না ☐ হ্যাঁ	☐☐
☐☐ ☐☐☐ ☐☐	☐☐ : ☐☐	☐☐☐☐	☐☐☐☐	☐☐☐☐	☐ না ☐ হ্যাঁ	☐☐

প্রস্রাব এবং মল ত্যাগের পরিমান

তারিখ (দিন মাস বছর)	পরিমাপ ৮ ঘন্টায় (ঘন্টা/ মিনিট)	এই ৮ ঘন্টায় পানির মত পায়খানা করেছে	পায়খানার পরিমান (গ্রাম)	পায়খানা কত বার	পায়খানার ধরণ*	প্রস্রাবের পরিমান (মি.লি)
☐☐ ☐☐☐ ☐☐	☐☐ : ☐☐	☐ না ☐ হ্যাঁ	☐☐☐☐	☐☐	☐	☐☐☐☐
☐☐ ☐☐☐ ☐☐	☐☐ : ☐☐	☐ না ☐ হ্যাঁ	☐☐☐☐	☐☐	☐	☐☐☐☐

Study ID No.ঃ | | | |

K	W	M	C	H

Hospital ID No

	:	না / হ্যাঁ				

* **পায়খানার ধরণ** *(১= শক্ত, ২= নরম, ৩ = পাতলা / পানির মত, ৪ = পাতলা পায়খানার সাথে রক্ত)*

সামগ্রিকভাবে খাবার এবং দুধ গ্রহনের পরিমান *(দিন শেষ হওয়ার ১ ঘন্টা পূর্বে)*

শশিুর খাবার / দুধ খাওয়ার অবস্থা নচিে উল্লখেতি স্কোর অনুযায়ী<br>(১=স্বাভাবিক, ২=কিছুটা কম, ৩=মধ্যম কম, ৪=খুব বেশি কম, ৫=খাবার গ্রহনে অস্বীকৃতি)	

পানি শূন্যতার পরিমান *(দিন শেষ হওয়ার ১ ঘন্টা পূর্বে)*

নিচে উল্লেখিত তালিকা অনুযায়ী শিশুর পানি শূন্যতা :<br>(০=পানি শূন্যতা নাই ১=কিছুটা পানি শূন্যতা ২= মারাত্বক পানি শূন্যতা *)<br>* যদি মারাত্বক পানি শূন্যতা হয় তাহলে শিশুকে গবেষণা থেকে বাদ দিন	

প্রতিকূল ঘটনায় প্রযোজ্য ঔষধ সমূহ

গত পরিদর্শনের সময় দেওয়া ঔষধ সমূহ পরিবর্তনের প্রয়োজন কিনা ?<br>*যদি হ্যাঁ হয় তবে প্রযোজ্য লিষ্ট থেকে নতুন ঔষধ যোগ করুন*	না	হ্যাঁ

CRF 32

পানীয় (ফাইবারসল-২/প্লাসিবু) গ্রহনের পর শিশুর প্রতিক্রিয়া :
৫ম দিনের ১ম ডোজ
৩০ মিনিটে ফাইবারসল-২/প্লাসিবু গ্রহনের পরিমান : ___ . ___ গ্রাম

বর্ণনা	না	হ্যাঁ
পেট ফুলে গিয়েছে কিনা?		
পেটে ব্যাথা আছে কিনা?		
পেট ডাকে কিনা?		
পেটে ফাঁপা আছে কিনা?		
শিশুটি খাবার থেকে মুখ ঘুরিয়ে নেয় কিনা ?		
শিশুটি মুখ বন্ধ করে রাখে কিনা ?		
শিশুটি মাড়ি কামড়ে রাখে কিনা ?		
শিশুটি বিরক্ত হয়েছে কিনা ?		

Study ID No.ঃ | | | |

K	W	M	C	H

Hospital ID No | | | | | | | | | | |

শিশুটি মুখ থেকে **ফাইবারসল-২/প্লাসিবু** ফেলে দেয় কিনা ?	☐	☐
শিশুটি **ফাইবারসল-২/প্লাসিবু** গিলতে অস্বীকার করে কিনা ?	☐	☐

৫ম দিনের ২য় ডোজ

৩০ মিনিটে ফাইবারসল-২/প্লাসিবু গ্রহনের পরিমান : ☐ . ☐ গ্রাম

বর্ণনা	না	হ্যাঁ
পেট ফুলে গিয়েছে কিনা?	☐	☐
পেটে ব্যাথা আছে কিনা?	☐	☐
পেট ডাকে কিনা?	☐	☐
পেটে ফাঁপা আছে কিনা?	☐	☐
শিশুটি খাবার থেকে মুখ ঘুরিয়ে নেয় কিনা ?	☐	☐
শিশুটি মুখ বন্ধ করে রাখে কিনা ?	☐	☐
শিশুটি মাড়ি কামড়ে রাখে কিনা ?	☐	☐
শিশুটি বিরক্ত হয়েছে কিনা ?	☐	☐
শিশুটি মুখ থেকে **ফাইবারসল-২/প্লাসিবু** ফেলে দেয় কিনা ?	☐	☐
শিশুটি **ফাইবারসল-২/প্লাসিবু** গিলতে অস্বীকার করে কিনা ?	☐	☐

CRF 33

বমি আছে কিনা?<br>যদি হ্যাঁ হয়, প্রতি দিন কত বার বমি হয় ? ☐☐ বার<br>প্রতিদিন কি পরিমান বমি করেছে ☐☐☐ গ্রাম	না ☐ হ্যাঁ ☐
মায়ের দৃষ্টিতে শিশুটি **ফাইবারসল-২/প্লাসিবু পছন্দ করেছে কিনা ?**	☐

*** শিশুটি পানীয় নিতে অস্বীকার করেছে বলে বিবেচনা করবো যদি সে পানীয় থেকে মুখ ফিরিয়ে নেয়, কান্নাকাটি করে ,মুখ জোর করে বন্ধ করে রাখে অথবা দাত খিচিয়ে রাখে অথবা বিরক্ত হয়ে যায়, মুখ থেকে ফেলে দেয় কিংবা গিলতে অস্বীকার করে। আগে থেকে ওজন নেয়া নেপকিন সরবরাহ করা হবে। কোনো খাবার যদি উগড়ে ফেলে বা বমি করে ফেলে দেয়া হয়, তা মুছে নেপকিনের ওজন নেয়া হবে এবং সরবরাহকৃত পরিমান থেকে বাদ দেয়া হবে। ৭ পয়েন্টের হেডোনিক স্কেল ব্যবহার করা হবে যেখানে প্রতিটি পয়েন্ট মুখভঙ্গির অঙ্কন দিয়ে নির্ধারন করা হবে। আমরা মায়েদের থেকে খাবারের রং,গন্ধ, স্বাদ এবং সার্বিক গ্রহনযোগ্যতা সর্ম্পকে মতামত চেয়েছি। (১=খুবই অপছন্দ, ২=কিছুটা অপছন্দ, ৩= অপছন্দ, ৪= পছন্দ-অপছন্দ কিছুই না, ৫=কিছুটা পছন্দ, ৬=মোটামুটি পছন্দ, ৭= খুবই পছন্দ)।

পায়খানার ধরণ :	☐

* **পায়খানার ধরণ** *(১= শক্ত, ২= নরম, ৩ = পাতলা / পানির মত, ৪ = পাতলা পায়খানার সাথে রক্ত)*

Study ID No.ঃ | | | |

K	W	M	C	H

Hospital ID No

CRF 34

গবেষণার ৬ষ্ঠ দিন

পরিদর্শনের তারিখ (দিন মাস বছর)	পরিদর্শনের সময় (ঘন্টা : মিনিট)
	:

গবেষণার জিনিসপত্র/ ঔষধ দেওয়ার সূচী

ডোজ	তারিখ (দিন মাস বছর)	সময় (ঘন্টা : মিনিট)	দেওয়ার পরিমান (মিলি)	দেওয়ার বাকী আছে (মিলি)
১		:		

জিংক দেওয়ার সূচী *[test article administration এর কমপক্ষে ৩ ঘন্টা পর খাবারের সাথে জিংক দিতে হবে]*

Study ID No.ঃ | | | |

K	W	M	C	H

Hospital ID No | | | | | | | | | | |

ডোজ	তারিখ (দিন মাস বছর)	সময় (ঘন্টা : মিনিট)	দেওয়ার পরিমান (মিলি)	দেওয়ার বাকী আছে (মিলি)
১		:	.	

গবেষণার জিনিসপত্র/ ঔষধ দেওয়ার সূচী

ডোজ	তারিখ (দিন মাস বছর)	সময় (ঘন্টা : মিনিট)	দেওয়ার পরিমান (মিলি)	দেওয়ার বাকী আছে (মিলি)
২		:		

জিংক দেওয়ার সূচী *[test article administration এর কমপক্ষে ৩ ঘন্টা পর খাবারের সাথে জিংক দিতে হবে]*

ডোজ	তারিখ (দিন মাস বছর)	সময় (ঘন্টা : মিনিট)	দেওয়ার পরিমান (মিলি)	দেওয়ার বাকী আছে (মিলি)
২		:	.	

CRF 35

অত্যাবশ্যকীয় লক্ষণ

**Variables** সময় গণনা (প্রতি ৮ ঘন্টা পর পর গণনা করুন)	ওজন (কেজি) [শুধু মাত্র সকালে]	নাড়ীর গতি (প্রতি মিনিটে)	শ্বাসের গতি (প্রতি মিনিট)	তাপমাত্রা (বগলের) ($^{0সে.}$)	ব্লাড প্রেসার. **Systolic** (mmHg) [শুধু মাত্র সকালে]	ব্লাড প্রেসার. **Diastolic** (mmHg) [শুধু মাত্র সকালে]
১ম আট ঘন্টা সময়						
২য় আট ঘন্টা সময়						
৩য় আট ঘন্টা সময়						
যদি কোন reading স্বাভাবিক পরিসীমা অতিক্রম করে?	☐ না ☐ হ্যাঁ	☐ না ☐ হ্যাঁ	☐ না ☐ হ্যাঁ	☐ না ☐ হ্যাঁ	☐ না ☐ হ্যাঁ	☐ না ☐ হ্যাঁ

Study ID No.ঃ ☐☐☐

K	W	M	C	H

Hospital ID No ☐☐☐☐☐☐☐☐☐☐

যদি হ্যাঁ হয় তবে, reading clinically Significant কিনা?*	☐ না ☐ হ্যাঁ	☐ না ☐ হ্যাঁ	☐ না ☐ হ্যাঁ	☐ না ☐ হ্যাঁ	☐ না ☐ হ্যাঁ	☐ না ☐ হ্যাঁ
**যদি reading Clinically Significant হয় তবে প্রয়োজন হলে অনুগ্রহ করে Adverse Event Form পূরণ করুন]*						

ডায়রিয়া সাথে সম্পর্কিত লক্ষন সমূহ

বর্ণনা	না	হ্যাঁ
পেটে ব্যাথা আছে কিনা?	☐	☐
পেটে ফাঁপা আছে কিনা?	☐	☐
জ্বর আছে কিনা?	☐	☐
শারিরীক দুর্বলতা আছে কিনা?	☐	☐
বমি আছে কিনা?<br>যদি হ্যাঁ হয়, প্রতি দিন কত বার বমি হয় ? ☐☐	☐	☐
অন্যান্য অস্বাভাবিকতা উপস্থিতি যদি হ্যাঁ হয়, নির্দিষ্ট করুন______________________________	☐	☐
পায়খানার সাথে মলের *উপস্থিতির ইতিহাস*	☐	☐
পায়খানার সাথে রক্তের *উপস্থিতির ইতিহাস*	☐	☐
চাল ধোয়া পানির মত পায়খানার ইতিহাস	☐	☐

CRF 36

খাবার এবং স্যালাইন গ্রহন

তারিখ (দিন মাস বছর)	গ্রহনের ৮ ঘন্টা সময় (ঘন্টা মিনিট)	খাবার গ্রহন (গ্রাম)	ORS/Rice-স্যালাইন গ্রহন (মি.লি)	তৈরি করা দুধ (মি.লি)	(শিশুটি) বুকের দুধ খায় কিনা	যদি হ্যাঁ হয় কত বার
☐☐ ☐☐☐ ☐☐	☐☐ : ☐☐	☐☐☐☐	☐☐☐☐	☐☐☐☐	☐ না ☐ হ্যাঁ	☐☐
☐☐ ☐☐☐ ☐☐	☐☐ : ☐☐	☐☐☐☐	☐☐☐☐	☐☐☐☐	☐ না ☐ হ্যাঁ	☐☐
☐☐ ☐☐☐ ☐☐	☐☐ : ☐☐	☐☐☐☐	☐☐☐☐	☐☐☐☐	☐ না ☐ হ্যাঁ	☐☐

প্রস্রাব এবং মল ত্যাগের পরিমান

তারিখ	পরিমাপ ৮ ঘন্টায় (ঘন্টা/ মিনিট)	এই ৮ ঘন্টায় পানির মত	পায়খানার পরিমান	পায়খানা কত বার	পায়খানার ধরণ*	প্রস্রাবের পরিমান (মি.লি)

Study ID No.ঃ

K	W	M	C	H

Hospital ID No

(দিন মাস বছর)		পায়খানা করেছে	(গ্রাম)			
	:	না হ্যাঁ				
	:	না হ্যাঁ				
	:	না হ্যাঁ				

* **পায়খানার ধরণ** *(১= শক্ত, ২= নরম, ৩ = পাতলা / পানির মত, ৪ = পাতলা পায়খানার সাথে রক্ত)*

সামগ্রিকভাবে খাবার এবং দুধ গ্রহনের পরিমান *(দিন শেষ হওয়ার ১ ঘন্টা পূর্বে)*

শশিুর খাবার / দুধ খাওয়ার অবস্থা নচিে উল্লখেতি স্কোর অনুযায়ী<br>(১=স্বাভাবিক, ২=কিছুটা কম, ৩=মধ্যম কম, ৪=খুব বেশি কম, ৫=খাবার গ্রহনে অস্বীকৃতি)	

পানি শূন্যতার পরিমান *(দিন শেষ হওয়ার ১ ঘন্টা পূর্বে)*

নিচে উল্লেখিত তালিকা অনুযায়ী শিশুর পানি শূন্যতা :<br>(০=পানি শূন্যতা নাই ১=কিছুটা পানি শূন্যতা ২= মারাত্বক পানি শূন্যতা *)<br>* যদি মারাত্বক পানি শূন্যতা হয় তাহলে শিশুকে গবেষণা থেকে বাদ দিন	

প্রতিকূল ঘটনায় প্রযোজ্য ঔষধ সমূহ

গত পরিদর্শনের সময় দেওয়া ঔষধ সমূহ পরিবর্তনের প্রয়োজন কিনা ?<br>*যদি হ্যাঁ হয় তবে প্রযোজ্য লিষ্ট থেকে নতুন ঔষধ যোগ করুন*	না	হ্যাঁ

CRF 37

পানীয় (ফাইবারসল-২/প্লাসিবু) গ্রহনের পর শিশুর প্রতিক্রিয়া :
৬ষ্ঠ দিনের ১ম ডোজ
৩০ মিনিটে ফাইবারসল-২/প্লাসিবু গ্রহনের পরিমান : . গ্রাম

বর্ণনা	না	হ্যাঁ
পেট ফুলে গিয়েছে কিনা?		
পেটে ব্যাথা আছে কিনা?		
পেট ডাকে কিনা?		
পেটে ফাঁপা আছে কিনা?		
শিশুটি খাবার থেকে মুখ ঘুরিয়ে নেয় কিনা ?		

Study ID No.ঃ | | | |

K	W	M	C	H

Hospital ID No | | | | | | | | | | |

শিশুটি মুখ বন্ধ করে রাখে কিনা ?		
শিশুটি মাড়ি কামড়ে রাখে কিনা ?	☐	☐
শিশুটি বিরক্ত হয়েছে কিনা ?	☐	☐
শিশুটি মুখ থেকে **ফাইবারসল-২/প্লাসিবু** ফেলে দেয় কিনা ?	☐	☐
শিশুটি **ফাইবারসল-২/প্লাসিবু** গিলতে অস্বীকার করে কিনা ?	☐	☐

৬ষ্ঠ দিনের ২য় ডোজ

৩০ মিনিটে ফাইবারসল-২/প্লাসিবু গ্রহনের পরিমান : ☐ . ☐ গ্রাম

CRF 38

বর্ণনা	না	হ্যাঁ
পেট ফুলে গিয়েছে কিনা?	☐	☐
পেটে ব্যাথা আছে কিনা?	☐	☐
পেট ডাকে কিনা?	☐	☐
পেটে ফাঁপা আছে কিনা?	☐	☐
শিশুটি খাবার থেকে মুখ ঘুরিয়ে নেয় কিনা ?	☐	☐
শিশুটি মুখ বন্ধ করে রাখে কিনা ?	☐	☐
শিশুটি মাড়ি কামড়ে রাখে কিনা ?	☐	☐
শিশুটি বিরক্ত হয়েছে কিনা ?	☐	☐
শিশুটি মুখ থেকে **ফাইবারসল-২/প্লাসিবু** ফেলে দেয় কিনা ?	☐	☐
শিশুটি **ফাইবারসল-২/প্লাসিবু** গিলতে অস্বীকার করে কিনা ?	☐	☐

বমি আছে কিনা?<br>যদি হ্যাঁ হয়, প্রতি দিন কত বার বমি হয় ? ☐☐ বার<br>প্রতিদিন কি পরিমান বমি করেছে ☐☐☐ গ্রাম	না ☐ হ্যাঁ ☐
মায়ের দৃষ্টিতে শিশুটি **ফাইবারসল-২/প্লাসিবু পছন্দ করেছে কিনা ?**	☐

*** শিশুটি পানীয় নিতে অস্বীকার করেছে বলে বিবেচনা করবো যদি সে পানীয় থেকে মুখ ফিরিয়ে নেয়, কান্নাকাটি করে ,মুখ জোর করে বন্ধ করে রাখে অথবা দাত খিচিয়ে রাখে অথবা বিরক্ত হয়ে যায়, মুখ থেকে ফেলে দেয় কিংবা গিলতে অস্বীকার করে। আগে থেকে ওজন নেয়া নেপকিন সরবরাহ করা হবে। কোনো খাবার যদি উগড়ে ফেলে বা বমি করে ফেলে দেয়া হয়, তা মুছে নেপকিনের ওজন নেয়া হবে এবং সরবরাহকৃত পরিমান থেকে বাদ দেয়া হবে। ৭ পয়েন্টের হেডোনিক স্কেল ব্যবহার করা হবে যেখানে প্রতিটি পয়েন্ট মুখভঙ্গির অঙ্কন দিয়ে নির্ধারন করা হবে। আমরা মায়েদের থেকে খাবারের রং,গন্ধ, স্বাদ এবং সার্বিক গ্রহনযোগ্যতা সর্ম্পকে মতামত চেয়েছি। (১=খুবই অপছন্দ, ২=কিছুটা অপছন্দ, ৩= অপছন্দ, ৪= পছন্দ-অপছন্দ কিছুই না, ৫=কিছুটা পছন্দ, ৬=মোটামুটি পছন্দ, ৭= খুবই পছন্দ)।

Study ID No.ঃ | | | |

K	W	M	C	H

Hospital ID No | | | | | | | | | | |

পায়খানার ধরণ :	☐

* **পায়খানার ধরণ** *(১= শক্ত, ২= নরম, ৩ = পাতলা / পানির মত, ৪ = পাতলা পায়খানার সাথে রক্ত*

CRF 39

গবেষণার ৭ম দিন

**পরিদর্শনের তারিখ** (দিন মাস বছর)	**পরিদর্শনের সময়** (ঘন্টা : মিনিট)
	:

গবেষণার জিনিসপত্র/ ঔষধ দেওয়ার সূচী

ডোজ	**তারিখ** (দিন মাস বছর)	**সময়** (ঘন্টা : মিনিট)	দেওয়ার পরিমান (মিলি)	দেওয়ার বাকী আছে (মিলি)
১		:		

জিংক দেওয়ার সূচী *[test article administration এর কমপক্ষে ৩ ঘন্টা পর খাবারের সাথে জিংক দিতে হবে]*

ডোজ	**তারিখ** (দিন মাস বছর)	**সময়** (ঘন্টা : মিনিট)	দেওয়ার পরিমান (মিলি)	দেওয়ার বাকী আছে (মিলি)
১		:	.	

গবেষণার জিনিসপত্র/ ঔষধ দেওয়ার সূচী

ডোজ	**তারিখ** (দিন মাস বছর)	**সময়** (ঘন্টা : মিনিট)	দেওয়ার পরিমান (মিলি)	দেওয়ার বাকী আছে (মিলি)
২		:		

জিংক দেওয়ার সূচী *[test article administration এর কমপক্ষে ৩ ঘন্টা পর খাবারের সাথে জিংক দিতে হবে]*

ডোজ	**তারিখ** (দিন মাস বছর)	**সময়** (ঘন্টা : মিনিট)	দেওয়ার পরিমান (মিলি)	দেওয়ার বাকী আছে (মিলি)
২		:	.	

Study ID No.: | | | |

K	W	M	C	H

Hospital ID No **CRF 40**

অত্যাবশ্যকীয় লক্ষণ

**Variables** সময় গণনা (প্রতি ৮ ঘন্টা পর পর গণনা করুন)	ওজন (কেজি) [শুধু মাত্র সকালে]	নাড়ীর গতি (প্রতি মিনিটে)	শ্বাসের গতি (প্রতি মিনিট)	তাপমাত্রা (বগলের) (^{0}সে.)	ব্লাড প্রেসার. **Systolic** (mmHg) [শুধু মাত্র সকালে]	ব্লাড প্রেসার. **Diastolic** (mmHg) [শুধু মাত্র সকালে]
১ম আট ঘন্টা সময়						
২য় আট ঘন্টা সময়						
৩য় আট ঘন্টা সময়						
যদি কোন reading স্বাভাবিক পরিসীমা অতিক্রম করে?	☐ না ☐ হ্যাঁ	☐ না ☐ হ্যাঁ	☐ না ☐ হ্যাঁ	☐ না ☐ হ্যাঁ	☐ না ☐ হ্যাঁ	☐ না ☐ হ্যাঁ
যদি হ্যাঁ হয় তবে, reading clinically Significant কিনা?*	☐ না ☐ হ্যাঁ	☐ না ☐ হ্যাঁ	☐ না ☐ হ্যাঁ	☐ না ☐ হ্যাঁ	☐ না ☐ হ্যাঁ	☐ না ☐ হ্যাঁ
***যদি reading Clinically Significant হয় তবে প্রয়োজন হলে অনুগ্রহ করে Adverse Event Form পূরণ করুন]**						

ডায়রিয়া সাথে সম্পর্কিত লক্ষন সমূহ

বর্ণনা	না	হ্যাঁ
পেট ফুলে গিয়েছে কিনা?	☐	☐
পেটে ব্যাথা আছে কিনা?	☐	☐
পেট ডাকে কিনা?	☐	☐
পেটে ফাঁপা আছে কিনা?	☐	☐
জ্বর আছে কিনা?	☐	☐
শারিরীক দুর্বলতা আছে কিনা?	☐	☐
বমি আছে কিনা?<br>যদি হ্যাঁ হয়, প্রতি দিন কত বার বমি হয় ? ☐☐	☐	☐
অন্যান্য অস্বাভাবিকতা উপস্থিতি যদি হ্যাঁ হয়, নির্দিষ্ট করুন____________________	☐	☐
পায়খানার সাথে মলের *উপস্থিতির ইতিহাস*	☐	☐

Study ID No.ঃ [| |]

K	W	M	C	H

Hospital ID No

[| | | | | | | | |]

পায়খানার সাথে রক্তের *উপস্থিতির ইতিহাস*	☐	☐
চাল ধোয়া পানির মত পায়খানার ইতিহাস	☐	☐

CRF 41

খাবার এবং স্যালাইন গ্রহন

তারিখ (দিন মাস বছর)	গ্রহনের ৮ ঘন্টা সময় (ঘন্টা মিনিট)	খাবার গ্রহন (গ্রাম)	ORS/Rice-স্যালাইন গ্রহন (মি.লি)	তৈরি করা দুধ (মি.লি)	(শিশুটি) বুকের দুধ খায় কিনা	যদি হ্যাঁ হয় কত বার
☐☐ ☐☐☐ ☐☐	☐☐ : ☐☐	☐☐☐☐	☐☐☐☐	☐☐☐☐	☐ না ☐ হ্যাঁ	☐☐
☐☐ ☐☐☐ ☐☐	☐☐ : ☐☐	☐☐☐☐	☐☐☐☐	☐☐☐☐	☐ না ☐ হ্যাঁ	☐☐
☐☐ ☐☐☐ ☐☐	☐☐ : ☐☐	☐☐☐☐	☐☐☐☐	☐☐☐☐	☐ না ☐ হ্যাঁ	☐☐

প্রস্রাব এবং মল ত্যাগের পরিমান

তারিখ (দিন মাস বছর)	পরিমাপ ৮ ঘন্টায় (ঘন্টা/ মিনিট)	এই ৮ ঘন্টায় পানির মত পায়খানা করেছে	পায়খানার পরিমান (গ্রাম)	পায়খানা কত বার	পায়খানার ধরণ*	প্রস্রাবের পরিমান (মি.লি)
☐☐ ☐☐☐ ☐☐	☐☐ : ☐☐	☐ না ☐ হ্যাঁ	☐☐☐☐	☐☐	☐	☐☐☐☐
☐☐ ☐☐☐ ☐☐	☐☐ : ☐☐	☐ না ☐ হ্যাঁ	☐☐☐☐	☐☐	☐	☐☐☐☐
☐☐ ☐☐☐ ☐☐	☐☐ : ☐☐	☐ না ☐ হ্যাঁ	☐☐☐☐	☐☐	☐	☐☐☐☐

* **পায়খানার ধরণ** *(১= শক্ত, ২= নরম, ৩ = পাতলা / পানির মত, ৪ = পাতলা পায়খানার সাথে রক্ত)*

সামগ্রিকভাবে খাবার এবং দুধ গ্রহনের পরিমান *(দিন শেষ হওয়ার ১ ঘন্টা পূর্বে)*

শশিুর খাবার / দুধ খাওয়ার অবস্থা নচিে উল্লখেতি স্কোর অনুযায়ী (১=স্বাভাবিক, ২=কিছুটা কম, ৩=মধ্যম কম, ৪=খুব বেশি কম, ৫=খাবার গ্রহনে অস্বীকৃতি)	☐

পানি শূন্যতার পরিমান *(দিন শেষ হওয়ার ১ ঘন্টা পূর্বে)*

নিচে উল্লেখিত তালিকা অনুযায়ী শিশুর পানি শূন্যতা :<br>(০=পানি শূন্যতা নাই ১=কিছুটা পানি শূন্যতা ২= মারাত্বক পানি শূন্যতা *)<br>* যদি মারাত্বক পানি শূন্যতা হয় তাহলে শিশুকে গবেষণা থেকে বাদ দিন	☐

প্রতিকূল ঘটনায় প্রযোজ্য ঔষধ সমূহ

গত পরিদর্শনের সময় দেওয়া ঔষধ সমূহ পরিবর্তনের প্রয়োজন কিনা ?<br>*যদি হ্যাঁ হয় তবে প্রযোজ্য লিষ্ট থেকে নতুন ঔষধ যোগ করুন*	☐ না	☐ হ্যাঁ

Study ID No.: | | | |

K	W	M	C	H

.

Hospital ID N **CRF 42**

পানীয় (ফাইবারসল-২/প্লাসিবু) গ্রহনের পর শিশুর প্রতিক্রিয়া :

৭ম দিনের ১ম ডোজ

৩০ মিনিটে ফাইবারসল-২/প্লাসিবু গ্রহনের পরিমান : ☐ . ☐ গ্রাম

বর্ণনা	না	হ্যাঁ
পেট ফুলে গিয়েছে কিনা?	☐	☐
পেটে ব্যাথা আছে কিনা?	☐	☐
পেট ডাকে কিনা?	☐	☐
পেটে ফাঁপা আছে কিনা?	☐	☐
শিশুটি খাবার থেকে মুখ ঘুরিয়ে নেয় কিনা ?	☐	☐
শিশুটি মুখ বন্ধ করে রাখে কিনা ?	☐	☐
শিশুটি মাড়ি কামড়ে রাখে কিনা ?	☐	☐
শিশুটি বিরক্ত হয়েছে কিনা ?	☐	☐
শিশুটি মুখ থেকে **ফাইবারসল-২/প্লাসিবু** ফেলে দেয় কিনা ?	☐	☐
শিশুটি **ফাইবারসল-২/প্লাসিবু** গিলতে অস্বীকার করে কিনা ?	☐	☐

৭ম দিনের ২য় ডোজ

৩০ মিনিটে ফাইবারসল-২/প্লাসিবু গ্রহনের পরিমান : ☐ . ☐ গ্রাম

বর্ণনা	না	হ্যাঁ
পেট ফুলে গিয়েছে কিনা?	☐	☐
পেটে ব্যাথা আছে কিনা?	☐	☐
পেট ডাকে কিনা?	☐	☐
পেটে ফাঁপা আছে কিনা?	☐	☐
শিশুটি খাবার থেকে মুখ ঘুরিয়ে নেয় কিনা ?	☐	☐
শিশুটি মুখ বন্ধ করে রাখে কিনা ?	☐	☐
শিশুটি মাড়ি কামড়ে রাখে কিনা ?	☐	☐
শিশুটি বিরক্ত হয়েছে কিনা ?	☐	☐
শিশুটি মুখ থেকে **ফাইবারসল-২/প্লাসিবু** ফেলে দেয় কিনা ?	☐	☐

☐ ☐

Study ID No.ঃ | | | |

K	W	M	C	H

Hospital ID No **CRF 43**

শিশুটি **ফাইবারসল-২/প্লাসিবু** গিলতে অস্বীকার করে কিনা ?		

বমি আছে কিনা?<br>যদি হ্যাঁ হয়, প্রতি দিন কত বার বমি হয় ? ☐☐ বার<br>প্রতিদিন কি পরিমান বমি করেছে ☐☐☐ গ্রাম	না ☐ হ্যাঁ ☐
মায়ের দৃষ্টিতে শিশুটি **ফাইবারসল-২/প্লাসিবু পছন্দ করেছে কিনা ?**	☐

*** শিশুটি পানীয় নিতে অস্বীকার করেছে বলে বিবেচনা করবো যদি সে পানীয় থেকে মুখ ফিরিয়ে নেয়, কান্নাকাটি করে ,মুখ জোর করে বন্ধ করে রাখে অথবা দাত খিচিয়ে রাখে অথবা বিরক্ত হয়ে যায়, মুখ থেকে ফেলে দেয় কিংবা গিলতে অস্বীকার করে। আগে থেকে ওজন নেয়া নেপকিন সরবরাহ করা হবে। কোনো খাবার যদি উগড়ে ফেলে বা বমি করে ফেলে দেয়া হয়, তা মুছে নেপকিনের ওজন নেয়া হবে এবং সরবরাহকৃত পরিমান থেকে বাদ দেয়া হবে। ৭ পয়েন্টের হেডোনিক স্কেল ব্যবহার করা হবে যেখানে প্রতিটি পয়েন্ট মুখভঙ্গির অঙ্কন দিয়ে নির্ধারন করা হবে। আমরা মায়েদের থেকে খাবারের রং,গন্ধ, স্বাদ এবং সার্বিক গ্রহনযোগ্যতা সর্ম্পকে মতামত চেয়েছি। (১=খুবই অপছন্দ, ২=কিছুটা অপছন্দ, ৩= অপছন্দ, ৪= পছন্দ-অপছন্দ কিছুই না, ৫=কিছুটা পছন্দ, ৬=মোটামুটি পছন্দ, ৭= খুবই পছন্দ)।

পায়খানার ধরণ :	☐

* **পায়খানার ধরণ** *(১= শক্ত, ২= নরম, ৩ = পাতলা / পানির মত, ৪ = পাতলা পায়খানার সাথে রক্ত)*

Study ID No.ঃ [| |]

K	W	M	C	H

Hospital ID No

CRF 44

৮ম দিন : ফলোআপ প্রশ্নপত্র
ছুটি হওয়ার ২৪ ঘন্টার মধ্যে

ফলোআপ পরিদর্শনের তারিখ :	দিন / মাস / বছর
পিতামাতা / বৈধ অভিভাবককে টেলিফোনে পাওয়া যাবে কিনা ?	☐ না ☐ হ্যাঁ

অন্যান্য রোগের ইতিহাস সাথে ডায়রিয়া

বর্ণনা	না	হ্যাঁ
পেট ফুলে গিয়েছে কিনা?	☐	☐
পেটে ব্যাথা আছে কিনা?	☐	☐
পেট ডাকে কিনা?	☐	☐
পেটে ফাঁপা আছে কিনা?	☐	☐
জ্বর আছে কিনা?	☐	☐
শারিরীক দুর্বলতা আছে কিনা?	☐	☐
বমি আছে কিনা?<br>যদি হ্যাঁ হয়, প্রতি দিন কত বার বমি হয়? [|]	☐	☐
অন্যান্য অস্বাভাবিকতা উপস্থিতি<br>যদি হ্যাঁ হয়, নির্দিষ্ট করুন________________	☐	☐
পায়খানার সাথে মলের *উপস্থিতির ইতিহাস*	☐	☐
পায়খানার সাথে রক্তের *উপস্থিতির ইতিহাস*	☐	☐
চাল ধোয়া পানির মত পায়খানার ইতিহাস	☐	☐

Study ID No.ঃ [| |]

K	W	M	C	H

Hospital ID No [| | | | | | | | |]

CRF 45

সামগ্রিকভাবে খাবার এবং দুধ গ্রহন

☐ (দুধ ১-৫ প্রতি স্কোর হিসাবে নিচে দেওয়া হল)

স্কোর	খাবার/ দুধ গ্রহন	প্রতিদিন দুধ গ্রহনের পরিমান
১	স্বাভাবিক	☐
২	হালকা কম	
৩	মাঝারী কম	
৪	খুব কম	
৫	খাবার গ্রহনে অস্বীকৃতি	

যদি দুধ খায় অনুগ্রহ করে নির্দিষ্ট করুন? ☐ বুকের দুধ খায় ☐ প্রস্তুতকৃত দুধ খায় ☐ উভয়ই

মল ত্যাগের / পায়খানার পরিমান

সময়	প্রতি ৮ ঘন্টায় পায়খানা করেছে কিনা ?	কত বার পায়খানা করেছে	কি রকম পায়খানা করেছে	কত কাপ খাবার স্যালাইন খেয়েছে
১ম আট ঘন্টা সময়	☐ না ☐ হ্যাঁ	[|]	☐	[|]
২য় আট ঘন্টা সময়	☐ না ☐ হ্যাঁ	[|]	☐	[|]
৩য় আট ঘন্টা সময়	☐ না ☐ হ্যাঁ	[|]	☐	[|]

* **পায়খানার ধরণ** *(১= শক্ত, ২= নরম, ৩ = পাতলা / পানির মত, ৪ = পাতলা পায়খানার সাথে রক্ত ৯=প্রযোজ্য নয়)*

পানি শূন্যতার পরিমান *(দিন শেষ হওয়ার ১ ঘন্টা পূর্বে)*

নিচে উল্লেখিত তালিকা অনুযায়ী শিশুর পানি শূন্যতা :<br>(০=পানি শূন্যতা নাই ১=কিছুটা পানি শূন্যতা ২= মারাত্বক পানি শূন্যতা *)<br><br>* যদি মারাত্বক পানি শূন্যতা হয় তাহলে শিশুকে গবেষণা থেকে বাদ দিন	☐

Study ID No.: | | | |

K	W	M	C	H

Hospital ID No | | | | | | | | | | |

প্রতিকূল ঘটনায় প্রযোজ্য ঔষধ সমূহ

গত পরিদর্শনের সময় দেওয়া ঔষধ সমূহ/ **প্রতিকূল ঘটনার** পরিবর্তন হয়েছে কিনা?<br>*যদি হ্যাঁ হয় তবে প্রযোজ্য লিষ্ট থেকে নতুন ঔষুধ যোগ করুন*	☐ না	☐ হ্যাঁ

CRF 46

৯ম দিন: ফলোআপ প্রশ্নপত্র
ছুটি হওয়ার ৪৮ ঘন্টার মধ্যে

ফলোআপ পরিদর্শনের তারিখ :	☐☐ দিন ☐☐☐ মাস ☐☐ বছর
পিতামাতা / বৈধ অভিভাবককে টেলিফোনে পাওয়া যাবে কিনা ?	☐ না ☐ হ্যাঁ

অন্যান্য রোগের ইতিহাস সাথে ডায়রিয়া

বর্ণনা	না	হ্যাঁ
পেট ফুলে গিয়েছে কিনা?		☐
পেটে ব্যাথা আছে কিনা?		
পেট ডাকে কিনা?		
পেটে ফাঁপা আছে কিনা?		
জ্বর আছে কিনা?	☐	☐
শারিরীক দুর্বলতা আছে কিনা?	☐	☐
বমি আছে কিনা?<br>যদি হ্যাঁ হয়, প্রতি দিন কত বার বমি হয়? ☐☐	☐	☐
অন্যান্য অস্বাভাবিকতা উপস্থিতি<br>যদি হ্যাঁ হয়, নির্দিষ্ট করুন____________________	☐	☐

Study ID No.ঃ | | | |

K	W	M	C	H

Hospital ID No

পায়খানার সাথে মলের উপস্থিতির ইতিহাস	☐	☐
পায়খানার সাথে রক্তের উপস্থিতির ইতিহাস	☐	☐
চাল ধোয়া পানির মত পায়খানার ইতিহাস	☐	☐

CRF 47

সামগ্রিকভাবে খাবার এবং দুধ গ্রহন

☐ **(দুধ ১-৫ প্রতি স্কোর হিসাবে নিচে দেয়া হল)**

স্কোর	খাবার/ দুধ গ্রহন	প্রতিদিন দুধ গ্রহনের পরিমান
১	স্বাভাবিক	☐
২	হালকা কম	
৩	মাঝারী কম	
৪	খুব কম	
৫	খাবার গ্রহনে অস্বীকৃতি	
যদি দুধ খায় অনুগ্রহ করে নির্দিষ্ট করুন?	☐ বুকের দুধ খায় ☐ প্রস্তুতকৃত দুধ খায় ☐ উভয়ই	

মল ত্যাগের / পায়খানার পরিমান

সময়	প্রতি ৮ ঘন্টায় পায়খানা করেছে কিনা ?	কত বার পায়খানা করেছে	কি রকম পায়খানা করেছে	কত কাপ খাবার স্যালাইন খেয়েছে
১ম আট ঘন্টা সময়	☐ না ☐ হ্যাঁ	☐☐	☐	☐☐
২য় আট ঘন্টা সময়	☐ না ☐ হ্যাঁ	☐☐	☐	☐☐
৩য় আট ঘন্টা সময়	☐ না ☐ হ্যাঁ	☐☐	☐	☐☐

* **পায়খানার ধরণ** *(১= শক্ত, ২= নরম, ৩ = পাতলা / পানির মত, ৪ = পাতলা পায়খানার সাথে রক্ত)*

Study ID No.ঃ | | | |

K	W	M	C	H

Hospital ID No

পানি শূন্যতার পরিমান *(দিন শেষ হওয়ার ১ ঘন্টা পূর্বে)*

নিচে উল্লেখিত তালিকা অনুযায়ী শিশুর পানি শূন্যতা :<br>(০=পানি শূন্যতা নাই ১=কিছুটা পানি শূন্যতা ২= মারাত্বক পানি শূন্যতা *)<br><br>* যদি মারাত্বক পানি শূন্যতা হয় তাহলে শিশুকে গবেষণা থেকে বাদ দিন	☐

প্রতিকূল ঘটনায় প্রযোজ্য ঔষধ সমূহ

গত পরিদর্শনের সময় দেওয়া ঔষধ সমূহ/ **প্রতিকূল ঘটনার** পরিবর্তন হয়েছে কিনা?<br><br>*যদি হ্যাঁ হয় তবে প্রযোজ্য লিষ্ট থেকে নতুন ঔষুধ যোগ করুন*	☐ না	☐ হ্যাঁ

CRF 48

১০ম দিন : ফলোআপ প্রশ্নপত্র
ছুটি হওয়ার ৭২ ঘন্টার মধ্যে

ফলোআপ পরিদর্শনের তারিখ :	☐☐ দিন ☐☐☐ মাস ☐☐ বছর
পিতামাতা / বৈধ অভিভাবককে টেলিফোনে পাওয়া যাবে কিনা ?	☐ না ☐ হ্যাঁ

ডায়রিয়া সাথে সম্পর্কিত লক্ষন সমূহ

বর্ণনা	না	হ্যাঁ
পেট ফুলে গিয়েছে কিনা?	☐	☐
পেটে ব্যাথা আছে কিনা?	☐	☐
পেট ডাকে কিনা?	☐	☐
পেটে ফাঁপা আছে কিনা?	☐	☐
জ্বর আছে কিনা?	☐	☐
শারিরীক দুর্বলতা আছে কিনা?	☐	☐
বমি আছে কিনা?<br>যদি হ্যাঁ হয়, প্রতি দিন কত বার বমি হয়? ☐☐	☐	☐

Study ID No.ঃ | | | |

K	W	M	C	H

Hospital ID No

অন্যান্য অস্বাভাবিকতা উপস্থিতি<br>যদি হ্যাঁ হয়, নির্দিষ্ট করুন______________________________	☐	☐
পায়খানার সাথে মলের *উপস্থিতির ইতিহাস*	☐	☐
পায়খানার সাথে রক্তের *উপস্থিতির ইতিহাস*	☐	☐
চাল ধোয়া পানির মত পায়খানার ইতিহাস	☐	☐

CRF 49

সামগ্রিকভাবে খাবার এবং দুধ গ্রহন

☐ **(দুধ ১-৫ প্রতি স্কোর হিসাবে নিচে দেয়া হল)**

স্কোর	খাবার/ দুধ গ্রহন	প্রতিদিন দুধ গ্রহনের পরিমান
১	স্বাভাবিক	☐
২	হালকা কম	
৩	মাঝারী কম	
৪	খুব কম	
৫	খাবার গ্রহনে অস্বীকৃতি	
যদি দুধ খায় অনুগ্রহ করে নির্দিষ্ট করুন? ☐ বুকের দুধ খায় ☐ প্রস্তুতকৃত দুধ খায় ☐ উভয়ই		

মল ত্যাগ / পায়খানার পরিমান

সময়	প্রতি ৮ ঘন্টায় পায়খানা করেছে কিনা ?	কত বার পায়খানা করেছে	কি রকম পায়খানা করেছে	কত কাপ খাবার স্যালাইন খেয়েছে
১ম আট ঘন্টা সময়	☐ না<br>☐ হ্যাঁ	☐☐	☐	☐☐
২য় আট ঘন্টা সময়	☐ না<br>☐ হ্যাঁ	☐☐	☐	☐☐
৩য় আট ঘন্টা সময়	☐ না<br>☐ হ্যাঁ	☐☐	☐	☐☐

* **পায়খানার ধরণ** *(১= শক্ত, ২= নরম, ৩ = পাতলা / পানির মত, ৪ = পাতলা পায়খানার সাথে রক্ত ৯=প্রযোজ্য নয়)*

পানি শূন্যতার পরিমান *(দিন শেষ হওয়ার ১ ঘন্টা পূর্বে)*

Study ID No.ঃ | | | |

K	W	M	C	H

Hospital ID No

নিচে উল্লেখিত তালিকা অনুযায়ী শিশুর পানি শূন্যতা :<br>(০=পানি শূন্যতা নাই ১=কিছুটা পানি শূন্যতা ২= মারাত্বক পানি শূন্যতা *)<br><br>* যদি মারাত্বক পানি শূন্যতা হয় তাহলে শিশুকে গবেষণা থেকে বাদ দিন	☐

প্রতিকূল ঘটনায় প্রযোজ্য ঔষধ সমূহ

গত পরিদর্শনের সময় দেওয়া ঔষধ সমূহ/ **প্রতিকূল ঘটনার** পরিবর্তন হয়েছে কিনা?<br>*যদি হ্যাঁ হয় তবে প্রযোজ্য লিষ্ট থেকে নতুন ঔষুধ যোগ করুন*	☐ না	☐ হ্যাঁ

CRF 50

১১ তম দিন : ফলোআপ প্রশ্নপত্র
ছুটি হওয়ার ৯৬ ঘন্টার মধ্যে

ফলোআপ পরিদর্শনের তারিখ :	দিন	মাস	বছর
পিতামাতা / বৈধ অভিভাবককে টেলিফোনে পাওয়া যাবে কিনা ?	☐ না	☐ হ্যাঁ	

ডায়রিয়া সাথে সম্পর্কিত লক্ষন সমূহ

বর্ণনা	না	হ্যাঁ
পেট ফুলে গিয়েছে কিনা?	☐	☐
পেটে ব্যাথা আছে কিনা?	☐	☐
পেট ডাকে কিনা?	☐	☐
পেটে ফাঁপা আছে কিনা?	☐	☐
জ্বর আছে কিনা?	☐	☐
শারিরীক দুর্বলতা আছে কিনা?	☐	☐
বমি আছে কিনা?<br>যদি হ্যাঁ হয়, প্রতি দিন কত বার বমি হয়? ☐☐	☐	☐
অন্যান্য অস্বাভাবিকতা উপস্থিতি<br>যদি হ্যাঁ হয়, নির্দিষ্ট করুন______________________________	☐	☐

Study ID No.ঃ | | | |

K	W	M	C	H

Hospital ID No

পায়খানার সাথে মলের *উপস্থিতির ইতিহাস*	☐	☐
পায়খানার সাথে রক্তের *উপস্থিতির ইতিহাস*	☐	☐
চাল ধোয়া পানির মত পায়খানার ইতিহাস	☐	☐

CRF 51

সামগ্রিকভাবে খাবার এবং দুধ গ্রহন

☐ **(দুধ ১-৫ প্রতি স্কোর হিসাবে নিচে দেয়া হল)**

স্কোর	খাবার/ দুধ গ্রহন	প্রতিদিন দুধ গ্রহনের পরিমান
১	স্বাভাবিক	☐
২	হালকা কম	
৩	মাঝারী কম	
৪	খুব কম	
৫	খাবার গ্রহনে অস্বীকৃতি	
যদি দুধ খায় অনুগ্রহ করে নির্দিষ্ট করুন? ☐ বুকের দুধ খায় ☐ প্রস্তুতকৃত দুধ খায় ☐ উভয়ই		

মল ত্যাগ / পায়খানার পরিমান

সময়	প্রতি ৮ ঘন্টায় পায়খানা করেছে কিনা ?	কত বার পায়খানা করেছে	কি রকম পায়খানা করেছে	কত কাপ খাবার স্যালাইন খেয়েছে
১ম আট ঘন্টা সময়	☐ না<br>☐ হ্যাঁ	☐☐	☐	☐☐
২য় আট ঘন্টা সময়	☐ না<br>☐ হ্যাঁ	☐☐	☐	☐☐
৩য় আট ঘন্টা সময়	☐ না<br>☐ হ্যাঁ	☐☐	☐	☐☐
* **পায়খানার ধরণ** *(১= শক্ত, ২= নরম, ৩ = পাতলা / পানির মত, ৪ = পাতলা পায়খানার সাথে রক্ত ৯=প্রযোজ্য নয়)*				

পানি শূন্যতার পরিমান *(দিন শেষ হওয়ার ১ ঘন্টা পূর্বে)*

Study ID No.ঃ | | | |

K	W	M	C	H

Hospital ID No | | | | | | | | | | |

নিচে উল্লেখিত তালিকা অনুযায়ী শিশুর পানি শূন্যতা :<br>(০=পানি শূন্যতা নাই ১=কিছুটা পানি শূন্যতা ২= মারাত্বক পানি শূন্যতা *)<br><br>* যদি মারাত্বক পানি শূন্যতা হয় তাহলে শিশুকে গবেষণা থেকে বাদ দিন	☐

প্রতিকূল ঘটনায় প্রযোজ্য ঔষধ সমূহ

গত পরিদর্শনের সময় দেওয়া ঔষধ সমূহ/ **প্রতিকূল ঘটনার** পরিবর্তন হয়েছে কিনা?<br>*যদি হ্যাঁ হয় তবে প্রযোজ্য লিষ্ট থেকে নতুন ঔষুধ যোগ করুন*	☐ না	☐ হ্যাঁ

CRF 52

১২ তম দিন : ফলোআপ প্রশ্নপত্র
ছুটি হওয়ার ১২০ ঘন্টার মধ্যে

ফলোআপ পরিদর্শনের তারিখ :	☐☐ দিন	☐☐☐ মাস	☐☐ বছর
পিতামাতা / বৈধ অভিভাবককে টেলিফোনে পাওয়া যাবে কিনা ?	☐ না	☐ হ্যাঁ	

ডায়রিয়া সাথে সম্পর্কিত লক্ষন সমূহ

বর্ণনা	না	হ্যাঁ
পেট ফুলে গিয়েছে কিনা?	☐	☐
পেটে ব্যাথা আছে কিনা?	☐	☐
পেট ডাকে কিনা?	☐	☐
পেটে ফাঁপা আছে কিনা?	☐	☐
জ্বর আছে কিনা?	☐	☐
শারিরীক দুর্বলতা আছে কিনা?	☐	☐
বমি আছে কিনা?<br>যদি হ্যাঁ হয়, প্রতি দিন কত বার বমি হয়? ☐☐	☐	☐
অন্যান্য অস্বাভাবিকতা উপস্থিতি<br>যদি হ্যাঁ হয়, নির্দিষ্ট করুন______________________________	☐	☐

Study ID No.ঃ | | | |

K	W	M	C	H

Hospital ID No

পায়খানার সাথে মলের *উপস্থিতির ইতিহাস*	☐	☐
পায়খানার সাথে রক্তের *উপস্থিতির ইতিহাস*	☐	☐
চাল ধোয়া পানির মত পায়খানার ইতিহাস	☐	☐

CRF 53

সামগ্রিকভাবে খাবার এবং দুধ গ্রহন

☐ **(দুধ ১-৫ প্রতি স্কোর হিসাবে নিচে দেয়া হল)**

স্কোর	খাবার/ দুধ গ্রহন	প্রতিদিন দুধ গ্রহনের পরিমান
১	স্বাভাবিক	☐
২	হালকা কম	
৩	মাঝারী কম	
৪	খুব কম	
৫	খাবার গ্রহনে অস্বীকৃতি	
যদি দুধ খায় অনুগ্রহ করে নির্দিষ্ট করুন? ☐ বুকের দুধ খায় ☐ প্রস্তুতকৃত দুধ খায় ☐ উভয়ই		

মল ত্যাগ / পায়খানার পরিমান

সময়	প্রতি ৮ ঘন্টায় পায়খানা করেছে কিনা ?	কত বার পায়খানা করেছে	কি রকম পায়খানা করেছে	কত কাপ খাবার স্যালাইন খেয়েছে
১ম আট ঘন্টা সময়	☐ না<br>☐ হ্যাঁ	☐☐	☐	☐☐
২য় আট ঘন্টা সময়	☐ না<br>☐ হ্যাঁ	☐☐	☐	☐☐
৩য় আট ঘন্টা সময়	☐ না<br>☐ হ্যাঁ	☐☐	☐	☐☐
* **পায়খানার ধরণ** *(১= শক্ত, ২= নরম, ৩ = পাতলা / পানির মত, ৪ = পাতলা পায়খানার সাথে রক্ত ৯=প্রযোজ্য নয়)*				

পানি শূন্যতার পরিমান *(দিন শেষ হওয়ার ১ ঘন্টা পূর্বে)*

Study ID No.: | | | |

K	W	M	C	H

Hospital ID No

নিচে উল্লেখিত তালিকা অনুযায়ী শিশুর পানি শূন্যতা :<br>(০=পানি শূন্যতা নাই ১=কিছুটা পানি শূন্যতা ২= মারাত্বক পানি শূন্যতা *)<br><br>* যদি মারাত্বক পানি শূন্যতা হয় তাহলে শিশুকে গবেষণা থেকে বাদ দিন	☐

প্রতিকূল ঘটনায় প্রযোজ্য ঔষধ সমূহ

গত পরিদর্শনের সময় দেওয়া ঔষধ সমূহ/ **প্রতিকূল ঘটনার** পরিবর্তন হয়েছে কিনা?<br>*যদি হ্যাঁ হয় তবে প্রযোজ্য লিষ্ট থেকে নতুন ঔষুধ যোগ করুন*	☐ না	☐ হ্যাঁ

CRF 54

১৩ তম দিন : ফলোআপ প্রশ্নপত্র
ছুটি হওয়ার ১৪৪ ঘন্টার মধ্যে

ফলোআপ পরিদর্শনের তারিখ : ☐☐ ☐☐☐ ☐☐
দিন মাস বছর

পিতামাতা / বৈধ অভিভাবককে টেলিফোনে পাওয়া যাবে কিনা ? ☐ না ☐ হ্যাঁ

ডায়রিয়া সাথে সম্পর্কিত লক্ষন সমূহ

বর্ণনা	না	হ্যাঁ
পেট ফুলে গিয়েছে কিনা?	☐	☐
পেটে ব্যাথা আছে কিনা?	☐	☐
পেট ডাকে কিনা?	☐	☐
পেটে ফাঁপা আছে কিনা?	☐	☐
জ্বর আছে কিনা?	☐	☐
শারিরীক দুর্বলতা আছে কিনা?	☐	☐
বমি আছে কিনা?<br>যদি হ্যাঁ হয়, প্রতি দিন কত বার বমি হয়? ☐☐	☐	☐
অন্যান্য অস্বাভাবিকতা উপস্থিতি<br>যদি হ্যাঁ হয়, নির্দিষ্ট করুন_______________	☐	☐

Study ID No.ঃ | | | |

K	W	M	C	H

Hospital ID No

পায়খানার সাথে মলের *উপস্থিতির ইতিহাস*	☐	☐
পায়খানার সাথে রক্তের *উপস্থিতির ইতিহাস*	☐	☐
চাল ধোয়া পানির মত পায়খানার ইতিহাস	☐	☐

CRF 55

সামগ্রিকভাবে খাবার এবং দুধ গ্রহন

☐ **(দুধ ১-৫ প্রতি স্কোর হিসাবে নিচে দেয়া হল)**

স্কোর	খাবার/ দুধ গ্রহন	প্রতিদিন দুধ গ্রহনের পরিমান
১	স্বাভাবিক	☐
২	হালকা কম	
৩	মাঝারী কম	
৪	খুব কম	
৫	খাবার গ্রহনে অস্বীকৃতি	

যদি দুধ খায় অনুগ্রহ করে নির্দিষ্ট করুন? ☐ বুকের দুধ খায় ☐ প্রস্তুতকৃত দুধ খায় ☐ উভয়ই

মল ত্যাগ / পায়খানার পরিমান

সময়	প্রতি ৮ ঘন্টায় পায়খানা করেছে কিনা ?	কত বার পায়খানা করেছে	কি রকম পায়খানা করেছে	কত কাপ খাবার স্যালাইন খেয়েছে
১ম আট ঘন্টা সময়	☐ না<br>☐ হ্যাঁ	☐☐	☐	☐☐
২য় আট ঘন্টা সময়	☐ না<br>☐ হ্যাঁ	☐☐	☐	☐☐

☐

Study ID No.ঃ | | | |

K	W	M	C	H

Hospital ID No

৩য় আট ঘন্টা সময়	☐ না<br>☐ হ্যাঁ	☐☐		☐☐
* **পায়খানার ধরণ** *(১= শক্ত, ২= নরম, ৩ = পাতলা / পানির মত, ৪ = পাতলা পায়খানার সাথে রক্ত ৯=প্রযোজ্য নয়)*				

পানি শূন্যতার পরিমান *(দিন শেষ হওয়ার ১ ঘন্টা পূর্বে)*

নিচে উল্লেখিত তালিকা অনুযায়ী শিশুর পানি শূন্যতা :<br>(০=পানি শূন্যতা নাই ১=কিছুটা পানি শূন্যতা ২= মারাত্বক পানি শূন্যতা *)<br><br>* যদি মারাত্বক পানি শূন্যতা হয় তাহলে শিশুকে গবেষণা থেকে বাদ দিন	☐

প্রতিকূল ঘটনায় প্রযোজ্য ঔষধ সমূহ

গত পরিদর্শনের সময় দেওয়া ঔষধ সমূহ/ **প্রতিকূল ঘটনার** পরিবর্তন হয়েছে কিনা?<br>*যদি হ্যাঁ হয় তবে প্রযোজ্য লিষ্ট থেকে নতুন ঔষুধ যোগ করুন*	☐ না	☐ হ্যাঁ

CRF 56

১৪ তম দিন : ফলোআপ প্রশ্নপত্র
ছুটি হওয়ার ১৬৮ ঘন্টার মধ্যে

ফলোআপ পরিদর্শনের তারিখ :	☐☐ দিন ☐☐☐ মাস ☐☐ বছর
পিতামাতা / বৈধ অভিভাবককে টেলিফোনে পাওয়া যাবে কিনা ?	☐ না ☐ হ্যাঁ

অত্যাবশ্যকীয় লক্ষণ

**Variables**<br>**সময় গণনা**<br>(প্রতি ৮ ঘন্টা পর পর গণনা করুন)	**ওজন (কেজি)**<br>[শুধু মাত্র সকালে]	**হৃদ স্পন্দন** (প্রতি মিনিটে)	**শ্বাসের গতি**(প্রতি মিনিট)	**কান/ কপালের (তাপমাত্রা)**	**ব্লাড প্রেসার. Systolic** (mmHg)<br>[শুধু মাত্র সকালে]	**ব্লাড প্রেসার. Diastolic** (mmHg)<br>[শুধু মাত্র সকালে]
১ম আট ঘন্টা সময়						
২য় আট ঘন্টা সময়						
৩য় আট ঘন্টা সময়						
যদি কোন reading স্বাভাবিক পরিসীমা	☐ না	☐ না	☐ না	☐ না	☐ না	☐ না

Study ID No.ঃ | | | |

K	W	M	C	H

Hospital ID No

অতিক্রম করে?	☐ হ্যাঁ	☐ হ্যাঁ	☐ হ্যাঁ	☐ হ্যাঁ	☐ হ্যাঁ	☐ হ্যাঁ
যদি হ্যাঁ হয় তবে, reading clinically Significant কিনা?*	☐ না<br>☐ হ্যাঁ	☐ না<br>☐ হ্যাঁ	☐ না<br>☐ হ্যাঁ	☐ না<br>☐ হ্যাঁ	☐ না<br>☐ হ্যাঁ	☐ না<br>☐ হ্যাঁ
**যদি reading Clinically Significant হয় তবে প্রয়োজন হলে অনুগ্রহ করে Adverse Event Form পূরণ করুন]*						

CRF 57

ডায়রিয়া সাথে সম্পর্কিত লক্ষন সমূহ

বর্ণনা	না	হ্যাঁ
পেট ফুলে গিয়েছে কিনা?	☐	☐
পেটে ব্যাথা আছে কিনা?	☐	☐
পেট ডাকে কিনা?	☐	☐
পেটে ফাঁপা আছে কিনা?	☐	☐
জ্বর আছে কিনা?	☐	☐
শারিরীক দুর্বলতা আছে কিনা?	☐	☐
বমি আছে কিনা?<br>যদি হ্যাঁ হয়, প্রতি দিন কত বার বমি হয়? ☐☐	☐	☐
অন্যান্য অস্বাভাবিকতা উপস্থিতি<br>যদি হ্যাঁ হয়, নির্দিষ্ট করুন____________________________	☐	☐
পায়খানার সাথে মলের *উপস্থিতির ইতিহাস*	☐	☐
পায়খানার সাথে রক্তের *উপস্থিতির ইতিহাস*	☐	☐
চাল ধোয়া পানির মত পায়খানার ইতিহাস	☐	☐

সামগ্রিকভাবে খাবার এবং দুধ গ্রহন

☐ **(দুধ ১-৫ প্রতি স্কোর হিসাবে নিচে দেয়া হল)**

স্কোর	খাবার/ দুধ গ্রহন	প্রতিদিন দুধ গ্রহনের পরিমান
১	স্বাভাবিক	

Study ID No.: | | | |

K	W	M	C	H

Hospital ID No

২	হালকা কম	
৩	মাঝারী কম	
৪	খুব কম	
৫	খাবার গ্রহনে অস্বীকৃতি	
যদি দুধ খায় অনুগ্রহ করে নির্দিষ্ট করুন? ☐ বুকের দুধ খায় ☐ প্রস্তুতকৃত দুধ খায় ☐ উভয়ই		

CRF 58

মল ত্যাগ / পায়খানার পরিমান

সময়	প্রতি ৮ ঘন্টায় পায়খানা করেছে কিনা ?	কত বার পায়খানা করেছে	কি রকম পায়খানা করেছে	কত কাপ খাবার স্যালাইন খেয়েছে
১ম আট ঘন্টা সময়	☐ না ☐ হ্যাঁ	☐☐	☐	☐☐
২য় আট ঘন্টা সময়	☐ না ☐ হ্যাঁ	☐☐	☐	☐☐
৩য় আট ঘন্টা সময়	☐ না ☐ হ্যাঁ	☐☐	☐	☐☐
* **পায়খানার ধরণ** *(১= শক্ত, ২= নরম, ৩ = পাতলা / পানির মত, ৪ = পাতলা পায়খানার সাথে রক্ত ৯=প্রযোজ্য নয়)*				

পানি শূন্যতার পরিমান *(দিন শেষ হওয়ার ১ ঘন্টা পূর্বে)*

নিচে উল্লেখিত তালিকা অনুযায়ী শিশুর পানি শূন্যতা :<br>(০=পানি শূন্যতা নাই ১=কিছুটা পানি শূন্যতা ২= মারাত্বক পানি শূন্যতা *)<br><br>* যদি মারাত্বক পানি শূন্যতা হয় তাহলে শিশুকে গবেষণা থেকে বাদ দিন	☐

প্রতিকূল ঘটনায় প্রযোজ্য ঔষধ সমূহ

গত পরিদর্শনের সময় দেওয়া ঔষধ সমূহ/ **প্রতিকূল ঘটনার** পরিবর্তন হয়েছে কিনা?<br><br>*যদি হ্যাঁ হয় তবে প্রযোজ্য লিষ্ট থেকে নতুন ঔষুধ যোগ করুন*	☐ না	☐ হ্যাঁ

ডায়রিয়া বন্ধ হওয়া

হাসপাতালে ভর্তি থাকাকালীন ডায়রিয়া বন্ধ হয়েছে কিনা?	☐ না	☐ হ্যাঁ
☐☐ - ☐☐☐ - ☐☐ ☐☐ - ☐☐		

Study ID No.ঃ | | | |

K	W	M	C	H

Hospital ID No

যদি হ্যাঁ হয়, ডায়রিয়া বন্ধ হওয়ার তারিখ : : বন্ধ হওয়ার সময় : :

দিন মাস বছর ঘন্টা মিনিট

যদি না হয়, বাড়ীতে ডায়রিয়া বন্ধ হওয়ার তারিখ : বন্ধ হওয়ার সময় : :

দিন মাস বছর ঘন্টা মিনিট

CRF 59

চূড়ান্ত মূল্যায়ন ফর্ম

অংশগ্রহন শেষ হওয়ার তারিখ:

গবেষণার দিন তারিখ

দিন মাস বছর

গবেষণার জিনিসপত্র/ ঔষধ গ্রহনের শেষ দিন :

গবেষণার দিন তারিখ :

দিন মাস বছর

হাসপাতাল হতে ছুটি হওয়ার তারিখ:

তারিখ:

ঘন্টা : মিনিট

শিশুটি কি গবেষণা সমাপ্ত করেছে না ☐ হ্যাঁ ☐

যদি হ্যাঁ হয়, সমাপ্ত হওয়ার তারিখ

দিন মাস বছর

যদি শিশুটি গবেষনা সমাপ্ত না করে তাহলে প্রত্যাহারের কারন *?(একটি মাত্র বক্সে টিক চিহ্ন দিন)*

- ☐ সম্মতি প্রত্যাহার
- ☐ ফলোআপ সম্ভব হয়নি *(appointment ব্যর্থ)*
- ☐ প্রত্যাহার করা হবে যদি পূর্বে উল্লেখিত অন্তভর্ক্তি শর্তাবলী ভঙ্গ হয় *(নির্দিষ্ট করুন)*________________
- ☐ প্রটোকল অনুযায়ী প্রত্যাহার *(নির্দিষ্ট করুন)*____
- ☐ প্রয়োজনীয় উন্নতির অভাবে হাসপাতালে অতিরিক্ত ভর্তি থাকা *(নির্দিষ্ট করুন)*

____

- ☐ প্রতিকূলতার জন্য প্রত্যাহার .

যদি প্রতিকূল অবস্থার কোন লক্ষন মারাত্বক হয় বা মৃত্যু হয় তবে চিকিৎসকে অবহিত করুন এবং তাৎক্ষনিক ভাবে ব্যবস্থা নিন.

মৃত্যু: মৃত্যুর তারিখ :

[illegible]

Study ID No.: | | | |

K	W	M	C	H

Hospital ID No

CRF 60

বিচ্যুতিকরন সূচি

CRF পৃষ্টা নম্বর	পরিদর্শন এবং CRF তারিখ পদ	বিচ্যুতির বর্ণনা	EC- কে জানানো
			☐ না ☐ হ্যাঁ
			☐ না ☐ হ্যাঁ
			☐ না ☐ হ্যাঁ
			☐ না ☐ হ্যাঁ
			☐ না ☐ হ্যাঁ
			☐ না ☐ হ্যাঁ
			☐ না ☐ হ্যাঁ
			☐ না ☐ হ্যাঁ
			☐ না ☐ হ্যাঁ
			☐ না ☐ হ্যাঁ
