# Supplementary material for: Digestive tolerability and acceptability of Fibersol-2 in healthy and diarrheal children 1–3 years old at a rural facility, Bangladesh: Results from a four arm exploratory study
Source: PLoS One. 2022 Sep 19;17(9):e0274302. doi: 10.1371/journal.pone.0274302 (PMC9484693; doi:10.1371/journal.pone.0274302)
Supplement: Attachment — Submitted filename: Response to reviewers_Shahid ASMSB_15.09.2021.docx [file pone.0274302.s006.docx]

Date: 15^th^ September, 2021

To

Ivan D. Florez, MD, MSc, PhD

Academic Editor, PLOS ONE

From:

Dr. Abu Sadat Mohammad Sayeem Bin Shahid

Corresponding Author

**Subject:** Response to the comments of the reviewers of PLOS ONE on manuscript Ref: PONE-D-21-01326 titled “Digestive tolerability and acceptability of Fibersol-2 in healthy and diarrheal children 1-3 years old at a rural facility, Bangladesh: results from a four arm exploratory study**.**”

Dear Ivan D. Florez,

Thank you for evaluating our manuscript and providing us with the opportunity to submit the revised manuscript after addressing academic editor’s and reviewers comments. We also express our sincere thanks to them for evaluating our manuscript. We are sending both the track change and clean versions of the manuscript that highlights the changes we have made from the previous version. We are also attaching this letter outlining a point-by-point response to the each point kindly raised by the respected academic editor and respected reviewers.

We hope that our response will be appropriate to qualify the manuscript for publication in your well-reputed journal.

We look forward to kindly hearing from you.

Thank you.

**Journal Requirements:**

1. Please ensure that your manuscript meets PLOS ONE's style requirements, including those for file naming.

**Response:** Thank you. It has been revised accordingly.

2. Please include additional information regarding the survey or questionnaire used in the study and ensure that you have provided sufficient details that others could replicate the analyses. For instance, if you developed a questionnaire as part of this study and it is not under a copyright more restrictive than CC-BY, please include a copy, in both the original language and English, as Supporting Information.

**Response:** Thank you. It has been uploaded as Supporting Information.

3. Please provide a sample size and power calculation in the Methods, or discuss the reasons for not performing one before study initiation.

**Response:** Thank you for the comment. As this was an exploratory study, we purposefully enrolled 30 healthy and 30 diarrheal children to see the digestive tolerability and acceptability of Fibersol-2 to find out the suitable dose for the proposed randomized trial (line no.141-142 in the clean version).

4. Please provide additional details about your study design in your Methods, including randomisation methods and outcomes measured.

**Response:** Thank you for the comment. As it was an exploratory study, there was no randomization and masking. We measured the episodes of abdominal symptoms, such as pain, distension, rumbling and bloating during the intervention and post intervention period for both healthy and diarrheal children for both the low (2.5g) and high (5 g) doses to see the outcomes (line no. 137-139 in the clean version).

5. In your Methods section, please provide additional information about the participant recruitment method and the demographic details of your participants. Please ensure you have provided sufficient details to replicate the analyses such as:
a) the recruitment date range (month and year)

**Response:** Thanks for the suggestion. It has already been mentioned under study design (line no. 108-109 in the clean version).

b) a table of relevant demographic details

**Response:** Thanks for the suggestion. It has been incorporated in Table S1.

c) a statement as to whether your sample can be considered representative of a larger population

**Response:** Thank you for the suggestion. It has been incorporated in line no.142-143 (clean version).

d) a description of how participants were recruited

**Response:** Thank you for the suggestion. It has been incorporated in line no.144-147 (clean version) and also Figure 1.

6. We note that you have indicated that data from this study are available upon request. PLOS only allows data to be available upon request if there are legal or ethical restrictions on sharing data publicly.

**Response:** Thank you for the comment. Our data contain a lot of personal information where we de-identified them during analysis. As per institutional policy data with personal information will be remained with our Research Administration (RA) due to ethical constraint and if someone wants to make the availability of the de-identified data, he/she may kindly communicate with the head of RA (aahmed@)icddrb.org).

7. Thank you for stating the following in the Financial Disclosure section:

'This research study was funded by Matsutani Chemical Industry Company Limited, Japan on behalf of ADM/Matsutani LLC, USA. The International Centre for Diarrhoeal Disease Research, Bangladesh, receives unrestricted support from the Government of the People's Republic of Bangladesh, Global Affairs Canada, the Swedish International Development Cooperation Agency and the UK Department for International Development.'

We note that one or more of the authors have an affiliation to the commercial funders of this research study, Matsutani Chemical Industry Co. Ltd

a. Please provide an amended Funding Statement declaring this commercial affiliation, as well as a statement regarding the Role of Funders in your study. If the funding organization did not play a role in the study design, data collection and analysis, decision to publish, or preparation of the manuscript and only provided financial support in the form of authors' salaries and/or research materials, please review your statements relating to the author contributions, and ensure you have specifically and accurately indicated the role(s) that these authors had in your study. You can update author roles in the Author Contributions section of the online submission form.

Please also include the following statement within your amended Funding Statement.

“The funder provided support in the form of salaries for authors [insert relevant initials], but did not have any additional role in the study design, data collection and analysis, decision to publish, or preparation of the manuscript. The specific roles of these authors are articulated in the ‘author contributions’ section.”

If your commercial affiliation did play a role in your study, please state and explain this role within your updated Funding Statement.

**Response:** Thank you for the suggestions. It has been incorporated in line no.200-207 (clean version).

b. Please also provide an updated Competing Interests Statement declaring this commercial affiliation along with any other relevant declarations relating to employment, consultancy, patents, products in development, or marketed products, etc.

**Response:** Thank you for the suggestion. It has been incorporated in line no. 289-291 (clean version).

c. Please include both an updated Funding Statement and Competing Interests Statement in your cover letter. We will change the online submission form on your behalf.

**Response:** Thank you for the suggestion. It has been incorporated accordingly.

8. Please include a separate caption for each figure in your manuscript.

**Response:** Thank you for the suggestion. It has been incorporated accordingly in line no.146 (clean version)

9. Please ensure that you refer to Figure 1 in your text as, if accepted, production will need this reference to link the reader to the figure.

**Response:** Thank you for the suggestion. It has been incorporated accordingly in line no. 146 (clean version)

10. Please include your tables 1-6 as part of your main manuscript and remove the individual files. Please note that supplementary tables should remain as separate "supporting information" files.

**Response:** Thank you for the suggestion. Tables have been incorporated in the revised manuscript as well as supplementary tables have been uploaded as supporting information.

11. Please include captions for your Supporting Information files at the end of your manuscript, and update any in-text citations to match accordingly.

**Response:** Thank you for the suggestion. It has been incorporated in line no.355-362 (clean version).

**Review Comments to the Author**

**Reviewer #1:**

This exploratory Phase 1/2 study examined the digestive tolerability and acceptability of two different doses of Fibersol-2 (a fermentable, non-viscous dextrin) to relieve abdominal symptoms and improve stool consistency in both healthy children and children with diarrhea, 1-3 years old, in Bangladesh. 2.5g and 5 g of Fibersol-2 were administered twice daily to 60 children each in both groups, and there was no placebo group. This study is part of a registered efficacy trial.

**Response:** Thanks for the overall positive comments.

Comments:

Abstract:

l. 40-41 “No adverse events…were observed in healthy children, except for one…” Probably more accurate to state “Only one of the healthy children experienced any adverse events…”

**Response:** Thank you for the suggestion. It has been revised in line no. 44-46 (clean version).

ll. 46-47 “…devoid of any adverse events” should more correctly stated as “minimal adverse events,” since the authors report one child who experienced an adverse event. One does not equal none.

**Response:** Thank you for the suggestion. It has been revised accordingly in line no.51 (clean version).

**Statistical analysis**

l. 187 Medians should be reported with their interquartile ranges, either in place of, or in addition to the range. Because the range is sensitive to outliers, the IQR gives a better summary of where the bulk of the data lie.

**Response:** Thank you for the suggestion. It has been incorporated in line no.189 (clean version).

ll. 189-90 Please specify which non-parametric tests were used.

**Response:** Thank you for the comment. It has been incorporated in line no.192 (clean version).

This is a four-arm study, but the authors have not taken advantage of this fact in the descriptive statistics or in the analyses. Rather than do two separate sets of analyses, comparing low vs. high dose within the groups of children with and without diarrhea separately, a more appropriate analysis would summarize data and perform comparisons across all four groups (Healthy/high dose, healthy/low dose, diarrheal high dose/diarrheal low dose) simultaneously. This could be done with two-way ANOVA for continuous variables (with dose as one factor and diarrheal group as the other factor) and chi square/Fisher’s exact test for categorical variables. This would allow for comparison across both the dosage and the health status of the children, which is not possible if children with and without diarrhea are examined separately. This would also eliminate the confusion that runs throughout the paper as to which groups are being compared at a given time; sometimes “groups” refers to dosage groups, at other times, to health status groups.
The authors should note that with such a small study, a finding of “no difference” could be due to a lack of power to detect a difference of a given size.

**Response:** Thank you for the suggestions. It has been revised in Tables 1, S1 and S2.

**Results**

See above for a suggested re-working of the analyses.

Also note that in Table S1, the comparison of wealth status between the two groups is incorrect; this calls for a Fisher’s exact test of the counts in each quintile by the groups being compared. In the table as currently reported, this represents a 5x2 table, and the test has a p-value of 0.13, indicating that there is no difference in wealth distribution between the two groups. In addition, neither family size nor number of sleeping rooms appears to be normally distributed. When this is the case, data should be summarized with medians and IQRs and compared with Wilcoxon rank sum tests.

**Response:** Thanks for the suggestions. It has been revised accordingly in Table S1.

Tables S2 and S3.
I am not sure why the authors present data on the characteristics of the children at baseline and at Day 21. This is not an efficacy study; if the authors wish to report on adverse events involving, e.g., weight loss over the one-week intervention period, they should look at changes and not aggregate before and after measurements. I would not expect that many of these measurements would change as a result of a one-week intervention, however, and the stated intention of the study is not efficacy, but tolerability and acceptability.

**Response:** Thanks for your valuable suggestions. It has been revised in Table S2.

Tables 3 and 4. The data in these tables is primary to the goal of the study and could be presented in a more informative manner. E.g., it is probably not necessary to present results by day; summarizing by study period across the four groups would provide more easily digestible information (and even with this level of detail, it is not possible to tell whether the same or a different child suffered from given symptoms in the different periods). I’m not sure why pre-intervention symptoms are presented if the goal is to assess tolerability of the treatment during the treatment period. If the authors are not going to do an analysis in which they adjust for pre-intervention characteristics of individual children, and eligibility characteristics of the children have eliminated any serious conditions, these data are not, I think, necessary.

**Response:** Thanks for your valuable suggestions. It has been revised in Table 2.

Conclusions

The authors purport to have shown the safety and tolerability of Fibrosol-2; it would seem that a large sample of children is not needed to confirm this, but to have sufficient power to examine efficacy outcomes.

**Response:** Thank you for the comment and we completely agree. The statement has been revised in line no. 269-70 (clean version).

The paper would also benefit from editing to correct grammatical and sentence structure errors.

**Response:** Thanks for the suggestion. We tried to improve the grammatical and sentence structure errors in the revised version.

**Reviewer #2:**

Interesting study about dextrin compound - fibersol2 use in healthy children and children with diarrhea (1-3 years of age). Study is executed well with a control and study population and with an N of 60 however not a randomized trial. Well written manuscript.

**Response:** Thank you for overall positive comments.
