# Supplementary material for: Digestive tolerability and acceptability of Fibersol-2 in healthy and diarrheal children 1–3 years old at a rural facility, Bangladesh: Results from a four arm exploratory study
Source: PLoS One. 2022 Sep 19;17(9):e0274302. doi: 10.1371/journal.pone.0274302 (PMC9484693; doi:10.1371/journal.pone.0274302)
Supplement: Attachment — Submitted filename: Response to Reviewers.docx [file pone.0274302.s007.docx]

Date: 19 December, 2021

To

Ivan D. Florez, MD, MSc, PhD

Academic Editor, PLOS ONE

From:

Dr. Abu Sadat Mohammad Sayeem Bin Shahid

Corresponding Author

**Subject:** Response to the comments of the academic editor and reviewer of PLOS ONE on manuscript Ref: PONE-D-21-01326R1 titled “Digestive tolerability and acceptability of Fibersol-2 in healthy and diarrheal children 1-3 years old at a rural facility, Bangladesh: results from a four arm exploratory study**.**”

Dear Ivan D. Florez,

Thank you for evaluating our manuscript and providing us with the opportunity to submit the revised manuscript after addressing respected academic editor’s and reviewer’s comments. We also express our sincere thanks to him/her for evaluating our manuscript. We are sending both the track change and clean versions of the manuscript that highlights the changes we have made from the previous version. We are also attaching this letter outlining a point-by-point response to each point kindly raised by the respected reviewer.

We hope that our response will be appropriate to qualify the manuscript for publication in your well-reputed journal.

We look forward to kindly hearing from you.

Thank you.

**Academic Editor**

The reviewer has raised key elements that need to be addressed. Particularly, they state that the concept of efficacy is not clear. Please address their comments and provide a point by point response.

**Response:** Thank you. We have tried to address respected reviewer’s comments in the revised version of the manuscript.

**Reviewer #1:**

I think that the authors, while purporting to have tolerability and feasibility as their primary outcomes, have confused these concepts with efficacy. For tolerability and feasibility, the important question is whether or not children had an unacceptable reaction to the Fibersol, and whether this was associated with health state or dosage. Therefore, the period that is of most importance is the period during which the children are taking the Fibersol (not before, or after, when it is no longer being given to them). Looking at changes in/alleviation of symptoms as a result of taking Fibersol is actually a different question, and would require comparing pre-intervention symptoms to end of intervention symptoms.

**Response:** Thank you for the overall comment. We have now re-analyzed the data and presented as per suggestion of the respected reviewer in tables 2, 3 and 4.

I would suggest a rehaul of the paper. The authors stand a better chance of making conclusions about tolerability than they do about efficacy. This is not an efficacy analysis, nor was it powered to be. So what about the responses to the Fibersol during the time it was taken? Is it actually feasible and tolerable as the authors conclude? Looking at Table 2, the authors observed the following:

Abdominal pain during intervention: 20% of D/L kids; 7% of D/H kids
Abdominal distention: 27% of D/L kids; 20% of D/H kids
Abdominal rumbling: 27% of D/L kids; 27% of D/H kids
Abdominal bloating: 40% of D/L kids; 47% of D/H kids
Vomiting: 33% of H/H, D/L, D/H kids
Fever: 7% of H/L and H/H kids, 27% of D/L and D/H kids

I would argue that these are some alarming side effects in children so young, and most of them (except for vomiting) occur much more frequently in the diarrheal group. I am not sure how the authors conclude that “Regarding the digestive tolerability, there were some symptoms observed for both [diarrheal] groups during the study period.” Isn’t that the crux of the paper?

I would suggest the following steps:
1. Look at each outcome (as listed above), comparing WITHIN groups across dosages. For no one does the dosage appear to come into play.
2. Because of the fact that dosage doesn’t matter, compare healthy to diarrheal kids, who have a much lower tolerance for Fibersol.
3. Conclude that it doesn’t appear, from your evidence, that Fibersol is tolerable for children with diarrhea.

If you compare the different dosages in healthy kids, there are no differences in symptoms while taking Fibersol. If you compare the different dosages in diarrheal kids, there are no differences in symptoms while taking Fibersol. If you lump dosages together, then you get the following, comparing healthy to diarrheal children (at any dose):
Abdominal pain: p=0.09
Abdominal distension: p=0.01
Abdominal rumbling: p=0.005
Abdominal bloating: p<0.001
Vomiting: p=0.37 (no difference, but bad in both groups!)
Fever: p=0.08.

This strongly suggests that healthy children tolerate the Fibersol much better than the diarrheal children. The fact that symptoms lessen after the treatment has been stopped doesn’t prove the treatment is efficacious, if the treatment caused the symptoms in the first place.

I believe that the authors should re-think their analysis in terms of what question they are trying to answer, and re-evaluate their conclusions.

**Response:** Thank you for the valuable comments and suggestions. We have now reanalyzed the data and revised the manuscript accordingly (please see line no. 49-57, page no. 3; line no. 236-245, page no.11; and line no. 268-280, page no.13 in track change version of the manuscript).

**Minor comments:**

summarize diarrhea duration with medians and IQRs, not means and sds. These do not appear to be normally distributed.

**Response:** Thank you for the suggestion. We have revised accordingly (please see line no. 43-44, page no 2-3; line no. 251-252, page no.12; line no. 265-266, page no.13 in track change version of the manuscript and table S2 in supporting information).
