# Supplementary material for: Digestive tolerability and acceptability of Fibersol-2 in healthy and diarrheal children 1–3 years old at a rural facility, Bangladesh: Results from a four arm exploratory study
Source: PLoS One. 2022 Sep 19;17(9):e0274302. doi: 10.1371/journal.pone.0274302 (PMC9484693; doi:10.1371/journal.pone.0274302)
Supplement: Attachment — Submitted filename: Responses to Reviewers.docx [file pone.0274302.s008.docx]

Date: 14 June, 2022

To

Ivan D. Florez, MD, MSc, PhD

Academic Editor, PLOS ONE

From:

Dr. Abu Sadat Mohammad Sayeem Bin Shahid

Corresponding Author

**Subject:** Response to the comments of the reviewer of PLOS ONE on manuscript Ref: PONE-D-21-01326R2 titled “Digestive tolerability and acceptability of Fibersol-2 in healthy and diarrheal children 1-3 years old at a rural facility, Bangladesh: results from a four arm exploratory study**.**”

Dear Ivan D. Florez,

Thank you for evaluating our manuscript and providing us with the opportunity to submit the revised manuscript after addressing respected reviewer’s comments. We also express our sincere thanks to the reviewer for evaluating our manuscript. We are sending both the track change and clean versions of the manuscript that highlights the changes we have made from the previous version. We are also attaching this letter outlining a point-by-point response to each point kindly raised by the reviewer.

We hope that our response will be appropriate to qualify the manuscript for publication in your well-reputed journal.

We look forward to kindly hearing from you.

Thank you.

Reviewer#1 Thank you for addressing most of my concerns; however, I still find the paper a bit confusing in part and suggest the following:

When summarizing with the median and IQR, please indicate in text, e.g.: the median (IQR) duration of resolution… (l. 43).

**Response:** Thank you for the suggestion. It has been revised in line no.44, page no.3 in track change version.

l. 62. Did children in the diarrheal group have more bloating after the intervention than before? If so, I would question the conclusion of “well-tolerated.” I see that this is addressed in the Discussion but the lack of pre-intervention data should be mentioned here if you are going to talk about it with respect to the other symptoms.

**Response:** Thank you for the comment. It has been mentioned in line no.239-240, page no 12 and line no 291-293, page no.14 in track change version.

ll. 102-03. I believe that the authors are confusing tolerability with efficacy. Tolerability=any side effects of use? Efficacy=reduces symptoms. This should be clarified.

**Response:** We quite agree with your insight thoughts about tolerability. We have also examined whether there were any side effects occurred after the introduction of intervention drug. We admit that during pre-intervention period we failed to document the information of abdominal rumbling and bloating. As like as other features shown in table 3, it is expected that these were also present at enrolment and improved during the intervention as observed in all other features. On the other hand, as there were no new episodes of illness (side effect) and simultaneously all the documented features (available both in pre-intervention and during intervention) shown in table 3 improved during intervention, we may infer that the drug didn`t cause any side effects. Hope this clarifies.

l. 114. Should add …Fibersol-2 at two different doses…

**Response:** Thank you for the suggestion. It has been revised in line no.107, page no 5 in track change version.

l. 145. Reduction of symptoms is different from tolerability. This should be clarified. See above.

**Response:** Thank you for the comment. We quite agree with your insight thoughts about tolerability. We have also examined whether there were any side effects occurred after the introduction of intervention drug. We admit that during pre-intervention period we failed to document the information of abdominal rumbling and bloating. As like as other features shown in table 3, it is expected that these were also present at enrolment and improved during the intervention as observed in all other features. On the other hand, as there were no new episodes of illness (side effect) and simultaneously all the documented features (available both in pre-intervention and during intervention) shown in table 3 improved during intervention, we may infer that the drug didn`t cause any side effects. Hope this clarifies.

ll. 162-164. Here the outcome is stated in terms of symptoms, not efficacy. This needs to be cleaned up throughout the paper.

**Response:** Thank you for the comment. Again, we quite agree with the respected reviewer. It is one of the big limitations of our study that unfortunately we didn`t document the information on abdominal bloating and rumbling during the pre-intervention period but documented those only during the intervention period. Thus, we don`t know whether these are increased or decreased after the intervention. As the information on other features such as abdominal pain, abdominal distension, and vomiting are available and these symptoms decreased during the intervention, we can speculate that if the pre-intervention information of bloating and rumbling were available, these two might also be improved.

ll. 220. I think it would be clearer to call the groups: healthy children/high dose, healthy children/low dose, children with diarrhea/high dose, children with diarrhea/low dose

**Response:** Thank you for the suggestion. It has been revised accordingly (please see line no.31-32, page no.2, line no.137-140, page no.7, line no.221-222, page no. 11 and Table 1, page no.18 in track change version).

Discussion, ll. 268-273. This information is in the Results and should not be repeated here. The authors should state as a limitation the fact that no data on bloating or rumbling were available for the pre-intervention period, but that these were increased in the group of children with diarrhea after the intervention. Then the authors can discuss why they think this was not related to the intervention, if that is what they believe.

**Response:** Thank you for the comment and suggestion. It has been revised accordingly (please see line no. 261-269, page no. 13 in track change version). Again, we quite agree with the respected reviewer. It is one of the big limitations of our study that unfortunately we didn`t document the information on abdominal bloating and rumbling during the pre-intervention period but documented those only during the intervention period. Thus, we don`t know whether these are increased or decreased after the intervention. As the information on other features such as abdominal pain, abdominal distension, and vomiting are available and these symptoms decreased after intervention, we can speculate that if the pre-intervention information of bloating and rumbling were available, these two might also be improved. However, to eliminate any confusion, we have decided not to show these two features in the table 3 due to lack of their pre-intervention information.

Overall, the paper should be edited for grammar and coherence.

**Response:** Thank you for the suggestion. We have tried to improve the grammar and coherence in the revised version of the manuscript.
